# A structural mean modeling Mendelian randomization approach to investigate the lifecourse effect of adiposity: applied and methodological considerations

**DOI:** 10.1093/aje/kwaf029

**Published:** 2025-02-17

**Authors:** Grace M Power, Tom Palmer, Nicole Warrington, Jon Heron, Tom G Richardson, Vanessa Didelez, Kate Tilling, George Davey Smith, Eleanor Sanderson

**Affiliations:** MRC Integrative Epidemiology Unit, University of Bristol, Bristol, United Kingdom; Population Health Sciences, Bristol Medical School, University of Bristol, Bristol, United Kingdom; Institute for Molecular Bioscience, The University of Queensland, Brisbane, QLD, Australia; MRC Integrative Epidemiology Unit, University of Bristol, Bristol, United Kingdom; Population Health Sciences, Bristol Medical School, University of Bristol, Bristol, United Kingdom; Institute for Molecular Bioscience, The University of Queensland, Brisbane, QLD, Australia; Frazer Institute, University of Queensland, Woolloongabba, QLD, Australia; MRC Integrative Epidemiology Unit, University of Bristol, Bristol, United Kingdom; Population Health Sciences, Bristol Medical School, University of Bristol, Bristol, United Kingdom; MRC Integrative Epidemiology Unit, University of Bristol, Bristol, United Kingdom; Population Health Sciences, Bristol Medical School, University of Bristol, Bristol, United Kingdom; Statistical Methods in Epidemiology, Leibniz Institute for Prevention Research and Epidemiology - BIPS, Bremen, Germany; Department of Mathematics and Computer Science, University of Bremen, Bremen, Germany; MRC Integrative Epidemiology Unit, University of Bristol, Bristol, United Kingdom; Population Health Sciences, Bristol Medical School, University of Bristol, Bristol, United Kingdom; MRC Integrative Epidemiology Unit, University of Bristol, Bristol, United Kingdom; Population Health Sciences, Bristol Medical School, University of Bristol, Bristol, United Kingdom; NIHR Bristol Biomedical Research Centre Bristol, University Hospitals Bristol and Weston NHS Foundation Trust, University of Bristol, Bristol, United Kingdom; MRC Integrative Epidemiology Unit, University of Bristol, Bristol, United Kingdom; Population Health Sciences, Bristol Medical School, University of Bristol, Bristol, United Kingdom

**Keywords:** lifecourse, time-varying, genetic epidemiology, causal inference methods, adiposity

## Abstract

Mendelian randomization (MR) is a technique that uses genetic variation to address causal questions about how modifiable exposures influence health. For some time-varying phenotypes, genetic effects may have differential importance at different periods in the lifecourse. MR studies often employ conventional instrumental variable (IV) methods designed to estimate average lifetime effects. Recently, several extensions of MR have been proposed to investigate time-varying effects, including structural mean models (SMMs). SMMs exploit IVs through g-estimation and circumvent some of the parametric assumptions required by other MR methods. In this study, we applied g-estimation of SMMs within an MR framework to estimate the period effects of adiposity measured at two life stages, childhood and adulthood, on cardiovascular disease (CVD), type 2 diabetes (T2D), and breast cancer. We found persistent period effects of higher adulthood adiposity on increased risk of CVD and T2D. Higher childhood adiposity had a protective period effect on breast cancer risk. We compared this approach with an inverse variance weighted multivariable MR method, which also uses multiple IVs to assess time-varying effects but relies on a different set of assumptions. We highlight the strengths and limitations of each approach and conclude by emphasizing the importance of underlying methodological assumptions in the application of MR to lifecourse research.

## Introduction

Mendelian randomization (MR) exploits the random assortment of genetic variants, independent of other traits, to mitigate confounding bias and reverse causality.^1^ Within a causal inference framework, MR builds on instrumental variable (IV) analysis, whereby specific assumptions are required to hold.^2^ The IV must (1) be associated with the exposure of interest (“relevance”), (2) not share common causes with the outcome (“independence”), and (3) not affect the outcome other than through the exposure (“exclusion-restriction”).^3^^,^^4^ MR studies typically use genetic instruments associated with a phenotype, that are not specific to any particular time period in the lifecourse, to estimate the effect of an exposure on an outcome. The effect estimate obtained is therefore defined as a “lifetime” effect. However, this approach cannot disentangle whether early life exposures directly influence the outcome, whether their effect is mediated through later exposures, or if the observed effect arises from genetic variants acting on the exposure in later periods. Equally, it is unclear whether the exposure experienced in later periods affects the outcome directly, or if the effect is confounded by the exposure in an earlier period affecting the later period and outcome.

A lifecourse approach sets out to investigate the contribution of early and later life exposures to identify risk and protective mechanisms across the lifespan.^5^^-^^7^ It has been shown that genetic effects may have differential importance in the development of an exposure at different periods in the lifecourse.^8^^-^^12^ Therefore, “standard” MR designs may lead to incorrect interpretations.^13^ MR methods that rely on having multiple instruments with different effects on an exposure have been developed, or adapted, to address lifecourse research questions.^8^^,^^9^^,^^13^^-^^24^ These include inverse variance weighted (IVW) multivariable MR (IVW-MVMR) and MR using g-estimation of structural mean models (SMM-MR).^9^^,^^23^

SMM-MR exploits IVs through g-estimation.^25^^,^^26^ Structural mean models are semi-parametric, circumventing some parametric assumptions made by other MR methods such as the assumption of a linear relationship with a continuous outcome when using two-stage least-squares (2SLS).^27^ They comprise a wide array of models, including those with binary, categorical, and time-to-event outcomes. In a lifecourse context, three distinct types of causal parameters may be estimated using this approach if combined with IVs that satisfy specific assumptions: the point effect, period effect, and lifetime effect.^23^ Each of these effects represents the difference between expected outcomes in a population when an exposure is increased by one unit under different scenarios. Definitions and assumptions of the causal parameters targeted by SMM-MR approaches are summarized in Table 1.

**Table 1:** Definitions, mathematical expressions, and key assumptions for causal parameters using a structural mean modeling MR approach.

**Causal parameter**	**Definition**	**Mathematical expression** ^**a**^	**Key assumption(s)**	**Application example** ^**b**^
Point effect	The causal effect of a one-unit increase in the exposure at a single, specific time point on the outcome.	$\mathrm{Point}\ \mathrm{Effect}=$ $\mathrm{E}\left[{\mathrm{Y}}_{\mathrm{x}+1}\left({\mathrm{t}}_0\right)\right]-\mathrm{E}\left[{\mathrm{Y}}_{\mathrm{x}}\left({\mathrm{t}}_0\right)\right]$ Where: ${\mathrm{Y}}_{\mathrm{x}+1}\left({\mathrm{t}}_0\right)$ = counterfactual outcome if exposure is increased by one unit at time $\left({\mathrm{t}}_0\right);{\mathrm{Y}}_{\mathrm{x}}\left({\mathrm{t}}_0\right)$ = counterfactual outcome under the original exposure at the same time; and $\mathrm{E}\left[\mathrm{Y}\right]$ = expected population outcome^c^	The IV(s) must have a direct association with the exposure only at the time point considered, with no direct association with the exposure outside that point	To identify the point effect of the early life measure, we assume that the IVs being used are directly associated with adiposity at age 10 years only. For adulthood adiposity, the IVs being used are directly associated with adiposity at age 57 years only
Period effect	The causal effect of a constant one-unit increase in the exposure over a specified period of time on the outcome	$\mathrm{Period}\ \mathrm{Effect}=$ $\mathrm{E}\left[{\mathrm{Y}}_{\mathrm{x}+1}\left(\left(\mathrm{m}-\mathrm{p}\right):\mathrm{m}\right)\right]-$ $\mathrm{E}\left[{\mathrm{Y}}_{\mathrm{x}}\left(\left(\mathrm{m}-\mathrm{p}\right):\mathrm{m}\right)\right]$ Where: ${\mathrm{Y}}_{\mathrm{x}+1}\left(\left(\mathrm{m}-\mathrm{p}\right):\mathrm{m}\right)$ = counterfactual outcome if exposure is increased by at each time point within the specified period from $\mathrm{m}-\mathrm{p}\ \mathrm{to}\ \mathrm{m};{\mathrm{Y}}_{\mathrm{x}}\left(\left(\mathrm{m}-\mathrm{p}\right):\mathrm{m}\right)$ = counterfactual outcome under the original exposure at each time point within the specified period; and $\mathrm{E}\left[\mathrm{Y}\right]$ = expected population outcome^c^	This can be estimated with one measure of the exposure if the IVs have the same effect on the exposure throughout that specific period, and no direct effect on the exposure outside that period.	To interpret the early life measure as a period effect, we assume that early life adiposity represents the preadolescent period (e.g., ages ~9-12 years) and that the IVs exert a constant effect during this time. For adulthood adiposity, we assume the IVs exert a constant effect on adiposity in the middle-late adulthood period (e.g., ages ~40-65 years)
Lifetime effect	The causal effect of a one-unit increase across the entire lifecourse (defined as starting at conception and ending at the outcome recording time)	$\mathrm{Lifetime}\ \mathrm{Effect}=$ $\mathrm{E}\left[{\mathrm{Y}}_{\mathrm{x}+1}\left(0:\mathrm{T}\right)\right]-$$\mathrm{E}\left[{\mathrm{Y}}_{\mathrm{x}}\left(0:\mathrm{T}\right)\right]$ Where: $\mathrm{E}\left[{\mathrm{Y}}_{\mathrm{x}+1}\left(0:\mathrm{T}\right)\right]$ = counterfactual outcome if exposure is increased by one unit at each time point continuously from time 0 (conception) to time T (the outcome measurement time); $\mathrm{E}\left[{\mathrm{Y}}_{\mathrm{x}}\left(0:\mathrm{T}\right)\right]$ = counterfactual outcome under the original exposure at each time point within the specified period; and $\mathrm{E}\left[\mathrm{Y}\right]$ = expected population outcome^c^	This can be estimated with one measure of the exposure if the IVs exert a uniform and constant influence across the entire lifespan, from conception to the time of outcome measurement	To interpret a lifetime effect, we assume that the IVs influence adiposity consistently across the entire lifecourse, from conception to the point of outcome measurement in adulthood

Abbreviations: IV, instrumental variable; MR, Mendelian randomization.

a The mathematical expression used here is on the risk difference scale.

b For the data application example, we consider the effect of adiposity measured at around age 10 years and around age 57 years on later life outcomes.

c The subscript $x+1$for $Y$ indicates the scenario where the exposure level is increased by one unit, whereas the subscript $x$ represents the scenario under the original exposure level.

IVW-MVMR offers an alternative approach to conduct analysis in a lifecourse setting.^9^ IVW-MVMR estimates the controlled effects of correlated exposures on an outcome, where each effect is controlled for other exposures included in the model.^9^^,^^16^ The method relies on three assumptions, which expand on the core MR assumptions highlighted above: (1) the “relevance” assumption is modified to indicate that the liability (an underlying normally distributed latent (unmeasured) variable) to each exposure is robustly predicted by the genetic variants controlled for other exposures included in the estimation; (2) the “independence” assumption remains unchanged; and (3) the “exclusion-restriction” assumption proposes genetic variants are not associated with the outcome other than via liabilities to exposures included in the estimation.^9^^,^^28^ In a lifecourse setting, the correlated exposures in IVW-MVMR correspond to repeated measures of the same exposure over time.

**Figure 1:**
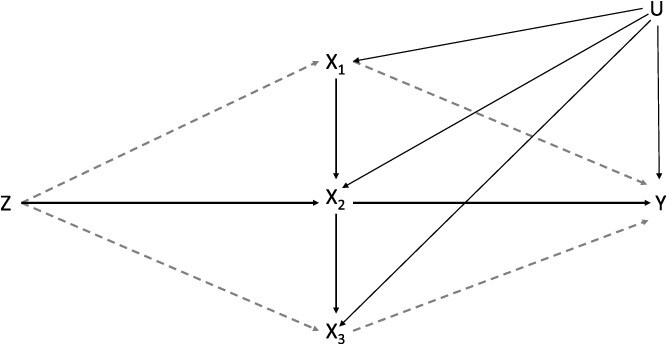
Causal diagram for IV analyses with one measured period (*X*_**2**_) (using instruments associated with three potential time periods) of an exposure. *Z* is a set of genetic variants associated with *X*_**2**_, *Y* is an outcome observed at one time only, *X*_**1**_ and *X*_**3**_ indicate potentially relevant periods that are unmeasured. *U* is a set of unobserved confounders of the exposure at each time period and the outcome. *X*_**1**_, *X*_**2,**_  *X*_**3**_, and *Y* are confounded by a set of unobserved confounders *U*. Dashed lines indicate paths via unmeasured exposures. Abbreviation: IV, instrumental variable.

Different assumptions about the nature of the association of the genetic variants used as instruments and the exposure at time periods not included in the model have been proposed for SMM-MR and IVW-MVMR. To satisfy the third instrumental condition when employing a SMM-MR design each component of the time-varying exposure outside of the period considered must not be directly associated with the instrument or the outcome. Under this assumption, using an example with an exposure measured at one time period (*X*_**2**_), there should be no direct pathway from genetic variants (*Z*) to *X*_**1**_ and *X*_**3**_ or no pathway from *X*_**1**_ or *X*_**3**_ directly to *Y* (see Figure 1). If multiple time periods had been measured and included in this model, the same assumptions would apply to those time periods included. This could be checked empirically.

**Figure 2:**
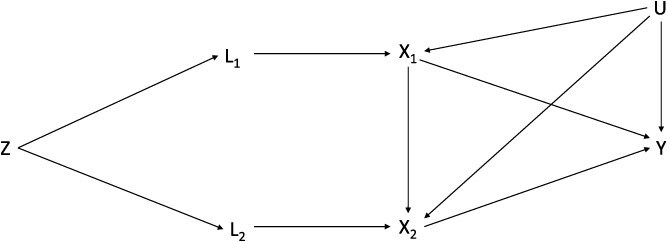
Causal diagram for IV analyses with two periods (using instruments associated with two time points assumed to represent two respective periods) of exposure (adapted from Sanderson et al.^9^). *L*_**1**_ is liability to the exposure in the earlier period; *L*_**2**_ is liability to the exposure in the later time period; and *Z* is a set of genetic variants associated with *L*_**1**_ and *L*_**2**_. *X*_**1**_ is a measure of the exposure in the early time period; *X*_**2**_ is a measure of the exposure at the second time period; *Y* is an outcome observed at one time only; and *U* is a set of unobserved confounders of the exposure at each time period and the outcome. The genetically predicted effect of increasing the exposure by a unit of liability at a particular time period will thus include genetic effects on the exposure at other time periods if the exposure in those periods has an effect on the outcome. If each genetic variant has a different trajectory for the liability, then the effects estimated will be an average across these. We have assumed that there is no time-varying confounding. Abbreviation: IV, instrumental variable.

Conversely, IVW-MVMR proposes that there may be a path from the instruments to the outcome via exposures at other periods, which have not been included in the estimation.^9^^,^^29^ This path will form part of the targeted causal effect. Consequently, the estimand being identified is the effect of an increase in one unit of liability to the exposure. With reference to Figure 2, the instruments associated with the liability (*L*) in at least one period of the lifecourse may be associated with *L* in different periods. The degree of this association may vary across periods.

IVW-MVMR has previously been used to evaluate whether childhood adiposity has an effect on disease outcomes, after controlling for adiposity in adulthood.^16^^,^^30^^-^^33^ If the main exposure (ie, childhood adiposity) has a causal influence on disease risk, IVW-MVMR allows us to decipher whether this effect is either partly mediated by, or is not acting through, adulthood adiposity. In this application, while increased childhood adiposity was a risk factor for coronary heart disease (CHD) and type 2 diabetes (T2D), the controlled period effects were negligible.^16^ This suggests that the influence of childhood adiposity on these diseases is mediated through adult adiposity, emphasizing the public health message that obesity prevention before adulthood could mitigate adverse effects.^34^ The study also found that increased childhood adiposity had a protective effect on breast cancer risk, independent of adult adiposity, and showed very little evidence of a causal effect on prostate cancer.^16^

In this paper we apply SMM-MR to estimate the period effects of childhood and adult adiposity on cardiovascular disease (CVD), T2D, and breast cancer in later life. We use this application to demonstrate this approach and provide a comprehensive interpretation of results. We compare results using the same data in an IVW-MVMR framework. We then discuss plausible next steps for MR methods used to address lifecourse epidemiology questions.

## Methods

### Data sources

UK Biobank data were collected between 2006 and 2010 on individuals aged between 40 and 69 years old at baseline, from clinical examinations, assays of biological samples, detailed information on self-reported health characteristics, and genome-wide genotyping, using a prospective cohort study design.^35^ The childhood adiposity measure applied in this study used recall questionnaire data, involving responses from adult participants who were asked whether, compared to the average, they were “thinner,” “about average,” or “plumper,” when they were aged 10 years old. The adult adiposity variable was derived using clinically measured body mass index (BMI) data (mean age, 56.5 years). It was then separated into a 3-tier variable based on the same proportions as the early life adiposity variable (ie, thinner, plumper, and about average).^36^^,^^37^ This was to ensure that derived effect estimates from subsequent analyses were as comparable as possible. Individuals that did not have data for both childhood and adult adiposity were excluded from analyses. Genetic variants strongly associated with childhood and adult adiposity (using *P* < 5 × 10^−8^ and *r*^2^ < 0.001) were identified in a large-scale genome-wide association study (GWAS), previously undertaken on 463 005 individuals in the UK Biobank study, adjusting for age, sex, and genotyping chip.^35^^,^^38^ These instruments have been independently validated in three distinct cohorts, validating their reliability in measuring childhood and adult adiposity.^16^^,^^36^^,^^37^ Phenotypic data for the outcomes under investigation, CVD, T2D, and breast cancer, were obtained from the UK Biobank. These outcomes were classified using the *International Classification of Diseases*, *Tenth Revision* (*ICD*-10) codes, described in Table S1. The UK Biobank study have obtained ethics approval from the Research Ethics Committee (REC; approval number: 11/NW/0382) and informed consent from all participants enrolled in UK Biobank. Estimates were derived using data from the UK Biobank (app #76538).

### Statistical analysis

First, we employed a SMM technique in a univariable framework using polygenic risk scores (PRSs) generated from selected genetic variants to estimate period effects of childhood and adulthood adiposity on CVD, T2D, and breast cancer. We then applied SMMs in a multivariable framework to compare with an IVW-MVMR approach used to estimate controlled period effects. For comparative purposes, we employed the IVW method to conduct MR analyses using summary statistics generated with the same sets of genetic variants used in the SMM analyses. These steps are described in further detail below.

### Polygenic risk scores

We calculated PRSs for each individual in the study using PLINK 2.00.^39^ Polygenic risk scores were generated with varying thresholds of association stringency between single nucleotide polymorphisms (SNPs) and the exposure phenotypes, childhood and adulthood adiposity, based on SNPs identified in the GWAS described above. The specific criteria for each PRS, including the SNP association thresholds, are detailed in Table 2. Complete lists of SNPs included in each PRS are available in Tables S2A-S2N.

**Table 2:** Details of the SNP association thresholds used for an MR analysis.

**Exposure**	**SNP threshold criteria**	**Stringency rating**	**Number of SNPs**
Childhood adiposity	The genetic variants strongly associated^a^ with childhood adiposity, regardless of their association with adulthood adiposity	Low	255
Adulthood adiposity	The genetic variants strongly associated^a^ with adulthood adiposity, regardless of their association with childhood adiposity	Low	514
Childhood adiposity	The genetic variants strongly associated^a^ with childhood adiposity and exclude SNPs that are associated with adulthood adiposity at *P* ≤ 5 × 10^−8^	Low-medium	180
Adulthood adiposity	The genetic variants strongly associated^a^ with adulthood adiposity and exclude SNPs that are associated with childhood adiposity at *P* ≤ 5 × 10^−8^	Low-medium	439
Childhood adiposity	The genetic variants strongly associated^a^ with childhood and exclude SNPs associated with adulthood adiposity at *P* ≤ .05 with Bonferroni correction^b^	Medium-high	106
Adulthood adiposity	The genetic variants strongly associated^a^ with adulthood adiposity and exclude SNPs associated with childhood adiposity at *P* ≤ .05 with Bonferroni correction^b^	Medium-high	365
Childhood adiposity	The genetic variants strongly associated^a^ with childhood adiposity and exclude SNPs associated with adulthood adiposity at *P* ≤ .05	High	55
Adulthood adiposity	The genetic variants strongly associated^a^ with adulthood adiposity and exclude SNPs associated with childhood adiposity at *P* ≤ .05	High	218

Abbreviations: MR, Mendelian randomization; SNPs, single nucleotide polymorphisms.

a Strongly associated refers to associated at genome wide significance (*P* ≤ 5 $\times$ 10^−8^).

b Genetic variants strongly associated with childhood adiposity (excluding variants associated with adulthood adiposity at *P* ≤ .05) and those strongly associated with adulthood adiposity (excluding variants associated with childhood adiposity at *P* ≤ .05) were counted. We then divided 0.05 by this number to generate the Bonferroni corrected *P* value.

Genetic IVs in a MR setting are conventionally selected from an independent dataset where the sample does not overlap with the dataset being analyzed to avoid overfitting bias.^40^ Conducting MR using overlapping sets of participant samples may bias in the direction of the conventional observational results between the exposure and outcome.^41^ In our investigation it was not possible to avoid this. However, we compare our IVW-MVMR results to the same analysis using non-overlapping outcome data, replicating analyses previously conducted,^16^ to assess how using these data may have affected our results. For this analysis GWAS data for CHD, T2D, and breast cancer were obtained from large scale consortia, which did not include data from the UK Biobank.^42^^-^^44^ Details on these datasets have been described previously.^16^

### MR using structural mean modeling

We used individual-level data to perform SMM-MR. To highlight results obtained in most “standard” MR analyses we first used genetic variants strongly associated with adiposity around age 10 years (*P* ≤ 5 × 10^−8^), regardless of their association with adulthood adiposity and genetic variants strongly associated with adiposity around 57 years, regardless of their association with childhood adiposity. These results are interpreted as period effects as we have assumed that the genetic variants are only directly associated with the exposure over the periods of childhood or adulthood. However, under an alternative assumption in which associations between genetic variants and adiposity remain constant throughout the lifecourse, they could be interpreted as lifetime effects. Next, we used the genetic variants strongly associated with adiposity around age 10 years (*P* ≤ 5 × 10^−8^), excluding SNPs strongly associated with adiposity around 57 years at *P* ≤ 5 $\times$ 10^−8^, to estimate the period effect of childhood adiposity for a period that started in childhood and ended at some point between 10 and 57 years. We used the genetic variants strongly associated with adiposity around age 57 years (*P* ≤ 5 × 10^−8^), excluding SNPs associated with childhood adiposity at *P* ≤ 5 $\times$ 10^−8^, to estimate the period effect of adulthood adiposity for a period that started at some point between 10 and 57 years up to the point the outcome is measured. While definition of these periods is imprecise, the interpretation of this causal parameter is supported by previous validation studies.^16^^,^^36^^,^^37^ These have demonstrated that the genetic variants strongly associated with adiposity at age 10 years were better predictors of childhood BMI than those predicting adulthood BMI. In late adolescence, neither set of variants optimally predicted BMI, but in adulthood, variants associated with adiposity at age 57 years were stronger predictors of this timeframe than early life variants.^16^^,^^36^^,^^37^ We recommend conducting comparable validation analyses for novel variants utilized in subsequent studies. For instance, if data permit, examining the phenotypic associations of these novel variants across various age groups could help clarify which assumptions are most plausible.

To further explore the impact of instrument selection when estimating period effects, we applied the SNP association thresholds outlined in Table 2 and ran models with the PRS generated from each of these, in turn. We additionally applied SMMs in a multivariable framework to calculate the effects of childhood adiposity and adulthood adiposity, simultaneously, on CVD, T2D, and breast cancer. This approach estimates the controlled period effect of an exposure at a specific period by controlling for the exposure at a period not considered the main exposure period. The MR analyses conducted and their interpretation, along with the closest equivalent IVW estimator, are summarized in Table 3.

**Table 3:** Presentation of the constituent components of SMM-MR, with the comparative interpretations and assumptions made when using IVW-MR.

**SMM/IVW effect**	**Alternative MR estimator**	**Exposure**	**Variables controlled for**	**Methodological assumptions being made using SMM-MR**	**Interpretation of the estimand (under assumptions) identified using SMM-MR**	**Methodological assumptions being made using IVW-MR**	**Interpretation of the estimand (under assumptions) identified using IVW-MR**
Point	Univariable MR	Adiposity at a specific time point		Genetic variants strongly associated with adiposity at a specific time point that do not have an effect through any other time point	The effect of adiposity at one specific time point on the outcome	--^a^	--^a^
Period (varies depending on SNP association stringency)	Univariable MR	Childhood adiposity		Genetic variants strongly associated with childhood adiposity do not have an effect through another time period, such as adulthood adiposity	The effect of childhood adiposity around the period childhood adiposity was measured, independent of other time periods	Genetic variants strongly associated with childhood adiposity may have an effect through another time period, such as adulthood adiposity. Genetic variants have different effects at different time periods	The effect of liability to childhood adiposity at around the age childhood adiposity was measured
Period (varies depending on SNP association stringency)	Univariable MR	Adulthood adiposity		Genetic variants strongly associated with adulthood adiposity do not have an effect through another time period, such as childhood adiposity	The effect of adulthood adiposity around the period adulthood adiposity was measured, independent of other time periods	Genetic variants strongly associated with adulthood adiposity may have an effect through another time period, such as childhood adiposity. Genetic variants have different effects at different time periods	The effect of liability to adulthood adiposity at around the age adulthood adiposity was measured
Controlled period	Multivariable MR	Childhood adiposity, controlled for adulthood adiposity	Adulthood adiposity	Genetic variants have different effects at the different time periods considered	The controlled effect of childhood adiposity including pathways comprising adiposity measures from different timeframes in the lifecourse, controlled for adiposity measures from other time periods included	Genetic variants have different effects at different time periods	The controlled period effect of liability to childhood adiposity including pathways comprising adiposity measures from different timeframes in the lifecourse, controlled for adiposity measures from other time periods included
Controlled period	Multivariable MR	Adulthood adiposity, controlled for childhood adiposity	Childhood adiposity	Genetic variants have different effects at the different time periods considered	The controlled effect of adulthood adiposity including pathways comprising adiposity measures from different timeframes in the lifecourse, controlled for adiposity measures from other time periods	Genetic variants have different effects at different time periods	The controlled period effect of liability to adulthood adiposity including pathways comprising adiposity measures from different timeframes in the lifecourse, controlled for adiposity measures from other time periods
Lifetime	Univariable MR	Adiposity over the entire lifecourse		Genetic variant associations with adiposity remain constant over the lifecourse	The effect of adiposity across the entire lifecourse on an outcome	--	The effect of liability to adiposity over the entire lifecourse on an outcome

Abbreviations: IVW, inverse variance weighted; MR, Mendelian randomization; SNP, single-nucleotide polymorphism.

a Estimation of the point effect IVW-MR is technically plausible; however, we do not recommend this. Assumptions and interpretation would be the same as with SMM-MR.

Importantly, childhood adiposity strongly influences adulthood adiposity.^45^ Genetic variants with varying levels of association with childhood and adult adiposity may be targeting different causal pathways underlying the relationship between adiposity and complex outcomes that vary in importance at different life stages.^46^ For instance, some variants linked to adult adiposity might affect aspects of early-life adiposity that childhood-associated variants do not capture.^46^ This can lead to deviations from the gene–environment equivalence assumption in MR,^47^ which asserts that genetic perturbations should mirror the effects of modifiable exposures on outcomes.^46^ This is important to consider when selecting genetic variants to instrument period effects.

### Genome-wide association studies

We conducted GWAS to assess associations between genetic variants and the three outcomes of interest: CVD, T2D, and breast cancer. These analyses were performed using BOLT-LMM, a Bayesian linear mixed model that accounts for relatedness and population stratification when estimating associations between genetic variants and a trait.^48^^,^^49^ Age at baseline, sex, and genotyping array type were included as covariates in the model. The resulting GWAS were subsequently used in the IVW-MR analyses.

### MR using inverse variance weighting

We employed the IVW method on outcomes from two data sources.^50^ The inverse variance weighted approach takes SNP-outcome estimates and regresses them on the SNP-exposure associations, weighting each SNP by the inverse of the variance of its SNP-outcome association.^51^ First, we used the GWAS generated from UK Biobank data to compare against the MR analyses using SMMs. Second, we used the GWAS obtained from large scale consortia, which did not include data from the UK Biobank,^42^^-^^44^ to assess how using overlapping outcome data may have affected our results. For each of the sets of IVW analyses, we estimated the “total” effects in a univariable framework. We then ran MVMR analyses to estimate the controlled period effect.

The IVW-MR analyses were conducted using the TwoSampleMR R package.^52^ Plots in this paper were generated using the R package “ggplot2.”^53^ These analyses were undertaken using R (version 3.5.1).

## Results

**Figure 3:**
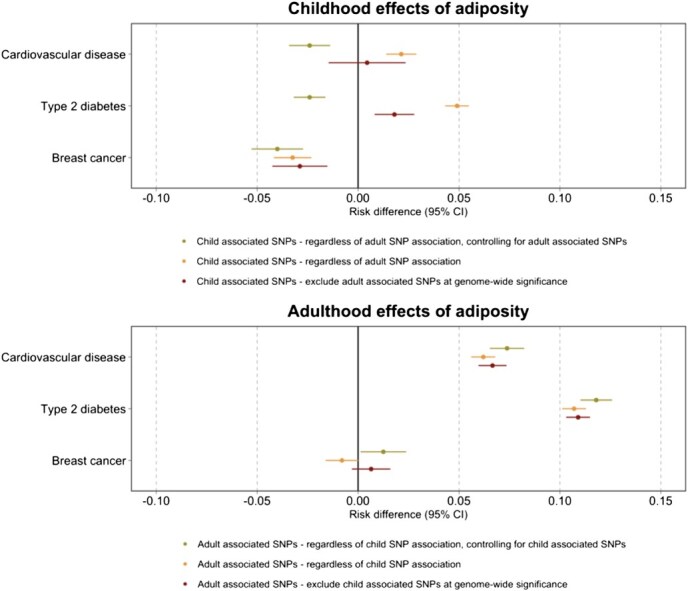
Causal risk difference estimates from univariable and multivariable MR using structural mean models (SMMs) for childhood and adulthood adiposity period effects on the outcome measures listed. Associated SNPs refers to strongly associated at genome wide significance (*P* ≤ 5 × 10^−8^).

Univariable SMM-MR analyses, representing a period effect, indicated that higher childhood adiposity (regardless of SNP association with adulthood adiposity) increased CVD and T2D risk (risk difference (RD), 95% CI: 0.021, 0.013 to 0.029 and 0.049, 0.043 to 0.054, respectively) as did higher adult adiposity (RD, 95% CI: 0.062, 0.056 to 0.068 and 0.107, 0.101 to 0.113, respectively; Figure 3, Tables S3A and S3B). There was minimal evidence for period effects of higher childhood adiposity (excluding SNPs associated with adulthood adiposity at *P* ≤ 5 × 10^−8^) on increased CVD risk and some evidence on T2D risk (RD, 95% CI: 0.004, −0.008 to 0.016 and 0.018, 0.008 to 0.028, respectively). There was strong evidence of a period effect of higher adult adiposity (excluding SNPs associated with childhood adiposity at *P* ≤ 5 × 10^−8^) on increased CVD and T2D risk (RD, 95% CI: 0.067, 0.059 to 0.075 and 0.109, 0.103 to 0.115, respectively). Under the alternative assumption that the associations between genetic variants and adiposity remain constant throughout the lifecourse these estimates could be interpreted as lifetime effects.

SMM-MVMR analyses showed a controlled period effect of higher childhood adiposity on reduced CVD and T2D risk (RD, 95% CI: −0.024, −0.034 to −0.014, and −0.024, −0.032 to −0.016, respectively). There was a controlled period effect of higher adult adiposity on the CVD and T2D risk in later life (RD, 95% CI: 0.074, 0.066 to 0.082 and 0.118, 0.110 to 0.126, respectively).

Univariable SMM-MR analyses indicated that higher childhood adiposity (regardless of SNP association with adulthood adiposity) reduced breast cancer risk (RD, 95% CI: −0.032, −0.042 to −0.022; Figure 3; Table S3C). There was evidence of a period effect of higher childhood adiposity (excluding SNPs associated with adulthood adiposity at *P* ≤ 5 × 10^−8^) on reduced breast cancer risk (RD, 95% CI: −0.029, −0.043 to −0.015). There was minimal evidence of a period effect of higher adult adiposity (excluding SNPs associated with childhood adiposity at *P* ≤ 5 × 10^−8^) on breast cancer (RD, 95% CI: 0.006, −0.004 to 0.016). SMM-MVMR analyses also showed evidence that higher childhood adiposity reduced the risk of breast cancer, after controlling for adult adiposity (RD, 95% CI: −0.040, −0.054 to −0.026), while higher adult adiposity slightly increased breast cancer risk, controlling for childhood adiposity (RD, 95% CI: 0.013, 0.001 to 0.025).

**Figure 4:**
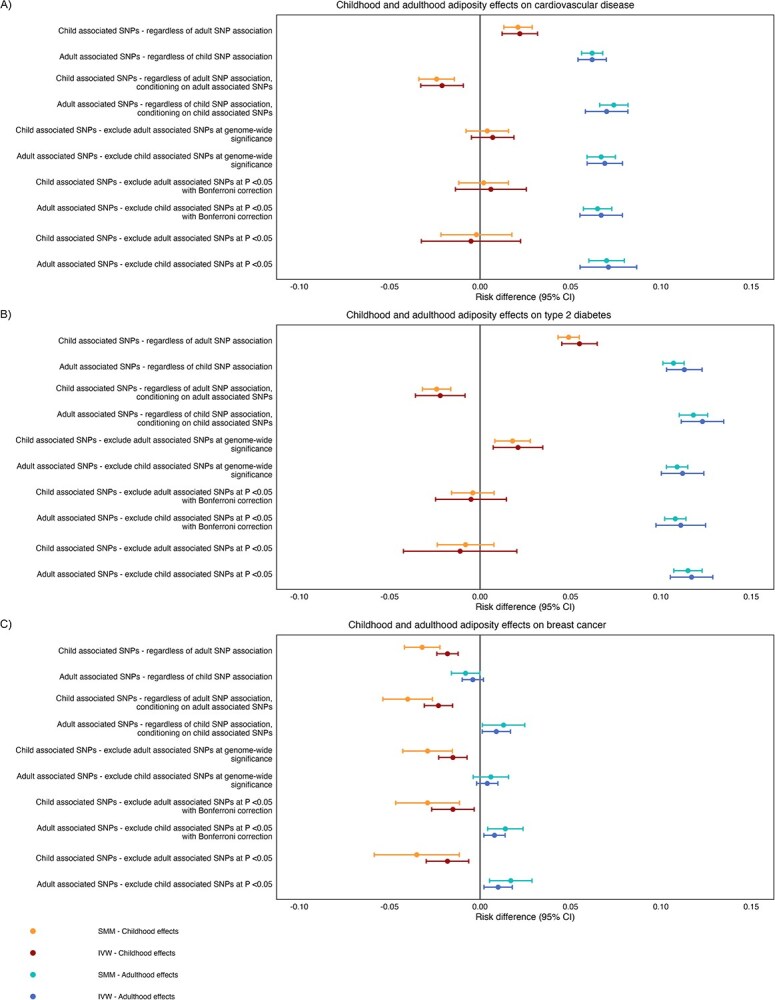
Causal risk difference estimates from univariable and multivariable MR using structural mean models (SMMs) and inverse variance weighted (IVW) models for childhood and adulthood adiposity, and liability to childhood and adult adiposity, respectively, on the outcome measures listed. (A) Childhood and adult adiposity period effects on cardiovascular disease (CVD). (B) Childhood and adult adiposity period effects on type 2 diabetes (T2D). (C) Childhood and adult adiposity period effects on breast cancer. Associated SNPs refers to strongly associated at genome wide significance (*P* ≤ 5 × 10^−8^).

When using different SNP association thresholds to estimate period effects, univariable SMM-MR consistently showed that that higher adulthood adiposity increased risk of CVD (Figure 4A; Table S3A) and T2D (Figure 4B; Table S3B). As the stringency of SNP selection increased, excluding increasing number of SNPs associated with adult adiposity, evidence of the effect of childhood adiposity on CVD diminished. Some evidence of an effect on T2D persisted using SNPs with the lowest stringency (exclusion threshold of adulthood adiposity SNPs at *P* ≤ 5 × 10^−8^).

Additionally, analyses consistently showed that higher childhood adiposity reduced breast cancer risk (Figure 4C; Table S3C). A protective effect of adulthood adiposity was observed when including all SNPs, but this effect diminished or reversed direction when excluding SNPs associated with childhood adiposity.

IVW univariable and MVMR analyses using UK Biobank data, showed similar trends to the SMM approach, though generally weaker (Figure 4A-C; Tables S4A-S4C). Inverse variance weighted univariable and MVMR analyses using the outcomes generated from the large-scale consortia data indicated effects in the same directions as the SMM approach (Tables S5A-S5C; Figure S1A-C), though with larger magnitude and broader confidence intervals, likely due to Winner’s curse from sample overlap between the discovery sample and dataset used in core analyses.^54^ Specifically, UK Biobank data indicated that increased adulthood adiposity increased breast cancer risk (Figure 4C; Table S4C). In contrast, there was minimal evidence of an effect of adulthood adiposity on breast cancer when using the large scale meta-analyzed outcome consortia data (Table S5C; Figure S1C).

## Discussion

In this investigation we apply MR within a lifecourse epidemiological framework, using SMM and IVW approaches with a time-varying exposure. SMM-MR and IVW-MVMR target different causal estimands that rely on distinct assumptions. SMM-MR computed the effect of increasing adiposity by one unit of measurement and IVW-MVMR, the effect of an increase in one unit of liability to adiposity. For SMM-MR, each component of the time-varying exposure outside of the period considered is assumed to be unaffected by the instrument or affect the outcome only by affecting exposures during the time period under consideration. For IVW-MVMR, the genetically predicted effect of increasing the exposure by a unit of liability at one time period is assumed to include genetic effects via the exposure at other time periods if the exposure in those periods has an effect on the outcome.

We estimated the effects of adiposity on CVD, T2D, and breast cancer. First, we evaluated the period effects of childhood and adult adiposity using genetic variants strongly associated with adiposity at each period, regardless of their association with adiposity at the alternate period. We then evaluated stringently defined period effects and finally controlled period effects.

Our findings indicated that the period effects of higher childhood and adult adiposity, when using less stringent SNP-association criteria, increased the risk of CVD and T2D, while reducing the risk of breast cancer. However, when applying stricter SNP-association criteria, the effect of higher childhood adiposity on CVD and T2D was attenuated, while controlled period effects indicated a reduced risk associated with higher childhood adiposity for these outcomes. Conversely, more stringently instrumented period effects, as well as controlled period effects, indicated that higher adulthood adiposity may elevate breast cancer risk. Our findings suggest that higher childhood adiposity was protective against breast cancer across both period and controlled period estimates, aligning with previous research.^33^ Interpretation of these results as period effects depends on the assumption that the genetic variants are associated with adiposity only for the period in which they were identified. Under the alternative assumption that the associations between genetic variants and adiposity remain constant throughout the lifecourse these could be interpreted as lifetime effects.

Our results highlight the importance of considering potential associations of the genetic variants with traits at other time periods when estimating period effects. For example, we see evidence of increased period effects on T2D in the set of SNPs strongly associated with childhood adiposity, irrespective of their association with adult adiposity, as well as in SNPs not associated with adulthood adiposity at *P* ≤ 5 $\times$ 10^−8^. However, increasing the stringency for SNP inclusion in childhood instruments showed little evidence of an effect of childhood adiposity on T2D. Furthermore, SNPs used to instrument adiposity at one period might still be associated with adiposity at another period, albeit with a lower level of association. Since our analysis includes only two time points, we cannot determine SNP associations with other time periods beyond those measured.

When researchers use genetic instruments that are not specific to a time period, which is commonplace in MR analyses, they are implicitly targeting a lifetime effect of liability to a unit higher level of the exposure at the time it was measured.^29^ This may result in incorrect interpretations as highlighted in our applied examples. To allow estimation of period-specific effects, we can also use a SMM- or IVW-MVMR approach, to estimate the controlled period effect of each exposure on the outcome. Results may still reflect the effects of other periods that are not included in the estimation if they are influenced by the genetic variants used.

We have assumed throughout that there is no time-varying confounding; however, the existence of time-varying confounding would not affect the interpretation of our results. A detailed explanation for this is provided in Table S6. We have also assumed all models are linear and that there are no interactions between the exposure at different time periods.

The IVW-MVMR results generated from largescale consortia indicated little evidence of a controlled effect of higher adult adiposity on breast cancer.^16^ The observed risk increase using the UK Biobank data could be due to higher adulthood adiposity influencing onset and survival from breast cancer differently in this cohort study compared to the case–control studies used in producing the largescale outcome data. Differences may also be partially due to a “healthy volunteer” selection bias. The UK Biobank is not representative of the sampling population—participants are a sample of volunteers with healthier lifestyles, higher levels of education and better health than the general UK population.^55^^,^^56^ If participation in the UK Biobank is a consequence of either our exposure or outcome, then a key MR condition (exchangeability) is violated, inducing an association between genetic instruments and unmeasured confounders of the exposure–outcome relationship.^56^ Another reason for inconsistencies could be weak instrument bias. This may push a null effect to be non-null, as seen in the effect between adulthood adiposity and breast cancer risk.^57^ In addition, when exposure and outcome effects are estimated in a single (ie, fully overlapping) sample, as they were for our analyses, bias will be in the direction of the (confounded) observational association.^58^

There is no meaningful difference between the estimation results obtained using SMM and IVW-MR in relatively simple models such as the one we have used; however, the importance of this work lies in both the clear explanation of the interpretations of the estimates from each method, and detailed assumptions being made when employing each method. Comprehensively understanding the interpretation of results after employing these methods is of key public health importance. For instance, when considering the potential differences in mediating pathways from one period (ie, childhood adiposity) and not another (ie, adulthood adiposity) we might hypothesize that prepubertal childhood and adult adiposity could be distinct phenotypes; an insight gained through conducting these approaches. This helps us to further unpick pathways associated with disease risk. For example, there are long-run effects of childhood adiposity on physical development shown though mammographic density being a strong mediator for the protective effects of childhood adiposity on breast cancer.^59^ In addition, situations in which SMMs may prove more useful, specifically when addressing lifecourse questions, should be explored in future work.

This study provides important detail on novel MR methods available to perform lifecourse research; however, an important limitation lies the limited availability of period-specific instruments. This may be attributed to the genetic associations of many phenotypes remaining constant over time as well as the lack of research investigating potential temporal variations in these associations. Compounding this is the absence of adequately large datasets that include age-specific information paired with corresponding genetic data across a wide range of phenotypes. Such data have the potential to greatly enhance our understanding of this research area. In cases where genetic associations do not vary over time, researchers seeking to estimate period effects might instead identify examples of period-specific non-genetic instruments and use alternative methods such as time-defined natural experiments.

This study underscores the importance of carefully considering underlying methodological assumptions when applying SMM-MR and IVW-MVMR approaches in a lifecourse setting and offers guidance on accurately interpreting the results generated by these methods. It also highlights the critical role of instrument selection in these analyses. A clear and informed understanding of the assumptions and interpretations is essential for conducting robust and meaningful MR analyses in lifecourse research—considerations that have yet to be fully explored in the current literature. We encourage practitioners to thoroughly think through their research question, IV selection, model conditions, and data availability before pursuing a particular MR approach within a lifecourse setting.

## Research ethics and informed consent

The UK Biobank study have obtained ethics approval from the Research Ethics Committee (REC; approval number: 11/NW/0382). The UK Biobank study have obtained informed consent from all participants enrolled in UK Biobank. Estimates were derived using data from the UK Biobank (app #76538).

## Supplementary Material

Web_Material_kwaf029

## Data Availability

Individual-level data used to derive these results can be obtained with an approved application to the UK Biobank study (http://www.ukbiobank.ac.uk). All genetic instruments derived in this study are in Tables S2A-S2N. Outcome estimates for summary-level sensitivity analyses were obtained using publicly accessible data made accessible by genome-wide association study consortiums. Template code generated to run the main analyses outlined in this article are available on GitHub (https://github.com/gracemarionpower/SMM-MR/). Code used to undertake IVW Mendelian randomization analyses can be found as part of the “TwoSampleMR” R package.
