# Supplementary material for: A structural mean modeling Mendelian randomization approach to investigate the lifecourse effect of adiposity: applied and methodological considerations
Source: Am J Epidemiol. 2025 Feb 17;195(1):21–31. doi: 10.1093/aje/kwaf029 (PMC12780781; doi:10.1093/aje/kwaf029)
Supplement: Web_Material_kwaf029 [file web_material_kwaf029.zip › Supplementary_aje.docx]

**Supplementary Material**

**Title:** A structural mean modelling Mendelian randomization approach to investigate the lifecourse effect of adiposity: applied and methodological considerations.

Grace M. Power^1,2,3*^, Tom Palmer^1,2^, Nicole Warrington^3,4^, Jon Heron^1,2^, Tom G. Richardson^1,2^, Vanessa Didelez^5,6^, Kate Tilling^1,2^, George Davey Smith^1,2,7^, Eleanor Sanderson^1,2^

*^1^ MRC Integrative Epidemiology Unit, University of Bristol, Bristol, UK*

*^2^ Population Health Sciences, Bristol Medical School, University of Bristol, Bristol, UK
^3^ Institute for Molecular Bioscience, The University of Queensland, Brisbane, Queensland, Australia*

*^4^ Frazer Institute, University of Queensland, Woolloongabba, Queensland, Australia*

*^5^ Leibniz Institute for Prevention Research and Epidemiology - BIPS, Bremen, Germany
^6^ Department of Mathematics and Computer Science, University of Bremen, Germany*

*^7^ NIHR Bristol Biomedical Research Centre Bristol, University Hospitals Bristol and Weston NHS Foundation Trust, University of Bristol, Bristol, UK*

^*^ Corresponding author: Grace M Power - grace.power@bristol.ac.uk - +44 (0)117 331 0098
MRC Integrative Epidemiology Unit, University of Bristol, Oakfield House, Oakfield Grove, Bristol, BS8 2BN, United Kingdom

**Supplementary Tables.**

**Table S1.** Outcome definitions classified using International Classification of Diseases, Tenth Revision (ICD-10) codes.

**Table S2A.** The full set of genetic variants strongly associated with childhood adiposity.
**Table S2B.** The full set of genetic variants strongly associated with adulthood adiposity.
**Table S2C.** The genetic variants strongly associated with childhood adiposity at genome wide significance and not adulthood adiposity (exclude adult SNPs at P ≤ 5×10^-8^).
**Table S2D.** The genetic variants strongly associated with childhood adiposity at genome wide significance and not adulthood adiposity (exclude adult SNPs at P ≤ 5×10^-8^) in females.
**Table S2E.** The genetic variants strongly associated with adulthood adiposity at genome wide significance and not childhood adiposity (exclude child SNPs at P ≤ 5×10^-8^).
**Table S2F.** The genetic variants strongly associated with adulthood adiposity at genome wide significance and not childhood adiposity (exclude child SNPs at P ≤ 5×10^-8^) in females.
**Table S2G.** The genetic variants strongly associated with childhood adiposity at genome wide significance and not adulthood adiposity (exclude adult SNPs at P ≤ 0.05 with Bonferroni correction*).
**Table S2H.** The genetic variants strongly associated with childhood adiposity at genome wide significance and not adulthood adiposity (exclude adult SNPs at P ≤ 0.05 with Bonferroni correction*) in females.
**Table S2I.** The genetic variants strongly associated with adulthood adiposity at genome wide significance and not childhood adiposity (exclude child SNPs at P ≤ 0.05 with Bonferroni correction**).
**Table S2J.** The genetic variants strongly associated with adulthood adiposity at genome wide significance and not childhood adiposity (exclude child SNPs at P ≤ 0.05 with Bonferroni correction**) in females.
**Table S2K.** The genetic variants strongly associated with childhood adiposity at genome wide significance and not adulthood adiposity (exclude adult SNPs at P ≤ 0.05).
**Table S2L.** The genetic variants strongly associated with childhood adiposity at genome wide significance and not adulthood adiposity (exclude adult SNPs at P ≤ 0.05) in females.
**Table 2SM.** The genetic variants strongly associated with adulthood adiposity at genome wide significance and not childhood adiposity (exclude child SNPs at P ≤ 0.05)
**Table S2N.** The genetic variants strongly associated with adulthood adiposity at genome wide significance and not childhood adiposity (exclude child SNPs at P ≤ 0.05) in females. **Table S3A.** Univariable and multivariable Mendelian randomization (MR) analyses using structural mean modelling (SMM) for child and adult adiposity on cardiovascular disease (CVD) - using UKB outcome data.
**Table S3B.** Univariable and multivariable Mendelian randomization analyses using structural mean models for child and adult adiposity on type 2 diabetes (T2D) - using UKB outcome data.
**Table S3C.** Univariable and multivariable Mendelian randomization analyses using structural mean models for child and adult adiposity on breast cancer - using UKB outcome data.
**Table S4A.** Univariable and multivariable two-sample Mendelian randomization (MR) analyses using inverse probability weighting for child and adult adiposity on cardiovascular disease (CVD) - using UKB outcome data. **Table S4B.** Univariable and multivariable two-sample Mendelian randomization (MR) analyses using inverse probability weighting for child and adult adiposity on type 2 diabetes (T2D) - using UKB outcome data.
**Table S4C.** Univariable and multivariable two-sample Mendelian randomization (MR) analyses using inverse probability weighting for child and adult adiposity on breast cancer - using UKB outcome data.
**Table S5A.** Univariable and multivariable two-sample Mendelian randomization (MR) analyses using inverse probability weighting for child and adult adiposity on cardiovascular (CVD) – using large scale consortium data. **Table S5B.** Univariable and multivariable two-sample Mendelian randomization (MR) analyses using inverse probability weighting for child and adult adiposity on type 2 diabetes (T2D) – using large scale consortium data. **Table S5C.** Univariable and multivariable two-sample Mendelian randomization (MR) analyses using inverse probability weighting for child and adult adiposity on breast cancer – using large scale consortium data.
**Table S6.** Description on time-varying confounding in the context of this study.

**Supplementary Figures.**

**Figure S1.** Causal risk difference estimates from univariable and multivariable MR using inverse variance weighted models (IVW) for childhood and adult adiposity period effects on the outcome measures listed using large scale consortia data referenced in manuscript. (A) Childhood and adult adiposity period and lifetime effects on cardiovascular disease (CVD). (B) Childhood and adult adiposity period and lifetime effects on Type 2 diabetes (T2D). (C) Childhood and adult adiposity period and lifetime effects on breast cancer.

**Table S1.** Outcome definitions classified using International Classification of Diseases, Tenth Revision (ICD-10) codes.

| Measure | Definition | ICD-10 code(s) |
| --- | --- | --- |
| Cardiovascular disease (CVD) | Cardiovascular disease cases | I210-I229, I231-36, I238, I240, I241, I248-I256, I258, I259 |
| Type 2 diabetes (T2D) | Type 2 diabetes mellitus | E110-19 |
| Breast cancer | Malignant neoplasm of the breast | C500-06, C508-09 |

**Table S2A.** The full set of genetic variants strongly associated with childhood adiposity.

| SNP | Chromosome | Base position | Effect allele | Other allele | Beta (Adult) | SE (Adult) | P (Adult) | Beta (Age 10) | SE (Age 10) | P (Age 10) |
| --- | --- | --- | --- | --- | --- | --- | --- | --- | --- | --- |
| rs56094641 | 16 | 53806453 | A | G | -4.65E-02 | 1.40E-03 | 4.00E-243 | -4.73E-02 | 1.43E-03 | 1.60E-240 |
| rs543874 | 1 | 177889480 | A | G | -3.01E-02 | 1.69E-03 | 1.00E-70 | -4.71E-02 | 1.73E-03 | 6.00E-163 |
| rs10182458 | 2 | 25150641 | A | G | -1.99E-02 | 1.37E-03 | 4.10E-48 | -3.59E-02 | 1.40E-03 | 3.50E-145 |
| rs62106258 | 2 | 417167 | T | C | 5.91E-02 | 3.18E-03 | 4.20E-77 | 8.02E-02 | 3.25E-03 | 3.60E-134 |
| rs12992672 | 2 | 632592 | G | A | -3.60E-02 | 1.81E-03 | 1.20E-87 | -4.33E-02 | 1.85E-03 | 4.00E-121 |
| rs7132908 | 12 | 50263148 | G | A | -1.86E-02 | 1.41E-03 | 8.40E-40 | -3.13E-02 | 1.44E-03 | 1.60E-104 |
| rs58084604 | 18 | 57849429 | C | T | -3.52E-02 | 1.62E-03 | 2.60E-104 | -3.32E-02 | 1.66E-03 | 3.00E-89 |
| rs12042908 | 1 | 74997762 | A | G | 1.04E-02 | 1.38E-03 | 3.90E-14 | 2.75E-02 | 1.41E-03 | 2.50E-84 |
| rs12641981 | 4 | 45179883 | C | T | -1.87E-02 | 1.38E-03 | 1.90E-41 | -2.22E-02 | 1.42E-03 | 1.80E-55 |
| rs55880046 | 16 | 19941557 | T | G | 1.69E-02 | 1.96E-03 | 8.70E-18 | 2.99E-02 | 2.01E-03 | 3.90E-50 |
| rs115319174 | 2 | 207066474 | G | C | -1.13E-02 | 2.97E-03 | 1.40E-04 | -4.23E-02 | 3.04E-03 | 3.30E-44 |
| rs72892910 | 6 | 50816887 | G | T | -2.53E-02 | 1.82E-03 | 5.70E-44 | -2.47E-02 | 1.86E-03 | 3.20E-40 |
| rs11209943 | 1 | 72750500 | A | G | -1.41E-02 | 1.40E-03 | 6.60E-24 | -1.83E-02 | 1.43E-03 | 8.80E-38 |
| rs8030456 | 15 | 68076856 | C | T | 1.83E-02 | 1.64E-03 | 3.70E-29 | 2.13E-02 | 1.67E-03 | 2.80E-37 |
| rs35918296 | 8 | 76862208 | C | T | 1.02E-02 | 1.39E-03 | 2.90E-13 | 1.67E-02 | 1.42E-03 | 1.40E-31 |
| rs2238435 | 16 | 4014282 | C | G | -1.41E-02 | 1.41E-03 | 1.20E-23 | -1.65E-02 | 1.44E-03 | 3.00E-30 |
| rs7550711 | 1 | 110082886 | C | T | -4.26E-02 | 4.32E-03 | 5.10E-23 | -4.84E-02 | 4.41E-03 | 5.20E-28 |
| rs4744246 | 9 | 96254464 | A | G | 9.44E-04 | 1.45E-03 | 5.10E-01 | -1.58E-02 | 1.48E-03 | 1.60E-26 |
| rs34260097 | 6 | 100727703 | T | G | -2.36E-03 | 1.64E-03 | 1.50E-01 | -1.78E-02 | 1.68E-03 | 2.50E-26 |
| rs12798028 | 11 | 47604639 | C | T | -1.50E-02 | 1.39E-03 | 3.40E-27 | -1.46E-02 | 1.42E-03 | 1.30E-24 |
| rs12110721 | 6 | 55190480 | G | A | -7.88E-03 | 1.85E-03 | 2.10E-05 | -1.89E-02 | 1.89E-03 | 1.50E-23 |
| rs3810291 | 19 | 47569003 | G | A | -1.58E-02 | 1.46E-03 | 4.10E-27 | -1.47E-02 | 1.50E-03 | 6.40E-23 |
| rs12429545 | 13 | 54102206 | G | A | -2.00E-02 | 2.06E-03 | 2.60E-22 | -2.06E-02 | 2.11E-03 | 1.20E-22 |
| rs1933437 | 13 | 28624294 | G | A | 7.25E-03 | 1.42E-03 | 3.10E-07 | 1.42E-02 | 1.45E-03 | 1.20E-22 |
| rs9265968 | 6 | 31315663 | A | T | -7.51E-03 | 2.06E-03 | 2.80E-04 | -2.01E-02 | 2.11E-03 | 1.40E-21 |
| rs7239114 | 18 | 45921214 | G | A | -6.44E-03 | 1.39E-03 | 3.40E-06 | -1.35E-02 | 1.42E-03 | 1.70E-21 |
| rs7719067 | 5 | 153538241 | A | G | 9.31E-03 | 1.38E-03 | 1.70E-11 | 1.35E-02 | 1.41E-03 | 1.80E-21 |
| rs1775255 | 6 | 51243035 | G | T | -9.46E-03 | 1.38E-03 | 6.10E-12 | -1.33E-02 | 1.41E-03 | 2.10E-21 |
| rs75001243 | 7 | 93216002 | C | T | -6.80E-03 | 1.43E-03 | 1.90E-06 | -1.37E-02 | 1.46E-03 | 5.10E-21 |
| rs9260164 | 6 | 29911684 | C | T | -9.86E-03 | 1.58E-03 | 4.40E-10 | -1.52E-02 | 1.61E-03 | 5.70E-21 |
| rs7498665 | 16 | 28883241 | A | G | -1.73E-02 | 1.40E-03 | 5.90E-35 | -1.34E-02 | 1.43E-03 | 6.00E-21 |
| rs61978655 | 14 | 30491807 | G | A | -1.77E-02 | 3.57E-03 | 7.30E-07 | -3.39E-02 | 3.65E-03 | 1.40E-20 |
| rs57636386 | 18 | 58048295 | T | C | 2.47E-02 | 2.48E-03 | 2.70E-23 | 2.35E-02 | 2.54E-03 | 2.00E-20 |
| rs16996644 | 20 | 15813475 | C | G | -1.06E-02 | 2.07E-03 | 2.90E-07 | -1.93E-02 | 2.11E-03 | 4.80E-20 |
| rs7084503 | 10 | 2666859 | T | C | 4.87E-03 | 1.38E-03 | 4.10E-04 | 1.29E-02 | 1.41E-03 | 5.20E-20 |
| rs4688359 | 3 | 61198880 | C | T | 1.05E-02 | 1.42E-03 | 1.50E-13 | 1.30E-02 | 1.45E-03 | 2.10E-19 |
| rs9291816 | 5 | 63932508 | C | T | 7.98E-03 | 1.47E-03 | 5.30E-08 | 1.34E-02 | 1.50E-03 | 4.20E-19 |
| rs12140153 | 1 | 62579891 | G | T | 2.14E-02 | 2.40E-03 | 4.20E-19 | 2.18E-02 | 2.45E-03 | 6.00E-19 |
| rs7355953 | 3 | 85792137 | T | C | -1.06E-02 | 1.67E-03 | 2.00E-10 | -1.52E-02 | 1.71E-03 | 7.40E-19 |
| rs7958241 | 12 | 49509262 | A | G | -4.99E-03 | 1.45E-03 | 5.50E-04 | -1.31E-02 | 1.48E-03 | 8.60E-19 |
| rs35162296 | 6 | 26318262 | C | T | -1.14E-02 | 2.22E-03 | 2.80E-07 | -2.01E-02 | 2.27E-03 | 1.00E-18 |
| rs2767486 | 1 | 65991203 | A | G | -1.36E-03 | 1.70E-03 | 4.20E-01 | -1.54E-02 | 1.74E-03 | 1.20E-18 |
| rs41310284 | 10 | 102447647 | C | A | 1.77E-02 | 2.29E-03 | 9.80E-15 | 2.06E-02 | 2.34E-03 | 1.40E-18 |
| rs1384660 | 2 | 142299735 | G | A | 7.46E-03 | 1.76E-03 | 2.30E-05 | 1.58E-02 | 1.80E-03 | 1.80E-18 |
| rs3131336 | 6 | 28831611 | C | T | -8.18E-03 | 2.13E-03 | 1.20E-04 | -1.91E-02 | 2.18E-03 | 2.00E-18 |
| rs34196306 | 6 | 27425644 | G | C | -9.19E-03 | 2.24E-03 | 4.00E-05 | -2.00E-02 | 2.29E-03 | 2.30E-18 |
| rs3129942 | 6 | 32338283 | G | T | -8.01E-03 | 1.56E-03 | 3.10E-07 | -1.39E-02 | 1.60E-03 | 2.70E-18 |
| rs9603697 | 13 | 40783323 | C | T | -8.53E-03 | 1.46E-03 | 5.70E-09 | -1.30E-02 | 1.50E-03 | 4.60E-18 |
| rs2234458 | 11 | 65639374 | C | T | 1.28E-02 | 1.43E-03 | 2.50E-19 | 1.26E-02 | 1.46E-03 | 6.00E-18 |
| rs200744777 | 20 | 6609610 | T | G | -9.38E-03 | 1.39E-03 | 1.70E-11 | -1.21E-02 | 1.42E-03 | 1.50E-17 |
| rs13254613 | 8 | 64804804 | A | C | 5.65E-03 | 1.44E-03 | 9.10E-05 | -1.26E-02 | 1.48E-03 | 1.60E-17 |
| rs13047416 | 21 | 40309436 | C | G | 8.66E-03 | 1.42E-03 | 1.10E-09 | 1.23E-02 | 1.45E-03 | 1.80E-17 |
| rs796915 | 6 | 154304628 | C | G | -7.37E-03 | 1.49E-03 | 7.70E-07 | -1.29E-02 | 1.52E-03 | 2.30E-17 |
| rs2594994 | 3 | 11339960 | T | A | 2.11E-03 | 1.79E-03 | 2.40E-01 | 1.54E-02 | 1.83E-03 | 3.10E-17 |
| rs55726687 | 12 | 991306 | G | A | -1.38E-02 | 1.68E-03 | 3.00E-16 | -1.45E-02 | 1.72E-03 | 3.20E-17 |
| rs1333010 | 13 | 66205228 | G | A | 6.53E-03 | 1.41E-03 | 3.50E-06 | 1.21E-02 | 1.44E-03 | 4.80E-17 |
| rs4432271 | 16 | 20245283 | C | T | -1.16E-02 | 2.06E-03 | 2.10E-08 | -1.75E-02 | 2.11E-03 | 9.00E-17 |
| rs78444298 | 1 | 184672098 | G | A | -1.67E-02 | 4.99E-03 | 8.20E-04 | 4.20E-02 | 5.10E-03 | 1.60E-16 |
| rs117903946 | 16 | 67449639 | G | A | -9.78E-03 | 3.87E-03 | 1.10E-02 | -3.23E-02 | 3.95E-03 | 3.20E-16 |
| rs9317002 | 13 | 59175727 | C | A | -1.14E-02 | 1.38E-03 | 1.90E-16 | -1.15E-02 | 1.41E-03 | 3.70E-16 |
| rs1576655 | 13 | 79587841 | A | C | -1.13E-02 | 1.43E-03 | 1.90E-15 | -1.18E-02 | 1.46E-03 | 4.40E-16 |
| rs59714050 | 3 | 141267294 | T | A | -1.94E-02 | 2.75E-03 | 2.00E-12 | -2.28E-02 | 2.81E-03 | 4.90E-16 |
| rs11215403 | 11 | 115058585 | G | A | 5.91E-03 | 1.60E-03 | 2.20E-04 | 1.32E-02 | 1.63E-03 | 5.50E-16 |
| rs7656673 | 4 | 30840331 | A | G | -8.28E-03 | 1.40E-03 | 3.20E-09 | -1.16E-02 | 1.43E-03 | 6.20E-16 |
| rs13107325 | 4 | 103188709 | C | T | -2.87E-02 | 2.61E-03 | 3.10E-28 | -2.15E-02 | 2.66E-03 | 6.70E-16 |
| rs11150745 | 17 | 78757626 | A | G | 1.38E-02 | 1.48E-03 | 9.20E-21 | 1.22E-02 | 1.51E-03 | 7.50E-16 |
| rs2207894 | 20 | 54387343 | C | T | 7.60E-03 | 1.75E-03 | 1.30E-05 | 1.44E-02 | 1.78E-03 | 7.70E-16 |
| rs11642090 | 16 | 81730582 | T | C | -7.28E-03 | 1.43E-03 | 3.40E-07 | -1.17E-02 | 1.46E-03 | 1.00E-15 |
| rs788858 | 4 | 82138300 | A | G | 5.76E-04 | 1.51E-03 | 7.00E-01 | 1.23E-02 | 1.54E-03 | 1.70E-15 |
| rs12484438 | 22 | 40558064 | T | C | 1.29E-02 | 1.45E-03 | 6.70E-19 | 1.18E-02 | 1.48E-03 | 1.80E-15 |
| rs72755233 | 15 | 100692953 | G | A | -9.68E-03 | 2.18E-03 | 8.80E-06 | -1.76E-02 | 2.22E-03 | 2.30E-15 |
| rs818898 | 9 | 6970806 | A | G | 6.19E-03 | 1.49E-03 | 3.30E-05 | 1.21E-02 | 1.52E-03 | 2.30E-15 |
| rs12713889 | 2 | 77225361 | T | C | 5.70E-03 | 1.45E-03 | 8.70E-05 | 1.17E-02 | 1.49E-03 | 3.80E-15 |
| rs12450028 | 17 | 2207425 | C | T | 6.86E-03 | 1.44E-03 | 2.00E-06 | 1.16E-02 | 1.47E-03 | 4.20E-15 |
| rs2722406 | 7 | 24306762 | C | T | -4.31E-03 | 1.52E-03 | 4.60E-03 | -1.22E-02 | 1.55E-03 | 4.40E-15 |
| rs2735556 | 3 | 88105360 | T | C | 1.29E-02 | 2.15E-03 | 1.70E-09 | 1.72E-02 | 2.20E-03 | 5.10E-15 |
| rs10796828 | 11 | 69490346 | T | G | -7.85E-03 | 1.43E-03 | 3.70E-08 | -1.13E-02 | 1.46E-03 | 6.90E-15 |
| rs2187642 | 12 | 11855624 | A | C | -1.58E-03 | 1.41E-03 | 2.60E-01 | -1.12E-02 | 1.44E-03 | 1.00E-14 |
| rs12606230 | 18 | 52492252 | T | C | -1.17E-02 | 1.62E-03 | 6.20E-13 | -1.27E-02 | 1.66E-03 | 1.70E-14 |
| rs6449532 | 5 | 60715446 | C | T | 6.98E-03 | 1.43E-03 | 9.90E-07 | 1.11E-02 | 1.46E-03 | 3.30E-14 |
| rs10896348 | 11 | 68357368 | T | C | 7.20E-03 | 1.53E-03 | 2.60E-06 | 1.19E-02 | 1.56E-03 | 3.30E-14 |
| rs1013737 | 18 | 937050 | G | C | -5.85E-03 | 1.37E-03 | 2.10E-05 | -1.06E-02 | 1.40E-03 | 3.40E-14 |
| rs17399739 | 10 | 87490850 | A | G | -1.78E-02 | 2.71E-03 | 5.90E-11 | -2.10E-02 | 2.77E-03 | 3.90E-14 |
| rs1199333 | 3 | 138091701 | G | T | 9.32E-03 | 1.76E-03 | 1.20E-07 | 1.35E-02 | 1.80E-03 | 5.90E-14 |
| rs2034963 | 3 | 48170802 | G | C | 5.00E-03 | 1.45E-03 | 5.40E-04 | 1.11E-02 | 1.48E-03 | 6.70E-14 |
| rs39862 | 5 | 66185151 | T | C | 9.63E-03 | 1.52E-03 | 2.60E-10 | 1.16E-02 | 1.56E-03 | 7.50E-14 |
| rs1342831 | 6 | 54096151 | T | C | -6.59E-03 | 2.95E-03 | 2.60E-02 | -2.25E-02 | 3.02E-03 | 9.00E-14 |
| rs7989098 | 13 | 27925496 | T | C | -7.42E-03 | 1.59E-03 | 3.10E-06 | -1.21E-02 | 1.63E-03 | 1.00E-13 |
| rs11525873 | 7 | 138817193 | T | C | 1.48E-02 | 2.31E-03 | 1.40E-10 | 1.75E-02 | 2.36E-03 | 1.30E-13 |
| rs117911387 | 9 | 130446836 | G | A | -3.68E-03 | 3.26E-03 | 2.60E-01 | -2.46E-02 | 3.33E-03 | 1.60E-13 |
| rs2229330 | 1 | 6649228 | T | G | -3.47E-03 | 2.64E-03 | 1.90E-01 | -1.97E-02 | 2.69E-03 | 2.30E-13 |
| rs9438393 | 1 | 205782718 | A | G | 5.27E-03 | 1.39E-03 | 1.50E-04 | 1.03E-02 | 1.42E-03 | 3.40E-13 |
| rs200801362 | 6 | 31555480 | T | C | -6.08E-03 | 2.54E-03 | 1.70E-02 | -1.88E-02 | 2.60E-03 | 3.80E-13 |
| rs2275241 | 9 | 129370576 | G | A | -4.11E-03 | 1.42E-03 | 3.70E-03 | -1.04E-02 | 1.45E-03 | 7.50E-13 |
| rs12214497 | 6 | 10015908 | G | T | 5.63E-03 | 1.44E-03 | 9.30E-05 | 1.05E-02 | 1.47E-03 | 9.40E-13 |
| rs12883788 | 14 | 33303540 | C | T | -1.24E-02 | 1.38E-03 | 2.30E-19 | -1.00E-02 | 1.41E-03 | 1.10E-12 |
| rs7306710 | 12 | 66376091 | T | C | 7.35E-04 | 1.38E-03 | 5.90E-01 | 9.97E-03 | 1.41E-03 | 1.60E-12 |
| rs3748126 | 7 | 76632736 | C | G | 1.65E-02 | 1.82E-03 | 1.30E-19 | 1.31E-02 | 1.86E-03 | 1.70E-12 |
| rs78607331 | 12 | 57648644 | C | T | -1.80E-02 | 3.31E-03 | 6.10E-08 | -2.39E-02 | 3.38E-03 | 1.70E-12 |
| rs77960 | 5 | 103964585 | G | A | -6.24E-03 | 1.46E-03 | 1.90E-05 | 1.05E-02 | 1.49E-03 | 1.70E-12 |
| rs630602 | 1 | 54728864 | G | C | -7.73E-03 | 1.41E-03 | 4.10E-08 | -1.01E-02 | 1.44E-03 | 1.80E-12 |
| rs7606059 | 2 | 188152749 | T | C | -4.05E-03 | 1.46E-03 | 5.70E-03 | -1.05E-02 | 1.50E-03 | 2.10E-12 |
| rs7305424 | 12 | 118399491 | A | T | -3.88E-03 | 1.45E-03 | 7.60E-03 | -1.04E-02 | 1.48E-03 | 2.40E-12 |
| rs7869098 | 9 | 27816218 | T | G | 6.36E-03 | 1.38E-03 | 3.80E-06 | 9.84E-03 | 1.41E-03 | 2.60E-12 |
| rs61937656 | 12 | 39483502 | G | A | 5.07E-03 | 1.64E-03 | 2.00E-03 | 1.17E-02 | 1.68E-03 | 2.80E-12 |
| rs73422097 | 6 | 41727740 | A | G | -5.87E-03 | 1.49E-03 | 8.30E-05 | -1.06E-02 | 1.52E-03 | 3.30E-12 |
| rs957512 | 9 | 120405705 | T | C | 8.80E-03 | 1.46E-03 | 1.80E-09 | 1.03E-02 | 1.49E-03 | 4.50E-12 |
| rs7931626 | 11 | 28421841 | C | T | 5.27E-03 | 1.38E-03 | 1.30E-04 | 9.70E-03 | 1.41E-03 | 5.00E-12 |
| rs1452991 | 6 | 141473363 | G | A | -5.77E-03 | 1.43E-03 | 5.30E-05 | -1.01E-02 | 1.46E-03 | 5.30E-12 |
| rs111768603 | 3 | 42329113 | G | T | 1.59E-02 | 2.19E-03 | 3.80E-13 | 1.54E-02 | 2.24E-03 | 5.60E-12 |
| rs7619139 | 3 | 25110415 | T | A | -8.84E-03 | 1.40E-03 | 2.40E-10 | -9.80E-03 | 1.43E-03 | 6.50E-12 |
| rs10790809 | 11 | 126372550 | A | G | -6.57E-03 | 1.38E-03 | 2.00E-06 | -9.67E-03 | 1.41E-03 | 7.00E-12 |
| rs7012648 | 8 | 28091482 | G | A | -6.85E-03 | 1.40E-03 | 9.30E-07 | -9.74E-03 | 1.43E-03 | 8.40E-12 |
| rs947088 | 20 | 17171373 | G | T | -7.27E-03 | 1.53E-03 | 2.00E-06 | -1.06E-02 | 1.56E-03 | 1.00E-11 |
| rs2968973 | 4 | 130740404 | C | T | 8.11E-03 | 1.43E-03 | 1.40E-08 | 9.93E-03 | 1.46E-03 | 1.10E-11 |
| rs3817428 | 15 | 89415247 | C | G | -3.19E-03 | 1.56E-03 | 4.10E-02 | -1.08E-02 | 1.59E-03 | 1.30E-11 |
| rs836179 | 12 | 50503082 | A | G | 2.37E-03 | 1.42E-03 | 9.50E-02 | 9.81E-03 | 1.45E-03 | 1.40E-11 |
| rs62425398 | 6 | 166416028 | C | A | -9.12E-03 | 2.24E-03 | 4.50E-05 | -1.54E-02 | 2.28E-03 | 1.50E-11 |
| rs11205303 | 1 | 149906413 | T | C | 1.24E-03 | 1.39E-03 | 3.80E-01 | 9.58E-03 | 1.43E-03 | 1.80E-11 |
| rs139497 | 22 | 41640098 | C | T | 6.20E-03 | 1.49E-03 | 3.30E-05 | 1.02E-02 | 1.52E-03 | 2.00E-11 |
| rs601338 | 19 | 49206674 | G | A | 9.17E-04 | 1.37E-03 | 5.00E-01 | 9.38E-03 | 1.40E-03 | 2.10E-11 |
| rs6979832 | 7 | 127856276 | A | G | -3.30E-03 | 1.38E-03 | 1.70E-02 | -9.42E-03 | 1.41E-03 | 2.30E-11 |
| rs661878 | 11 | 29188691 | A | G | 8.60E-03 | 2.02E-03 | 2.00E-05 | 1.38E-02 | 2.06E-03 | 2.50E-11 |
| rs112898427 | 2 | 67561335 | C | T | 1.04E-03 | 1.52E-03 | 4.90E-01 | 1.03E-02 | 1.55E-03 | 2.50E-11 |
| rs10423928 | 19 | 46182304 | T | A | 2.08E-02 | 1.73E-03 | 4.20E-33 | 1.18E-02 | 1.77E-03 | 2.60E-11 |
| rs1320903 | 3 | 131758077 | G | A | -1.46E-02 | 1.47E-03 | 2.60E-23 | -1.00E-02 | 1.50E-03 | 2.70E-11 |
| rs7808296 | 7 | 103127620 | C | T | -2.55E-03 | 1.48E-03 | 8.50E-02 | -1.00E-02 | 1.51E-03 | 2.80E-11 |
| rs146910503 | 2 | 25446473 | G | A | 1.48E-02 | 4.91E-03 | 2.60E-03 | 3.34E-02 | 5.03E-03 | 2.90E-11 |
| rs7759938 | 6 | 105378954 | C | T | -3.05E-03 | 1.47E-03 | 3.80E-02 | -9.98E-03 | 1.50E-03 | 2.90E-11 |
| rs824207 | 15 | 24007729 | A | G | -2.21E-03 | 1.38E-03 | 1.10E-01 | -9.31E-03 | 1.40E-03 | 3.30E-11 |
| rs1422067 | 5 | 77424836 | C | T | 8.70E-03 | 1.61E-03 | 6.50E-08 | 1.09E-02 | 1.65E-03 | 3.80E-11 |
| rs7162542 | 15 | 84514290 | C | G | 3.15E-03 | 1.38E-03 | 2.30E-02 | 9.31E-03 | 1.41E-03 | 4.00E-11 |
| rs2970356 | 15 | 90623540 | C | G | -3.62E-03 | 1.55E-03 | 2.00E-02 | -1.05E-02 | 1.58E-03 | 4.10E-11 |
| rs4074404 | 1 | 187683956 | T | A | -4.84E-03 | 1.88E-03 | 1.00E-02 | -1.27E-02 | 1.92E-03 | 4.30E-11 |
| rs12045879 | 1 | 15817090 | C | T | 6.18E-03 | 1.47E-03 | 2.70E-05 | 9.89E-03 | 1.51E-03 | 5.30E-11 |
| rs6265 | 11 | 27679916 | C | T | 2.45E-02 | 1.75E-03 | 1.50E-44 | 1.17E-02 | 1.79E-03 | 6.30E-11 |
| rs55896564 | 8 | 11447093 | G | A | 1.17E-02 | 1.38E-03 | 2.60E-17 | 9.11E-03 | 1.41E-03 | 1.10E-10 |
| rs7020564 | 9 | 109670016 | A | T | 4.97E-03 | 1.52E-03 | 1.10E-03 | 1.00E-02 | 1.55E-03 | 1.10E-10 |
| rs10095724 | 8 | 53739232 | G | A | 2.96E-03 | 1.43E-03 | 3.90E-02 | 9.37E-03 | 1.46E-03 | 1.40E-10 |
| rs7536458 | 1 | 118864602 | T | G | -7.40E-03 | 1.55E-03 | 1.90E-06 | -1.01E-02 | 1.59E-03 | 1.90E-10 |
| rs34517439 | 1 | 78450517 | C | A | -2.47E-02 | 2.12E-03 | 1.90E-31 | -1.38E-02 | 2.16E-03 | 2.00E-10 |
| rs8096658 | 18 | 77156537 | C | G | 4.29E-03 | 1.39E-03 | 2.00E-03 | 9.02E-03 | 1.42E-03 | 2.00E-10 |
| rs4958568 | 5 | 152016093 | G | A | 4.81E-03 | 1.53E-03 | 1.70E-03 | 9.96E-03 | 1.57E-03 | 2.10E-10 |
| rs7424771 | 2 | 161276378 | G | A | -2.31E-03 | 1.38E-03 | 9.40E-02 | 8.93E-03 | 1.41E-03 | 2.20E-10 |
| rs4572029 | 10 | 70889053 | A | G | -7.22E-04 | 1.71E-03 | 6.70E-01 | 1.11E-02 | 1.75E-03 | 2.20E-10 |
| rs2246623 | 17 | 74084449 | C | T | 4.79E-03 | 1.38E-03 | 5.20E-04 | 8.94E-03 | 1.41E-03 | 2.30E-10 |
| rs2141004 | 2 | 6194359 | A | C | 7.39E-03 | 1.55E-03 | 2.00E-06 | 1.00E-02 | 1.59E-03 | 2.50E-10 |
| rs559231 | 18 | 39644247 | G | T | -9.52E-03 | 1.41E-03 | 1.50E-11 | -9.10E-03 | 1.44E-03 | 2.70E-10 |
| rs10133279 | 14 | 82702712 | C | T | -4.83E-03 | 1.39E-03 | 5.20E-04 | -8.96E-03 | 1.42E-03 | 2.80E-10 |
| rs9922288 | 16 | 24550930 | A | G | 9.39E-03 | 1.64E-03 | 9.80E-09 | 1.05E-02 | 1.67E-03 | 3.00E-10 |
| rs1788808 | 18 | 21090023 | A | G | 1.27E-02 | 1.37E-03 | 1.90E-20 | 8.81E-03 | 1.40E-03 | 3.40E-10 |
| rs77976727 | 8 | 4300554 | C | T | -7.44E-03 | 2.36E-03 | 1.60E-03 | -1.51E-02 | 2.41E-03 | 3.60E-10 |
| rs2629881 | 3 | 59778271 | C | T | -2.64E-03 | 1.65E-03 | 1.10E-01 | -1.06E-02 | 1.69E-03 | 3.80E-10 |
| rs78907487 | 22 | 22151939 | A | C | -6.77E-03 | 1.94E-03 | 4.70E-04 | -1.24E-02 | 1.98E-03 | 3.90E-10 |
| rs10503246 | 8 | 4130363 | A | G | -3.08E-03 | 1.52E-03 | 4.20E-02 | -9.66E-03 | 1.55E-03 | 4.60E-10 |
| rs7753558 | 6 | 117523471 | C | A | 5.70E-03 | 1.43E-03 | 6.80E-05 | 9.09E-03 | 1.46E-03 | 5.20E-10 |
| rs7951870 | 11 | 46373311 | T | C | -7.15E-03 | 1.82E-03 | 8.70E-05 | -1.16E-02 | 1.86E-03 | 5.40E-10 |
| rs34811474 | 4 | 25408838 | G | A | 1.66E-02 | 1.63E-03 | 2.00E-24 | 1.02E-02 | 1.66E-03 | 8.20E-10 |
| rs62621197 | 19 | 8670147 | C | T | -6.50E-03 | 3.77E-03 | 8.50E-02 | -2.35E-02 | 3.85E-03 | 9.60E-10 |
| rs212517 | 1 | 21577159 | T | A | 1.56E-03 | 1.40E-03 | 2.60E-01 | 8.75E-03 | 1.43E-03 | 9.60E-10 |
| rs62134189 | 2 | 45046339 | A | G | -1.82E-03 | 2.27E-03 | 4.20E-01 | 1.42E-02 | 2.32E-03 | 1.00E-09 |
| rs2307111 | 5 | 75003678 | T | C | 1.68E-02 | 1.40E-03 | 6.90E-33 | 8.74E-03 | 1.43E-03 | 1.10E-09 |
| rs117455294 | 20 | 57427951 | C | A | 4.90E-03 | 3.11E-03 | 1.10E-01 | 1.93E-02 | 3.17E-03 | 1.10E-09 |
| rs10234366 | 7 | 46743746 | G | A | -1.75E-03 | 2.24E-03 | 4.40E-01 | -1.40E-02 | 2.29E-03 | 1.10E-09 |
| rs3936511 | 5 | 55860781 | A | G | 2.42E-04 | 1.74E-03 | 8.90E-01 | 1.08E-02 | 1.78E-03 | 1.10E-09 |
| rs7827182 | 8 | 8380471 | G | C | -1.12E-02 | 1.37E-03 | 2.90E-16 | -8.49E-03 | 1.40E-03 | 1.40E-09 |
| rs2281148 | 20 | 36433288 | T | C | -5.55E-03 | 1.59E-03 | 4.70E-04 | -9.79E-03 | 1.62E-03 | 1.50E-09 |
| rs75387636 | 10 | 120278394 | G | A | -7.46E-03 | 3.44E-03 | 3.00E-02 | -2.12E-02 | 3.51E-03 | 1.50E-09 |
| rs10860295 | 12 | 98542699 | T | C | -1.81E-03 | 1.38E-03 | 1.90E-01 | -8.52E-03 | 1.41E-03 | 1.60E-09 |
| rs1852006 | 7 | 77829768 | G | A | 8.83E-03 | 1.43E-03 | 6.70E-10 | 8.77E-03 | 1.46E-03 | 1.90E-09 |
| rs11496125 | 7 | 103417557 | C | T | -1.09E-02 | 1.39E-03 | 4.80E-15 | -8.54E-03 | 1.42E-03 | 2.00E-09 |
| rs10498713 | 6 | 22729300 | G | T | -1.36E-03 | 1.92E-03 | 4.80E-01 | -1.18E-02 | 1.97E-03 | 2.00E-09 |
| rs2999158 | 1 | 113239478 | T | C | -3.26E-03 | 1.45E-03 | 2.50E-02 | 8.87E-03 | 1.48E-03 | 2.20E-09 |
| rs7814267 | 8 | 5545084 | A | G | -5.53E-03 | 1.79E-03 | 2.00E-03 | -1.09E-02 | 1.83E-03 | 2.50E-09 |
| rs8117463 | 20 | 17231063 | G | A | 5.19E-03 | 1.47E-03 | 4.10E-04 | 8.90E-03 | 1.50E-03 | 2.90E-09 |
| rs146980124 | 17 | 44627649 | A | C | 5.48E-03 | 1.69E-03 | 1.20E-03 | 1.02E-02 | 1.72E-03 | 2.90E-09 |
| rs10953577 | 7 | 108263540 | T | C | -2.48E-03 | 1.42E-03 | 8.00E-02 | -8.58E-03 | 1.45E-03 | 3.00E-09 |
| rs12748436 | 1 | 177761109 | C | G | -5.65E-03 | 2.58E-03 | 2.90E-02 | -1.56E-02 | 2.64E-03 | 3.50E-09 |
| rs4723263 | 7 | 33194826 | G | C | -2.54E-03 | 1.38E-03 | 6.60E-02 | -8.33E-03 | 1.41E-03 | 3.70E-09 |
| rs7123283 | 11 | 122809055 | C | T | 2.53E-03 | 1.38E-03 | 6.70E-02 | 8.32E-03 | 1.41E-03 | 3.70E-09 |
| rs67603370 | 17 | 7524504 | G | A | -4.59E-04 | 2.63E-03 | 8.60E-01 | -1.58E-02 | 2.68E-03 | 3.70E-09 |
| rs11891707 | 2 | 207120604 | T | C | 4.36E-04 | 2.01E-03 | 8.30E-01 | 1.21E-02 | 2.05E-03 | 3.80E-09 |
| rs9652090 | 13 | 27983367 | G | T | -3.71E-03 | 1.39E-03 | 7.40E-03 | -8.33E-03 | 1.42E-03 | 4.00E-09 |
| rs10842356 | 12 | 24621348 | A | T | 5.19E-03 | 1.37E-03 | 1.60E-04 | 8.23E-03 | 1.40E-03 | 4.10E-09 |
| rs62032001 | 16 | 49065630 | A | C | -1.94E-03 | 1.73E-03 | 2.60E-01 | -1.04E-02 | 1.77E-03 | 4.20E-09 |
| rs10116891 | 9 | 122651993 | G | A | -9.94E-03 | 2.29E-03 | 1.40E-05 | -1.36E-02 | 2.34E-03 | 5.80E-09 |
| rs884152 | 8 | 25770557 | G | T | -3.32E-03 | 1.43E-03 | 2.00E-02 | -8.51E-03 | 1.46E-03 | 6.00E-09 |
| rs3118252 | 9 | 25115154 | G | C | -1.47E-03 | 1.40E-03 | 2.90E-01 | -8.32E-03 | 1.43E-03 | 6.00E-09 |
| rs61936936 | 12 | 116391685 | A | T | -4.86E-03 | 2.30E-03 | 3.40E-02 | -1.36E-02 | 2.34E-03 | 6.20E-09 |
| rs9594686 | 13 | 42723197 | C | T | 6.04E-03 | 1.80E-03 | 8.10E-04 | 1.07E-02 | 1.84E-03 | 6.40E-09 |
| rs3181269 | 11 | 33755956 | C | T | 3.49E-03 | 1.57E-03 | 2.60E-02 | 9.33E-03 | 1.61E-03 | 6.40E-09 |
| rs9370527 | 6 | 56245812 | G | A | -3.28E-03 | 1.60E-03 | 4.10E-02 | -9.49E-03 | 1.64E-03 | 6.40E-09 |
| rs686431 | 6 | 35974217 | C | T | -1.48E-02 | 5.09E-03 | 3.70E-03 | -3.02E-02 | 5.20E-03 | 6.50E-09 |
| rs4739558 | 8 | 38337264 | A | G | 8.10E-03 | 1.40E-03 | 7.40E-09 | 8.30E-03 | 1.43E-03 | 6.70E-09 |
| rs74080008 | 1 | 51047717 | G | T | 5.59E-03 | 1.52E-03 | 2.40E-04 | 9.01E-03 | 1.56E-03 | 6.80E-09 |
| rs7354849 | 1 | 232765308 | A | G | -4.26E-03 | 1.38E-03 | 2.00E-03 | -8.17E-03 | 1.41E-03 | 6.80E-09 |
| rs2712667 | 12 | 99588917 | G | C | 9.62E-03 | 1.44E-03 | 2.10E-11 | 8.49E-03 | 1.47E-03 | 7.30E-09 |
| rs7672 | 16 | 68294800 | C | G | 4.69E-03 | 1.53E-03 | 2.20E-03 | 9.00E-03 | 1.56E-03 | 7.90E-09 |
| rs7503580 | 17 | 79087036 | C | T | -4.88E-03 | 1.89E-03 | 1.00E-02 | -1.11E-02 | 1.93E-03 | 8.90E-09 |
| rs115359679 | 7 | 755987 | C | A | -5.38E-03 | 2.80E-03 | 5.40E-02 | -1.64E-02 | 2.86E-03 | 9.20E-09 |
| rs7711823 | 5 | 158489315 | A | G | 6.57E-03 | 1.43E-03 | 4.30E-06 | 8.39E-03 | 1.46E-03 | 9.30E-09 |
| rs7565437 | 2 | 65646966 | T | C | 2.01E-03 | 1.39E-03 | 1.50E-01 | 8.18E-03 | 1.43E-03 | 9.50E-09 |
| rs11256627 | 10 | 10535954 | G | A | -9.45E-04 | 1.51E-03 | 5.30E-01 | -8.88E-03 | 1.55E-03 | 9.50E-09 |
| rs12941038 | 17 | 66509143 | C | T | -4.75E-03 | 1.63E-03 | 3.70E-03 | -9.54E-03 | 1.67E-03 | 1.10E-08 |
| rs4545941 | 19 | 16534207 | T | C | -2.12E-03 | 1.86E-03 | 2.60E-01 | -1.09E-02 | 1.90E-03 | 1.10E-08 |
| rs4783789 | 16 | 51446707 | T | C | 1.65E-03 | 1.65E-03 | 3.20E-01 | 9.58E-03 | 1.68E-03 | 1.20E-08 |
| rs12308065 | 12 | 120624085 | A | G | -6.54E-04 | 1.42E-03 | 6.50E-01 | -8.29E-03 | 1.45E-03 | 1.20E-08 |
| rs11134679 | 5 | 170623391 | A | G | -1.24E-02 | 1.48E-03 | 5.20E-17 | -8.60E-03 | 1.51E-03 | 1.30E-08 |
| rs201666051 | 9 | 20920868 | C | T | -1.49E-03 | 1.42E-03 | 2.90E-01 | -8.24E-03 | 1.45E-03 | 1.30E-08 |
| rs1296328 | 4 | 137083193 | A | C | 1.17E-02 | 1.39E-03 | 2.60E-17 | 8.04E-03 | 1.42E-03 | 1.40E-08 |
| rs6870983 | 5 | 87697533 | C | T | 1.38E-02 | 1.67E-03 | 1.60E-16 | 9.70E-03 | 1.71E-03 | 1.40E-08 |
| rs1476698 | 2 | 242296449 | A | G | 1.39E-03 | 1.42E-03 | 3.30E-01 | 8.22E-03 | 1.45E-03 | 1.40E-08 |
| rs7439324 | 4 | 44501351 | C | T | 7.53E-03 | 1.86E-03 | 5.30E-05 | 1.08E-02 | 1.90E-03 | 1.50E-08 |
| rs2755253 | 1 | 67470843 | C | T | 1.53E-03 | 1.51E-03 | 3.10E-01 | 8.71E-03 | 1.54E-03 | 1.50E-08 |
| rs1177279 | 2 | 61295122 | A | G | 2.06E-03 | 1.53E-03 | 1.80E-01 | 8.83E-03 | 1.56E-03 | 1.60E-08 |
| rs60644673 | 7 | 100096742 | G | T | -4.06E-03 | 1.74E-03 | 2.00E-02 | 1.00E-02 | 1.78E-03 | 1.70E-08 |
| rs34722008 | 4 | 38659594 | G | A | 6.99E-03 | 1.44E-03 | 1.10E-06 | 8.27E-03 | 1.47E-03 | 1.80E-08 |
| rs1619120 | 9 | 87302196 | A | G | -6.79E-03 | 1.40E-03 | 1.30E-06 | -8.06E-03 | 1.43E-03 | 1.80E-08 |
| rs1696057 | 12 | 90767160 | T | C | 3.91E-03 | 1.45E-03 | 6.80E-03 | 8.31E-03 | 1.48E-03 | 1.80E-08 |
| rs6577497 | 1 | 8605667 | A | T | -2.20E-03 | 1.40E-03 | 1.20E-01 | 8.07E-03 | 1.43E-03 | 1.80E-08 |
| rs2958542 | 11 | 62181882 | C | T | 3.51E-04 | 1.43E-03 | 8.10E-01 | 8.24E-03 | 1.46E-03 | 1.80E-08 |
| rs10887571 | 10 | 88030441 | C | T | -6.82E-03 | 1.39E-03 | 9.10E-07 | -7.98E-03 | 1.42E-03 | 1.90E-08 |
| rs115903965 | 3 | 66009529 | G | A | 1.86E-03 | 4.44E-03 | 6.80E-01 | -2.55E-02 | 4.54E-03 | 2.00E-08 |
| rs2939931 | 10 | 121636406 | T | C | -2.74E-03 | 1.37E-03 | 4.60E-02 | -7.86E-03 | 1.40E-03 | 2.20E-08 |
| rs8130408 | 21 | 39237138 | A | C | -4.68E-03 | 1.58E-03 | 3.10E-03 | -9.01E-03 | 1.61E-03 | 2.30E-08 |
| rs3815156 | 17 | 29685150 | A | G | -5.29E-03 | 1.81E-03 | 3.40E-03 | -1.03E-02 | 1.84E-03 | 2.30E-08 |
| rs538579 | 3 | 62711674 | G | C | -7.99E-03 | 1.48E-03 | 6.30E-08 | -8.42E-03 | 1.51E-03 | 2.40E-08 |
| rs6601451 | 8 | 10243681 | C | G | 1.16E-02 | 1.37E-03 | 3.70E-17 | 7.81E-03 | 1.40E-03 | 2.60E-08 |
| rs2268762 | 3 | 38516075 | A | G | -4.39E-03 | 1.40E-03 | 1.80E-03 | -7.99E-03 | 1.44E-03 | 2.60E-08 |
| rs1402989 | 3 | 27056851 | C | T | -4.03E-03 | 1.37E-03 | 3.30E-03 | -7.79E-03 | 1.40E-03 | 2.70E-08 |
| rs7840305 | 8 | 57168101 | A | G | 3.42E-03 | 1.42E-03 | 1.60E-02 | 8.06E-03 | 1.45E-03 | 2.70E-08 |
| rs7145882 | 14 | 103255461 | T | C | 1.15E-02 | 1.45E-03 | 2.20E-15 | 8.19E-03 | 1.48E-03 | 2.90E-08 |
| rs6719507 | 2 | 29733801 | G | A | 3.02E-03 | 1.38E-03 | 2.90E-02 | 7.82E-03 | 1.41E-03 | 2.90E-08 |
| rs73085586 | 20 | 22430241 | G | A | -4.65E-03 | 1.72E-03 | 6.70E-03 | -9.71E-03 | 1.75E-03 | 3.00E-08 |
| rs11040333 | 11 | 49346332 | G | A | -3.98E-03 | 1.51E-03 | 8.50E-03 | -8.55E-03 | 1.54E-03 | 3.10E-08 |
| rs55658481 | 2 | 219284215 | G | A | -8.97E-03 | 1.44E-03 | 5.30E-10 | -8.16E-03 | 1.48E-03 | 3.30E-08 |
| rs3791478 | 2 | 240064139 | T | C | 4.64E-03 | 2.21E-03 | 3.60E-02 | 1.25E-02 | 2.26E-03 | 3.30E-08 |
| rs594585 | 16 | 65939803 | T | G | 4.87E-03 | 1.41E-03 | 5.50E-04 | 7.95E-03 | 1.44E-03 | 3.40E-08 |
| rs10823504 | 10 | 72034062 | G | A | 9.02E-04 | 2.81E-03 | 7.50E-01 | 1.59E-02 | 2.87E-03 | 3.40E-08 |
| rs16839832 | 1 | 196349909 | G | T | -2.65E-03 | 2.50E-03 | 2.90E-01 | -1.41E-02 | 2.55E-03 | 3.50E-08 |
| rs76187039 | 6 | 43233990 | G | T | -1.12E-03 | 2.01E-03 | 5.80E-01 | -1.13E-02 | 2.05E-03 | 3.50E-08 |
| rs10791902 | 11 | 67093360 | C | T | -5.63E-03 | 1.41E-03 | 6.20E-05 | -7.91E-03 | 1.44E-03 | 3.60E-08 |
| rs2175171 | 1 | 7028842 | G | C | -1.93E-03 | 1.38E-03 | 1.60E-01 | -7.77E-03 | 1.41E-03 | 3.60E-08 |
| rs1000471 | 15 | 89986583 | C | T | 9.10E-04 | 1.69E-03 | 5.90E-01 | -9.51E-03 | 1.73E-03 | 3.60E-08 |
| rs9610387 | 22 | 36476762 | G | A | 7.89E-03 | 2.46E-03 | 1.30E-03 | 1.38E-02 | 2.51E-03 | 3.70E-08 |
| rs4677156 | 3 | 72417857 | A | T | 3.78E-03 | 1.64E-03 | 2.10E-02 | 9.25E-03 | 1.68E-03 | 3.70E-08 |
| rs3172332 | 3 | 153973408 | T | C | 2.69E-03 | 1.42E-03 | 5.80E-02 | 8.01E-03 | 1.46E-03 | 3.70E-08 |
| rs2157295 | 22 | 42701984 | T | G | -4.94E-03 | 1.42E-03 | 5.00E-04 | -7.96E-03 | 1.45E-03 | 3.80E-08 |
| rs11655704 | 17 | 47448172 | T | C | 2.44E-03 | 1.47E-03 | 9.70E-02 | 8.27E-03 | 1.50E-03 | 3.80E-08 |
| rs10503555 | 8 | 15763818 | A | G | -6.46E-04 | 1.39E-03 | 6.40E-01 | 7.78E-03 | 1.41E-03 | 3.90E-08 |
| rs762705 | 3 | 50313527 | A | C | 7.21E-03 | 1.92E-03 | 1.70E-04 | 1.08E-02 | 1.96E-03 | 4.00E-08 |
| rs68015088 | 18 | 51484010 | G | A | 5.01E-03 | 1.45E-03 | 5.50E-04 | 8.10E-03 | 1.48E-03 | 4.30E-08 |
| rs10111937 | 8 | 54160092 | C | T | -4.90E-03 | 1.50E-03 | 1.10E-03 | -8.37E-03 | 1.53E-03 | 4.30E-08 |
| rs62048187 | 15 | 38117049 | G | C | -6.01E-03 | 1.50E-03 | 6.10E-05 | -8.39E-03 | 1.53E-03 | 4.40E-08 |
| rs67679818 | 7 | 110672704 | C | T | 4.06E-04 | 1.40E-03 | 7.70E-01 | 7.80E-03 | 1.43E-03 | 4.90E-08 |

**Table S2B.** The full set of genetic variants strongly associated with adulthood adiposity.

| SNP | Chromosome | Base position | Effect allele | Other allele | Beta (Adult) | SE (Adult) | P (Adult) | Beta (Age 10) | SE (Age 10) | P (Age 10) |
| --- | --- | --- | --- | --- | --- | --- | --- | --- | --- | --- |
| rs56094641 | 16 | 53806453 | A | G | -4.65E-02 | 1.40E-03 | 4.00E-243 | -4.73E-02 | 1.43E-03 | 1.60E-240 |
| rs543874 | 1 | 177889480 | A | G | -3.01E-02 | 1.69E-03 | 1.00E-70 | -4.71E-02 | 1.73E-03 | 6.00E-163 |
| rs10182458 | 2 | 25150641 | A | G | -1.99E-02 | 1.37E-03 | 4.10E-48 | -3.59E-02 | 1.40E-03 | 3.50E-145 |
| rs62106258 | 2 | 417167 | T | C | 5.91E-02 | 3.18E-03 | 4.20E-77 | 8.02E-02 | 3.25E-03 | 3.60E-134 |
| rs12992672 | 2 | 632592 | G | A | -3.60E-02 | 1.81E-03 | 1.20E-87 | -4.33E-02 | 1.85E-03 | 4.00E-121 |
| rs7132908 | 12 | 50263148 | G | A | -1.86E-02 | 1.41E-03 | 8.40E-40 | -3.13E-02 | 1.44E-03 | 1.60E-104 |
| rs58084604 | 18 | 57849429 | C | T | -3.52E-02 | 1.62E-03 | 2.60E-104 | -3.32E-02 | 1.66E-03 | 3.00E-89 |
| rs12042908 | 1 | 74997762 | A | G | 1.04E-02 | 1.38E-03 | 3.90E-14 | 2.75E-02 | 1.41E-03 | 2.50E-84 |
| rs12641981 | 4 | 45179883 | C | T | -1.87E-02 | 1.38E-03 | 1.90E-41 | -2.22E-02 | 1.42E-03 | 1.80E-55 |
| rs55880046 | 16 | 19941557 | T | G | 1.69E-02 | 1.96E-03 | 8.70E-18 | 2.99E-02 | 2.01E-03 | 3.90E-50 |
| rs72892910 | 6 | 50816887 | G | T | -2.53E-02 | 1.82E-03 | 5.70E-44 | -2.47E-02 | 1.86E-03 | 3.20E-40 |
| rs11209943 | 1 | 72750500 | A | G | -1.41E-02 | 1.40E-03 | 6.60E-24 | -1.83E-02 | 1.43E-03 | 8.80E-38 |
| rs8030456 | 15 | 68076856 | C | T | 1.83E-02 | 1.64E-03 | 3.70E-29 | 2.13E-02 | 1.67E-03 | 2.80E-37 |
| rs35918296 | 8 | 76862208 | C | T | 1.02E-02 | 1.39E-03 | 2.90E-13 | 1.67E-02 | 1.42E-03 | 1.40E-31 |
| rs2238435 | 16 | 4014282 | C | G | -1.41E-02 | 1.41E-03 | 1.20E-23 | -1.65E-02 | 1.44E-03 | 3.00E-30 |
| rs7550711 | 1 | 110082886 | C | T | -4.26E-02 | 4.32E-03 | 5.10E-23 | -4.84E-02 | 4.41E-03 | 5.20E-28 |
| rs12798028 | 11 | 47604639 | C | T | -1.50E-02 | 1.39E-03 | 3.40E-27 | -1.46E-02 | 1.42E-03 | 1.30E-24 |
| rs3810291 | 19 | 47569003 | G | A | -1.58E-02 | 1.46E-03 | 4.10E-27 | -1.47E-02 | 1.50E-03 | 6.40E-23 |
| rs12429545 | 13 | 54102206 | G | A | -2.00E-02 | 2.06E-03 | 2.60E-22 | -2.06E-02 | 2.11E-03 | 1.20E-22 |
| rs7719067 | 5 | 153538241 | A | G | 9.31E-03 | 1.38E-03 | 1.70E-11 | 1.35E-02 | 1.41E-03 | 1.80E-21 |
| rs1775255 | 6 | 51243035 | G | T | -9.46E-03 | 1.38E-03 | 6.10E-12 | -1.33E-02 | 1.41E-03 | 2.10E-21 |
| rs9260164 | 6 | 29911684 | C | T | -9.86E-03 | 1.58E-03 | 4.40E-10 | -1.52E-02 | 1.61E-03 | 5.70E-21 |
| rs7498665 | 16 | 28883241 | A | G | -1.73E-02 | 1.40E-03 | 5.90E-35 | -1.34E-02 | 1.43E-03 | 6.00E-21 |
| rs57636386 | 18 | 58048295 | T | C | 2.47E-02 | 2.48E-03 | 2.70E-23 | 2.35E-02 | 2.54E-03 | 2.00E-20 |
| rs4688359 | 3 | 61198880 | C | T | 1.05E-02 | 1.42E-03 | 1.50E-13 | 1.30E-02 | 1.45E-03 | 2.10E-19 |
| rs12140153 | 1 | 62579891 | G | T | 2.14E-02 | 2.40E-03 | 4.20E-19 | 2.18E-02 | 2.45E-03 | 6.00E-19 |
| rs7355953 | 3 | 85792137 | T | C | -1.06E-02 | 1.67E-03 | 2.00E-10 | -1.52E-02 | 1.71E-03 | 7.40E-19 |
| rs41310284 | 10 | 102447647 | C | A | 1.77E-02 | 2.29E-03 | 9.80E-15 | 2.06E-02 | 2.34E-03 | 1.40E-18 |
| rs9603697 | 13 | 40783323 | C | T | -8.53E-03 | 1.46E-03 | 5.70E-09 | -1.30E-02 | 1.50E-03 | 4.60E-18 |
| rs2234458 | 11 | 65639374 | C | T | 1.28E-02 | 1.43E-03 | 2.50E-19 | 1.26E-02 | 1.46E-03 | 6.00E-18 |
| rs200744777 | 20 | 6609610 | T | G | -9.38E-03 | 1.39E-03 | 1.70E-11 | -1.21E-02 | 1.42E-03 | 1.50E-17 |
| rs13047416 | 21 | 40309436 | C | G | 8.66E-03 | 1.42E-03 | 1.10E-09 | 1.23E-02 | 1.45E-03 | 1.80E-17 |
| rs55726687 | 12 | 991306 | G | A | -1.38E-02 | 1.68E-03 | 3.00E-16 | -1.45E-02 | 1.72E-03 | 3.20E-17 |
| rs4432271 | 16 | 20245283 | C | T | -1.16E-02 | 2.06E-03 | 2.10E-08 | -1.75E-02 | 2.11E-03 | 9.00E-17 |
| rs9317002 | 13 | 59175727 | C | A | -1.14E-02 | 1.38E-03 | 1.90E-16 | -1.15E-02 | 1.41E-03 | 3.70E-16 |
| rs1576655 | 13 | 79587841 | A | C | -1.13E-02 | 1.43E-03 | 1.90E-15 | -1.18E-02 | 1.46E-03 | 4.40E-16 |
| rs59714050 | 3 | 141267294 | T | A | -1.94E-02 | 2.75E-03 | 2.00E-12 | -2.28E-02 | 2.81E-03 | 4.90E-16 |
| rs7656673 | 4 | 30840331 | A | G | -8.28E-03 | 1.40E-03 | 3.20E-09 | -1.16E-02 | 1.43E-03 | 6.20E-16 |
| rs13107325 | 4 | 103188709 | C | T | -2.87E-02 | 2.61E-03 | 3.10E-28 | -2.15E-02 | 2.66E-03 | 6.70E-16 |
| rs11150745 | 17 | 78757626 | A | G | 1.38E-02 | 1.48E-03 | 9.20E-21 | 1.22E-02 | 1.51E-03 | 7.50E-16 |
| rs12484438 | 22 | 40558064 | T | C | 1.29E-02 | 1.45E-03 | 6.70E-19 | 1.18E-02 | 1.48E-03 | 1.80E-15 |
| rs2735556 | 3 | 88105360 | T | C | 1.29E-02 | 2.15E-03 | 1.70E-09 | 1.72E-02 | 2.20E-03 | 5.10E-15 |
| rs10796828 | 11 | 69490346 | T | G | -7.85E-03 | 1.43E-03 | 3.70E-08 | -1.13E-02 | 1.46E-03 | 6.90E-15 |
| rs12606230 | 18 | 52492252 | T | C | -1.17E-02 | 1.62E-03 | 6.20E-13 | -1.27E-02 | 1.66E-03 | 1.70E-14 |
| rs17399739 | 10 | 87490850 | A | G | -1.78E-02 | 2.71E-03 | 5.90E-11 | -2.10E-02 | 2.77E-03 | 3.90E-14 |
| rs39862 | 5 | 66185151 | T | C | 9.63E-03 | 1.52E-03 | 2.60E-10 | 1.16E-02 | 1.56E-03 | 7.50E-14 |
| rs11525873 | 7 | 138817193 | T | C | 1.48E-02 | 2.31E-03 | 1.40E-10 | 1.75E-02 | 2.36E-03 | 1.30E-13 |
| rs12883788 | 14 | 33303540 | C | T | -1.24E-02 | 1.38E-03 | 2.30E-19 | -1.00E-02 | 1.41E-03 | 1.10E-12 |
| rs3748126 | 7 | 76632736 | C | G | 1.65E-02 | 1.82E-03 | 1.30E-19 | 1.31E-02 | 1.86E-03 | 1.70E-12 |
| rs630602 | 1 | 54728864 | G | C | -7.73E-03 | 1.41E-03 | 4.10E-08 | -1.01E-02 | 1.44E-03 | 1.80E-12 |
| rs957512 | 9 | 120405705 | T | C | 8.80E-03 | 1.46E-03 | 1.80E-09 | 1.03E-02 | 1.49E-03 | 4.50E-12 |
| rs111768603 | 3 | 42329113 | G | T | 1.59E-02 | 2.19E-03 | 3.80E-13 | 1.54E-02 | 2.24E-03 | 5.60E-12 |
| rs7619139 | 3 | 25110415 | T | A | -8.84E-03 | 1.40E-03 | 2.40E-10 | -9.80E-03 | 1.43E-03 | 6.50E-12 |
| rs2968973 | 4 | 130740404 | C | T | 8.11E-03 | 1.43E-03 | 1.40E-08 | 9.93E-03 | 1.46E-03 | 1.10E-11 |
| rs10423928 | 19 | 46182304 | T | A | 2.08E-02 | 1.73E-03 | 4.20E-33 | 1.18E-02 | 1.77E-03 | 2.60E-11 |
| rs1320903 | 3 | 131758077 | G | A | -1.46E-02 | 1.47E-03 | 2.60E-23 | -1.00E-02 | 1.50E-03 | 2.70E-11 |
| rs6265 | 11 | 27679916 | C | T | 2.45E-02 | 1.75E-03 | 1.50E-44 | 1.17E-02 | 1.79E-03 | 6.30E-11 |
| rs55896564 | 8 | 11447093 | G | A | 1.17E-02 | 1.38E-03 | 2.60E-17 | 9.11E-03 | 1.41E-03 | 1.10E-10 |
| rs34517439 | 1 | 78450517 | C | A | -2.47E-02 | 2.12E-03 | 1.90E-31 | -1.38E-02 | 2.16E-03 | 2.00E-10 |
| rs559231 | 18 | 39644247 | G | T | -9.52E-03 | 1.41E-03 | 1.50E-11 | -9.10E-03 | 1.44E-03 | 2.70E-10 |
| rs9922288 | 16 | 24550930 | A | G | 9.39E-03 | 1.64E-03 | 9.80E-09 | 1.05E-02 | 1.67E-03 | 3.00E-10 |
| rs1788808 | 18 | 21090023 | A | G | 1.27E-02 | 1.37E-03 | 1.90E-20 | 8.81E-03 | 1.40E-03 | 3.40E-10 |
| rs34811474 | 4 | 25408838 | G | A | 1.66E-02 | 1.63E-03 | 2.00E-24 | 1.02E-02 | 1.66E-03 | 8.20E-10 |
| rs2307111 | 5 | 75003678 | T | C | 1.68E-02 | 1.40E-03 | 6.90E-33 | 8.74E-03 | 1.43E-03 | 1.10E-09 |
| rs7827182 | 8 | 8380471 | G | C | -1.12E-02 | 1.37E-03 | 2.90E-16 | -8.49E-03 | 1.40E-03 | 1.40E-09 |
| rs1852006 | 7 | 77829768 | G | A | 8.83E-03 | 1.43E-03 | 6.70E-10 | 8.77E-03 | 1.46E-03 | 1.90E-09 |
| rs11496125 | 7 | 103417557 | C | T | -1.09E-02 | 1.39E-03 | 4.80E-15 | -8.54E-03 | 1.42E-03 | 2.00E-09 |
| rs4739558 | 8 | 38337264 | A | G | 8.10E-03 | 1.40E-03 | 7.40E-09 | 8.30E-03 | 1.43E-03 | 6.70E-09 |
| rs2712667 | 12 | 99588917 | G | C | 9.62E-03 | 1.44E-03 | 2.10E-11 | 8.49E-03 | 1.47E-03 | 7.30E-09 |
| rs11134679 | 5 | 170623391 | A | G | -1.24E-02 | 1.48E-03 | 5.20E-17 | -8.60E-03 | 1.51E-03 | 1.30E-08 |
| rs1296328 | 4 | 137083193 | A | C | 1.17E-02 | 1.39E-03 | 2.60E-17 | 8.04E-03 | 1.42E-03 | 1.40E-08 |
| rs6870983 | 5 | 87697533 | C | T | 1.38E-02 | 1.67E-03 | 1.60E-16 | 9.70E-03 | 1.71E-03 | 1.40E-08 |
| rs6601451 | 8 | 10243681 | C | G | 1.16E-02 | 1.37E-03 | 3.70E-17 | 7.81E-03 | 1.40E-03 | 2.60E-08 |
| rs7145882 | 14 | 103255461 | T | C | 1.15E-02 | 1.45E-03 | 2.20E-15 | 8.19E-03 | 1.48E-03 | 2.90E-08 |
| rs55658481 | 2 | 219284215 | G | A | -8.97E-03 | 1.44E-03 | 5.30E-10 | -8.16E-03 | 1.48E-03 | 3.30E-08 |
| rs4764949 | 12 | 103658096 | A | G | 1.19E-02 | 1.47E-03 | 3.80E-16 | 8.01E-03 | 1.50E-03 | 8.50E-08 |
| rs112253053 | 19 | 19425145 | T | A | 1.38E-02 | 1.87E-03 | 1.80E-13 | 1.01E-02 | 1.91E-03 | 1.10E-07 |
| rs28350 | 3 | 42418446 | A | G | 1.26E-02 | 1.79E-03 | 1.70E-12 | 9.70E-03 | 1.83E-03 | 1.20E-07 |
| rs512121 | 18 | 7548501 | T | C | 1.06E-02 | 1.75E-03 | 1.50E-09 | 9.31E-03 | 1.79E-03 | 1.90E-07 |
| rs72673947 | 8 | 118884379 | A | G | -1.40E-02 | 2.23E-03 | 3.30E-10 | -1.18E-02 | 2.28E-03 | 2.10E-07 |
| rs12951079 | 17 | 34933059 | G | A | 1.08E-02 | 1.41E-03 | 1.60E-14 | 7.42E-03 | 1.43E-03 | 2.30E-07 |
| rs61992671 | 14 | 101531854 | A | G | 9.88E-03 | 1.43E-03 | 5.70E-12 | 7.58E-03 | 1.47E-03 | 2.30E-07 |
| rs10146997 | 14 | 79945162 | A | G | -1.60E-02 | 1.65E-03 | 3.20E-22 | -8.69E-03 | 1.69E-03 | 2.60E-07 |
| rs62004865 | 15 | 74207695 | T | A | -1.36E-02 | 2.24E-03 | 1.40E-09 | -1.18E-02 | 2.29E-03 | 2.70E-07 |
| rs869400 | 3 | 185826740 | T | G | -1.83E-02 | 1.77E-03 | 3.50E-25 | -9.22E-03 | 1.81E-03 | 3.30E-07 |
| rs9366863 | 6 | 34688946 | T | C | 1.74E-02 | 1.46E-03 | 8.50E-33 | 7.54E-03 | 1.49E-03 | 4.10E-07 |
| rs1423534 | 5 | 63977000 | G | A | 7.71E-03 | 1.39E-03 | 3.20E-08 | 7.14E-03 | 1.42E-03 | 5.30E-07 |
| rs3861871 | 9 | 129424719 | A | G | -8.29E-03 | 1.39E-03 | 2.60E-09 | -7.12E-03 | 1.42E-03 | 5.40E-07 |
| rs4971239 | 1 | 203491150 | G | A | -1.15E-02 | 1.84E-03 | 4.50E-10 | -9.43E-03 | 1.88E-03 | 5.50E-07 |
| rs67257872 | 11 | 8530218 | A | G | 1.06E-02 | 1.38E-03 | 1.50E-14 | 7.02E-03 | 1.41E-03 | 6.30E-07 |
| rs8089514 | 18 | 69224478 | T | A | -8.17E-03 | 1.44E-03 | 1.40E-08 | -7.32E-03 | 1.47E-03 | 6.30E-07 |
| rs2051559 | 4 | 3298800 | T | C | -1.39E-02 | 2.03E-03 | 8.30E-12 | -1.03E-02 | 2.07E-03 | 6.60E-07 |
| rs8015400 | 14 | 25930988 | C | A | -1.26E-02 | 1.47E-03 | 6.90E-18 | -7.34E-03 | 1.50E-03 | 9.80E-07 |
| rs73026723 | 19 | 31017177 | C | T | 1.41E-02 | 1.90E-03 | 1.30E-13 | 9.49E-03 | 1.94E-03 | 1.00E-06 |
| rs61909165 | 11 | 134589355 | T | A | -1.23E-02 | 1.82E-03 | 1.60E-11 | -8.87E-03 | 1.86E-03 | 1.90E-06 |
| rs8008772 | 14 | 88321884 | A | T | -9.26E-03 | 1.59E-03 | 5.70E-09 | -7.72E-03 | 1.62E-03 | 2.00E-06 |
| rs6679458 | 1 | 96946253 | G | T | -1.16E-02 | 1.39E-03 | 9.10E-17 | -6.70E-03 | 1.42E-03 | 2.40E-06 |
| rs9788550 | 14 | 29681138 | G | C | 1.38E-02 | 1.60E-03 | 5.60E-18 | 7.68E-03 | 1.63E-03 | 2.40E-06 |
| rs1834144 | 18 | 40744790 | C | A | 7.93E-03 | 1.42E-03 | 2.50E-08 | 6.82E-03 | 1.45E-03 | 2.70E-06 |
| rs10404726 | 19 | 18834514 | C | T | 1.23E-02 | 1.38E-03 | 4.70E-19 | 6.56E-03 | 1.41E-03 | 3.20E-06 |
| rs34898535 | 16 | 31025641 | C | T | 1.43E-02 | 1.41E-03 | 4.80E-24 | 6.67E-03 | 1.45E-03 | 3.90E-06 |
| rs112859723 | 3 | 131625376 | T | C | 1.11E-02 | 1.91E-03 | 5.50E-09 | 8.82E-03 | 1.95E-03 | 6.40E-06 |
| rs138329430 | 1 | 174619318 | G | A | -1.28E-02 | 2.19E-03 | 5.40E-09 | -1.01E-02 | 2.24E-03 | 7.20E-06 |
| rs28465175 | 15 | 53427155 | A | G | 1.50E-02 | 2.70E-03 | 2.60E-08 | 1.24E-02 | 2.76E-03 | 7.20E-06 |
| rs73982435 | 17 | 31473455 | C | T | 9.43E-03 | 1.67E-03 | 1.60E-08 | 7.56E-03 | 1.70E-03 | 9.00E-06 |
| rs6669341 | 1 | 47678458 | A | G | 1.07E-02 | 1.39E-03 | 1.30E-14 | 6.28E-03 | 1.42E-03 | 9.60E-06 |
| rs10969334 | 9 | 29717279 | C | A | 8.67E-03 | 1.41E-03 | 6.90E-10 | 6.34E-03 | 1.44E-03 | 1.00E-05 |
| rs11223204 | 11 | 132652554 | A | G | -8.74E-03 | 1.39E-03 | 3.00E-10 | -6.17E-03 | 1.42E-03 | 1.30E-05 |
| rs862320 | 16 | 69651866 | C | T | 1.44E-02 | 1.40E-03 | 6.20E-25 | 6.19E-03 | 1.43E-03 | 1.40E-05 |
| rs113230003 | 19 | 18460956 | G | A | 1.26E-02 | 1.58E-03 | 1.20E-15 | 7.00E-03 | 1.61E-03 | 1.40E-05 |
| rs3814883 | 16 | 29994922 | C | T | -1.48E-02 | 1.38E-03 | 8.10E-27 | -6.09E-03 | 1.41E-03 | 1.50E-05 |
| rs11976084 | 7 | 137437156 | C | T | -8.57E-03 | 1.52E-03 | 1.70E-08 | -6.61E-03 | 1.55E-03 | 2.10E-05 |
| rs17773370 | 18 | 57951433 | G | A | -1.59E-02 | 2.76E-03 | 8.70E-09 | -1.19E-02 | 2.82E-03 | 2.60E-05 |
| rs12454712 | 18 | 60845884 | T | C | -8.11E-03 | 1.42E-03 | 1.00E-08 | -6.03E-03 | 1.45E-03 | 3.10E-05 |
| rs815163 | 1 | 190294726 | T | C | 1.08E-02 | 1.38E-03 | 6.10E-15 | 5.84E-03 | 1.41E-03 | 3.40E-05 |
| rs6938973 | 6 | 98421721 | T | C | -1.18E-02 | 1.40E-03 | 3.50E-17 | 5.90E-03 | 1.43E-03 | 3.80E-05 |
| rs827803 | 3 | 157920266 | G | T | 7.87E-03 | 1.38E-03 | 1.20E-08 | 5.81E-03 | 1.41E-03 | 3.90E-05 |
| rs7752998 | 6 | 34398358 | A | T | 1.02E-02 | 1.75E-03 | 4.60E-09 | 7.34E-03 | 1.79E-03 | 3.90E-05 |
| rs59428052 | 2 | 53861389 | A | G | 1.15E-02 | 2.00E-03 | 9.80E-09 | 8.33E-03 | 2.04E-03 | 4.60E-05 |
| rs10736156 | 10 | 104019447 | C | A | -1.18E-02 | 1.89E-03 | 3.80E-10 | -7.85E-03 | 1.93E-03 | 4.80E-05 |
| rs329651 | 11 | 133767622 | G | T | -1.11E-02 | 1.74E-03 | 1.70E-10 | -7.17E-03 | 1.77E-03 | 5.20E-05 |
| rs34778589 | 22 | 50709957 | A | C | -1.43E-02 | 2.50E-03 | 9.80E-09 | -1.03E-02 | 2.55E-03 | 5.30E-05 |
| rs55838622 | 5 | 95711605 | A | C | -9.90E-03 | 1.64E-03 | 1.70E-09 | -6.73E-03 | 1.68E-03 | 6.10E-05 |
| rs4790292 | 17 | 1824305 | C | A | 1.63E-02 | 1.91E-03 | 1.30E-17 | 7.82E-03 | 1.95E-03 | 6.10E-05 |
| rs35775580 | 7 | 130420740 | A | G | 1.80E-02 | 3.24E-03 | 2.70E-08 | 1.32E-02 | 3.31E-03 | 6.30E-05 |
| rs34298980 | 6 | 40409243 | T | C | 1.33E-02 | 1.44E-03 | 4.40E-20 | 5.88E-03 | 1.48E-03 | 6.70E-05 |
| rs6606686 | 12 | 110903380 | G | C | 9.75E-03 | 1.47E-03 | 3.40E-11 | 5.96E-03 | 1.50E-03 | 7.10E-05 |
| rs4671328 | 2 | 58935282 | T | G | 1.32E-02 | 1.39E-03 | 2.10E-21 | 5.62E-03 | 1.42E-03 | 7.30E-05 |
| rs1477290 | 5 | 87988934 | T | C | -1.99E-02 | 2.01E-03 | 3.50E-23 | -8.15E-03 | 2.05E-03 | 7.30E-05 |
| rs62277889 | 2 | 198783693 | C | T | 1.06E-02 | 1.58E-03 | 2.10E-11 | 6.41E-03 | 1.62E-03 | 7.40E-05 |
| rs4467770 | 6 | 12086826 | G | A | -9.87E-03 | 1.55E-03 | 2.00E-10 | -6.25E-03 | 1.59E-03 | 8.10E-05 |
| rs28711392 | 11 | 13349559 | T | C | 1.18E-02 | 1.43E-03 | 2.10E-16 | 5.76E-03 | 1.46E-03 | 8.40E-05 |
| rs1517037 | 18 | 56878274 | C | T | 9.92E-03 | 1.76E-03 | 1.60E-08 | 7.03E-03 | 1.79E-03 | 8.90E-05 |
| rs6575340 | 14 | 94023972 | G | A | -1.33E-02 | 1.43E-03 | 1.20E-20 | -5.69E-03 | 1.46E-03 | 9.70E-05 |
| rs2678204 | 1 | 201800511 | T | G | -1.50E-02 | 1.44E-03 | 2.70E-25 | -5.75E-03 | 1.48E-03 | 9.90E-05 |
| rs35193668 | 13 | 33092929 | C | T | 1.05E-02 | 1.43E-03 | 2.50E-13 | 5.68E-03 | 1.46E-03 | 1.00E-04 |
| rs8111074 | 19 | 51776117 | G | T | 8.39E-03 | 1.52E-03 | 3.20E-08 | 6.01E-03 | 1.55E-03 | 1.00E-04 |
| rs2417998 | 9 | 111958746 | C | G | 8.41E-03 | 1.52E-03 | 2.80E-08 | 5.97E-03 | 1.55E-03 | 1.20E-04 |
| rs7453694 | 6 | 51739528 | C | T | -9.79E-03 | 1.52E-03 | 1.20E-10 | -5.98E-03 | 1.55E-03 | 1.20E-04 |
| rs114593013 | 3 | 84113491 | A | G | 1.97E-02 | 2.94E-03 | 2.10E-11 | 1.16E-02 | 3.01E-03 | 1.20E-04 |
| rs10749659 | 1 | 151033979 | C | T | 1.02E-02 | 1.63E-03 | 4.40E-10 | 6.39E-03 | 1.67E-03 | 1.30E-04 |
| rs57590313 | 4 | 113323430 | C | A | -1.02E-02 | 1.79E-03 | 1.30E-08 | -6.98E-03 | 1.83E-03 | 1.30E-04 |
| rs75706763 | 2 | 145669168 | A | G | -1.77E-02 | 3.14E-03 | 1.70E-08 | -1.21E-02 | 3.21E-03 | 1.50E-04 |
| rs28462076 | 4 | 65696174 | A | G | 9.39E-03 | 1.62E-03 | 6.70E-09 | 6.24E-03 | 1.66E-03 | 1.60E-04 |
| rs59893724 | 5 | 80830788 | A | G | 1.14E-02 | 1.60E-03 | 8.70E-13 | 6.14E-03 | 1.63E-03 | 1.70E-04 |
| rs1582931 | 5 | 122657199 | G | A | 9.66E-03 | 1.38E-03 | 3.00E-12 | 5.30E-03 | 1.41E-03 | 1.80E-04 |
| rs10799778 | 1 | 23313353 | T | G | 1.26E-02 | 1.84E-03 | 7.80E-12 | 7.05E-03 | 1.88E-03 | 1.80E-04 |
| rs12187066 | 5 | 88800355 | A | G | -1.02E-02 | 1.58E-03 | 9.80E-11 | -5.99E-03 | 1.61E-03 | 2.00E-04 |
| rs79236537 | 5 | 86727690 | G | T | -2.99E-02 | 4.89E-03 | 1.00E-09 | -1.85E-02 | 5.00E-03 | 2.20E-04 |
| rs3931548 | 9 | 103113652 | C | A | -1.05E-02 | 1.44E-03 | 2.80E-13 | -5.41E-03 | 1.47E-03 | 2.30E-04 |
| rs1286138 | 14 | 91485445 | T | G | -9.00E-03 | 1.47E-03 | 8.40E-10 | -5.44E-03 | 1.50E-03 | 2.80E-04 |
| rs4482463 | 2 | 205375909 | C | A | 1.89E-02 | 2.57E-03 | 2.30E-13 | 9.47E-03 | 2.63E-03 | 3.20E-04 |
| rs1805123 | 7 | 150645534 | T | G | 1.10E-02 | 1.59E-03 | 4.10E-12 | 5.84E-03 | 1.63E-03 | 3.30E-04 |
| rs12788343 | 11 | 131452912 | T | C | -9.65E-03 | 1.39E-03 | 4.30E-12 | -5.10E-03 | 1.42E-03 | 3.40E-04 |
| rs142315514 | 1 | 147050816 | C | A | -2.14E-02 | 3.79E-03 | 1.70E-08 | -1.39E-02 | 3.87E-03 | 3.50E-04 |
| rs2450444 | 10 | 93010383 | G | A | 8.07E-03 | 1.44E-03 | 2.00E-08 | 5.24E-03 | 1.47E-03 | 3.60E-04 |
| rs2253310 | 6 | 108888593 | C | G | -1.06E-02 | 1.42E-03 | 9.00E-14 | -5.14E-03 | 1.45E-03 | 3.90E-04 |
| rs56803094 | 15 | 99222509 | A | G | 9.58E-03 | 1.65E-03 | 5.80E-09 | 5.94E-03 | 1.68E-03 | 4.00E-04 |
| rs6963840 | 7 | 78144371 | C | T | -1.29E-02 | 1.89E-03 | 8.50E-12 | -6.84E-03 | 1.93E-03 | 4.10E-04 |
| rs12147845 | 14 | 101144596 | C | T | -1.36E-02 | 2.15E-03 | 2.60E-10 | -7.75E-03 | 2.19E-03 | 4.10E-04 |
| rs1296685 | 22 | 18230964 | A | G | -9.49E-03 | 1.70E-03 | 2.20E-08 | -6.09E-03 | 1.73E-03 | 4.40E-04 |
| rs115866895 | 1 | 1592638 | A | G | 1.20E-02 | 1.57E-03 | 1.70E-14 | 5.60E-03 | 1.60E-03 | 4.70E-04 |
| rs4677813 | 3 | 194863860 | T | C | 8.93E-03 | 1.58E-03 | 1.60E-08 | 5.60E-03 | 1.62E-03 | 5.30E-04 |
| rs72618637 | 2 | 48953979 | T | A | 9.99E-03 | 1.77E-03 | 1.80E-08 | 6.29E-03 | 1.81E-03 | 5.30E-04 |
| rs6548220 | 2 | 225951 | A | G | 9.36E-03 | 1.48E-03 | 2.80E-10 | 5.24E-03 | 1.52E-03 | 5.50E-04 |
| rs13329943 | 16 | 24733751 | C | T | -1.13E-02 | 1.55E-03 | 3.20E-13 | -5.46E-03 | 1.59E-03 | 5.70E-04 |
| rs12634936 | 3 | 147716498 | T | C | -1.81E-02 | 3.15E-03 | 8.80E-09 | -1.11E-02 | 3.22E-03 | 5.80E-04 |
| rs12462975 | 19 | 30272202 | G | A | -1.15E-02 | 1.47E-03 | 5.90E-15 | -5.15E-03 | 1.50E-03 | 6.00E-04 |
| rs11633022 | 15 | 73074890 | C | A | -1.09E-02 | 1.44E-03 | 4.30E-14 | -5.04E-03 | 1.47E-03 | 6.30E-04 |
| rs3823674 | 7 | 50571996 | C | T | 7.66E-03 | 1.39E-03 | 3.50E-08 | 4.84E-03 | 1.42E-03 | 6.40E-04 |
| rs6843852 | 4 | 162132758 | C | T | -8.72E-03 | 1.37E-03 | 2.00E-10 | -4.77E-03 | 1.40E-03 | 6.70E-04 |
| rs1633418 | 22 | 20091756 | T | C | 7.92E-03 | 1.41E-03 | 2.00E-08 | 4.89E-03 | 1.44E-03 | 6.90E-04 |
| rs6798941 | 3 | 52893465 | C | T | -1.10E-02 | 1.51E-03 | 2.70E-13 | -5.21E-03 | 1.54E-03 | 7.20E-04 |
| rs9814758 | 3 | 123062657 | T | G | 8.39E-03 | 1.44E-03 | 5.40E-09 | 4.89E-03 | 1.47E-03 | 8.80E-04 |
| rs7102934 | 11 | 84648068 | T | C | -9.08E-03 | 1.49E-03 | 1.20E-09 | -5.07E-03 | 1.53E-03 | 8.90E-04 |
| rs113962925 | 17 | 46044446 | C | T | -1.71E-02 | 2.64E-03 | 8.60E-11 | -8.95E-03 | 2.69E-03 | 8.90E-04 |
| rs78565420 | 8 | 85703065 | C | T | -1.88E-02 | 3.15E-03 | 2.70E-09 | -1.07E-02 | 3.22E-03 | 9.20E-04 |
| rs112875651 | 8 | 126506694 | G | A | -8.35E-03 | 1.42E-03 | 4.30E-09 | -4.79E-03 | 1.45E-03 | 9.70E-04 |
| rs811054 | 16 | 72251132 | C | T | -8.64E-03 | 1.38E-03 | 4.40E-10 | -4.66E-03 | 1.41E-03 | 9.80E-04 |
| rs80082536 | 3 | 35195311 | A | G | -1.29E-02 | 2.12E-03 | 1.30E-09 | -7.15E-03 | 2.17E-03 | 9.90E-04 |
| rs8038574 | 15 | 95275890 | T | C | 9.07E-03 | 1.45E-03 | 4.00E-10 | 4.85E-03 | 1.48E-03 | 1.10E-03 |
| rs13061117 | 3 | 181186466 | T | C | -1.47E-02 | 2.42E-03 | 1.30E-09 | -7.99E-03 | 2.48E-03 | 1.20E-03 |
| rs273505 | 19 | 18217147 | T | C | -1.01E-02 | 1.39E-03 | 3.10E-13 | -4.52E-03 | 1.42E-03 | 1.40E-03 |
| rs12517187 | 5 | 112444682 | C | T | -8.25E-03 | 1.39E-03 | 2.80E-09 | -4.51E-03 | 1.42E-03 | 1.50E-03 |
| rs2289379 | 7 | 44804225 | C | T | 9.76E-03 | 1.41E-03 | 4.10E-12 | 4.57E-03 | 1.44E-03 | 1.50E-03 |
| rs2186118 | 1 | 66456465 | C | A | 9.86E-03 | 1.51E-03 | 6.90E-11 | 4.90E-03 | 1.55E-03 | 1.50E-03 |
| rs62171698 | 2 | 143959096 | C | A | -1.21E-02 | 1.97E-03 | 8.80E-10 | -6.39E-03 | 2.01E-03 | 1.50E-03 |
| rs1167311 | 1 | 49996959 | G | A | 1.23E-02 | 1.48E-03 | 1.20E-16 | 4.77E-03 | 1.51E-03 | 1.60E-03 |
| rs2875762 | 6 | 124925032 | G | C | -1.10E-02 | 1.60E-03 | 6.00E-12 | -5.17E-03 | 1.64E-03 | 1.60E-03 |
| rs1436348 | 3 | 104612668 | A | G | -9.66E-03 | 1.39E-03 | 3.50E-12 | -4.46E-03 | 1.42E-03 | 1.70E-03 |
| rs845084 | 10 | 125220036 | G | A | -9.85E-03 | 1.57E-03 | 3.70E-10 | -5.04E-03 | 1.61E-03 | 1.70E-03 |
| rs724623 | 14 | 47303577 | A | C | 1.02E-02 | 1.37E-03 | 9.00E-14 | 4.37E-03 | 1.40E-03 | 1.80E-03 |
| rs7103389 | 11 | 881639 | T | C | -9.46E-03 | 1.42E-03 | 2.90E-11 | -4.53E-03 | 1.45E-03 | 1.80E-03 |
| rs34994596 | 15 | 80991447 | T | C | 1.12E-02 | 1.50E-03 | 1.10E-13 | 4.77E-03 | 1.53E-03 | 1.80E-03 |
| rs544200874 | 4 | 20124826 | C | T | -1.77E-02 | 2.71E-03 | 7.20E-11 | -8.65E-03 | 2.77E-03 | 1.80E-03 |
| rs4672338 | 2 | 60217457 | C | T | -8.35E-03 | 1.45E-03 | 8.00E-09 | -4.60E-03 | 1.48E-03 | 1.90E-03 |
| rs4648450 | 1 | 2723214 | C | A | 9.80E-03 | 1.38E-03 | 1.20E-12 | 4.35E-03 | 1.41E-03 | 2.00E-03 |
| rs3125326 | 10 | 63053788 | A | C | -7.89E-03 | 1.42E-03 | 2.70E-08 | -4.48E-03 | 1.45E-03 | 2.00E-03 |
| rs11856579 | 15 | 78012688 | G | A | 9.18E-03 | 1.55E-03 | 3.40E-09 | 4.88E-03 | 1.59E-03 | 2.10E-03 |
| rs114263339 | 5 | 50932343 | C | T | -2.53E-02 | 4.33E-03 | 5.20E-09 | -1.36E-02 | 4.42E-03 | 2.10E-03 |
| rs1477890 | 4 | 18511738 | A | G | -9.78E-03 | 1.37E-03 | 1.10E-12 | -4.27E-03 | 1.40E-03 | 2.40E-03 |
| rs6029180 | 20 | 39178923 | A | G | -8.19E-03 | 1.48E-03 | 3.10E-08 | -4.57E-03 | 1.51E-03 | 2.40E-03 |
| rs7749708 | 6 | 153375907 | C | T | -9.98E-03 | 1.51E-03 | 3.70E-11 | -4.69E-03 | 1.54E-03 | 2.40E-03 |
| rs1222216 | 11 | 30346052 | C | T | 1.22E-02 | 1.64E-03 | 1.10E-13 | 5.09E-03 | 1.67E-03 | 2.40E-03 |
| rs35894137 | 8 | 43071838 | C | T | 1.42E-02 | 2.52E-03 | 1.60E-08 | 7.82E-03 | 2.57E-03 | 2.40E-03 |
| rs72652703 | 8 | 67209548 | T | C | 9.92E-03 | 1.58E-03 | 3.20E-10 | 4.87E-03 | 1.61E-03 | 2.50E-03 |
| rs12363672 | 11 | 55684028 | A | C | -2.38E-02 | 4.29E-03 | 2.80E-08 | -1.32E-02 | 4.38E-03 | 2.50E-03 |
| rs74929176 | 17 | 54905494 | C | T | 9.05E-03 | 1.65E-03 | 4.40E-08 | 5.04E-03 | 1.69E-03 | 2.80E-03 |
| rs17619860 | 8 | 87779603 | T | C | -1.04E-02 | 1.86E-03 | 2.20E-08 | -5.67E-03 | 1.90E-03 | 2.80E-03 |
| rs35221880 | 12 | 133301500 | T | C | 8.57E-03 | 1.55E-03 | 3.50E-08 | 4.72E-03 | 1.59E-03 | 2.90E-03 |
| rs11000993 | 10 | 76084111 | T | C | -1.38E-02 | 2.08E-03 | 3.10E-11 | -6.34E-03 | 2.12E-03 | 2.90E-03 |
| rs35809007 | 2 | 47019521 | G | A | 1.06E-02 | 1.43E-03 | 1.20E-13 | 4.34E-03 | 1.46E-03 | 3.00E-03 |
| rs9480184 | 6 | 155987788 | C | T | -9.86E-03 | 1.68E-03 | 4.90E-09 | -5.11E-03 | 1.72E-03 | 3.00E-03 |
| rs66460909 | 20 | 51195387 | G | A | 1.57E-02 | 1.75E-03 | 2.90E-19 | 5.27E-03 | 1.79E-03 | 3.10E-03 |
| rs7924036 | 10 | 65191645 | G | T | 9.63E-03 | 1.37E-03 | 2.20E-12 | 4.10E-03 | 1.40E-03 | 3.40E-03 |
| rs4575195 | 10 | 114765747 | C | A | 9.59E-03 | 1.48E-03 | 9.00E-11 | 4.43E-03 | 1.51E-03 | 3.40E-03 |
| rs4148155 | 4 | 89054667 | A | G | 1.43E-02 | 2.16E-03 | 2.90E-11 | 6.42E-03 | 2.21E-03 | 3.60E-03 |
| rs9843653 | 3 | 49920571 | T | C | -1.76E-02 | 1.37E-03 | 1.50E-37 | -4.07E-03 | 1.40E-03 | 3.70E-03 |
| rs7141912 | 14 | 35649431 | A | T | 1.18E-02 | 2.07E-03 | 1.10E-08 | 6.14E-03 | 2.11E-03 | 3.70E-03 |
| rs148137538 | 1 | 173399677 | A | G | 2.50E-02 | 4.58E-03 | 4.40E-08 | 1.35E-02 | 4.68E-03 | 3.80E-03 |
| rs45521740 | 19 | 2245622 | G | A | -1.80E-02 | 2.95E-03 | 1.00E-09 | -8.69E-03 | 3.01E-03 | 3.90E-03 |
| rs13218383 | 6 | 120173501 | C | G | 9.32E-03 | 1.45E-03 | 1.40E-10 | 4.22E-03 | 1.48E-03 | 4.40E-03 |
| rs3902951 | 14 | 69789755 | T | G | -1.05E-02 | 1.63E-03 | 1.10E-10 | -4.73E-03 | 1.67E-03 | 4.50E-03 |
| rs680071 | 11 | 103088414 | T | C | -1.17E-02 | 2.11E-03 | 2.80E-08 | -6.14E-03 | 2.16E-03 | 4.50E-03 |
| rs412243 | 16 | 339672 | T | C | 1.05E-02 | 1.42E-03 | 1.40E-13 | 4.08E-03 | 1.45E-03 | 4.70E-03 |
| rs10791113 | 11 | 130873165 | A | G | -8.57E-03 | 1.38E-03 | 4.60E-10 | -3.96E-03 | 1.40E-03 | 4.80E-03 |
| rs113132247 | 9 | 131026108 | G | A | -1.23E-02 | 1.91E-03 | 1.10E-10 | -5.50E-03 | 1.95E-03 | 4.80E-03 |
| rs651533 | 1 | 82375561 | T | A | 9.30E-03 | 1.59E-03 | 5.60E-09 | 4.56E-03 | 1.63E-03 | 5.20E-03 |
| rs34292685 | 11 | 64049021 | C | T | 1.27E-02 | 1.86E-03 | 7.90E-12 | 5.31E-03 | 1.90E-03 | 5.20E-03 |
| rs17029006 | 3 | 12329452 | C | T | 1.04E-02 | 1.55E-03 | 2.20E-11 | 4.42E-03 | 1.59E-03 | 5.40E-03 |
| rs7424120 | 2 | 59313974 | C | T | 1.42E-02 | 1.40E-03 | 4.00E-24 | 3.98E-03 | 1.43E-03 | 5.50E-03 |
| rs544957562 | 9 | 33978015 | A | T | 1.24E-02 | 2.02E-03 | 9.00E-10 | 5.74E-03 | 2.07E-03 | 5.50E-03 |
| rs1250597 | 10 | 81010250 | A | G | -8.54E-03 | 1.40E-03 | 1.00E-09 | 3.94E-03 | 1.43E-03 | 5.80E-03 |
| rs2083323 | 18 | 1856272 | G | A | -1.04E-02 | 1.80E-03 | 7.30E-09 | -5.03E-03 | 1.84E-03 | 6.20E-03 |
| rs719802 | 11 | 113234679 | T | C | 8.20E-03 | 1.41E-03 | 5.70E-09 | 3.91E-03 | 1.44E-03 | 6.50E-03 |
| rs1568488 | 3 | 153657951 | G | C | -1.10E-02 | 1.41E-03 | 4.40E-15 | -3.86E-03 | 1.44E-03 | 7.20E-03 |
| rs17716502 | 8 | 116659731 | C | T | 1.62E-02 | 1.71E-03 | 2.70E-21 | 4.71E-03 | 1.75E-03 | 7.20E-03 |
| rs57488047 | 15 | 79403002 | T | C | 9.86E-03 | 1.39E-03 | 1.10E-12 | 3.77E-03 | 1.41E-03 | 7.60E-03 |
| rs28839214 | 4 | 145313641 | G | T | -8.92E-03 | 1.41E-03 | 2.20E-10 | -3.83E-03 | 1.44E-03 | 7.60E-03 |
| rs2583410 | 4 | 102182199 | A | C | -1.34E-02 | 1.94E-03 | 5.30E-12 | -5.22E-03 | 1.98E-03 | 8.30E-03 |
| rs2343681 | 3 | 136535024 | G | A | -1.24E-02 | 1.68E-03 | 1.80E-13 | -4.51E-03 | 1.72E-03 | 8.60E-03 |
| rs12821683 | 12 | 58588964 | G | C | -1.12E-02 | 2.01E-03 | 2.80E-08 | -5.32E-03 | 2.06E-03 | 9.60E-03 |
| rs72917533 | 2 | 175238924 | T | C | 1.15E-02 | 1.76E-03 | 8.20E-11 | 4.66E-03 | 1.80E-03 | 9.70E-03 |
| rs76824303 | 3 | 62459819 | A | C | 1.52E-02 | 2.34E-03 | 9.20E-11 | 6.18E-03 | 2.40E-03 | 9.80E-03 |
| rs143662847 | 8 | 48804722 | C | T | 1.93E-02 | 3.53E-03 | 4.60E-08 | 9.32E-03 | 3.61E-03 | 9.90E-03 |
| rs1778830 | 1 | 156489974 | G | A | -9.91E-03 | 1.43E-03 | 3.80E-12 | -3.74E-03 | 1.46E-03 | 1.00E-02 |
| rs17770336 | 9 | 28414625 | C | T | -1.55E-02 | 1.46E-03 | 3.10E-26 | -3.84E-03 | 1.50E-03 | 1.00E-02 |
| rs61813324 | 1 | 156049877 | C | T | -1.83E-02 | 2.03E-03 | 1.80E-19 | -5.34E-03 | 2.07E-03 | 1.00E-02 |
| rs80082351 | 3 | 114415926 | A | G | 1.85E-02 | 2.85E-03 | 8.10E-11 | 7.49E-03 | 2.91E-03 | 1.00E-02 |
| rs7038966 | 9 | 73777777 | C | T | -9.48E-03 | 1.40E-03 | 1.40E-11 | -3.65E-03 | 1.43E-03 | 1.10E-02 |
| rs2247401 | 15 | 53156672 | G | A | -8.52E-03 | 1.56E-03 | 4.70E-08 | -4.08E-03 | 1.59E-03 | 1.10E-02 |
| rs329118 | 5 | 133861663 | C | T | 9.49E-03 | 1.39E-03 | 8.80E-12 | 3.53E-03 | 1.42E-03 | 1.30E-02 |
| rs7549358 | 1 | 115252609 | G | C | 8.83E-03 | 1.43E-03 | 6.50E-10 | 3.62E-03 | 1.46E-03 | 1.30E-02 |
| rs8124896 | 20 | 21385659 | T | C | -1.32E-02 | 2.28E-03 | 8.10E-09 | -5.77E-03 | 2.33E-03 | 1.30E-02 |
| rs2425856 | 20 | 44911954 | A | G | 8.42E-03 | 1.38E-03 | 1.10E-09 | 3.48E-03 | 1.41E-03 | 1.40E-02 |
| rs4792716 | 17 | 15943144 | A | G | -8.11E-03 | 1.38E-03 | 4.80E-09 | -3.47E-03 | 1.41E-03 | 1.40E-02 |
| rs9834519 | 3 | 156379637 | C | T | 1.56E-02 | 2.54E-03 | 8.40E-10 | 6.36E-03 | 2.60E-03 | 1.40E-02 |
| rs6752979 | 2 | 81741750 | G | A | -8.96E-03 | 1.47E-03 | 1.10E-09 | -3.67E-03 | 1.50E-03 | 1.50E-02 |
| rs1451533 | 2 | 105466005 | G | A | -1.10E-02 | 1.55E-03 | 1.30E-12 | -3.84E-03 | 1.58E-03 | 1.50E-02 |
| rs79686965 | 11 | 46020909 | A | G | -3.10E-02 | 5.56E-03 | 2.30E-08 | -1.38E-02 | 5.68E-03 | 1.50E-02 |
| rs61746970 | 19 | 51132746 | G | A | -2.13E-02 | 3.62E-03 | 3.80E-09 | -8.88E-03 | 3.69E-03 | 1.60E-02 |
| rs215634 | 7 | 32369148 | A | G | 1.04E-02 | 1.41E-03 | 1.40E-13 | 3.44E-03 | 1.44E-03 | 1.70E-02 |
| rs12031634 | 1 | 34584393 | G | A | 8.81E-03 | 1.50E-03 | 4.80E-09 | 3.60E-03 | 1.54E-03 | 1.90E-02 |
| rs2658797 | 11 | 93212254 | C | T | 7.79E-03 | 1.37E-03 | 1.30E-08 | 3.25E-03 | 1.40E-03 | 2.00E-02 |
| rs529200 | 3 | 173114305 | A | G | -1.03E-02 | 1.37E-03 | 5.80E-14 | -3.24E-03 | 1.40E-03 | 2.10E-02 |
| rs6583310 | 3 | 196170985 | G | C | -8.78E-03 | 1.38E-03 | 2.20E-10 | -3.26E-03 | 1.42E-03 | 2.10E-02 |
| rs61986330 | 14 | 73314450 | C | A | 8.73E-03 | 1.54E-03 | 1.40E-08 | 3.63E-03 | 1.57E-03 | 2.10E-02 |
| rs537508 | 4 | 171042158 | G | C | -7.77E-03 | 1.40E-03 | 2.60E-08 | -3.26E-03 | 1.43E-03 | 2.20E-02 |
| rs556992087 | 12 | 124500725 | T | C | -1.06E-02 | 1.67E-03 | 2.00E-10 | -3.91E-03 | 1.70E-03 | 2.20E-02 |
| rs73078357 | 3 | 48695834 | T | C | 1.19E-02 | 2.09E-03 | 1.30E-08 | 4.89E-03 | 2.14E-03 | 2.20E-02 |
| rs9673839 | 16 | 76895693 | A | G | -8.30E-03 | 1.38E-03 | 1.70E-09 | -3.20E-03 | 1.41E-03 | 2.30E-02 |
| rs788163 | 2 | 172931559 | A | C | -1.01E-02 | 1.54E-03 | 5.90E-11 | -3.57E-03 | 1.57E-03 | 2.30E-02 |
| rs62379271 | 5 | 105870033 | T | G | -7.81E-03 | 1.39E-03 | 2.00E-08 | -3.22E-03 | 1.42E-03 | 2.40E-02 |
| rs2725371 | 8 | 30854033 | A | G | 1.00E-02 | 1.50E-03 | 2.00E-11 | -3.46E-03 | 1.53E-03 | 2.40E-02 |
| rs6069625 | 20 | 54747469 | A | G | 8.73E-03 | 1.56E-03 | 2.00E-08 | 3.58E-03 | 1.59E-03 | 2.40E-02 |
| rs12213441 | 6 | 143208838 | C | T | -1.05E-02 | 1.67E-03 | 3.30E-10 | -3.85E-03 | 1.71E-03 | 2.40E-02 |
| rs76702514 | 1 | 195148296 | C | G | 1.01E-02 | 1.69E-03 | 2.00E-09 | 3.91E-03 | 1.73E-03 | 2.40E-02 |
| rs11218510 | 11 | 121922587 | G | A | 8.10E-03 | 1.40E-03 | 7.80E-09 | 3.22E-03 | 1.43E-03 | 2.50E-02 |
| rs1598121 | 3 | 82694710 | A | G | -8.96E-03 | 1.42E-03 | 3.00E-10 | -3.23E-03 | 1.46E-03 | 2.70E-02 |
| rs10457469 | 6 | 126083658 | G | A | -7.63E-03 | 1.37E-03 | 2.60E-08 | -3.08E-03 | 1.40E-03 | 2.80E-02 |
| rs9579775 | 13 | 20616557 | A | C | -1.46E-02 | 2.08E-03 | 2.10E-12 | -4.68E-03 | 2.13E-03 | 2.80E-02 |
| rs6511826 | 19 | 12706991 | G | A | 1.40E-02 | 2.46E-03 | 1.30E-08 | 5.51E-03 | 2.51E-03 | 2.80E-02 |
| rs7925100 | 11 | 118941596 | G | A | -9.17E-03 | 1.40E-03 | 6.30E-11 | -3.13E-03 | 1.43E-03 | 2.90E-02 |
| rs347551 | 5 | 119389031 | C | G | -8.89E-03 | 1.39E-03 | 1.80E-10 | -3.09E-03 | 1.43E-03 | 3.00E-02 |
| rs1899689 | 7 | 121964349 | C | T | -7.74E-03 | 1.41E-03 | 3.70E-08 | -3.10E-03 | 1.44E-03 | 3.10E-02 |
| rs11150462 | 16 | 82451679 | T | A | 8.44E-03 | 1.42E-03 | 3.10E-09 | 3.10E-03 | 1.46E-03 | 3.30E-02 |
| rs6597653 | 9 | 133788465 | G | C | -8.53E-03 | 1.41E-03 | 1.30E-09 | -3.05E-03 | 1.44E-03 | 3.40E-02 |
| rs115778101 | 1 | 78198554 | T | C | 1.78E-02 | 3.22E-03 | 3.40E-08 | 6.92E-03 | 3.29E-03 | 3.50E-02 |
| rs67913249 | 5 | 43204126 | C | G | 9.35E-03 | 1.45E-03 | 1.20E-10 | 3.07E-03 | 1.48E-03 | 3.80E-02 |
| rs7511698 | 1 | 25015638 | C | T | 8.21E-03 | 1.49E-03 | 3.20E-08 | 3.14E-03 | 1.52E-03 | 3.80E-02 |
| rs9852062 | 3 | 45373442 | T | A | 8.17E-03 | 1.38E-03 | 3.50E-09 | 2.89E-03 | 1.41E-03 | 4.10E-02 |
| rs2114210 | 8 | 95595162 | G | A | -9.86E-03 | 1.45E-03 | 1.20E-11 | -3.02E-03 | 1.49E-03 | 4.20E-02 |
| rs6030803 | 20 | 41986507 | T | C | 1.29E-02 | 2.07E-03 | 4.50E-10 | 4.30E-03 | 2.12E-03 | 4.20E-02 |
| rs1409158 | 1 | 119538890 | C | T | 9.23E-03 | 1.61E-03 | 1.00E-08 | 3.33E-03 | 1.65E-03 | 4.30E-02 |
| rs13174863 | 5 | 139080745 | A | G | -1.36E-02 | 1.94E-03 | 2.40E-12 | -4.03E-03 | 1.99E-03 | 4.30E-02 |
| rs10174253 | 2 | 181323160 | A | C | -1.12E-02 | 1.53E-03 | 2.50E-13 | -3.08E-03 | 1.57E-03 | 4.90E-02 |
| rs61754230 | 12 | 72179446 | C | T | -2.74E-02 | 4.93E-03 | 2.80E-08 | -9.81E-03 | 5.03E-03 | 5.10E-02 |
| rs7086898 | 10 | 104386152 | A | G | -1.39E-02 | 2.53E-03 | 4.30E-08 | -4.98E-03 | 2.59E-03 | 5.40E-02 |
| rs1631026 | 2 | 26953850 | C | T | -1.07E-02 | 1.37E-03 | 5.90E-15 | -2.69E-03 | 1.40E-03 | 5.50E-02 |
| rs1964926 | 21 | 42653121 | A | G | -8.23E-03 | 1.44E-03 | 1.10E-08 | -2.82E-03 | 1.47E-03 | 5.50E-02 |
| rs112852122 | 20 | 47498117 | G | A | 1.34E-02 | 1.91E-03 | 2.70E-12 | 3.75E-03 | 1.95E-03 | 5.50E-02 |
| rs6761463 | 2 | 50201547 | G | C | 1.26E-02 | 1.86E-03 | 1.30E-11 | 3.61E-03 | 1.90E-03 | 5.70E-02 |
| rs11047138 | 12 | 24019853 | C | G | -1.44E-02 | 2.63E-03 | 4.60E-08 | -5.08E-03 | 2.68E-03 | 5.80E-02 |
| rs16940823 | 18 | 22137319 | C | A | 1.08E-02 | 1.79E-03 | 1.60E-09 | 3.42E-03 | 1.82E-03 | 6.10E-02 |
| rs66674732 | 13 | 62721160 | G | A | -8.16E-03 | 1.41E-03 | 7.20E-09 | -2.68E-03 | 1.44E-03 | 6.30E-02 |
| rs1381010 | 4 | 112677085 | G | A | 8.24E-03 | 1.49E-03 | 3.00E-08 | 2.81E-03 | 1.52E-03 | 6.40E-02 |
| rs369461388 | 14 | 40104718 | G | C | -1.26E-02 | 2.01E-03 | 3.60E-10 | -3.80E-03 | 2.05E-03 | 6.40E-02 |
| rs1369159 | 15 | 66360842 | C | T | 7.88E-03 | 1.40E-03 | 1.70E-08 | 2.63E-03 | 1.43E-03 | 6.50E-02 |
| rs4989244 | 9 | 102100348 | G | A | 7.63E-03 | 1.39E-03 | 3.60E-08 | 2.61E-03 | 1.42E-03 | 6.60E-02 |
| rs12149660 | 16 | 70309237 | G | A | 1.62E-02 | 2.16E-03 | 6.20E-14 | 4.01E-03 | 2.21E-03 | 6.90E-02 |
| rs2035806 | 10 | 133984916 | G | A | 9.55E-03 | 1.39E-03 | 5.70E-12 | 2.56E-03 | 1.42E-03 | 7.00E-02 |
| rs429358 | 19 | 45411941 | T | C | 1.61E-02 | 1.90E-03 | 2.90E-17 | -3.45E-03 | 1.94E-03 | 7.50E-02 |
| rs7230240 | 18 | 42597978 | C | T | 9.40E-03 | 1.50E-03 | 4.20E-10 | 2.73E-03 | 1.54E-03 | 7.60E-02 |
| rs34542489 | 17 | 51917844 | A | C | 7.78E-03 | 1.40E-03 | 2.80E-08 | 2.52E-03 | 1.43E-03 | 7.80E-02 |
| rs201475383 | 20 | 26273991 | G | A | 2.40E-02 | 3.92E-03 | 8.80E-10 | 6.98E-03 | 4.00E-03 | 8.10E-02 |
| rs2056477 | 7 | 2079744 | G | C | 1.16E-02 | 1.64E-03 | 1.50E-12 | 2.90E-03 | 1.67E-03 | 8.30E-02 |
| rs6075658 | 20 | 2094078 | T | C | 8.49E-03 | 1.38E-03 | 6.80E-10 | 2.43E-03 | 1.40E-03 | 8.40E-02 |
| rs368540015 | 7 | 74292165 | A | G | -1.82E-02 | 3.25E-03 | 2.20E-08 | -5.73E-03 | 3.32E-03 | 8.40E-02 |
| rs142503704 | 5 | 92622421 | G | A | -2.63E-02 | 4.68E-03 | 1.90E-08 | -8.13E-03 | 4.78E-03 | 8.90E-02 |
| rs6050446 | 20 | 25195509 | A | G | -2.64E-02 | 3.89E-03 | 1.20E-11 | -6.70E-03 | 3.98E-03 | 9.20E-02 |
| rs1987960 | 20 | 30649834 | T | C | -1.89E-02 | 3.31E-03 | 1.10E-08 | -5.63E-03 | 3.38E-03 | 9.60E-02 |
| rs2660241 | 16 | 4940023 | T | C | -8.13E-03 | 1.43E-03 | 1.20E-08 | -2.41E-03 | 1.46E-03 | 9.90E-02 |
| rs3737992 | 1 | 33234128 | G | A | 1.37E-02 | 1.83E-03 | 5.30E-14 | 3.07E-03 | 1.87E-03 | 9.90E-02 |
| rs6823268 | 4 | 145982563 | A | G | -8.18E-03 | 1.42E-03 | 8.20E-09 | -2.36E-03 | 1.45E-03 | 1.00E-01 |
| rs75957461 | 19 | 11166163 | C | T | -1.77E-02 | 3.05E-03 | 6.60E-09 | -5.11E-03 | 3.11E-03 | 1.00E-01 |
| rs7182917 | 15 | 52080803 | T | C | 8.74E-03 | 1.39E-03 | 2.80E-10 | 2.25E-03 | 1.41E-03 | 1.10E-01 |
| rs9615723 | 22 | 48386670 | C | T | 7.71E-03 | 1.40E-03 | 3.80E-08 | 2.26E-03 | 1.43E-03 | 1.10E-01 |
| rs10960276 | 9 | 11819686 | C | A | 8.09E-03 | 1.43E-03 | 1.60E-08 | -2.34E-03 | 1.46E-03 | 1.10E-01 |
| rs4911382 | 20 | 32553095 | C | T | -8.32E-03 | 1.40E-03 | 2.60E-09 | -2.23E-03 | 1.43E-03 | 1.20E-01 |
| rs868784 | 11 | 43944388 | G | A | 7.87E-03 | 1.42E-03 | 2.70E-08 | 2.28E-03 | 1.45E-03 | 1.20E-01 |
| rs10823826 | 10 | 53649431 | C | T | -8.98E-03 | 1.59E-03 | 1.60E-08 | -2.56E-03 | 1.62E-03 | 1.20E-01 |
| rs12885251 | 14 | 99670791 | G | A | 7.63E-03 | 1.39E-03 | 4.10E-08 | 2.13E-03 | 1.42E-03 | 1.30E-01 |
| rs10505836 | 12 | 19288508 | A | C | -1.21E-02 | 1.99E-03 | 1.10E-09 | -3.08E-03 | 2.03E-03 | 1.30E-01 |
| rs77560793 | 1 | 175001179 | G | A | 2.46E-02 | 3.99E-03 | 7.80E-10 | 6.25E-03 | 4.08E-03 | 1.30E-01 |
| rs7570446 | 2 | 193801010 | C | A | -7.80E-03 | 1.37E-03 | 1.30E-08 | 2.06E-03 | 1.40E-03 | 1.40E-01 |
| rs12477088 | 2 | 67841326 | T | C | 1.05E-02 | 1.39E-03 | 4.70E-14 | 2.08E-03 | 1.42E-03 | 1.40E-01 |
| rs2542615 | 10 | 131128952 | C | T | 8.44E-03 | 1.46E-03 | 7.30E-09 | 2.21E-03 | 1.49E-03 | 1.40E-01 |
| rs1799507 | 12 | 16427314 | G | A | -1.08E-02 | 1.96E-03 | 3.10E-08 | -2.95E-03 | 2.00E-03 | 1.40E-01 |
| rs945211 | 1 | 32191798 | G | C | -8.49E-03 | 1.41E-03 | 1.60E-09 | -2.06E-03 | 1.44E-03 | 1.50E-01 |
| rs2516726 | 16 | 2095065 | T | C | 9.91E-03 | 1.64E-03 | 1.70E-09 | 2.42E-03 | 1.68E-03 | 1.50E-01 |
| rs72753485 | 9 | 96673230 | G | C | -1.56E-02 | 2.50E-03 | 4.30E-10 | -3.68E-03 | 2.55E-03 | 1.50E-01 |
| rs1373349 | 18 | 63282992 | C | T | 9.81E-03 | 1.48E-03 | 3.50E-11 | 2.13E-03 | 1.51E-03 | 1.60E-01 |
| rs79675564 | 2 | 211286896 | C | A | -1.51E-02 | 2.55E-03 | 3.40E-09 | -3.67E-03 | 2.61E-03 | 1.60E-01 |
| rs12927792 | 16 | 9713194 | C | T | -8.67E-03 | 1.40E-03 | 5.20E-10 | -1.97E-03 | 1.43E-03 | 1.70E-01 |
| rs7264802 | 20 | 62692440 | A | G | -9.81E-03 | 1.59E-03 | 7.20E-10 | -2.22E-03 | 1.62E-03 | 1.70E-01 |
| rs12477385 | 2 | 166144850 | G | T | 9.24E-03 | 1.64E-03 | 1.80E-08 | 2.28E-03 | 1.68E-03 | 1.70E-01 |
| rs10774018 | 12 | 2157925 | G | C | -9.48E-03 | 1.66E-03 | 1.10E-08 | -2.32E-03 | 1.69E-03 | 1.70E-01 |
| rs12705894 | 7 | 113351252 | G | A | 7.80E-03 | 1.38E-03 | 1.60E-08 | 1.90E-03 | 1.41E-03 | 1.80E-01 |
| rs187067151 | 20 | 29539588 | G | T | 2.67E-02 | 3.98E-03 | 2.00E-11 | 5.44E-03 | 4.06E-03 | 1.80E-01 |
| rs403694 | 21 | 46567625 | C | T | -1.22E-02 | 1.38E-03 | 1.30E-18 | -1.86E-03 | 1.41E-03 | 1.90E-01 |
| rs7893571 | 10 | 16750129 | G | T | -1.02E-02 | 1.46E-03 | 2.90E-12 | -1.93E-03 | 1.49E-03 | 1.90E-01 |
| rs36007635 | 6 | 163009335 | G | A | 1.37E-02 | 1.99E-03 | 5.60E-12 | 2.65E-03 | 2.03E-03 | 1.90E-01 |
| rs7548936 | 1 | 91207757 | G | C | -8.48E-03 | 1.42E-03 | 2.10E-09 | 1.85E-03 | 1.45E-03 | 2.00E-01 |
| rs151252883 | 2 | 228998026 | T | G | -9.22E-03 | 1.44E-03 | 1.60E-10 | -1.91E-03 | 1.47E-03 | 2.00E-01 |
| rs183315407 | 1 | 46201427 | G | A | 2.64E-02 | 3.49E-03 | 4.30E-14 | 4.54E-03 | 3.57E-03 | 2.00E-01 |
| rs2074686 | 7 | 100800635 | G | A | 8.28E-03 | 1.39E-03 | 2.90E-09 | 1.79E-03 | 1.42E-03 | 2.10E-01 |
| rs34373881 | 3 | 20432033 | G | A | 8.83E-03 | 1.54E-03 | 9.00E-09 | 1.98E-03 | 1.57E-03 | 2.10E-01 |
| rs12644329 | 4 | 143634746 | G | A | 7.91E-03 | 1.42E-03 | 2.50E-08 | 1.78E-03 | 1.45E-03 | 2.20E-01 |
| rs7518221 | 1 | 225561346 | T | C | 7.95E-03 | 1.43E-03 | 2.80E-08 | 1.80E-03 | 1.46E-03 | 2.20E-01 |
| rs4759228 | 12 | 56508409 | G | C | 1.16E-02 | 1.51E-03 | 1.30E-14 | 1.89E-03 | 1.54E-03 | 2.20E-01 |
| rs575840515 | 1 | 80796649 | A | G | -9.01E-03 | 1.52E-03 | 3.20E-09 | -1.90E-03 | 1.56E-03 | 2.20E-01 |
| rs10973159 | 9 | 36992547 | G | T | -8.05E-03 | 1.41E-03 | 1.20E-08 | 1.74E-03 | 1.44E-03 | 2.30E-01 |
| rs10760277 | 9 | 126093999 | C | T | -8.87E-03 | 1.41E-03 | 3.60E-10 | -1.68E-03 | 1.44E-03 | 2.40E-01 |
| rs61903695 | 11 | 89922417 | A | G | -1.13E-02 | 1.58E-03 | 6.60E-13 | -1.87E-03 | 1.61E-03 | 2.40E-01 |
| rs78517245 | 3 | 42587865 | T | C | -3.44E-02 | 5.85E-03 | 4.10E-09 | -6.98E-03 | 5.98E-03 | 2.40E-01 |
| rs10805383 | 5 | 63034606 | G | A | -1.05E-02 | 1.37E-03 | 2.60E-14 | -1.57E-03 | 1.40E-03 | 2.60E-01 |
| rs9522180 | 13 | 111970212 | C | T | 9.24E-03 | 1.38E-03 | 2.40E-11 | 1.55E-03 | 1.41E-03 | 2.70E-01 |
| rs1324110 | 6 | 93913200 | G | C | 7.77E-03 | 1.38E-03 | 2.00E-08 | 1.57E-03 | 1.41E-03 | 2.70E-01 |
| rs72910629 | 6 | 69761994 | A | G | -1.33E-02 | 2.01E-03 | 4.30E-11 | 2.26E-03 | 2.05E-03 | 2.70E-01 |
| rs78369934 | 17 | 61739101 | T | C | 1.99E-02 | 3.05E-03 | 6.00E-11 | -3.44E-03 | 3.11E-03 | 2.70E-01 |
| rs78886584 | 1 | 16859325 | A | G | -8.49E-03 | 1.38E-03 | 8.70E-10 | -1.52E-03 | 1.42E-03 | 2.80E-01 |
| rs409696 | 2 | 147900651 | G | A | 1.14E-02 | 1.39E-03 | 1.80E-16 | 1.55E-03 | 1.42E-03 | 2.80E-01 |
| rs12357890 | 10 | 99762693 | A | G | -1.22E-02 | 1.39E-03 | 1.60E-18 | -1.53E-03 | 1.42E-03 | 2.80E-01 |
| rs4806814 | 19 | 1860147 | G | A | 1.27E-02 | 1.90E-03 | 3.10E-11 | -2.10E-03 | 1.94E-03 | 2.80E-01 |
| rs78508049 | 1 | 210344884 | T | C | -1.11E-02 | 1.75E-03 | 2.10E-10 | -1.88E-03 | 1.79E-03 | 2.90E-01 |
| rs55931203 | 17 | 65854602 | C | T | -1.26E-02 | 1.78E-03 | 1.60E-12 | -1.92E-03 | 1.82E-03 | 2.90E-01 |
| rs149457 | 5 | 107438057 | C | T | 1.50E-02 | 1.83E-03 | 2.10E-16 | 1.98E-03 | 1.87E-03 | 2.90E-01 |
| rs11708540 | 3 | 70593081 | G | A | -1.06E-02 | 1.89E-03 | 2.00E-08 | 2.07E-03 | 1.93E-03 | 2.90E-01 |
| rs567230078 | 6 | 43588227 | T | A | 2.51E-02 | 4.25E-03 | 3.70E-09 | 4.57E-03 | 4.34E-03 | 2.90E-01 |
| rs13292699 | 9 | 15910044 | A | C | 1.25E-02 | 1.39E-03 | 1.50E-19 | -1.47E-03 | 1.42E-03 | 3.00E-01 |
| rs7913496 | 10 | 10257277 | C | T | 1.00E-02 | 1.79E-03 | 2.00E-08 | -1.88E-03 | 1.83E-03 | 3.00E-01 |
| rs76520838 | 15 | 47916618 | C | T | -2.32E-02 | 3.87E-03 | 1.90E-09 | -4.12E-03 | 3.95E-03 | 3.00E-01 |
| rs2396625 | 7 | 113028634 | T | A | 9.94E-03 | 1.40E-03 | 1.10E-12 | 1.45E-03 | 1.43E-03 | 3.10E-01 |
| rs3806114 | 6 | 20482335 | G | A | 8.79E-03 | 1.47E-03 | 2.30E-09 | 1.51E-03 | 1.50E-03 | 3.10E-01 |
| rs116195355 | 1 | 39941508 | C | A | 2.60E-02 | 3.96E-03 | 5.90E-11 | 4.09E-03 | 4.05E-03 | 3.10E-01 |
| rs698147 | 5 | 3513485 | A | G | 8.78E-03 | 1.38E-03 | 1.80E-10 | 1.41E-03 | 1.41E-03 | 3.20E-01 |
| rs1840660 | 7 | 114352615 | G | A | -9.95E-03 | 1.41E-03 | 2.00E-12 | 1.44E-03 | 1.45E-03 | 3.20E-01 |
| rs2866720 | 7 | 70106310 | C | T | -9.13E-03 | 1.42E-03 | 1.20E-10 | -1.46E-03 | 1.45E-03 | 3.20E-01 |
| rs2269610 | 6 | 33289935 | G | C | -1.28E-02 | 1.77E-03 | 5.10E-13 | 1.78E-03 | 1.81E-03 | 3.20E-01 |
| rs4962725 | 10 | 126733321 | T | C | -9.97E-03 | 1.39E-03 | 6.60E-13 | -1.39E-03 | 1.42E-03 | 3.30E-01 |
| rs2837398 | 21 | 41427168 | A | C | -8.20E-03 | 1.40E-03 | 4.80E-09 | 1.39E-03 | 1.43E-03 | 3.30E-01 |
| rs262956 | 3 | 183486117 | T | G | 9.12E-03 | 1.44E-03 | 2.20E-10 | 1.43E-03 | 1.47E-03 | 3.30E-01 |
| rs6530737 | 8 | 14095763 | A | G | 9.02E-03 | 1.44E-03 | 3.50E-10 | 1.40E-03 | 1.47E-03 | 3.40E-01 |
| rs11079849 | 17 | 47090785 | C | T | 1.22E-02 | 1.46E-03 | 6.40E-17 | 1.42E-03 | 1.49E-03 | 3.40E-01 |
| rs35957544 | 8 | 73440371 | G | T | 1.27E-02 | 1.39E-03 | 7.40E-20 | 1.33E-03 | 1.42E-03 | 3.50E-01 |
| rs28726372 | 1 | 84353839 | T | C | -9.08E-03 | 1.48E-03 | 9.40E-10 | -1.43E-03 | 1.52E-03 | 3.50E-01 |
| rs9421249 | 10 | 118623322 | C | T | -1.04E-02 | 1.56E-03 | 2.50E-11 | 1.49E-03 | 1.60E-03 | 3.50E-01 |
| rs61217499 | 12 | 108417780 | G | C | 1.28E-02 | 1.67E-03 | 2.30E-14 | 1.60E-03 | 1.71E-03 | 3.50E-01 |
| rs57654548 | 3 | 125196904 | C | A | 1.16E-02 | 2.12E-03 | 4.20E-08 | 2.02E-03 | 2.17E-03 | 3.50E-01 |
| rs11642387 | 16 | 6753239 | A | G | 1.29E-02 | 2.29E-03 | 2.10E-08 | -2.20E-03 | 2.34E-03 | 3.50E-01 |
| rs4836133 | 5 | 124332103 | C | A | -8.36E-03 | 1.41E-03 | 3.10E-09 | 2.31E-03 | 2.47E-03 | 3.50E-01 |
| rs2164300 | 4 | 67813017 | C | T | 7.55E-03 | 1.38E-03 | 4.20E-08 | -1.26E-03 | 1.41E-03 | 3.70E-01 |
| rs1454687 | 3 | 94038085 | C | G | 1.23E-02 | 1.37E-03 | 3.00E-19 | 1.24E-03 | 1.40E-03 | 3.80E-01 |
| rs114728753 | 3 | 78483402 | A | C | -8.95E-03 | 1.47E-03 | 1.00E-09 | -1.32E-03 | 1.50E-03 | 3.80E-01 |
| rs396354 | 2 | 86850022 | T | C | 1.04E-02 | 1.52E-03 | 8.00E-12 | 1.36E-03 | 1.55E-03 | 3.80E-01 |
| rs236660 | 7 | 75050086 | T | C | -1.45E-02 | 1.44E-03 | 9.10E-24 | -1.27E-03 | 1.47E-03 | 3.90E-01 |
| rs12427047 | 12 | 90213070 | C | T | 1.09E-02 | 1.60E-03 | 8.90E-12 | 1.40E-03 | 1.63E-03 | 3.90E-01 |
| rs1547205 | 9 | 98815145 | G | C | 1.32E-02 | 2.32E-03 | 1.30E-08 | 2.02E-03 | 2.37E-03 | 3.90E-01 |
| rs1458156 | 12 | 41887940 | C | T | -9.29E-03 | 1.37E-03 | 1.30E-11 | -1.19E-03 | 1.40E-03 | 4.00E-01 |
| rs1704190 | 2 | 200760629 | G | A | -8.33E-03 | 1.42E-03 | 4.50E-09 | -1.23E-03 | 1.45E-03 | 4.00E-01 |
| rs3753639 | 1 | 154986091 | T | C | -1.15E-02 | 1.60E-03 | 7.30E-13 | -1.38E-03 | 1.64E-03 | 4.00E-01 |
| rs7539903 | 1 | 209208033 | T | A | 7.69E-03 | 1.41E-03 | 4.60E-08 | 1.18E-03 | 1.44E-03 | 4.10E-01 |
| rs7321285 | 13 | 54319327 | A | C | 1.13E-02 | 1.72E-03 | 4.10E-11 | 1.46E-03 | 1.76E-03 | 4.10E-01 |
| rs1411432 | 9 | 16728532 | A | C | -1.24E-02 | 1.77E-03 | 2.00E-12 | 1.49E-03 | 1.81E-03 | 4.10E-01 |
| rs10756555 | 9 | 14459089 | G | A | 1.03E-02 | 1.39E-03 | 1.60E-13 | 1.13E-03 | 1.42E-03 | 4.20E-01 |
| rs4419475 | 4 | 96150044 | A | T | -8.10E-03 | 1.39E-03 | 6.50E-09 | 1.16E-03 | 1.43E-03 | 4.20E-01 |
| rs9888533 | 13 | 107854612 | C | T | -7.65E-03 | 1.40E-03 | 4.50E-08 | -1.15E-03 | 1.43E-03 | 4.20E-01 |
| rs7175642 | 15 | 59450079 | T | G | 8.59E-03 | 1.47E-03 | 5.20E-09 | 1.22E-03 | 1.50E-03 | 4.20E-01 |
| rs111584879 | 15 | 66678173 | T | C | 8.79E-03 | 1.61E-03 | 4.80E-08 | 1.33E-03 | 1.64E-03 | 4.20E-01 |
| rs12630209 | 3 | 156881392 | T | G | -8.75E-03 | 1.58E-03 | 3.00E-08 | -1.27E-03 | 1.61E-03 | 4.30E-01 |
| rs12376870 | 9 | 117890567 | G | A | 8.87E-03 | 1.62E-03 | 4.10E-08 | 1.28E-03 | 1.65E-03 | 4.40E-01 |
| rs11513729 | 12 | 112273499 | C | T | 7.99E-03 | 1.41E-03 | 1.50E-08 | 1.07E-03 | 1.44E-03 | 4.60E-01 |
| rs11134512 | 5 | 167847460 | T | G | 8.24E-03 | 1.47E-03 | 1.90E-08 | 1.12E-03 | 1.50E-03 | 4.60E-01 |
| rs7928320 | 11 | 116942753 | C | G | -1.73E-02 | 2.97E-03 | 6.10E-09 | -2.23E-03 | 3.04E-03 | 4.60E-01 |
| rs473837 | 8 | 60906881 | G | T | 8.63E-03 | 1.44E-03 | 1.80E-09 | -1.01E-03 | 1.47E-03 | 4.90E-01 |
| rs11765062 | 7 | 54417515 | T | C | 7.52E-03 | 1.37E-03 | 4.60E-08 | 9.37E-04 | 1.40E-03 | 5.00E-01 |
| rs12281009 | 11 | 117032959 | A | G | -1.79E-02 | 2.95E-03 | 1.30E-09 | -2.02E-03 | 3.01E-03 | 5.00E-01 |
| rs6950388 | 7 | 1270699 | G | A | -9.45E-03 | 1.70E-03 | 2.60E-08 | 1.15E-03 | 1.74E-03 | 5.10E-01 |
| rs12538826 | 7 | 99030228 | T | C | 1.68E-02 | 2.15E-03 | 7.20E-15 | -1.43E-03 | 2.20E-03 | 5.10E-01 |
| rs7433076 | 3 | 90234502 | T | A | 9.52E-03 | 1.38E-03 | 5.10E-12 | -9.17E-04 | 1.41E-03 | 5.20E-01 |
| rs1977658 | 1 | 107607037 | T | G | 8.10E-03 | 1.45E-03 | 2.50E-08 | -9.64E-04 | 1.49E-03 | 5.20E-01 |
| rs217672 | 14 | 62361021 | A | C | -1.18E-02 | 1.55E-03 | 2.30E-14 | 1.02E-03 | 1.58E-03 | 5.20E-01 |
| rs1040046 | 6 | 83473573 | C | A | -1.13E-02 | 1.92E-03 | 4.60E-09 | -1.25E-03 | 1.96E-03 | 5.20E-01 |
| rs35390852 | 4 | 143067054 | G | A | -1.14E-02 | 2.09E-03 | 4.80E-08 | -1.33E-03 | 2.13E-03 | 5.30E-01 |
| rs4663213 | 2 | 236807893 | G | A | 9.46E-03 | 1.66E-03 | 1.20E-08 | 1.04E-03 | 1.70E-03 | 5.40E-01 |
| rs73213484 | 4 | 28489339 | A | T | 1.46E-02 | 1.97E-03 | 1.10E-13 | 1.24E-03 | 2.01E-03 | 5.40E-01 |
| rs6507054 | 18 | 31248323 | T | C | -9.28E-03 | 1.39E-03 | 2.70E-11 | 8.60E-04 | 1.42E-03 | 5.50E-01 |
| rs9462670 | 6 | 41014309 | G | C | -9.42E-03 | 1.63E-03 | 7.10E-09 | -9.86E-04 | 1.66E-03 | 5.50E-01 |
| rs13275517 | 8 | 143364521 | T | C | -8.15E-03 | 1.39E-03 | 4.10E-09 | -8.14E-04 | 1.42E-03 | 5.70E-01 |
| rs11782074 | 8 | 142617096 | G | T | -9.72E-03 | 1.43E-03 | 1.20E-11 | 8.24E-04 | 1.46E-03 | 5.70E-01 |
| rs10160769 | 11 | 76474827 | G | C | 9.18E-03 | 1.68E-03 | 4.60E-08 | -9.87E-04 | 1.72E-03 | 5.70E-01 |
| rs113079574 | 4 | 147354089 | C | T | 9.65E-03 | 1.75E-03 | 3.30E-08 | -9.93E-04 | 1.78E-03 | 5.80E-01 |
| rs10935143 | 3 | 134665159 | G | A | 8.40E-03 | 1.38E-03 | 1.10E-09 | 7.63E-04 | 1.41E-03 | 5.90E-01 |
| rs1905616 | 8 | 93235675 | G | A | 8.20E-03 | 1.46E-03 | 1.80E-08 | 8.02E-04 | 1.49E-03 | 5.90E-01 |
| rs12072739 | 1 | 98315893 | A | G | -1.16E-02 | 1.64E-03 | 1.80E-12 | -8.95E-04 | 1.68E-03 | 5.90E-01 |
| rs12037905 | 1 | 219628036 | C | T | 7.93E-03 | 1.39E-03 | 1.10E-08 | 7.49E-04 | 1.42E-03 | 6.00E-01 |
| rs8134638 | 21 | 40644170 | T | C | -7.80E-03 | 1.42E-03 | 4.00E-08 | 7.68E-04 | 1.45E-03 | 6.00E-01 |
| rs12033257 | 1 | 112318484 | A | G | 9.80E-03 | 1.42E-03 | 4.90E-12 | -7.57E-04 | 1.45E-03 | 6.00E-01 |
| rs2237402 | 7 | 39449768 | G | A | 9.23E-03 | 1.45E-03 | 1.80E-10 | 7.69E-04 | 1.48E-03 | 6.00E-01 |
| rs61985411 | 14 | 41336102 | T | A | -1.65E-02 | 2.76E-03 | 2.10E-09 | -1.47E-03 | 2.82E-03 | 6.00E-01 |
| rs809955 | 4 | 140874760 | G | A | 1.01E-02 | 1.42E-03 | 1.40E-12 | -7.30E-04 | 1.46E-03 | 6.20E-01 |
| rs8192675 | 3 | 170724883 | T | C | -1.17E-02 | 1.51E-03 | 8.70E-15 | -7.69E-04 | 1.54E-03 | 6.20E-01 |
| rs7331420 | 13 | 99236471 | G | A | 9.14E-03 | 1.53E-03 | 2.10E-09 | -7.63E-04 | 1.56E-03 | 6.20E-01 |
| rs1017529 | 17 | 27912415 | C | A | -1.06E-02 | 1.84E-03 | 7.70E-09 | -9.30E-04 | 1.88E-03 | 6.20E-01 |
| rs2576135 | 13 | 54691442 | T | A | 1.32E-02 | 2.39E-03 | 3.50E-08 | 1.21E-03 | 2.44E-03 | 6.20E-01 |
| rs8011566 | 14 | 42939471 | T | A | -8.73E-03 | 1.39E-03 | 3.50E-10 | -6.89E-04 | 1.42E-03 | 6.30E-01 |
| rs39674 | 16 | 9413210 | C | G | -8.71E-03 | 1.50E-03 | 6.00E-09 | -7.29E-04 | 1.53E-03 | 6.30E-01 |
| rs2433733 | 2 | 230816703 | G | A | 1.05E-02 | 1.46E-03 | 7.30E-13 | 6.91E-04 | 1.50E-03 | 6.40E-01 |
| rs36131051 | 3 | 107888841 | T | G | 1.08E-02 | 1.71E-03 | 2.90E-10 | 8.28E-04 | 1.75E-03 | 6.40E-01 |
| rs112380819 | 3 | 9498519 | G | A | -1.37E-02 | 2.26E-03 | 1.30E-09 | -1.05E-03 | 2.31E-03 | 6.50E-01 |
| rs147730268 | 12 | 123024476 | G | T | 2.28E-02 | 2.49E-03 | 4.00E-20 | -1.15E-03 | 2.54E-03 | 6.50E-01 |
| rs62259692 | 3 | 51847709 | G | A | -1.78E-02 | 2.93E-03 | 1.40E-09 | 1.34E-03 | 3.00E-03 | 6.50E-01 |
| rs13186637 | 5 | 153108558 | T | C | 8.68E-03 | 1.44E-03 | 1.70E-09 | -6.46E-04 | 1.47E-03 | 6.60E-01 |
| rs9395520 | 6 | 13183523 | C | T | 9.76E-03 | 1.49E-03 | 5.90E-11 | 6.65E-04 | 1.52E-03 | 6.60E-01 |
| rs10927006 | 1 | 243557659 | T | C | 1.20E-02 | 1.95E-03 | 8.30E-10 | 8.84E-04 | 2.00E-03 | 6.60E-01 |
| rs10204994 | 2 | 35443726 | G | A | 1.01E-02 | 1.63E-03 | 5.30E-10 | 7.02E-04 | 1.66E-03 | 6.70E-01 |
| rs10499014 | 6 | 97947755 | C | G | 1.00E-02 | 1.56E-03 | 1.10E-10 | -6.65E-04 | 1.59E-03 | 6.80E-01 |
| rs533493779 | 2 | 104447054 | A | T | -8.69E-03 | 1.42E-03 | 1.10E-09 | -5.76E-04 | 1.46E-03 | 6.90E-01 |
| rs4660586 | 1 | 42407229 | C | T | 8.96E-03 | 1.56E-03 | 1.00E-08 | -6.28E-04 | 1.60E-03 | 6.90E-01 |
| rs7020196 | 9 | 12289527 | C | T | 7.79E-03 | 1.41E-03 | 3.60E-08 | 5.52E-04 | 1.44E-03 | 7.00E-01 |
| rs1865341 | 9 | 8845911 | C | T | -9.00E-03 | 1.62E-03 | 2.50E-08 | -6.28E-04 | 1.65E-03 | 7.00E-01 |
| rs12681792 | 8 | 62054463 | C | A | -9.56E-03 | 1.75E-03 | 4.30E-08 | -6.92E-04 | 1.78E-03 | 7.00E-01 |
| rs59227842 | 11 | 43692423 | A | G | -1.52E-02 | 1.49E-03 | 1.80E-24 | -5.69E-04 | 1.53E-03 | 7.10E-01 |
| rs13427822 | 2 | 213414265 | A | G | 9.45E-03 | 1.56E-03 | 1.30E-09 | -5.84E-04 | 1.59E-03 | 7.10E-01 |
| rs270689 | 6 | 104790532 | A | T | -1.01E-02 | 1.72E-03 | 4.20E-09 | -6.49E-04 | 1.76E-03 | 7.10E-01 |
| rs6478538 | 9 | 124627012 | A | G | 8.81E-03 | 1.47E-03 | 1.90E-09 | -5.27E-04 | 1.50E-03 | 7.30E-01 |
| rs2616143 | 8 | 20632022 | G | A | 8.85E-03 | 1.47E-03 | 1.90E-09 | 5.15E-04 | 1.51E-03 | 7.30E-01 |
| rs72976986 | 19 | 4050424 | G | A | 1.39E-02 | 1.77E-03 | 4.40E-15 | 6.31E-04 | 1.80E-03 | 7.30E-01 |
| rs9515446 | 13 | 112217108 | A | G | -9.69E-03 | 1.38E-03 | 2.20E-12 | -4.59E-04 | 1.41E-03 | 7.40E-01 |
| rs4759073 | 12 | 54653258 | G | A | 9.35E-03 | 1.39E-03 | 1.90E-11 | 4.35E-04 | 1.42E-03 | 7.60E-01 |
| rs10169594 | 2 | 41637688 | T | C | -7.88E-03 | 1.43E-03 | 3.30E-08 | -4.55E-04 | 1.46E-03 | 7.60E-01 |
| rs2744801 | 1 | 41155486 | C | T | 7.95E-03 | 1.45E-03 | 4.30E-08 | 4.44E-04 | 1.48E-03 | 7.60E-01 |
| rs149778057 | 13 | 31007805 | A | C | 9.05E-03 | 1.53E-03 | 3.80E-09 | 4.37E-04 | 1.57E-03 | 7.80E-01 |
| rs1320251 | 17 | 21264396 | C | T | 1.24E-02 | 1.38E-03 | 3.20E-19 | -3.69E-04 | 1.41E-03 | 7.90E-01 |
| rs7255223 | 19 | 32824310 | C | A | 8.97E-03 | 1.56E-03 | 8.50E-09 | 4.27E-04 | 1.59E-03 | 7.90E-01 |
| rs60497719 | 19 | 33971746 | G | A | -9.67E-03 | 1.58E-03 | 1.00E-09 | -4.30E-04 | 1.62E-03 | 7.90E-01 |
| rs4077093 | 12 | 51593616 | T | G | 9.69E-03 | 1.68E-03 | 7.90E-09 | 4.66E-04 | 1.72E-03 | 7.90E-01 |
| rs11691869 | 2 | 100805996 | C | A | 1.12E-02 | 1.43E-03 | 3.50E-15 | -3.65E-04 | 1.46E-03 | 8.00E-01 |
| rs10779835 | 1 | 230299949 | T | C | 8.01E-03 | 1.41E-03 | 1.20E-08 | 3.37E-04 | 1.44E-03 | 8.10E-01 |
| rs12971645 | 19 | 45807945 | G | A | 8.59E-03 | 1.54E-03 | 2.60E-08 | 3.43E-04 | 1.58E-03 | 8.30E-01 |
| rs2237025 | 4 | 55541879 | T | C | 1.02E-02 | 1.39E-03 | 1.80E-13 | -2.88E-04 | 1.42E-03 | 8.40E-01 |
| rs9529148 | 13 | 67419495 | G | A | -8.00E-03 | 1.42E-03 | 1.90E-08 | -2.79E-04 | 1.45E-03 | 8.50E-01 |
| rs3759584 | 14 | 103990799 | T | C | 9.74E-03 | 1.43E-03 | 1.10E-11 | 2.76E-04 | 1.46E-03 | 8.50E-01 |
| rs7601895 | 2 | 55281901 | C | G | 1.03E-02 | 1.49E-03 | 4.10E-12 | 2.78E-04 | 1.52E-03 | 8.50E-01 |
| rs7498044 | 15 | 92573639 | G | A | 9.95E-03 | 1.68E-03 | 3.20E-09 | -3.19E-04 | 1.72E-03 | 8.50E-01 |
| rs4916229 | 1 | 171443368 | C | G | -1.51E-02 | 2.33E-03 | 8.90E-11 | 3.77E-04 | 2.39E-03 | 8.70E-01 |
| rs35852935 | 4 | 17991522 | A | C | -2.19E-02 | 3.74E-03 | 4.90E-09 | 6.39E-04 | 3.82E-03 | 8.70E-01 |
| rs13104584 | 4 | 80811227 | G | A | -8.64E-03 | 1.40E-03 | 6.20E-10 | -1.93E-04 | 1.43E-03 | 8.90E-01 |
| rs4500770 | 16 | 74658430 | A | T | 7.82E-03 | 1.43E-03 | 4.30E-08 | 1.96E-04 | 1.46E-03 | 8.90E-01 |
| rs71495049 | 10 | 34014435 | G | A | -1.71E-02 | 2.48E-03 | 5.70E-12 | -3.46E-04 | 2.53E-03 | 8.90E-01 |
| rs1229984 | 4 | 100239319 | T | C | -2.30E-02 | 4.16E-03 | 3.20E-08 | 5.90E-04 | 4.26E-03 | 8.90E-01 |
| rs113706999 | 3 | 44159156 | T | A | -2.88E-02 | 4.73E-03 | 1.10E-09 | -6.03E-04 | 4.84E-03 | 9.00E-01 |
| rs58351927 | 17 | 5297038 | A | G | -1.01E-02 | 1.50E-03 | 1.20E-11 | -1.67E-04 | 1.53E-03 | 9.10E-01 |
| rs113569731 | 3 | 47093206 | C | A | -1.52E-02 | 2.41E-03 | 3.10E-10 | -2.93E-04 | 2.47E-03 | 9.10E-01 |
| rs61971082 | 13 | 86494667 | T | G | -1.01E-02 | 1.52E-03 | 3.00E-11 | 1.62E-04 | 1.56E-03 | 9.20E-01 |
| rs9477762 | 6 | 18507853 | A | T | -2.02E-02 | 3.05E-03 | 3.80E-11 | 2.72E-04 | 3.12E-03 | 9.30E-01 |
| rs10185199 | 2 | 40282202 | G | A | 1.00E-02 | 1.56E-03 | 1.60E-10 | 1.00E-04 | 1.60E-03 | 9.50E-01 |
| rs4658403 | 1 | 243832560 | C | T | 1.36E-02 | 1.84E-03 | 1.20E-13 | 9.35E-05 | 1.88E-03 | 9.60E-01 |
| rs80236973 | 3 | 188001014 | C | T | 1.22E-02 | 2.01E-03 | 1.40E-09 | 9.83E-05 | 2.05E-03 | 9.60E-01 |
| rs4425224 | 3 | 56249398 | C | A | 1.24E-02 | 2.24E-03 | 2.70E-08 | -1.19E-04 | 2.29E-03 | 9.60E-01 |
| rs181617194 | 12 | 122011598 | T | C | 2.27E-02 | 3.76E-03 | 1.70E-09 | 1.22E-04 | 3.84E-03 | 9.70E-01 |
| rs28408562 | 15 | 60917079 | C | G | -7.84E-03 | 1.38E-03 | 1.30E-08 | 4.07E-05 | 1.41E-03 | 9.80E-01 |
| rs7206608 | 16 | 82872628 | C | G | -9.79E-03 | 1.47E-03 | 2.60E-11 | 4.55E-05 | 1.50E-03 | 9.80E-01 |
| rs12253527 | 10 | 21819824 | G | A | -1.36E-02 | 1.47E-03 | 2.00E-20 | -9.61E-06 | 1.50E-03 | 9.90E-01 |

**Table S2C.** The genetic variants strongly associated with childhood adiposity at genome wide significance and not adulthood adiposity (exclude adult SNPs at P ≤ 5×10^-8^).

| SNP | Chromosome | Base position | Effect allele | Other allele | Beta (Adult) | SE (Adult) | P (Adult) | Beta (Age 10) | SE (Age 10) | P (Age 10) |
| --- | --- | --- | --- | --- | --- | --- | --- | --- | --- | --- |
| rs115319174 | 2 | 207066474 | G | C | -1.13E-02 | 2.97E-03 | 1.40E-04 | -4.23E-02 | 3.04E-03 | 3.30E-44 |
| rs4744246 | 9 | 96254464 | A | G | 9.44E-04 | 1.45E-03 | 5.10E-01 | -1.58E-02 | 1.48E-03 | 1.60E-26 |
| rs34260097 | 6 | 100727703 | T | G | -2.36E-03 | 1.64E-03 | 1.50E-01 | -1.78E-02 | 1.68E-03 | 2.50E-26 |
| rs12110721 | 6 | 55190480 | G | A | -7.88E-03 | 1.85E-03 | 2.10E-05 | -1.89E-02 | 1.89E-03 | 1.50E-23 |
| rs1933437 | 13 | 28624294 | G | A | 7.25E-03 | 1.42E-03 | 3.10E-07 | 1.42E-02 | 1.45E-03 | 1.20E-22 |
| rs9265968 | 6 | 31315663 | A | T | -7.51E-03 | 2.06E-03 | 2.80E-04 | -2.01E-02 | 2.11E-03 | 1.40E-21 |
| rs7239114 | 18 | 45921214 | G | A | -6.44E-03 | 1.39E-03 | 3.40E-06 | -1.35E-02 | 1.42E-03 | 1.70E-21 |
| rs75001243 | 7 | 93216002 | C | T | -6.80E-03 | 1.43E-03 | 1.90E-06 | -1.37E-02 | 1.46E-03 | 5.10E-21 |
| rs61978655 | 14 | 30491807 | G | A | -1.77E-02 | 3.57E-03 | 7.30E-07 | -3.39E-02 | 3.65E-03 | 1.40E-20 |
| rs16996644 | 20 | 15813475 | C | G | -1.06E-02 | 2.07E-03 | 2.90E-07 | -1.93E-02 | 2.11E-03 | 4.80E-20 |
| rs7084503 | 10 | 2666859 | T | C | 4.87E-03 | 1.38E-03 | 4.10E-04 | 1.29E-02 | 1.41E-03 | 5.20E-20 |
| rs9291816 | 5 | 63932508 | C | T | 7.98E-03 | 1.47E-03 | 5.30E-08 | 1.34E-02 | 1.50E-03 | 4.20E-19 |
| rs7958241 | 12 | 49509262 | A | G | -4.99E-03 | 1.45E-03 | 5.50E-04 | -1.31E-02 | 1.48E-03 | 8.60E-19 |
| rs35162296 | 6 | 26318262 | C | T | -1.14E-02 | 2.22E-03 | 2.80E-07 | -2.01E-02 | 2.27E-03 | 1.00E-18 |
| rs2767486 | 1 | 65991203 | A | G | -1.36E-03 | 1.70E-03 | 4.20E-01 | -1.54E-02 | 1.74E-03 | 1.20E-18 |
| rs1384660 | 2 | 142299735 | G | A | 7.46E-03 | 1.76E-03 | 2.30E-05 | 1.58E-02 | 1.80E-03 | 1.80E-18 |
| rs3131336 | 6 | 28831611 | C | T | -8.18E-03 | 2.13E-03 | 1.20E-04 | -1.91E-02 | 2.18E-03 | 2.00E-18 |
| rs34196306 | 6 | 27425644 | G | C | -9.19E-03 | 2.24E-03 | 4.00E-05 | -2.00E-02 | 2.29E-03 | 2.30E-18 |
| rs3129942 | 6 | 32338283 | G | T | -8.01E-03 | 1.56E-03 | 3.10E-07 | -1.39E-02 | 1.60E-03 | 2.70E-18 |
| rs13254613 | 8 | 64804804 | A | C | 5.65E-03 | 1.44E-03 | 9.10E-05 | -1.26E-02 | 1.48E-03 | 1.60E-17 |
| rs796915 | 6 | 154304628 | C | G | -7.37E-03 | 1.49E-03 | 7.70E-07 | -1.29E-02 | 1.52E-03 | 2.30E-17 |
| rs2594994 | 3 | 11339960 | T | A | 2.11E-03 | 1.79E-03 | 2.40E-01 | 1.54E-02 | 1.83E-03 | 3.10E-17 |
| rs1333010 | 13 | 66205228 | G | A | 6.53E-03 | 1.41E-03 | 3.50E-06 | 1.21E-02 | 1.44E-03 | 4.80E-17 |
| rs78444298 | 1 | 184672098 | G | A | -1.67E-02 | 4.99E-03 | 8.20E-04 | 4.20E-02 | 5.10E-03 | 1.60E-16 |
| rs117903946 | 16 | 67449639 | G | A | -9.78E-03 | 3.87E-03 | 1.10E-02 | -3.23E-02 | 3.95E-03 | 3.20E-16 |
| rs11215403 | 11 | 115058585 | G | A | 5.91E-03 | 1.60E-03 | 2.20E-04 | 1.32E-02 | 1.63E-03 | 5.50E-16 |
| rs2207894 | 20 | 54387343 | C | T | 7.60E-03 | 1.75E-03 | 1.30E-05 | 1.44E-02 | 1.78E-03 | 7.70E-16 |
| rs11642090 | 16 | 81730582 | T | C | -7.28E-03 | 1.43E-03 | 3.40E-07 | -1.17E-02 | 1.46E-03 | 1.00E-15 |
| rs788858 | 4 | 82138300 | A | G | 5.76E-04 | 1.51E-03 | 7.00E-01 | 1.23E-02 | 1.54E-03 | 1.70E-15 |
| rs72755233 | 15 | 100692953 | G | A | -9.68E-03 | 2.18E-03 | 8.80E-06 | -1.76E-02 | 2.22E-03 | 2.30E-15 |
| rs818898 | 9 | 6970806 | A | G | 6.19E-03 | 1.49E-03 | 3.30E-05 | 1.21E-02 | 1.52E-03 | 2.30E-15 |
| rs12713889 | 2 | 77225361 | T | C | 5.70E-03 | 1.45E-03 | 8.70E-05 | 1.17E-02 | 1.49E-03 | 3.80E-15 |
| rs12450028 | 17 | 2207425 | C | T | 6.86E-03 | 1.44E-03 | 2.00E-06 | 1.16E-02 | 1.47E-03 | 4.20E-15 |
| rs2722406 | 7 | 24306762 | C | T | -4.31E-03 | 1.52E-03 | 4.60E-03 | -1.22E-02 | 1.55E-03 | 4.40E-15 |
| rs2187642 | 12 | 11855624 | A | C | -1.58E-03 | 1.41E-03 | 2.60E-01 | -1.12E-02 | 1.44E-03 | 1.00E-14 |
| rs6449532 | 5 | 60715446 | C | T | 6.98E-03 | 1.43E-03 | 9.90E-07 | 1.11E-02 | 1.46E-03 | 3.30E-14 |
| rs10896348 | 11 | 68357368 | T | C | 7.20E-03 | 1.53E-03 | 2.60E-06 | 1.19E-02 | 1.56E-03 | 3.30E-14 |
| rs1013737 | 18 | 937050 | G | C | -5.85E-03 | 1.37E-03 | 2.10E-05 | -1.06E-02 | 1.40E-03 | 3.40E-14 |
| rs1199333 | 3 | 138091701 | G | T | 9.32E-03 | 1.76E-03 | 1.20E-07 | 1.35E-02 | 1.80E-03 | 5.90E-14 |
| rs2034963 | 3 | 48170802 | G | C | 5.00E-03 | 1.45E-03 | 5.40E-04 | 1.11E-02 | 1.48E-03 | 6.70E-14 |
| rs1342831 | 6 | 54096151 | T | C | -6.59E-03 | 2.95E-03 | 2.60E-02 | -2.25E-02 | 3.02E-03 | 9.00E-14 |
| rs7989098 | 13 | 27925496 | T | C | -7.42E-03 | 1.59E-03 | 3.10E-06 | -1.21E-02 | 1.63E-03 | 1.00E-13 |
| rs117911387 | 9 | 130446836 | G | A | -3.68E-03 | 3.26E-03 | 2.60E-01 | -2.46E-02 | 3.33E-03 | 1.60E-13 |
| rs2229330 | 1 | 6649228 | T | G | -3.47E-03 | 2.64E-03 | 1.90E-01 | -1.97E-02 | 2.69E-03 | 2.30E-13 |
| rs9438393 | 1 | 205782718 | A | G | 5.27E-03 | 1.39E-03 | 1.50E-04 | 1.03E-02 | 1.42E-03 | 3.40E-13 |
| rs200801362 | 6 | 31555480 | T | C | -6.08E-03 | 2.54E-03 | 1.70E-02 | -1.88E-02 | 2.60E-03 | 3.80E-13 |
| rs2275241 | 9 | 129370576 | G | A | -4.11E-03 | 1.42E-03 | 3.70E-03 | -1.04E-02 | 1.45E-03 | 7.50E-13 |
| rs12214497 | 6 | 10015908 | G | T | 5.63E-03 | 1.44E-03 | 9.30E-05 | 1.05E-02 | 1.47E-03 | 9.40E-13 |
| rs7306710 | 12 | 66376091 | T | C | 7.35E-04 | 1.38E-03 | 5.90E-01 | 9.97E-03 | 1.41E-03 | 1.60E-12 |
| rs78607331 | 12 | 57648644 | C | T | -1.80E-02 | 3.31E-03 | 6.10E-08 | -2.39E-02 | 3.38E-03 | 1.70E-12 |
| rs77960 | 5 | 103964585 | G | A | -6.24E-03 | 1.46E-03 | 1.90E-05 | 1.05E-02 | 1.49E-03 | 1.70E-12 |
| rs7606059 | 2 | 188152749 | T | C | -4.05E-03 | 1.46E-03 | 5.70E-03 | -1.05E-02 | 1.50E-03 | 2.10E-12 |
| rs7305424 | 12 | 118399491 | A | T | -3.88E-03 | 1.45E-03 | 7.60E-03 | -1.04E-02 | 1.48E-03 | 2.40E-12 |
| rs7869098 | 9 | 27816218 | T | G | 6.36E-03 | 1.38E-03 | 3.80E-06 | 9.84E-03 | 1.41E-03 | 2.60E-12 |
| rs61937656 | 12 | 39483502 | G | A | 5.07E-03 | 1.64E-03 | 2.00E-03 | 1.17E-02 | 1.68E-03 | 2.80E-12 |
| rs73422097 | 6 | 41727740 | A | G | -5.87E-03 | 1.49E-03 | 8.30E-05 | -1.06E-02 | 1.52E-03 | 3.30E-12 |
| rs7931626 | 11 | 28421841 | C | T | 5.27E-03 | 1.38E-03 | 1.30E-04 | 9.70E-03 | 1.41E-03 | 5.00E-12 |
| rs1452991 | 6 | 141473363 | G | A | -5.77E-03 | 1.43E-03 | 5.30E-05 | -1.01E-02 | 1.46E-03 | 5.30E-12 |
| rs10790809 | 11 | 126372550 | A | G | -6.57E-03 | 1.38E-03 | 2.00E-06 | -9.67E-03 | 1.41E-03 | 7.00E-12 |
| rs7012648 | 8 | 28091482 | G | A | -6.85E-03 | 1.40E-03 | 9.30E-07 | -9.74E-03 | 1.43E-03 | 8.40E-12 |
| rs947088 | 20 | 17171373 | G | T | -7.27E-03 | 1.53E-03 | 2.00E-06 | -1.06E-02 | 1.56E-03 | 1.00E-11 |
| rs3817428 | 15 | 89415247 | C | G | -3.19E-03 | 1.56E-03 | 4.10E-02 | -1.08E-02 | 1.59E-03 | 1.30E-11 |
| rs836179 | 12 | 50503082 | A | G | 2.37E-03 | 1.42E-03 | 9.50E-02 | 9.81E-03 | 1.45E-03 | 1.40E-11 |
| rs62425398 | 6 | 166416028 | C | A | -9.12E-03 | 2.24E-03 | 4.50E-05 | -1.54E-02 | 2.28E-03 | 1.50E-11 |
| rs11205303 | 1 | 149906413 | T | C | 1.24E-03 | 1.39E-03 | 3.80E-01 | 9.58E-03 | 1.43E-03 | 1.80E-11 |
| rs139497 | 22 | 41640098 | C | T | 6.20E-03 | 1.49E-03 | 3.30E-05 | 1.02E-02 | 1.52E-03 | 2.00E-11 |
| rs601338 | 19 | 49206674 | G | A | 9.17E-04 | 1.37E-03 | 5.00E-01 | 9.38E-03 | 1.40E-03 | 2.10E-11 |
| rs6979832 | 7 | 127856276 | A | G | -3.30E-03 | 1.38E-03 | 1.70E-02 | -9.42E-03 | 1.41E-03 | 2.30E-11 |
| rs661878 | 11 | 29188691 | A | G | 8.60E-03 | 2.02E-03 | 2.00E-05 | 1.38E-02 | 2.06E-03 | 2.50E-11 |
| rs112898427 | 2 | 67561335 | C | T | 1.04E-03 | 1.52E-03 | 4.90E-01 | 1.03E-02 | 1.55E-03 | 2.50E-11 |
| rs7808296 | 7 | 103127620 | C | T | -2.55E-03 | 1.48E-03 | 8.50E-02 | -1.00E-02 | 1.51E-03 | 2.80E-11 |
| rs146910503 | 2 | 25446473 | G | A | 1.48E-02 | 4.91E-03 | 2.60E-03 | 3.34E-02 | 5.03E-03 | 2.90E-11 |
| rs7759938 | 6 | 105378954 | C | T | -3.05E-03 | 1.47E-03 | 3.80E-02 | -9.98E-03 | 1.50E-03 | 2.90E-11 |
| rs824207 | 15 | 24007729 | A | G | -2.21E-03 | 1.38E-03 | 1.10E-01 | -9.31E-03 | 1.40E-03 | 3.30E-11 |
| rs1422067 | 5 | 77424836 | C | T | 8.70E-03 | 1.61E-03 | 6.50E-08 | 1.09E-02 | 1.65E-03 | 3.80E-11 |
| rs7162542 | 15 | 84514290 | C | G | 3.15E-03 | 1.38E-03 | 2.30E-02 | 9.31E-03 | 1.41E-03 | 4.00E-11 |
| rs2970356 | 15 | 90623540 | C | G | -3.62E-03 | 1.55E-03 | 2.00E-02 | -1.05E-02 | 1.58E-03 | 4.10E-11 |
| rs4074404 | 1 | 187683956 | T | A | -4.84E-03 | 1.88E-03 | 1.00E-02 | -1.27E-02 | 1.92E-03 | 4.30E-11 |
| rs12045879 | 1 | 15817090 | C | T | 6.18E-03 | 1.47E-03 | 2.70E-05 | 9.89E-03 | 1.51E-03 | 5.30E-11 |
| rs7020564 | 9 | 109670016 | A | T | 4.97E-03 | 1.52E-03 | 1.10E-03 | 1.00E-02 | 1.55E-03 | 1.10E-10 |
| rs10095724 | 8 | 53739232 | G | A | 2.96E-03 | 1.43E-03 | 3.90E-02 | 9.37E-03 | 1.46E-03 | 1.40E-10 |
| rs7536458 | 1 | 118864602 | T | G | -7.40E-03 | 1.55E-03 | 1.90E-06 | -1.01E-02 | 1.59E-03 | 1.90E-10 |
| rs8096658 | 18 | 77156537 | C | G | 4.29E-03 | 1.39E-03 | 2.00E-03 | 9.02E-03 | 1.42E-03 | 2.00E-10 |
| rs4958568 | 5 | 152016093 | G | A | 4.81E-03 | 1.53E-03 | 1.70E-03 | 9.96E-03 | 1.57E-03 | 2.10E-10 |
| rs7424771 | 2 | 161276378 | G | A | -2.31E-03 | 1.38E-03 | 9.40E-02 | 8.93E-03 | 1.41E-03 | 2.20E-10 |
| rs4572029 | 10 | 70889053 | A | G | -7.22E-04 | 1.71E-03 | 6.70E-01 | 1.11E-02 | 1.75E-03 | 2.20E-10 |
| rs2246623 | 17 | 74084449 | C | T | 4.79E-03 | 1.38E-03 | 5.20E-04 | 8.94E-03 | 1.41E-03 | 2.30E-10 |
| rs2141004 | 2 | 6194359 | A | C | 7.39E-03 | 1.55E-03 | 2.00E-06 | 1.00E-02 | 1.59E-03 | 2.50E-10 |
| rs10133279 | 14 | 82702712 | C | T | -4.83E-03 | 1.39E-03 | 5.20E-04 | -8.96E-03 | 1.42E-03 | 2.80E-10 |
| rs77976727 | 8 | 4300554 | C | T | -7.44E-03 | 2.36E-03 | 1.60E-03 | -1.51E-02 | 2.41E-03 | 3.60E-10 |
| rs2629881 | 3 | 59778271 | C | T | -2.64E-03 | 1.65E-03 | 1.10E-01 | -1.06E-02 | 1.69E-03 | 3.80E-10 |
| rs78907487 | 22 | 22151939 | A | C | -6.77E-03 | 1.94E-03 | 4.70E-04 | -1.24E-02 | 1.98E-03 | 3.90E-10 |
| rs10503246 | 8 | 4130363 | A | G | -3.08E-03 | 1.52E-03 | 4.20E-02 | -9.66E-03 | 1.55E-03 | 4.60E-10 |
| rs7753558 | 6 | 117523471 | C | A | 5.70E-03 | 1.43E-03 | 6.80E-05 | 9.09E-03 | 1.46E-03 | 5.20E-10 |
| rs7951870 | 11 | 46373311 | T | C | -7.15E-03 | 1.82E-03 | 8.70E-05 | -1.16E-02 | 1.86E-03 | 5.40E-10 |
| rs62621197 | 19 | 8670147 | C | T | -6.50E-03 | 3.77E-03 | 8.50E-02 | -2.35E-02 | 3.85E-03 | 9.60E-10 |
| rs212517 | 1 | 21577159 | T | A | 1.56E-03 | 1.40E-03 | 2.60E-01 | 8.75E-03 | 1.43E-03 | 9.60E-10 |
| rs62134189 | 2 | 45046339 | A | G | -1.82E-03 | 2.27E-03 | 4.20E-01 | 1.42E-02 | 2.32E-03 | 1.00E-09 |
| rs117455294 | 20 | 57427951 | C | A | 4.90E-03 | 3.11E-03 | 1.10E-01 | 1.93E-02 | 3.17E-03 | 1.10E-09 |
| rs10234366 | 7 | 46743746 | G | A | -1.75E-03 | 2.24E-03 | 4.40E-01 | -1.40E-02 | 2.29E-03 | 1.10E-09 |
| rs3936511 | 5 | 55860781 | A | G | 2.42E-04 | 1.74E-03 | 8.90E-01 | 1.08E-02 | 1.78E-03 | 1.10E-09 |
| rs2281148 | 20 | 36433288 | T | C | -5.55E-03 | 1.59E-03 | 4.70E-04 | -9.79E-03 | 1.62E-03 | 1.50E-09 |
| rs75387636 | 10 | 120278394 | G | A | -7.46E-03 | 3.44E-03 | 3.00E-02 | -2.12E-02 | 3.51E-03 | 1.50E-09 |
| rs10860295 | 12 | 98542699 | T | C | -1.81E-03 | 1.38E-03 | 1.90E-01 | -8.52E-03 | 1.41E-03 | 1.60E-09 |
| rs10498713 | 6 | 22729300 | G | T | -1.36E-03 | 1.92E-03 | 4.80E-01 | -1.18E-02 | 1.97E-03 | 2.00E-09 |
| rs2999158 | 1 | 113239478 | T | C | -3.26E-03 | 1.45E-03 | 2.50E-02 | 8.87E-03 | 1.48E-03 | 2.20E-09 |
| rs7814267 | 8 | 5545084 | A | G | -5.53E-03 | 1.79E-03 | 2.00E-03 | -1.09E-02 | 1.83E-03 | 2.50E-09 |
| rs8117463 | 20 | 17231063 | G | A | 5.19E-03 | 1.47E-03 | 4.10E-04 | 8.90E-03 | 1.50E-03 | 2.90E-09 |
| rs146980124 | 17 | 44627649 | A | C | 5.48E-03 | 1.69E-03 | 1.20E-03 | 1.02E-02 | 1.72E-03 | 2.90E-09 |
| rs10953577 | 7 | 108263540 | T | C | -2.48E-03 | 1.42E-03 | 8.00E-02 | -8.58E-03 | 1.45E-03 | 3.00E-09 |
| rs12748436 | 1 | 177761109 | C | G | -5.65E-03 | 2.58E-03 | 2.90E-02 | -1.56E-02 | 2.64E-03 | 3.50E-09 |
| rs4723263 | 7 | 33194826 | G | C | -2.54E-03 | 1.38E-03 | 6.60E-02 | -8.33E-03 | 1.41E-03 | 3.70E-09 |
| rs7123283 | 11 | 122809055 | C | T | 2.53E-03 | 1.38E-03 | 6.70E-02 | 8.32E-03 | 1.41E-03 | 3.70E-09 |
| rs67603370 | 17 | 7524504 | G | A | -4.59E-04 | 2.63E-03 | 8.60E-01 | -1.58E-02 | 2.68E-03 | 3.70E-09 |
| rs11891707 | 2 | 207120604 | T | C | 4.36E-04 | 2.01E-03 | 8.30E-01 | 1.21E-02 | 2.05E-03 | 3.80E-09 |
| rs9652090 | 13 | 27983367 | G | T | -3.71E-03 | 1.39E-03 | 7.40E-03 | -8.33E-03 | 1.42E-03 | 4.00E-09 |
| rs10842356 | 12 | 24621348 | A | T | 5.19E-03 | 1.37E-03 | 1.60E-04 | 8.23E-03 | 1.40E-03 | 4.10E-09 |
| rs62032001 | 16 | 49065630 | A | C | -1.94E-03 | 1.73E-03 | 2.60E-01 | -1.04E-02 | 1.77E-03 | 4.20E-09 |
| rs10116891 | 9 | 122651993 | G | A | -9.94E-03 | 2.29E-03 | 1.40E-05 | -1.36E-02 | 2.34E-03 | 5.80E-09 |
| rs884152 | 8 | 25770557 | G | T | -3.32E-03 | 1.43E-03 | 2.00E-02 | -8.51E-03 | 1.46E-03 | 6.00E-09 |
| rs3118252 | 9 | 25115154 | G | C | -1.47E-03 | 1.40E-03 | 2.90E-01 | -8.32E-03 | 1.43E-03 | 6.00E-09 |
| rs61936936 | 12 | 116391685 | A | T | -4.86E-03 | 2.30E-03 | 3.40E-02 | -1.36E-02 | 2.34E-03 | 6.20E-09 |
| rs9594686 | 13 | 42723197 | C | T | 6.04E-03 | 1.80E-03 | 8.10E-04 | 1.07E-02 | 1.84E-03 | 6.40E-09 |
| rs3181269 | 11 | 33755956 | C | T | 3.49E-03 | 1.57E-03 | 2.60E-02 | 9.33E-03 | 1.61E-03 | 6.40E-09 |
| rs9370527 | 6 | 56245812 | G | A | -3.28E-03 | 1.60E-03 | 4.10E-02 | -9.49E-03 | 1.64E-03 | 6.40E-09 |
| rs686431 | 6 | 35974217 | C | T | -1.48E-02 | 5.09E-03 | 3.70E-03 | -3.02E-02 | 5.20E-03 | 6.50E-09 |
| rs74080008 | 1 | 51047717 | G | T | 5.59E-03 | 1.52E-03 | 2.40E-04 | 9.01E-03 | 1.56E-03 | 6.80E-09 |
| rs7354849 | 1 | 232765308 | A | G | -4.26E-03 | 1.38E-03 | 2.00E-03 | -8.17E-03 | 1.41E-03 | 6.80E-09 |
| rs7672 | 16 | 68294800 | C | G | 4.69E-03 | 1.53E-03 | 2.20E-03 | 9.00E-03 | 1.56E-03 | 7.90E-09 |
| rs7503580 | 17 | 79087036 | C | T | -4.88E-03 | 1.89E-03 | 1.00E-02 | -1.11E-02 | 1.93E-03 | 8.90E-09 |
| rs115359679 | 7 | 755987 | C | A | -5.38E-03 | 2.80E-03 | 5.40E-02 | -1.64E-02 | 2.86E-03 | 9.20E-09 |
| rs7711823 | 5 | 158489315 | A | G | 6.57E-03 | 1.43E-03 | 4.30E-06 | 8.39E-03 | 1.46E-03 | 9.30E-09 |
| rs7565437 | 2 | 65646966 | T | C | 2.01E-03 | 1.39E-03 | 1.50E-01 | 8.18E-03 | 1.43E-03 | 9.50E-09 |
| rs11256627 | 10 | 10535954 | G | A | -9.45E-04 | 1.51E-03 | 5.30E-01 | -8.88E-03 | 1.55E-03 | 9.50E-09 |
| rs12941038 | 17 | 66509143 | C | T | -4.75E-03 | 1.63E-03 | 3.70E-03 | -9.54E-03 | 1.67E-03 | 1.10E-08 |
| rs4545941 | 19 | 16534207 | T | C | -2.12E-03 | 1.86E-03 | 2.60E-01 | -1.09E-02 | 1.90E-03 | 1.10E-08 |
| rs4783789 | 16 | 51446707 | T | C | 1.65E-03 | 1.65E-03 | 3.20E-01 | 9.58E-03 | 1.68E-03 | 1.20E-08 |
| rs12308065 | 12 | 120624085 | A | G | -6.54E-04 | 1.42E-03 | 6.50E-01 | -8.29E-03 | 1.45E-03 | 1.20E-08 |
| rs201666051 | 9 | 20920868 | C | T | -1.49E-03 | 1.42E-03 | 2.90E-01 | -8.24E-03 | 1.45E-03 | 1.30E-08 |
| rs1476698 | 2 | 242296449 | A | G | 1.39E-03 | 1.42E-03 | 3.30E-01 | 8.22E-03 | 1.45E-03 | 1.40E-08 |
| rs7439324 | 4 | 44501351 | C | T | 7.53E-03 | 1.86E-03 | 5.30E-05 | 1.08E-02 | 1.90E-03 | 1.50E-08 |
| rs2755253 | 1 | 67470843 | C | T | 1.53E-03 | 1.51E-03 | 3.10E-01 | 8.71E-03 | 1.54E-03 | 1.50E-08 |
| rs1177279 | 2 | 61295122 | A | G | 2.06E-03 | 1.53E-03 | 1.80E-01 | 8.83E-03 | 1.56E-03 | 1.60E-08 |
| rs60644673 | 7 | 100096742 | G | T | -4.06E-03 | 1.74E-03 | 2.00E-02 | 1.00E-02 | 1.78E-03 | 1.70E-08 |
| rs34722008 | 4 | 38659594 | G | A | 6.99E-03 | 1.44E-03 | 1.10E-06 | 8.27E-03 | 1.47E-03 | 1.80E-08 |
| rs1619120 | 9 | 87302196 | A | G | -6.79E-03 | 1.40E-03 | 1.30E-06 | -8.06E-03 | 1.43E-03 | 1.80E-08 |
| rs1696057 | 12 | 90767160 | T | C | 3.91E-03 | 1.45E-03 | 6.80E-03 | 8.31E-03 | 1.48E-03 | 1.80E-08 |
| rs6577497 | 1 | 8605667 | A | T | -2.20E-03 | 1.40E-03 | 1.20E-01 | 8.07E-03 | 1.43E-03 | 1.80E-08 |
| rs2958542 | 11 | 62181882 | C | T | 3.51E-04 | 1.43E-03 | 8.10E-01 | 8.24E-03 | 1.46E-03 | 1.80E-08 |
| rs10887571 | 10 | 88030441 | C | T | -6.82E-03 | 1.39E-03 | 9.10E-07 | -7.98E-03 | 1.42E-03 | 1.90E-08 |
| rs115903965 | 3 | 66009529 | G | A | 1.86E-03 | 4.44E-03 | 6.80E-01 | -2.55E-02 | 4.54E-03 | 2.00E-08 |
| rs2939931 | 10 | 121636406 | T | C | -2.74E-03 | 1.37E-03 | 4.60E-02 | -7.86E-03 | 1.40E-03 | 2.20E-08 |
| rs8130408 | 21 | 39237138 | A | C | -4.68E-03 | 1.58E-03 | 3.10E-03 | -9.01E-03 | 1.61E-03 | 2.30E-08 |
| rs3815156 | 17 | 29685150 | A | G | -5.29E-03 | 1.81E-03 | 3.40E-03 | -1.03E-02 | 1.84E-03 | 2.30E-08 |
| rs538579 | 3 | 62711674 | G | C | -7.99E-03 | 1.48E-03 | 6.30E-08 | -8.42E-03 | 1.51E-03 | 2.40E-08 |
| rs2268762 | 3 | 38516075 | A | G | -4.39E-03 | 1.40E-03 | 1.80E-03 | -7.99E-03 | 1.44E-03 | 2.60E-08 |
| rs1402989 | 3 | 27056851 | C | T | -4.03E-03 | 1.37E-03 | 3.30E-03 | -7.79E-03 | 1.40E-03 | 2.70E-08 |
| rs7840305 | 8 | 57168101 | A | G | 3.42E-03 | 1.42E-03 | 1.60E-02 | 8.06E-03 | 1.45E-03 | 2.70E-08 |
| rs6719507 | 2 | 29733801 | G | A | 3.02E-03 | 1.38E-03 | 2.90E-02 | 7.82E-03 | 1.41E-03 | 2.90E-08 |
| rs73085586 | 20 | 22430241 | G | A | -4.65E-03 | 1.72E-03 | 6.70E-03 | -9.71E-03 | 1.75E-03 | 3.00E-08 |
| rs11040333 | 11 | 49346332 | G | A | -3.98E-03 | 1.51E-03 | 8.50E-03 | -8.55E-03 | 1.54E-03 | 3.10E-08 |
| rs3791478 | 2 | 240064139 | T | C | 4.64E-03 | 2.21E-03 | 3.60E-02 | 1.25E-02 | 2.26E-03 | 3.30E-08 |
| rs594585 | 16 | 65939803 | T | G | 4.87E-03 | 1.41E-03 | 5.50E-04 | 7.95E-03 | 1.44E-03 | 3.40E-08 |
| rs10823504 | 10 | 72034062 | G | A | 9.02E-04 | 2.81E-03 | 7.50E-01 | 1.59E-02 | 2.87E-03 | 3.40E-08 |
| rs16839832 | 1 | 196349909 | G | T | -2.65E-03 | 2.50E-03 | 2.90E-01 | -1.41E-02 | 2.55E-03 | 3.50E-08 |
| rs76187039 | 6 | 43233990 | G | T | -1.12E-03 | 2.01E-03 | 5.80E-01 | -1.13E-02 | 2.05E-03 | 3.50E-08 |
| rs10791902 | 11 | 67093360 | C | T | -5.63E-03 | 1.41E-03 | 6.20E-05 | -7.91E-03 | 1.44E-03 | 3.60E-08 |
| rs2175171 | 1 | 7028842 | G | C | -1.93E-03 | 1.38E-03 | 1.60E-01 | -7.77E-03 | 1.41E-03 | 3.60E-08 |
| rs1000471 | 15 | 89986583 | C | T | 9.10E-04 | 1.69E-03 | 5.90E-01 | -9.51E-03 | 1.73E-03 | 3.60E-08 |
| rs9610387 | 22 | 36476762 | G | A | 7.89E-03 | 2.46E-03 | 1.30E-03 | 1.38E-02 | 2.51E-03 | 3.70E-08 |
| rs4677156 | 3 | 72417857 | A | T | 3.78E-03 | 1.64E-03 | 2.10E-02 | 9.25E-03 | 1.68E-03 | 3.70E-08 |
| rs3172332 | 3 | 153973408 | T | C | 2.69E-03 | 1.42E-03 | 5.80E-02 | 8.01E-03 | 1.46E-03 | 3.70E-08 |
| rs2157295 | 22 | 42701984 | T | G | -4.94E-03 | 1.42E-03 | 5.00E-04 | -7.96E-03 | 1.45E-03 | 3.80E-08 |
| rs11655704 | 17 | 47448172 | T | C | 2.44E-03 | 1.47E-03 | 9.70E-02 | 8.27E-03 | 1.50E-03 | 3.80E-08 |
| rs10503555 | 8 | 15763818 | A | G | -6.46E-04 | 1.39E-03 | 6.40E-01 | 7.78E-03 | 1.41E-03 | 3.90E-08 |
| rs762705 | 3 | 50313527 | A | C | 7.21E-03 | 1.92E-03 | 1.70E-04 | 1.08E-02 | 1.96E-03 | 4.00E-08 |
| rs68015088 | 18 | 51484010 | G | A | 5.01E-03 | 1.45E-03 | 5.50E-04 | 8.10E-03 | 1.48E-03 | 4.30E-08 |
| rs10111937 | 8 | 54160092 | C | T | -4.90E-03 | 1.50E-03 | 1.10E-03 | -8.37E-03 | 1.53E-03 | 4.30E-08 |
| rs62048187 | 15 | 38117049 | G | C | -6.01E-03 | 1.50E-03 | 6.10E-05 | -8.39E-03 | 1.53E-03 | 4.40E-08 |
| rs67679818 | 7 | 110672704 | C | T | 4.06E-04 | 1.40E-03 | 7.70E-01 | 7.80E-03 | 1.43E-03 | 4.90E-08 |

**Table S2D.** The genetic variants strongly associated with childhood adiposity at genome wide significance and not adulthood adiposity (exclude adult SNPs at P ≤ 5×10^-8^) in females.

| SNP | Chromosome | Base position | Effect allele | Other allele | Beta (Age 10) | SE (Age 10) | P (Age 10) | Beta (Adult) | SE (Adult) | P (Adult) |
| --- | --- | --- | --- | --- | --- | --- | --- | --- | --- | --- |
| rs212540 | 1 | 21593117 | C | T | 1.27E-02 | 2.00E-03 | 9.90E-10 | 1.56E-03 | 2.03E-03 | 4.40E-01 |
| rs582220 | 1 | 54724762 | A | G | -1.15E-02 | 1.96E-03 | 9.90E-09 | -7.97E-03 | 1.99E-03 | 6.20E-05 |
| rs2767486 | 1 | 65991203 | A | G | -2.03E-02 | 2.41E-03 | 9.90E-17 | -1.24E-03 | 2.45E-03 | 6.10E-01 |
| rs10798139 | 1 | 187714179 | C | T | -1.37E-02 | 2.36E-03 | 8.40E-09 | -8.76E-03 | 2.39E-03 | 2.50E-04 |
| rs4971239 | 1 | 203491150 | G | A | -1.49E-02 | 2.60E-03 | 9.90E-09 | -1.41E-02 | 2.65E-03 | 9.00E-08 |
| rs139197338 | 2 | 24864916 | C | T | -3.14E-02 | 5.07E-03 | 1.10E-09 | -1.67E-02 | 5.15E-03 | 1.20E-03 |
| rs142465373 | 2 | 77248236 | G | GTATC | 1.61E-02 | 2.28E-03 | 4.20E-12 | 7.31E-03 | 2.32E-03 | 1.60E-03 |
| rs1483153 | 2 | 142358477 | C | T | -1.45E-02 | 2.34E-03 | 3.40E-10 | -5.33E-03 | 2.37E-03 | 2.50E-02 |
| rs36134621 | 2 | 161046192 | G | A | 1.11E-02 | 1.95E-03 | 3.50E-08 | -2.61E-03 | 1.98E-03 | 1.90E-01 |
| rs17464221 | 2 | 188278203 | C | T | 1.23E-02 | 2.14E-03 | 1.50E-08 | 2.65E-03 | 2.17E-03 | 2.20E-01 |
| rs115319174 | 2 | 207066474 | G | C | -4.11E-02 | 4.20E-03 | 7.60E-23 | -7.38E-03 | 4.27E-03 | 8.40E-02 |
| rs7562973 | 2 | 219471455 | T | C | -1.10E-02 | 1.99E-03 | 3.00E-08 | -9.44E-03 | 2.02E-03 | 3.00E-06 |
| rs2594989 | 3 | 11316143 | C | T | 1.87E-02 | 2.53E-03 | 4.00E-14 | -4.61E-04 | 2.58E-03 | 8.60E-01 |
| rs754635 | 3 | 42305131 | C | G | -1.66E-02 | 3.05E-03 | 4.50E-08 | -1.60E-02 | 3.10E-03 | 2.30E-07 |
| rs2034963 | 3 | 48170802 | G | C | 1.34E-02 | 2.04E-03 | 5.40E-11 | 3.43E-03 | 2.08E-03 | 9.90E-02 |
| rs2629881 | 3 | 59778271 | C | T | -1.32E-02 | 2.34E-03 | 1.70E-08 | -4.55E-03 | 2.37E-03 | 5.50E-02 |
| rs79569013 | 3 | 61218295 | T | G | 2.01E-02 | 2.69E-03 | 1.50E-14 | 1.46E-02 | 2.73E-03 | 9.90E-08 |
| rs818219 | 3 | 85374589 | T | C | -1.34E-02 | 1.95E-03 | 1.50E-11 | -3.91E-03 | 1.98E-03 | 4.80E-02 |
| rs2735556 | 3 | 88105360 | T | C | 1.81E-02 | 3.02E-03 | 2.10E-09 | 1.08E-02 | 3.07E-03 | 4.40E-04 |
| rs5852911 | 3 | 138112533 | T | TTTC | 1.58E-02 | 2.46E-03 | 7.10E-11 | 8.21E-03 | 2.51E-03 | 1.00E-03 |
| rs7656673 | 4 | 30840331 | A | G | -1.37E-02 | 1.97E-03 | 1.70E-11 | -1.05E-02 | 2.01E-03 | 1.50E-07 |
| rs1349641 | 4 | 82212652 | T | G | 1.25E-02 | 1.98E-03 | 9.70E-10 | 2.39E-03 | 2.02E-03 | 2.40E-01 |
| rs7377083 | 4 | 102708997 | C | A | -1.54E-02 | 1.97E-03 | 1.10E-15 | -1.08E-02 | 2.00E-03 | 6.20E-08 |
| rs3936511 | 5 | 55860781 | A | G | 1.54E-02 | 2.46E-03 | 7.10E-11 | 8.43E-04 | 2.50E-03 | 7.40E-01 |
| rs10050620 | 5 | 63927239 | C | T | 1.59E-02 | 2.07E-03 | 2.90E-14 | 9.68E-03 | 2.10E-03 | 4.10E-06 |
| rs13190020 | 5 | 65012526 | G | A | -1.15E-02 | 2.03E-03 | 1.40E-08 | -1.57E-03 | 2.07E-03 | 4.50E-01 |
| rs4235642 | 5 | 103818412 | A | G | 1.24E-02 | 2.00E-03 | 7.70E-10 | -5.90E-03 | 2.03E-03 | 3.70E-03 |
| rs199724602 | 5 | 142879714 | CT | C | 1.09E-02 | 1.96E-03 | 2.10E-08 | 8.80E-03 | 1.99E-03 | 1.00E-05 |
| rs815610 | 5 | 153517178 | C | G | 1.53E-02 | 1.95E-03 | 7.20E-15 | 8.67E-03 | 1.98E-03 | 1.20E-05 |
| rs767647218 | 6 | 10015295 | TTA | T | 1.37E-02 | 2.05E-03 | 2.40E-11 | 6.45E-03 | 2.09E-03 | 2.00E-03 |
| rs146863150 | 6 | 26408472 | C | CATAT | -2.52E-02 | 3.12E-03 | 6.70E-16 | -1.24E-02 | 3.18E-03 | 1.00E-04 |
| rs34196306 | 6 | 27425644 | G | C | -2.62E-02 | 3.16E-03 | 1.00E-16 | -1.12E-02 | 3.21E-03 | 5.00E-04 |
| rs3749971 | 6 | 29342775 | G | A | -2.38E-02 | 2.95E-03 | 1.50E-15 | -1.11E-02 | 3.00E-03 | 2.30E-04 |
| rs3131934 | 6 | 30931844 | T | C | -2.27E-02 | 2.58E-03 | 9.00E-18 | -8.53E-03 | 2.62E-03 | 1.20E-03 |
| rs9268235 | 6 | 32290208 | C | T | -2.07E-02 | 2.80E-03 | 2.30E-13 | -9.52E-03 | 2.85E-03 | 8.40E-04 |
| rs1775255 | 6 | 51243035 | G | T | -1.37E-02 | 1.94E-03 | 1.40E-12 | -9.60E-03 | 1.97E-03 | 1.20E-06 |
| rs12110721 | 6 | 55190480 | G | A | -1.93E-02 | 2.61E-03 | 4.70E-14 | -6.96E-03 | 2.66E-03 | 8.70E-03 |
| rs34260097 | 6 | 100727703 | T | G | -2.52E-02 | 2.32E-03 | 2.40E-28 | -3.62E-03 | 2.36E-03 | 1.20E-01 |
| rs7759938 | 6 | 105378954 | C | T | -1.16E-02 | 2.07E-03 | 1.70E-08 | -1.87E-03 | 2.11E-03 | 3.80E-01 |
| rs796915 | 6 | 154304628 | C | G | -1.60E-02 | 2.10E-03 | 7.40E-15 | -8.60E-03 | 2.14E-03 | 5.80E-05 |
| rs62425122 | 6 | 166311987 | G | A | 1.24E-02 | 2.12E-03 | 4.10E-09 | 3.30E-03 | 2.15E-03 | 1.30E-01 |
| rs983949 | 7 | 24299013 | T | G | -1.30E-02 | 2.15E-03 | 4.10E-09 | -5.59E-03 | 2.18E-03 | 1.00E-02 |
| rs75001243 | 7 | 93216002 | C | T | -1.59E-02 | 2.02E-03 | 1.70E-15 | -7.50E-03 | 2.05E-03 | 2.50E-04 |
| rs7808296 | 7 | 103127620 | C | T | -1.17E-02 | 2.08E-03 | 4.80E-08 | -3.17E-03 | 2.12E-03 | 1.30E-01 |
| rs6979832 | 7 | 127856276 | A | G | -1.13E-02 | 1.95E-03 | 1.60E-08 | -3.09E-03 | 1.98E-03 | 1.20E-01 |
| rs13233916 | 7 | 138874416 | C | G | 2.10E-02 | 3.39E-03 | 6.10E-10 | 1.49E-02 | 3.45E-03 | 1.60E-05 |
| rs12334731 | 8 | 11098975 | G | A | 1.23E-02 | 2.00E-03 | 4.40E-10 | 7.70E-03 | 2.03E-03 | 1.50E-04 |
| rs351776 | 8 | 28191306 | A | C | -1.17E-02 | 1.94E-03 | 2.00E-09 | -3.90E-03 | 1.98E-03 | 4.90E-02 |
| rs62515439 | 8 | 57165417 | C | T | 1.27E-02 | 2.01E-03 | 1.20E-10 | 3.68E-03 | 2.04E-03 | 7.20E-02 |
| rs13254613 | 8 | 64804804 | A | C | -1.66E-02 | 2.03E-03 | 1.50E-16 | 8.56E-03 | 2.07E-03 | 3.60E-05 |
| rs35422050 | 8 | 76862206 | A | AC | 1.98E-02 | 1.97E-03 | 6.50E-24 | 9.66E-03 | 2.00E-03 | 1.40E-06 |
| rs818898 | 9 | 6970806 | A | G | 1.39E-02 | 2.10E-03 | 1.50E-11 | 7.55E-03 | 2.14E-03 | 4.20E-04 |
| rs10821163 | 9 | 96343060 | G | C | -1.84E-02 | 2.05E-03 | 3.50E-19 | 2.15E-03 | 2.09E-03 | 3.00E-01 |
| rs957512 | 9 | 120405705 | T | C | 1.15E-02 | 2.06E-03 | 2.10E-08 | 8.54E-03 | 2.10E-03 | 4.60E-05 |
| rs2275241 | 9 | 129370576 | G | A | -1.38E-02 | 2.00E-03 | 2.30E-12 | -6.74E-03 | 2.04E-03 | 9.40E-04 |
| rs7084503 | 10 | 2666859 | T | C | 1.54E-02 | 1.94E-03 | 2.00E-16 | 5.66E-03 | 1.97E-03 | 4.20E-03 |
| rs551169574 | 10 | 87351591 | TA | T | -2.62E-02 | 3.89E-03 | 7.90E-12 | -2.08E-02 | 3.96E-03 | 1.40E-07 |
| rs79855417 | 11 | 29189203 | T | TAC | 1.90E-02 | 2.85E-03 | 7.40E-11 | 6.20E-03 | 2.90E-03 | 3.30E-02 |
| rs59604964 | 11 | 69501586 | C | CTGATG | -1.22E-02 | 2.01E-03 | 1.00E-09 | -9.48E-03 | 2.04E-03 | 3.40E-06 |
| rs11215403 | 11 | 115058585 | G | A | 1.62E-02 | 2.25E-03 | 7.90E-13 | 6.71E-03 | 2.29E-03 | 3.40E-03 |
| rs2187642 | 12 | 11855624 | A | C | -1.19E-02 | 1.99E-03 | 3.80E-09 | -5.08E-04 | 2.03E-03 | 8.00E-01 |
| rs10876457 | 12 | 39453689 | G | A | 1.28E-02 | 2.32E-03 | 2.90E-08 | 6.15E-03 | 2.37E-03 | 9.30E-03 |
| rs78607331 | 12 | 57648644 | C | T | -2.89E-02 | 4.67E-03 | 1.20E-09 | -1.54E-02 | 4.75E-03 | 1.20E-03 |
| rs10784514 | 12 | 66452879 | C | T | -1.16E-02 | 2.07E-03 | 4.20E-08 | -5.94E-04 | 2.11E-03 | 7.80E-01 |
| rs2364232 | 12 | 93994827 | A | C | 1.23E-02 | 2.21E-03 | 2.10E-08 | 1.02E-03 | 2.25E-03 | 6.50E-01 |
| rs60550417 | 12 | 99644477 | A | AGATAATTCATT | -1.35E-02 | 2.35E-03 | 2.80E-09 | -9.64E-03 | 2.39E-03 | 5.60E-05 |
| rs7305424 | 12 | 118399491 | A | T | -1.21E-02 | 2.05E-03 | 5.30E-09 | -3.79E-03 | 2.08E-03 | 6.90E-02 |
| rs4771106 | 13 | 27929146 | T | C | -1.31E-02 | 2.25E-03 | 4.10E-09 | -7.81E-03 | 2.29E-03 | 6.70E-04 |
| rs9551428 | 13 | 28618462 | C | T | 1.53E-02 | 2.00E-03 | 2.90E-14 | 6.26E-03 | 2.04E-03 | 2.10E-03 |
| rs1336486 | 13 | 40784814 | T | G | -1.47E-02 | 2.06E-03 | 1.90E-12 | -6.46E-03 | 2.10E-03 | 2.10E-03 |
| rs9317002 | 13 | 59175727 | C | A | -1.38E-02 | 1.95E-03 | 2.80E-12 | -8.76E-03 | 1.98E-03 | 1.00E-05 |
| rs58681688 | 13 | 62467001 | C | G | -1.37E-02 | 2.50E-03 | 1.90E-08 | -7.24E-03 | 2.54E-03 | 4.40E-03 |
| rs59041875 | 13 | 66203733 | AG | A | 1.16E-02 | 2.05E-03 | 7.40E-09 | 7.86E-03 | 2.08E-03 | 1.60E-04 |
| rs61980008 | 14 | 30464716 | G | A | -3.66E-02 | 5.01E-03 | 4.60E-13 | -2.22E-02 | 5.10E-03 | 1.30E-05 |
| rs4932430 | 15 | 89363866 | A | C | 1.18E-02 | 1.95E-03 | 3.70E-09 | 4.89E-03 | 1.99E-03 | 1.40E-02 |
| rs2970356 | 15 | 90623540 | C | G | -1.33E-02 | 2.19E-03 | 7.70E-10 | -5.48E-03 | 2.23E-03 | 1.40E-02 |
| rs72755233 | 15 | 100692953 | G | A | -1.78E-02 | 3.07E-03 | 1.20E-08 | -1.06E-02 | 3.13E-03 | 7.40E-04 |
| rs8049326 | 16 | 3572268 | G | A | -1.22E-02 | 2.17E-03 | 9.40E-09 | -4.98E-03 | 2.20E-03 | 2.40E-02 |
| rs11863799 | 16 | 61933401 | C | T | 1.14E-02 | 2.07E-03 | 1.80E-08 | 1.35E-03 | 2.10E-03 | 5.20E-01 |
| rs34229857 | 16 | 67434917 | C | T | -4.47E-02 | 5.78E-03 | 1.70E-15 | -1.80E-02 | 5.88E-03 | 2.20E-03 |
| rs11642090 | 16 | 81730582 | T | C | -1.34E-02 | 2.02E-03 | 2.20E-11 | -9.49E-03 | 2.05E-03 | 3.70E-06 |
| rs999493 | 17 | 46625519 | G | A | -1.12E-02 | 2.01E-03 | 6.30E-09 | -5.23E-03 | 2.04E-03 | 1.00E-02 |
| rs12185242 | 17 | 47407071 | A | C | -1.10E-02 | 1.94E-03 | 7.10E-09 | -2.62E-03 | 1.98E-03 | 1.90E-01 |
| rs1013737 | 18 | 937050 | G | C | -1.09E-02 | 1.94E-03 | 1.80E-08 | -5.75E-03 | 1.97E-03 | 3.60E-03 |
| rs7239114 | 18 | 45921214 | G | A | -1.41E-02 | 1.96E-03 | 4.10E-13 | -8.76E-03 | 1.99E-03 | 1.10E-05 |
| rs12606230 | 18 | 52492252 | T | C | -1.26E-02 | 2.29E-03 | 2.50E-08 | -1.02E-02 | 2.33E-03 | 1.10E-05 |
| rs3810304 | 19 | 30861683 | A | G | 1.43E-02 | 2.32E-03 | 1.30E-09 | 1.90E-04 | 2.36E-03 | 9.40E-01 |
| rs4805881 | 19 | 33896432 | A | C | -1.17E-02 | 2.05E-03 | 4.50E-08 | -5.86E-03 | 2.09E-03 | 5.00E-03 |
| rs633372 | 19 | 49209226 | G | A | 1.11E-02 | 1.94E-03 | 5.70E-09 | 1.84E-03 | 1.97E-03 | 3.50E-01 |
| rs994308 | 20 | 6603622 | C | T | -1.24E-02 | 1.97E-03 | 5.30E-10 | -9.30E-03 | 2.01E-03 | 3.60E-06 |
| rs7268466 | 20 | 15810676 | C | T | -2.01E-02 | 2.78E-03 | 7.40E-13 | -8.36E-03 | 2.83E-03 | 3.10E-03 |
| rs947088 | 20 | 17171373 | G | T | -1.25E-02 | 2.16E-03 | 3.50E-09 | -1.01E-02 | 2.20E-03 | 4.60E-06 |
| rs763842194 | 20 | 54377089 | AG | A | 1.50E-02 | 2.20E-03 | 1.30E-11 | 2.91E-03 | 2.23E-03 | 1.90E-01 |
| rs11399239 | 21 | 40310590 | G | GT | 1.40E-02 | 2.04E-03 | 7.00E-12 | 9.14E-03 | 2.08E-03 | 1.10E-05 |
| rs374873051 | 22 | 22266944 | CTTTTTTTTTTTTT | C | -1.13E-02 | 2.02E-03 | 1.70E-09 | -2.21E-03 | 2.06E-03 | 2.80E-01 |

**Table S2E.** The genetic variants strongly associated with adulthood adiposity at genome wide significance and not childhood adiposity (exclude child SNPs at P ≤ 5×10^-8^).

| SNP | Chromosome | Base position | Effect allele | Other allele | Beta (Adult) | SE (Adult) | P (Adult) | Beta (Age 10) | SE (Age 10) | P (Age 10) |
| --- | --- | --- | --- | --- | --- | --- | --- | --- | --- | --- |
| rs4764949 | 12 | 103658096 | A | G | 1.19E-02 | 1.47E-03 | 3.80E-16 | 8.01E-03 | 1.50E-03 | 8.50E-08 |
| rs112253053 | 19 | 19425145 | T | A | 1.38E-02 | 1.87E-03 | 1.80E-13 | 1.01E-02 | 1.91E-03 | 1.10E-07 |
| rs28350 | 3 | 42418446 | A | G | 1.26E-02 | 1.79E-03 | 1.70E-12 | 9.70E-03 | 1.83E-03 | 1.20E-07 |
| rs512121 | 18 | 7548501 | T | C | 1.06E-02 | 1.75E-03 | 1.50E-09 | 9.31E-03 | 1.79E-03 | 1.90E-07 |
| rs72673947 | 8 | 118884379 | A | G | -1.40E-02 | 2.23E-03 | 3.30E-10 | -1.18E-02 | 2.28E-03 | 2.10E-07 |
| rs12951079 | 17 | 34933059 | G | A | 1.08E-02 | 1.41E-03 | 1.60E-14 | 7.42E-03 | 1.43E-03 | 2.30E-07 |
| rs61992671 | 14 | 101531854 | A | G | 9.88E-03 | 1.43E-03 | 5.70E-12 | 7.58E-03 | 1.47E-03 | 2.30E-07 |
| rs10146997 | 14 | 79945162 | A | G | -1.60E-02 | 1.65E-03 | 3.20E-22 | -8.69E-03 | 1.69E-03 | 2.60E-07 |
| rs62004865 | 15 | 74207695 | T | A | -1.36E-02 | 2.24E-03 | 1.40E-09 | -1.18E-02 | 2.29E-03 | 2.70E-07 |
| rs869400 | 3 | 185826740 | T | G | -1.83E-02 | 1.77E-03 | 3.50E-25 | -9.22E-03 | 1.81E-03 | 3.30E-07 |
| rs9366863 | 6 | 34688946 | T | C | 1.74E-02 | 1.46E-03 | 8.50E-33 | 7.54E-03 | 1.49E-03 | 4.10E-07 |
| rs1423534 | 5 | 63977000 | G | A | 7.71E-03 | 1.39E-03 | 3.20E-08 | 7.14E-03 | 1.42E-03 | 5.30E-07 |
| rs3861871 | 9 | 129424719 | A | G | -8.29E-03 | 1.39E-03 | 2.60E-09 | -7.12E-03 | 1.42E-03 | 5.40E-07 |
| rs4971239 | 1 | 203491150 | G | A | -1.15E-02 | 1.84E-03 | 4.50E-10 | -9.43E-03 | 1.88E-03 | 5.50E-07 |
| rs67257872 | 11 | 8530218 | A | G | 1.06E-02 | 1.38E-03 | 1.50E-14 | 7.02E-03 | 1.41E-03 | 6.30E-07 |
| rs8089514 | 18 | 69224478 | T | A | -8.17E-03 | 1.44E-03 | 1.40E-08 | -7.32E-03 | 1.47E-03 | 6.30E-07 |
| rs2051559 | 4 | 3298800 | T | C | -1.39E-02 | 2.03E-03 | 8.30E-12 | -1.03E-02 | 2.07E-03 | 6.60E-07 |
| rs8015400 | 14 | 25930988 | C | A | -1.26E-02 | 1.47E-03 | 6.90E-18 | -7.34E-03 | 1.50E-03 | 9.80E-07 |
| rs73026723 | 19 | 31017177 | C | T | 1.41E-02 | 1.90E-03 | 1.30E-13 | 9.49E-03 | 1.94E-03 | 1.00E-06 |
| rs61909165 | 11 | 134589355 | T | A | -1.23E-02 | 1.82E-03 | 1.60E-11 | -8.87E-03 | 1.86E-03 | 1.90E-06 |
| rs8008772 | 14 | 88321884 | A | T | -9.26E-03 | 1.59E-03 | 5.70E-09 | -7.72E-03 | 1.62E-03 | 2.00E-06 |
| rs6679458 | 1 | 96946253 | G | T | -1.16E-02 | 1.39E-03 | 9.10E-17 | -6.70E-03 | 1.42E-03 | 2.40E-06 |
| rs9788550 | 14 | 29681138 | G | C | 1.38E-02 | 1.60E-03 | 5.60E-18 | 7.68E-03 | 1.63E-03 | 2.40E-06 |
| rs1834144 | 18 | 40744790 | C | A | 7.93E-03 | 1.42E-03 | 2.50E-08 | 6.82E-03 | 1.45E-03 | 2.70E-06 |
| rs10404726 | 19 | 18834514 | C | T | 1.23E-02 | 1.38E-03 | 4.70E-19 | 6.56E-03 | 1.41E-03 | 3.20E-06 |
| rs34898535 | 16 | 31025641 | C | T | 1.43E-02 | 1.41E-03 | 4.80E-24 | 6.67E-03 | 1.45E-03 | 3.90E-06 |
| rs112859723 | 3 | 131625376 | T | C | 1.11E-02 | 1.91E-03 | 5.50E-09 | 8.82E-03 | 1.95E-03 | 6.40E-06 |
| rs138329430 | 1 | 174619318 | G | A | -1.28E-02 | 2.19E-03 | 5.40E-09 | -1.01E-02 | 2.24E-03 | 7.20E-06 |
| rs28465175 | 15 | 53427155 | A | G | 1.50E-02 | 2.70E-03 | 2.60E-08 | 1.24E-02 | 2.76E-03 | 7.20E-06 |
| rs73982435 | 17 | 31473455 | C | T | 9.43E-03 | 1.67E-03 | 1.60E-08 | 7.56E-03 | 1.70E-03 | 9.00E-06 |
| rs6669341 | 1 | 47678458 | A | G | 1.07E-02 | 1.39E-03 | 1.30E-14 | 6.28E-03 | 1.42E-03 | 9.60E-06 |
| rs10969334 | 9 | 29717279 | C | A | 8.67E-03 | 1.41E-03 | 6.90E-10 | 6.34E-03 | 1.44E-03 | 1.00E-05 |
| rs11223204 | 11 | 132652554 | A | G | -8.74E-03 | 1.39E-03 | 3.00E-10 | -6.17E-03 | 1.42E-03 | 1.30E-05 |
| rs862320 | 16 | 69651866 | C | T | 1.44E-02 | 1.40E-03 | 6.20E-25 | 6.19E-03 | 1.43E-03 | 1.40E-05 |
| rs113230003 | 19 | 18460956 | G | A | 1.26E-02 | 1.58E-03 | 1.20E-15 | 7.00E-03 | 1.61E-03 | 1.40E-05 |
| rs3814883 | 16 | 29994922 | C | T | -1.48E-02 | 1.38E-03 | 8.10E-27 | -6.09E-03 | 1.41E-03 | 1.50E-05 |
| rs11976084 | 7 | 137437156 | C | T | -8.57E-03 | 1.52E-03 | 1.70E-08 | -6.61E-03 | 1.55E-03 | 2.10E-05 |
| rs17773370 | 18 | 57951433 | G | A | -1.59E-02 | 2.76E-03 | 8.70E-09 | -1.19E-02 | 2.82E-03 | 2.60E-05 |
| rs12454712 | 18 | 60845884 | T | C | -8.11E-03 | 1.42E-03 | 1.00E-08 | -6.03E-03 | 1.45E-03 | 3.10E-05 |
| rs815163 | 1 | 190294726 | T | C | 1.08E-02 | 1.38E-03 | 6.10E-15 | 5.84E-03 | 1.41E-03 | 3.40E-05 |
| rs6938973 | 6 | 98421721 | T | C | -1.18E-02 | 1.40E-03 | 3.50E-17 | 5.90E-03 | 1.43E-03 | 3.80E-05 |
| rs827803 | 3 | 157920266 | G | T | 7.87E-03 | 1.38E-03 | 1.20E-08 | 5.81E-03 | 1.41E-03 | 3.90E-05 |
| rs7752998 | 6 | 34398358 | A | T | 1.02E-02 | 1.75E-03 | 4.60E-09 | 7.34E-03 | 1.79E-03 | 3.90E-05 |
| rs59428052 | 2 | 53861389 | A | G | 1.15E-02 | 2.00E-03 | 9.80E-09 | 8.33E-03 | 2.04E-03 | 4.60E-05 |
| rs10736156 | 10 | 104019447 | C | A | -1.18E-02 | 1.89E-03 | 3.80E-10 | -7.85E-03 | 1.93E-03 | 4.80E-05 |
| rs329651 | 11 | 133767622 | G | T | -1.11E-02 | 1.74E-03 | 1.70E-10 | -7.17E-03 | 1.77E-03 | 5.20E-05 |
| rs34778589 | 22 | 50709957 | A | C | -1.43E-02 | 2.50E-03 | 9.80E-09 | -1.03E-02 | 2.55E-03 | 5.30E-05 |
| rs55838622 | 5 | 95711605 | A | C | -9.90E-03 | 1.64E-03 | 1.70E-09 | -6.73E-03 | 1.68E-03 | 6.10E-05 |
| rs4790292 | 17 | 1824305 | C | A | 1.63E-02 | 1.91E-03 | 1.30E-17 | 7.82E-03 | 1.95E-03 | 6.10E-05 |
| rs35775580 | 7 | 130420740 | A | G | 1.80E-02 | 3.24E-03 | 2.70E-08 | 1.32E-02 | 3.31E-03 | 6.30E-05 |
| rs34298980 | 6 | 40409243 | T | C | 1.33E-02 | 1.44E-03 | 4.40E-20 | 5.88E-03 | 1.48E-03 | 6.70E-05 |
| rs6606686 | 12 | 110903380 | G | C | 9.75E-03 | 1.47E-03 | 3.40E-11 | 5.96E-03 | 1.50E-03 | 7.10E-05 |
| rs4671328 | 2 | 58935282 | T | G | 1.32E-02 | 1.39E-03 | 2.10E-21 | 5.62E-03 | 1.42E-03 | 7.30E-05 |
| rs1477290 | 5 | 87988934 | T | C | -1.99E-02 | 2.01E-03 | 3.50E-23 | -8.15E-03 | 2.05E-03 | 7.30E-05 |
| rs62277889 | 2 | 198783693 | C | T | 1.06E-02 | 1.58E-03 | 2.10E-11 | 6.41E-03 | 1.62E-03 | 7.40E-05 |
| rs4467770 | 6 | 12086826 | G | A | -9.87E-03 | 1.55E-03 | 2.00E-10 | -6.25E-03 | 1.59E-03 | 8.10E-05 |
| rs28711392 | 11 | 13349559 | T | C | 1.18E-02 | 1.43E-03 | 2.10E-16 | 5.76E-03 | 1.46E-03 | 8.40E-05 |
| rs1517037 | 18 | 56878274 | C | T | 9.92E-03 | 1.76E-03 | 1.60E-08 | 7.03E-03 | 1.79E-03 | 8.90E-05 |
| rs6575340 | 14 | 94023972 | G | A | -1.33E-02 | 1.43E-03 | 1.20E-20 | -5.69E-03 | 1.46E-03 | 9.70E-05 |
| rs2678204 | 1 | 201800511 | T | G | -1.50E-02 | 1.44E-03 | 2.70E-25 | -5.75E-03 | 1.48E-03 | 9.90E-05 |
| rs35193668 | 13 | 33092929 | C | T | 1.05E-02 | 1.43E-03 | 2.50E-13 | 5.68E-03 | 1.46E-03 | 1.00E-04 |
| rs8111074 | 19 | 51776117 | G | T | 8.39E-03 | 1.52E-03 | 3.20E-08 | 6.01E-03 | 1.55E-03 | 1.00E-04 |
| rs2417998 | 9 | 111958746 | C | G | 8.41E-03 | 1.52E-03 | 2.80E-08 | 5.97E-03 | 1.55E-03 | 1.20E-04 |
| rs7453694 | 6 | 51739528 | C | T | -9.79E-03 | 1.52E-03 | 1.20E-10 | -5.98E-03 | 1.55E-03 | 1.20E-04 |
| rs114593013 | 3 | 84113491 | A | G | 1.97E-02 | 2.94E-03 | 2.10E-11 | 1.16E-02 | 3.01E-03 | 1.20E-04 |
| rs10749659 | 1 | 151033979 | C | T | 1.02E-02 | 1.63E-03 | 4.40E-10 | 6.39E-03 | 1.67E-03 | 1.30E-04 |
| rs57590313 | 4 | 113323430 | C | A | -1.02E-02 | 1.79E-03 | 1.30E-08 | -6.98E-03 | 1.83E-03 | 1.30E-04 |
| rs75706763 | 2 | 145669168 | A | G | -1.77E-02 | 3.14E-03 | 1.70E-08 | -1.21E-02 | 3.21E-03 | 1.50E-04 |
| rs28462076 | 4 | 65696174 | A | G | 9.39E-03 | 1.62E-03 | 6.70E-09 | 6.24E-03 | 1.66E-03 | 1.60E-04 |
| rs59893724 | 5 | 80830788 | A | G | 1.14E-02 | 1.60E-03 | 8.70E-13 | 6.14E-03 | 1.63E-03 | 1.70E-04 |
| rs1582931 | 5 | 122657199 | G | A | 9.66E-03 | 1.38E-03 | 3.00E-12 | 5.30E-03 | 1.41E-03 | 1.80E-04 |
| rs10799778 | 1 | 23313353 | T | G | 1.26E-02 | 1.84E-03 | 7.80E-12 | 7.05E-03 | 1.88E-03 | 1.80E-04 |
| rs12187066 | 5 | 88800355 | A | G | -1.02E-02 | 1.58E-03 | 9.80E-11 | -5.99E-03 | 1.61E-03 | 2.00E-04 |
| rs79236537 | 5 | 86727690 | G | T | -2.99E-02 | 4.89E-03 | 1.00E-09 | -1.85E-02 | 5.00E-03 | 2.20E-04 |
| rs3931548 | 9 | 103113652 | C | A | -1.05E-02 | 1.44E-03 | 2.80E-13 | -5.41E-03 | 1.47E-03 | 2.30E-04 |
| rs1286138 | 14 | 91485445 | T | G | -9.00E-03 | 1.47E-03 | 8.40E-10 | -5.44E-03 | 1.50E-03 | 2.80E-04 |
| rs4482463 | 2 | 205375909 | C | A | 1.89E-02 | 2.57E-03 | 2.30E-13 | 9.47E-03 | 2.63E-03 | 3.20E-04 |
| rs1805123 | 7 | 150645534 | T | G | 1.10E-02 | 1.59E-03 | 4.10E-12 | 5.84E-03 | 1.63E-03 | 3.30E-04 |
| rs12788343 | 11 | 131452912 | T | C | -9.65E-03 | 1.39E-03 | 4.30E-12 | -5.10E-03 | 1.42E-03 | 3.40E-04 |
| rs142315514 | 1 | 147050816 | C | A | -2.14E-02 | 3.79E-03 | 1.70E-08 | -1.39E-02 | 3.87E-03 | 3.50E-04 |
| rs2450444 | 10 | 93010383 | G | A | 8.07E-03 | 1.44E-03 | 2.00E-08 | 5.24E-03 | 1.47E-03 | 3.60E-04 |
| rs2253310 | 6 | 108888593 | C | G | -1.06E-02 | 1.42E-03 | 9.00E-14 | -5.14E-03 | 1.45E-03 | 3.90E-04 |
| rs56803094 | 15 | 99222509 | A | G | 9.58E-03 | 1.65E-03 | 5.80E-09 | 5.94E-03 | 1.68E-03 | 4.00E-04 |
| rs6963840 | 7 | 78144371 | C | T | -1.29E-02 | 1.89E-03 | 8.50E-12 | -6.84E-03 | 1.93E-03 | 4.10E-04 |
| rs12147845 | 14 | 101144596 | C | T | -1.36E-02 | 2.15E-03 | 2.60E-10 | -7.75E-03 | 2.19E-03 | 4.10E-04 |
| rs1296685 | 22 | 18230964 | A | G | -9.49E-03 | 1.70E-03 | 2.20E-08 | -6.09E-03 | 1.73E-03 | 4.40E-04 |
| rs115866895 | 1 | 1592638 | A | G | 1.20E-02 | 1.57E-03 | 1.70E-14 | 5.60E-03 | 1.60E-03 | 4.70E-04 |
| rs4677813 | 3 | 194863860 | T | C | 8.93E-03 | 1.58E-03 | 1.60E-08 | 5.60E-03 | 1.62E-03 | 5.30E-04 |
| rs72618637 | 2 | 48953979 | T | A | 9.99E-03 | 1.77E-03 | 1.80E-08 | 6.29E-03 | 1.81E-03 | 5.30E-04 |
| rs6548220 | 2 | 225951 | A | G | 9.36E-03 | 1.48E-03 | 2.80E-10 | 5.24E-03 | 1.52E-03 | 5.50E-04 |
| rs13329943 | 16 | 24733751 | C | T | -1.13E-02 | 1.55E-03 | 3.20E-13 | -5.46E-03 | 1.59E-03 | 5.70E-04 |
| rs12634936 | 3 | 147716498 | T | C | -1.81E-02 | 3.15E-03 | 8.80E-09 | -1.11E-02 | 3.22E-03 | 5.80E-04 |
| rs12462975 | 19 | 30272202 | G | A | -1.15E-02 | 1.47E-03 | 5.90E-15 | -5.15E-03 | 1.50E-03 | 6.00E-04 |
| rs11633022 | 15 | 73074890 | C | A | -1.09E-02 | 1.44E-03 | 4.30E-14 | -5.04E-03 | 1.47E-03 | 6.30E-04 |
| rs3823674 | 7 | 50571996 | C | T | 7.66E-03 | 1.39E-03 | 3.50E-08 | 4.84E-03 | 1.42E-03 | 6.40E-04 |
| rs6843852 | 4 | 162132758 | C | T | -8.72E-03 | 1.37E-03 | 2.00E-10 | -4.77E-03 | 1.40E-03 | 6.70E-04 |
| rs1633418 | 22 | 20091756 | T | C | 7.92E-03 | 1.41E-03 | 2.00E-08 | 4.89E-03 | 1.44E-03 | 6.90E-04 |
| rs6798941 | 3 | 52893465 | C | T | -1.10E-02 | 1.51E-03 | 2.70E-13 | -5.21E-03 | 1.54E-03 | 7.20E-04 |
| rs9814758 | 3 | 123062657 | T | G | 8.39E-03 | 1.44E-03 | 5.40E-09 | 4.89E-03 | 1.47E-03 | 8.80E-04 |
| rs7102934 | 11 | 84648068 | T | C | -9.08E-03 | 1.49E-03 | 1.20E-09 | -5.07E-03 | 1.53E-03 | 8.90E-04 |
| rs113962925 | 17 | 46044446 | C | T | -1.71E-02 | 2.64E-03 | 8.60E-11 | -8.95E-03 | 2.69E-03 | 8.90E-04 |
| rs78565420 | 8 | 85703065 | C | T | -1.88E-02 | 3.15E-03 | 2.70E-09 | -1.07E-02 | 3.22E-03 | 9.20E-04 |
| rs112875651 | 8 | 126506694 | G | A | -8.35E-03 | 1.42E-03 | 4.30E-09 | -4.79E-03 | 1.45E-03 | 9.70E-04 |
| rs811054 | 16 | 72251132 | C | T | -8.64E-03 | 1.38E-03 | 4.40E-10 | -4.66E-03 | 1.41E-03 | 9.80E-04 |
| rs80082536 | 3 | 35195311 | A | G | -1.29E-02 | 2.12E-03 | 1.30E-09 | -7.15E-03 | 2.17E-03 | 9.90E-04 |
| rs8038574 | 15 | 95275890 | T | C | 9.07E-03 | 1.45E-03 | 4.00E-10 | 4.85E-03 | 1.48E-03 | 1.10E-03 |
| rs13061117 | 3 | 181186466 | T | C | -1.47E-02 | 2.42E-03 | 1.30E-09 | -7.99E-03 | 2.48E-03 | 1.20E-03 |
| rs273505 | 19 | 18217147 | T | C | -1.01E-02 | 1.39E-03 | 3.10E-13 | -4.52E-03 | 1.42E-03 | 1.40E-03 |
| rs12517187 | 5 | 112444682 | C | T | -8.25E-03 | 1.39E-03 | 2.80E-09 | -4.51E-03 | 1.42E-03 | 1.50E-03 |
| rs2289379 | 7 | 44804225 | C | T | 9.76E-03 | 1.41E-03 | 4.10E-12 | 4.57E-03 | 1.44E-03 | 1.50E-03 |
| rs2186118 | 1 | 66456465 | C | A | 9.86E-03 | 1.51E-03 | 6.90E-11 | 4.90E-03 | 1.55E-03 | 1.50E-03 |
| rs62171698 | 2 | 143959096 | C | A | -1.21E-02 | 1.97E-03 | 8.80E-10 | -6.39E-03 | 2.01E-03 | 1.50E-03 |
| rs1167311 | 1 | 49996959 | G | A | 1.23E-02 | 1.48E-03 | 1.20E-16 | 4.77E-03 | 1.51E-03 | 1.60E-03 |
| rs2875762 | 6 | 124925032 | G | C | -1.10E-02 | 1.60E-03 | 6.00E-12 | -5.17E-03 | 1.64E-03 | 1.60E-03 |
| rs1436348 | 3 | 104612668 | A | G | -9.66E-03 | 1.39E-03 | 3.50E-12 | -4.46E-03 | 1.42E-03 | 1.70E-03 |
| rs845084 | 10 | 125220036 | G | A | -9.85E-03 | 1.57E-03 | 3.70E-10 | -5.04E-03 | 1.61E-03 | 1.70E-03 |
| rs724623 | 14 | 47303577 | A | C | 1.02E-02 | 1.37E-03 | 9.00E-14 | 4.37E-03 | 1.40E-03 | 1.80E-03 |
| rs7103389 | 11 | 881639 | T | C | -9.46E-03 | 1.42E-03 | 2.90E-11 | -4.53E-03 | 1.45E-03 | 1.80E-03 |
| rs34994596 | 15 | 80991447 | T | C | 1.12E-02 | 1.50E-03 | 1.10E-13 | 4.77E-03 | 1.53E-03 | 1.80E-03 |
| rs544200874 | 4 | 20124826 | C | T | -1.77E-02 | 2.71E-03 | 7.20E-11 | -8.65E-03 | 2.77E-03 | 1.80E-03 |
| rs4672338 | 2 | 60217457 | C | T | -8.35E-03 | 1.45E-03 | 8.00E-09 | -4.60E-03 | 1.48E-03 | 1.90E-03 |
| rs4648450 | 1 | 2723214 | C | A | 9.80E-03 | 1.38E-03 | 1.20E-12 | 4.35E-03 | 1.41E-03 | 2.00E-03 |
| rs3125326 | 10 | 63053788 | A | C | -7.89E-03 | 1.42E-03 | 2.70E-08 | -4.48E-03 | 1.45E-03 | 2.00E-03 |
| rs11856579 | 15 | 78012688 | G | A | 9.18E-03 | 1.55E-03 | 3.40E-09 | 4.88E-03 | 1.59E-03 | 2.10E-03 |
| rs114263339 | 5 | 50932343 | C | T | -2.53E-02 | 4.33E-03 | 5.20E-09 | -1.36E-02 | 4.42E-03 | 2.10E-03 |
| rs1477890 | 4 | 18511738 | A | G | -9.78E-03 | 1.37E-03 | 1.10E-12 | -4.27E-03 | 1.40E-03 | 2.40E-03 |
| rs6029180 | 20 | 39178923 | A | G | -8.19E-03 | 1.48E-03 | 3.10E-08 | -4.57E-03 | 1.51E-03 | 2.40E-03 |
| rs7749708 | 6 | 153375907 | C | T | -9.98E-03 | 1.51E-03 | 3.70E-11 | -4.69E-03 | 1.54E-03 | 2.40E-03 |
| rs1222216 | 11 | 30346052 | C | T | 1.22E-02 | 1.64E-03 | 1.10E-13 | 5.09E-03 | 1.67E-03 | 2.40E-03 |
| rs35894137 | 8 | 43071838 | C | T | 1.42E-02 | 2.52E-03 | 1.60E-08 | 7.82E-03 | 2.57E-03 | 2.40E-03 |
| rs72652703 | 8 | 67209548 | T | C | 9.92E-03 | 1.58E-03 | 3.20E-10 | 4.87E-03 | 1.61E-03 | 2.50E-03 |
| rs12363672 | 11 | 55684028 | A | C | -2.38E-02 | 4.29E-03 | 2.80E-08 | -1.32E-02 | 4.38E-03 | 2.50E-03 |
| rs74929176 | 17 | 54905494 | C | T | 9.05E-03 | 1.65E-03 | 4.40E-08 | 5.04E-03 | 1.69E-03 | 2.80E-03 |
| rs17619860 | 8 | 87779603 | T | C | -1.04E-02 | 1.86E-03 | 2.20E-08 | -5.67E-03 | 1.90E-03 | 2.80E-03 |
| rs35221880 | 12 | 133301500 | T | C | 8.57E-03 | 1.55E-03 | 3.50E-08 | 4.72E-03 | 1.59E-03 | 2.90E-03 |
| rs11000993 | 10 | 76084111 | T | C | -1.38E-02 | 2.08E-03 | 3.10E-11 | -6.34E-03 | 2.12E-03 | 2.90E-03 |
| rs35809007 | 2 | 47019521 | G | A | 1.06E-02 | 1.43E-03 | 1.20E-13 | 4.34E-03 | 1.46E-03 | 3.00E-03 |
| rs9480184 | 6 | 155987788 | C | T | -9.86E-03 | 1.68E-03 | 4.90E-09 | -5.11E-03 | 1.72E-03 | 3.00E-03 |
| rs66460909 | 20 | 51195387 | G | A | 1.57E-02 | 1.75E-03 | 2.90E-19 | 5.27E-03 | 1.79E-03 | 3.10E-03 |
| rs7924036 | 10 | 65191645 | G | T | 9.63E-03 | 1.37E-03 | 2.20E-12 | 4.10E-03 | 1.40E-03 | 3.40E-03 |
| rs4575195 | 10 | 114765747 | C | A | 9.59E-03 | 1.48E-03 | 9.00E-11 | 4.43E-03 | 1.51E-03 | 3.40E-03 |
| rs4148155 | 4 | 89054667 | A | G | 1.43E-02 | 2.16E-03 | 2.90E-11 | 6.42E-03 | 2.21E-03 | 3.60E-03 |
| rs9843653 | 3 | 49920571 | T | C | -1.76E-02 | 1.37E-03 | 1.50E-37 | -4.07E-03 | 1.40E-03 | 3.70E-03 |
| rs7141912 | 14 | 35649431 | A | T | 1.18E-02 | 2.07E-03 | 1.10E-08 | 6.14E-03 | 2.11E-03 | 3.70E-03 |
| rs148137538 | 1 | 173399677 | A | G | 2.50E-02 | 4.58E-03 | 4.40E-08 | 1.35E-02 | 4.68E-03 | 3.80E-03 |
| rs45521740 | 19 | 2245622 | G | A | -1.80E-02 | 2.95E-03 | 1.00E-09 | -8.69E-03 | 3.01E-03 | 3.90E-03 |
| rs13218383 | 6 | 120173501 | C | G | 9.32E-03 | 1.45E-03 | 1.40E-10 | 4.22E-03 | 1.48E-03 | 4.40E-03 |
| rs3902951 | 14 | 69789755 | T | G | -1.05E-02 | 1.63E-03 | 1.10E-10 | -4.73E-03 | 1.67E-03 | 4.50E-03 |
| rs680071 | 11 | 103088414 | T | C | -1.17E-02 | 2.11E-03 | 2.80E-08 | -6.14E-03 | 2.16E-03 | 4.50E-03 |
| rs412243 | 16 | 339672 | T | C | 1.05E-02 | 1.42E-03 | 1.40E-13 | 4.08E-03 | 1.45E-03 | 4.70E-03 |
| rs10791113 | 11 | 130873165 | A | G | -8.57E-03 | 1.38E-03 | 4.60E-10 | -3.96E-03 | 1.40E-03 | 4.80E-03 |
| rs113132247 | 9 | 131026108 | G | A | -1.23E-02 | 1.91E-03 | 1.10E-10 | -5.50E-03 | 1.95E-03 | 4.80E-03 |
| rs651533 | 1 | 82375561 | T | A | 9.30E-03 | 1.59E-03 | 5.60E-09 | 4.56E-03 | 1.63E-03 | 5.20E-03 |
| rs34292685 | 11 | 64049021 | C | T | 1.27E-02 | 1.86E-03 | 7.90E-12 | 5.31E-03 | 1.90E-03 | 5.20E-03 |
| rs17029006 | 3 | 12329452 | C | T | 1.04E-02 | 1.55E-03 | 2.20E-11 | 4.42E-03 | 1.59E-03 | 5.40E-03 |
| rs7424120 | 2 | 59313974 | C | T | 1.42E-02 | 1.40E-03 | 4.00E-24 | 3.98E-03 | 1.43E-03 | 5.50E-03 |
| rs544957562 | 9 | 33978015 | A | T | 1.24E-02 | 2.02E-03 | 9.00E-10 | 5.74E-03 | 2.07E-03 | 5.50E-03 |
| rs1250597 | 10 | 81010250 | A | G | -8.54E-03 | 1.40E-03 | 1.00E-09 | 3.94E-03 | 1.43E-03 | 5.80E-03 |
| rs2083323 | 18 | 1856272 | G | A | -1.04E-02 | 1.80E-03 | 7.30E-09 | -5.03E-03 | 1.84E-03 | 6.20E-03 |
| rs719802 | 11 | 113234679 | T | C | 8.20E-03 | 1.41E-03 | 5.70E-09 | 3.91E-03 | 1.44E-03 | 6.50E-03 |
| rs1568488 | 3 | 153657951 | G | C | -1.10E-02 | 1.41E-03 | 4.40E-15 | -3.86E-03 | 1.44E-03 | 7.20E-03 |
| rs17716502 | 8 | 116659731 | C | T | 1.62E-02 | 1.71E-03 | 2.70E-21 | 4.71E-03 | 1.75E-03 | 7.20E-03 |
| rs57488047 | 15 | 79403002 | T | C | 9.86E-03 | 1.39E-03 | 1.10E-12 | 3.77E-03 | 1.41E-03 | 7.60E-03 |
| rs28839214 | 4 | 145313641 | G | T | -8.92E-03 | 1.41E-03 | 2.20E-10 | -3.83E-03 | 1.44E-03 | 7.60E-03 |
| rs2583410 | 4 | 102182199 | A | C | -1.34E-02 | 1.94E-03 | 5.30E-12 | -5.22E-03 | 1.98E-03 | 8.30E-03 |
| rs2343681 | 3 | 136535024 | G | A | -1.24E-02 | 1.68E-03 | 1.80E-13 | -4.51E-03 | 1.72E-03 | 8.60E-03 |
| rs12821683 | 12 | 58588964 | G | C | -1.12E-02 | 2.01E-03 | 2.80E-08 | -5.32E-03 | 2.06E-03 | 9.60E-03 |
| rs72917533 | 2 | 175238924 | T | C | 1.15E-02 | 1.76E-03 | 8.20E-11 | 4.66E-03 | 1.80E-03 | 9.70E-03 |
| rs76824303 | 3 | 62459819 | A | C | 1.52E-02 | 2.34E-03 | 9.20E-11 | 6.18E-03 | 2.40E-03 | 9.80E-03 |
| rs143662847 | 8 | 48804722 | C | T | 1.93E-02 | 3.53E-03 | 4.60E-08 | 9.32E-03 | 3.61E-03 | 9.90E-03 |
| rs1778830 | 1 | 156489974 | G | A | -9.91E-03 | 1.43E-03 | 3.80E-12 | -3.74E-03 | 1.46E-03 | 1.00E-02 |
| rs17770336 | 9 | 28414625 | C | T | -1.55E-02 | 1.46E-03 | 3.10E-26 | -3.84E-03 | 1.50E-03 | 1.00E-02 |
| rs61813324 | 1 | 156049877 | C | T | -1.83E-02 | 2.03E-03 | 1.80E-19 | -5.34E-03 | 2.07E-03 | 1.00E-02 |
| rs80082351 | 3 | 114415926 | A | G | 1.85E-02 | 2.85E-03 | 8.10E-11 | 7.49E-03 | 2.91E-03 | 1.00E-02 |
| rs7038966 | 9 | 73777777 | C | T | -9.48E-03 | 1.40E-03 | 1.40E-11 | -3.65E-03 | 1.43E-03 | 1.10E-02 |
| rs2247401 | 15 | 53156672 | G | A | -8.52E-03 | 1.56E-03 | 4.70E-08 | -4.08E-03 | 1.59E-03 | 1.10E-02 |
| rs329118 | 5 | 133861663 | C | T | 9.49E-03 | 1.39E-03 | 8.80E-12 | 3.53E-03 | 1.42E-03 | 1.30E-02 |
| rs7549358 | 1 | 115252609 | G | C | 8.83E-03 | 1.43E-03 | 6.50E-10 | 3.62E-03 | 1.46E-03 | 1.30E-02 |
| rs8124896 | 20 | 21385659 | T | C | -1.32E-02 | 2.28E-03 | 8.10E-09 | -5.77E-03 | 2.33E-03 | 1.30E-02 |
| rs2425856 | 20 | 44911954 | A | G | 8.42E-03 | 1.38E-03 | 1.10E-09 | 3.48E-03 | 1.41E-03 | 1.40E-02 |
| rs4792716 | 17 | 15943144 | A | G | -8.11E-03 | 1.38E-03 | 4.80E-09 | -3.47E-03 | 1.41E-03 | 1.40E-02 |
| rs9834519 | 3 | 156379637 | C | T | 1.56E-02 | 2.54E-03 | 8.40E-10 | 6.36E-03 | 2.60E-03 | 1.40E-02 |
| rs6752979 | 2 | 81741750 | G | A | -8.96E-03 | 1.47E-03 | 1.10E-09 | -3.67E-03 | 1.50E-03 | 1.50E-02 |
| rs1451533 | 2 | 105466005 | G | A | -1.10E-02 | 1.55E-03 | 1.30E-12 | -3.84E-03 | 1.58E-03 | 1.50E-02 |
| rs79686965 | 11 | 46020909 | A | G | -3.10E-02 | 5.56E-03 | 2.30E-08 | -1.38E-02 | 5.68E-03 | 1.50E-02 |
| rs61746970 | 19 | 51132746 | G | A | -2.13E-02 | 3.62E-03 | 3.80E-09 | -8.88E-03 | 3.69E-03 | 1.60E-02 |
| rs215634 | 7 | 32369148 | A | G | 1.04E-02 | 1.41E-03 | 1.40E-13 | 3.44E-03 | 1.44E-03 | 1.70E-02 |
| rs12031634 | 1 | 34584393 | G | A | 8.81E-03 | 1.50E-03 | 4.80E-09 | 3.60E-03 | 1.54E-03 | 1.90E-02 |
| rs2658797 | 11 | 93212254 | C | T | 7.79E-03 | 1.37E-03 | 1.30E-08 | 3.25E-03 | 1.40E-03 | 2.00E-02 |
| rs529200 | 3 | 173114305 | A | G | -1.03E-02 | 1.37E-03 | 5.80E-14 | -3.24E-03 | 1.40E-03 | 2.10E-02 |
| rs6583310 | 3 | 196170985 | G | C | -8.78E-03 | 1.38E-03 | 2.20E-10 | -3.26E-03 | 1.42E-03 | 2.10E-02 |
| rs61986330 | 14 | 73314450 | C | A | 8.73E-03 | 1.54E-03 | 1.40E-08 | 3.63E-03 | 1.57E-03 | 2.10E-02 |
| rs537508 | 4 | 171042158 | G | C | -7.77E-03 | 1.40E-03 | 2.60E-08 | -3.26E-03 | 1.43E-03 | 2.20E-02 |
| rs556992087 | 12 | 124500725 | T | C | -1.06E-02 | 1.67E-03 | 2.00E-10 | -3.91E-03 | 1.70E-03 | 2.20E-02 |
| rs73078357 | 3 | 48695834 | T | C | 1.19E-02 | 2.09E-03 | 1.30E-08 | 4.89E-03 | 2.14E-03 | 2.20E-02 |
| rs9673839 | 16 | 76895693 | A | G | -8.30E-03 | 1.38E-03 | 1.70E-09 | -3.20E-03 | 1.41E-03 | 2.30E-02 |
| rs788163 | 2 | 172931559 | A | C | -1.01E-02 | 1.54E-03 | 5.90E-11 | -3.57E-03 | 1.57E-03 | 2.30E-02 |
| rs62379271 | 5 | 105870033 | T | G | -7.81E-03 | 1.39E-03 | 2.00E-08 | -3.22E-03 | 1.42E-03 | 2.40E-02 |
| rs2725371 | 8 | 30854033 | A | G | 1.00E-02 | 1.50E-03 | 2.00E-11 | -3.46E-03 | 1.53E-03 | 2.40E-02 |
| rs6069625 | 20 | 54747469 | A | G | 8.73E-03 | 1.56E-03 | 2.00E-08 | 3.58E-03 | 1.59E-03 | 2.40E-02 |
| rs12213441 | 6 | 143208838 | C | T | -1.05E-02 | 1.67E-03 | 3.30E-10 | -3.85E-03 | 1.71E-03 | 2.40E-02 |
| rs76702514 | 1 | 195148296 | C | G | 1.01E-02 | 1.69E-03 | 2.00E-09 | 3.91E-03 | 1.73E-03 | 2.40E-02 |
| rs11218510 | 11 | 121922587 | G | A | 8.10E-03 | 1.40E-03 | 7.80E-09 | 3.22E-03 | 1.43E-03 | 2.50E-02 |
| rs1598121 | 3 | 82694710 | A | G | -8.96E-03 | 1.42E-03 | 3.00E-10 | -3.23E-03 | 1.46E-03 | 2.70E-02 |
| rs10457469 | 6 | 126083658 | G | A | -7.63E-03 | 1.37E-03 | 2.60E-08 | -3.08E-03 | 1.40E-03 | 2.80E-02 |
| rs9579775 | 13 | 20616557 | A | C | -1.46E-02 | 2.08E-03 | 2.10E-12 | -4.68E-03 | 2.13E-03 | 2.80E-02 |
| rs6511826 | 19 | 12706991 | G | A | 1.40E-02 | 2.46E-03 | 1.30E-08 | 5.51E-03 | 2.51E-03 | 2.80E-02 |
| rs7925100 | 11 | 118941596 | G | A | -9.17E-03 | 1.40E-03 | 6.30E-11 | -3.13E-03 | 1.43E-03 | 2.90E-02 |
| rs347551 | 5 | 119389031 | C | G | -8.89E-03 | 1.39E-03 | 1.80E-10 | -3.09E-03 | 1.43E-03 | 3.00E-02 |
| rs1899689 | 7 | 121964349 | C | T | -7.74E-03 | 1.41E-03 | 3.70E-08 | -3.10E-03 | 1.44E-03 | 3.10E-02 |
| rs11150462 | 16 | 82451679 | T | A | 8.44E-03 | 1.42E-03 | 3.10E-09 | 3.10E-03 | 1.46E-03 | 3.30E-02 |
| rs6597653 | 9 | 133788465 | G | C | -8.53E-03 | 1.41E-03 | 1.30E-09 | -3.05E-03 | 1.44E-03 | 3.40E-02 |
| rs115778101 | 1 | 78198554 | T | C | 1.78E-02 | 3.22E-03 | 3.40E-08 | 6.92E-03 | 3.29E-03 | 3.50E-02 |
| rs67913249 | 5 | 43204126 | C | G | 9.35E-03 | 1.45E-03 | 1.20E-10 | 3.07E-03 | 1.48E-03 | 3.80E-02 |
| rs7511698 | 1 | 25015638 | C | T | 8.21E-03 | 1.49E-03 | 3.20E-08 | 3.14E-03 | 1.52E-03 | 3.80E-02 |
| rs9852062 | 3 | 45373442 | T | A | 8.17E-03 | 1.38E-03 | 3.50E-09 | 2.89E-03 | 1.41E-03 | 4.10E-02 |
| rs2114210 | 8 | 95595162 | G | A | -9.86E-03 | 1.45E-03 | 1.20E-11 | -3.02E-03 | 1.49E-03 | 4.20E-02 |
| rs6030803 | 20 | 41986507 | T | C | 1.29E-02 | 2.07E-03 | 4.50E-10 | 4.30E-03 | 2.12E-03 | 4.20E-02 |
| rs1409158 | 1 | 119538890 | C | T | 9.23E-03 | 1.61E-03 | 1.00E-08 | 3.33E-03 | 1.65E-03 | 4.30E-02 |
| rs13174863 | 5 | 139080745 | A | G | -1.36E-02 | 1.94E-03 | 2.40E-12 | -4.03E-03 | 1.99E-03 | 4.30E-02 |
| rs10174253 | 2 | 181323160 | A | C | -1.12E-02 | 1.53E-03 | 2.50E-13 | -3.08E-03 | 1.57E-03 | 4.90E-02 |
| rs61754230 | 12 | 72179446 | C | T | -2.74E-02 | 4.93E-03 | 2.80E-08 | -9.81E-03 | 5.03E-03 | 5.10E-02 |
| rs7086898 | 10 | 104386152 | A | G | -1.39E-02 | 2.53E-03 | 4.30E-08 | -4.98E-03 | 2.59E-03 | 5.40E-02 |
| rs1631026 | 2 | 26953850 | C | T | -1.07E-02 | 1.37E-03 | 5.90E-15 | -2.69E-03 | 1.40E-03 | 5.50E-02 |
| rs1964926 | 21 | 42653121 | A | G | -8.23E-03 | 1.44E-03 | 1.10E-08 | -2.82E-03 | 1.47E-03 | 5.50E-02 |
| rs112852122 | 20 | 47498117 | G | A | 1.34E-02 | 1.91E-03 | 2.70E-12 | 3.75E-03 | 1.95E-03 | 5.50E-02 |
| rs6761463 | 2 | 50201547 | G | C | 1.26E-02 | 1.86E-03 | 1.30E-11 | 3.61E-03 | 1.90E-03 | 5.70E-02 |
| rs11047138 | 12 | 24019853 | C | G | -1.44E-02 | 2.63E-03 | 4.60E-08 | -5.08E-03 | 2.68E-03 | 5.80E-02 |
| rs16940823 | 18 | 22137319 | C | A | 1.08E-02 | 1.79E-03 | 1.60E-09 | 3.42E-03 | 1.82E-03 | 6.10E-02 |
| rs66674732 | 13 | 62721160 | G | A | -8.16E-03 | 1.41E-03 | 7.20E-09 | -2.68E-03 | 1.44E-03 | 6.30E-02 |
| rs1381010 | 4 | 112677085 | G | A | 8.24E-03 | 1.49E-03 | 3.00E-08 | 2.81E-03 | 1.52E-03 | 6.40E-02 |
| rs369461388 | 14 | 40104718 | G | C | -1.26E-02 | 2.01E-03 | 3.60E-10 | -3.80E-03 | 2.05E-03 | 6.40E-02 |
| rs1369159 | 15 | 66360842 | C | T | 7.88E-03 | 1.40E-03 | 1.70E-08 | 2.63E-03 | 1.43E-03 | 6.50E-02 |
| rs4989244 | 9 | 102100348 | G | A | 7.63E-03 | 1.39E-03 | 3.60E-08 | 2.61E-03 | 1.42E-03 | 6.60E-02 |
| rs12149660 | 16 | 70309237 | G | A | 1.62E-02 | 2.16E-03 | 6.20E-14 | 4.01E-03 | 2.21E-03 | 6.90E-02 |
| rs2035806 | 10 | 133984916 | G | A | 9.55E-03 | 1.39E-03 | 5.70E-12 | 2.56E-03 | 1.42E-03 | 7.00E-02 |
| rs429358 | 19 | 45411941 | T | C | 1.61E-02 | 1.90E-03 | 2.90E-17 | -3.45E-03 | 1.94E-03 | 7.50E-02 |
| rs7230240 | 18 | 42597978 | C | T | 9.40E-03 | 1.50E-03 | 4.20E-10 | 2.73E-03 | 1.54E-03 | 7.60E-02 |
| rs34542489 | 17 | 51917844 | A | C | 7.78E-03 | 1.40E-03 | 2.80E-08 | 2.52E-03 | 1.43E-03 | 7.80E-02 |
| rs201475383 | 20 | 26273991 | G | A | 2.40E-02 | 3.92E-03 | 8.80E-10 | 6.98E-03 | 4.00E-03 | 8.10E-02 |
| rs2056477 | 7 | 2079744 | G | C | 1.16E-02 | 1.64E-03 | 1.50E-12 | 2.90E-03 | 1.67E-03 | 8.30E-02 |
| rs6075658 | 20 | 2094078 | T | C | 8.49E-03 | 1.38E-03 | 6.80E-10 | 2.43E-03 | 1.40E-03 | 8.40E-02 |
| rs368540015 | 7 | 74292165 | A | G | -1.82E-02 | 3.25E-03 | 2.20E-08 | -5.73E-03 | 3.32E-03 | 8.40E-02 |
| rs142503704 | 5 | 92622421 | G | A | -2.63E-02 | 4.68E-03 | 1.90E-08 | -8.13E-03 | 4.78E-03 | 8.90E-02 |
| rs6050446 | 20 | 25195509 | A | G | -2.64E-02 | 3.89E-03 | 1.20E-11 | -6.70E-03 | 3.98E-03 | 9.20E-02 |
| rs1987960 | 20 | 30649834 | T | C | -1.89E-02 | 3.31E-03 | 1.10E-08 | -5.63E-03 | 3.38E-03 | 9.60E-02 |
| rs2660241 | 16 | 4940023 | T | C | -8.13E-03 | 1.43E-03 | 1.20E-08 | -2.41E-03 | 1.46E-03 | 9.90E-02 |
| rs3737992 | 1 | 33234128 | G | A | 1.37E-02 | 1.83E-03 | 5.30E-14 | 3.07E-03 | 1.87E-03 | 9.90E-02 |
| rs6823268 | 4 | 145982563 | A | G | -8.18E-03 | 1.42E-03 | 8.20E-09 | -2.36E-03 | 1.45E-03 | 1.00E-01 |
| rs75957461 | 19 | 11166163 | C | T | -1.77E-02 | 3.05E-03 | 6.60E-09 | -5.11E-03 | 3.11E-03 | 1.00E-01 |
| rs7182917 | 15 | 52080803 | T | C | 8.74E-03 | 1.39E-03 | 2.80E-10 | 2.25E-03 | 1.41E-03 | 1.10E-01 |
| rs9615723 | 22 | 48386670 | C | T | 7.71E-03 | 1.40E-03 | 3.80E-08 | 2.26E-03 | 1.43E-03 | 1.10E-01 |
| rs10960276 | 9 | 11819686 | C | A | 8.09E-03 | 1.43E-03 | 1.60E-08 | -2.34E-03 | 1.46E-03 | 1.10E-01 |
| rs4911382 | 20 | 32553095 | C | T | -8.32E-03 | 1.40E-03 | 2.60E-09 | -2.23E-03 | 1.43E-03 | 1.20E-01 |
| rs868784 | 11 | 43944388 | G | A | 7.87E-03 | 1.42E-03 | 2.70E-08 | 2.28E-03 | 1.45E-03 | 1.20E-01 |
| rs10823826 | 10 | 53649431 | C | T | -8.98E-03 | 1.59E-03 | 1.60E-08 | -2.56E-03 | 1.62E-03 | 1.20E-01 |
| rs12885251 | 14 | 99670791 | G | A | 7.63E-03 | 1.39E-03 | 4.10E-08 | 2.13E-03 | 1.42E-03 | 1.30E-01 |
| rs10505836 | 12 | 19288508 | A | C | -1.21E-02 | 1.99E-03 | 1.10E-09 | -3.08E-03 | 2.03E-03 | 1.30E-01 |
| rs77560793 | 1 | 175001179 | G | A | 2.46E-02 | 3.99E-03 | 7.80E-10 | 6.25E-03 | 4.08E-03 | 1.30E-01 |
| rs7570446 | 2 | 193801010 | C | A | -7.80E-03 | 1.37E-03 | 1.30E-08 | 2.06E-03 | 1.40E-03 | 1.40E-01 |
| rs12477088 | 2 | 67841326 | T | C | 1.05E-02 | 1.39E-03 | 4.70E-14 | 2.08E-03 | 1.42E-03 | 1.40E-01 |
| rs2542615 | 10 | 131128952 | C | T | 8.44E-03 | 1.46E-03 | 7.30E-09 | 2.21E-03 | 1.49E-03 | 1.40E-01 |
| rs1799507 | 12 | 16427314 | G | A | -1.08E-02 | 1.96E-03 | 3.10E-08 | -2.95E-03 | 2.00E-03 | 1.40E-01 |
| rs945211 | 1 | 32191798 | G | C | -8.49E-03 | 1.41E-03 | 1.60E-09 | -2.06E-03 | 1.44E-03 | 1.50E-01 |
| rs2516726 | 16 | 2095065 | T | C | 9.91E-03 | 1.64E-03 | 1.70E-09 | 2.42E-03 | 1.68E-03 | 1.50E-01 |
| rs72753485 | 9 | 96673230 | G | C | -1.56E-02 | 2.50E-03 | 4.30E-10 | -3.68E-03 | 2.55E-03 | 1.50E-01 |
| rs1373349 | 18 | 63282992 | C | T | 9.81E-03 | 1.48E-03 | 3.50E-11 | 2.13E-03 | 1.51E-03 | 1.60E-01 |
| rs79675564 | 2 | 211286896 | C | A | -1.51E-02 | 2.55E-03 | 3.40E-09 | -3.67E-03 | 2.61E-03 | 1.60E-01 |
| rs12927792 | 16 | 9713194 | C | T | -8.67E-03 | 1.40E-03 | 5.20E-10 | -1.97E-03 | 1.43E-03 | 1.70E-01 |
| rs7264802 | 20 | 62692440 | A | G | -9.81E-03 | 1.59E-03 | 7.20E-10 | -2.22E-03 | 1.62E-03 | 1.70E-01 |
| rs12477385 | 2 | 166144850 | G | T | 9.24E-03 | 1.64E-03 | 1.80E-08 | 2.28E-03 | 1.68E-03 | 1.70E-01 |
| rs10774018 | 12 | 2157925 | G | C | -9.48E-03 | 1.66E-03 | 1.10E-08 | -2.32E-03 | 1.69E-03 | 1.70E-01 |
| rs12705894 | 7 | 113351252 | G | A | 7.80E-03 | 1.38E-03 | 1.60E-08 | 1.90E-03 | 1.41E-03 | 1.80E-01 |
| rs187067151 | 20 | 29539588 | G | T | 2.67E-02 | 3.98E-03 | 2.00E-11 | 5.44E-03 | 4.06E-03 | 1.80E-01 |
| rs403694 | 21 | 46567625 | C | T | -1.22E-02 | 1.38E-03 | 1.30E-18 | -1.86E-03 | 1.41E-03 | 1.90E-01 |
| rs7893571 | 10 | 16750129 | G | T | -1.02E-02 | 1.46E-03 | 2.90E-12 | -1.93E-03 | 1.49E-03 | 1.90E-01 |
| rs36007635 | 6 | 163009335 | G | A | 1.37E-02 | 1.99E-03 | 5.60E-12 | 2.65E-03 | 2.03E-03 | 1.90E-01 |
| rs7548936 | 1 | 91207757 | G | C | -8.48E-03 | 1.42E-03 | 2.10E-09 | 1.85E-03 | 1.45E-03 | 2.00E-01 |
| rs151252883 | 2 | 228998026 | T | G | -9.22E-03 | 1.44E-03 | 1.60E-10 | -1.91E-03 | 1.47E-03 | 2.00E-01 |
| rs183315407 | 1 | 46201427 | G | A | 2.64E-02 | 3.49E-03 | 4.30E-14 | 4.54E-03 | 3.57E-03 | 2.00E-01 |
| rs2074686 | 7 | 100800635 | G | A | 8.28E-03 | 1.39E-03 | 2.90E-09 | 1.79E-03 | 1.42E-03 | 2.10E-01 |
| rs34373881 | 3 | 20432033 | G | A | 8.83E-03 | 1.54E-03 | 9.00E-09 | 1.98E-03 | 1.57E-03 | 2.10E-01 |
| rs12644329 | 4 | 143634746 | G | A | 7.91E-03 | 1.42E-03 | 2.50E-08 | 1.78E-03 | 1.45E-03 | 2.20E-01 |
| rs7518221 | 1 | 225561346 | T | C | 7.95E-03 | 1.43E-03 | 2.80E-08 | 1.80E-03 | 1.46E-03 | 2.20E-01 |
| rs4759228 | 12 | 56508409 | G | C | 1.16E-02 | 1.51E-03 | 1.30E-14 | 1.89E-03 | 1.54E-03 | 2.20E-01 |
| rs575840515 | 1 | 80796649 | A | G | -9.01E-03 | 1.52E-03 | 3.20E-09 | -1.90E-03 | 1.56E-03 | 2.20E-01 |
| rs10973159 | 9 | 36992547 | G | T | -8.05E-03 | 1.41E-03 | 1.20E-08 | 1.74E-03 | 1.44E-03 | 2.30E-01 |
| rs10760277 | 9 | 126093999 | C | T | -8.87E-03 | 1.41E-03 | 3.60E-10 | -1.68E-03 | 1.44E-03 | 2.40E-01 |
| rs61903695 | 11 | 89922417 | A | G | -1.13E-02 | 1.58E-03 | 6.60E-13 | -1.87E-03 | 1.61E-03 | 2.40E-01 |
| rs78517245 | 3 | 42587865 | T | C | -3.44E-02 | 5.85E-03 | 4.10E-09 | -6.98E-03 | 5.98E-03 | 2.40E-01 |
| rs10805383 | 5 | 63034606 | G | A | -1.05E-02 | 1.37E-03 | 2.60E-14 | -1.57E-03 | 1.40E-03 | 2.60E-01 |
| rs9522180 | 13 | 111970212 | C | T | 9.24E-03 | 1.38E-03 | 2.40E-11 | 1.55E-03 | 1.41E-03 | 2.70E-01 |
| rs1324110 | 6 | 93913200 | G | C | 7.77E-03 | 1.38E-03 | 2.00E-08 | 1.57E-03 | 1.41E-03 | 2.70E-01 |
| rs72910629 | 6 | 69761994 | A | G | -1.33E-02 | 2.01E-03 | 4.30E-11 | 2.26E-03 | 2.05E-03 | 2.70E-01 |
| rs78369934 | 17 | 61739101 | T | C | 1.99E-02 | 3.05E-03 | 6.00E-11 | -3.44E-03 | 3.11E-03 | 2.70E-01 |
| rs78886584 | 1 | 16859325 | A | G | -8.49E-03 | 1.38E-03 | 8.70E-10 | -1.52E-03 | 1.42E-03 | 2.80E-01 |
| rs409696 | 2 | 147900651 | G | A | 1.14E-02 | 1.39E-03 | 1.80E-16 | 1.55E-03 | 1.42E-03 | 2.80E-01 |
| rs12357890 | 10 | 99762693 | A | G | -1.22E-02 | 1.39E-03 | 1.60E-18 | -1.53E-03 | 1.42E-03 | 2.80E-01 |
| rs4806814 | 19 | 1860147 | G | A | 1.27E-02 | 1.90E-03 | 3.10E-11 | -2.10E-03 | 1.94E-03 | 2.80E-01 |
| rs78508049 | 1 | 210344884 | T | C | -1.11E-02 | 1.75E-03 | 2.10E-10 | -1.88E-03 | 1.79E-03 | 2.90E-01 |
| rs55931203 | 17 | 65854602 | C | T | -1.26E-02 | 1.78E-03 | 1.60E-12 | -1.92E-03 | 1.82E-03 | 2.90E-01 |
| rs149457 | 5 | 107438057 | C | T | 1.50E-02 | 1.83E-03 | 2.10E-16 | 1.98E-03 | 1.87E-03 | 2.90E-01 |
| rs11708540 | 3 | 70593081 | G | A | -1.06E-02 | 1.89E-03 | 2.00E-08 | 2.07E-03 | 1.93E-03 | 2.90E-01 |
| rs567230078 | 6 | 43588227 | T | A | 2.51E-02 | 4.25E-03 | 3.70E-09 | 4.57E-03 | 4.34E-03 | 2.90E-01 |
| rs13292699 | 9 | 15910044 | A | C | 1.25E-02 | 1.39E-03 | 1.50E-19 | -1.47E-03 | 1.42E-03 | 3.00E-01 |
| rs7913496 | 10 | 10257277 | C | T | 1.00E-02 | 1.79E-03 | 2.00E-08 | -1.88E-03 | 1.83E-03 | 3.00E-01 |
| rs76520838 | 15 | 47916618 | C | T | -2.32E-02 | 3.87E-03 | 1.90E-09 | -4.12E-03 | 3.95E-03 | 3.00E-01 |
| rs2396625 | 7 | 113028634 | T | A | 9.94E-03 | 1.40E-03 | 1.10E-12 | 1.45E-03 | 1.43E-03 | 3.10E-01 |
| rs3806114 | 6 | 20482335 | G | A | 8.79E-03 | 1.47E-03 | 2.30E-09 | 1.51E-03 | 1.50E-03 | 3.10E-01 |
| rs116195355 | 1 | 39941508 | C | A | 2.60E-02 | 3.96E-03 | 5.90E-11 | 4.09E-03 | 4.05E-03 | 3.10E-01 |
| rs698147 | 5 | 3513485 | A | G | 8.78E-03 | 1.38E-03 | 1.80E-10 | 1.41E-03 | 1.41E-03 | 3.20E-01 |
| rs1840660 | 7 | 114352615 | G | A | -9.95E-03 | 1.41E-03 | 2.00E-12 | 1.44E-03 | 1.45E-03 | 3.20E-01 |
| rs2866720 | 7 | 70106310 | C | T | -9.13E-03 | 1.42E-03 | 1.20E-10 | -1.46E-03 | 1.45E-03 | 3.20E-01 |
| rs2269610 | 6 | 33289935 | G | C | -1.28E-02 | 1.77E-03 | 5.10E-13 | 1.78E-03 | 1.81E-03 | 3.20E-01 |
| rs4962725 | 10 | 126733321 | T | C | -9.97E-03 | 1.39E-03 | 6.60E-13 | -1.39E-03 | 1.42E-03 | 3.30E-01 |
| rs2837398 | 21 | 41427168 | A | C | -8.20E-03 | 1.40E-03 | 4.80E-09 | 1.39E-03 | 1.43E-03 | 3.30E-01 |
| rs262956 | 3 | 183486117 | T | G | 9.12E-03 | 1.44E-03 | 2.20E-10 | 1.43E-03 | 1.47E-03 | 3.30E-01 |
| rs6530737 | 8 | 14095763 | A | G | 9.02E-03 | 1.44E-03 | 3.50E-10 | 1.40E-03 | 1.47E-03 | 3.40E-01 |
| rs11079849 | 17 | 47090785 | C | T | 1.22E-02 | 1.46E-03 | 6.40E-17 | 1.42E-03 | 1.49E-03 | 3.40E-01 |
| rs35957544 | 8 | 73440371 | G | T | 1.27E-02 | 1.39E-03 | 7.40E-20 | 1.33E-03 | 1.42E-03 | 3.50E-01 |
| rs28726372 | 1 | 84353839 | T | C | -9.08E-03 | 1.48E-03 | 9.40E-10 | -1.43E-03 | 1.52E-03 | 3.50E-01 |
| rs9421249 | 10 | 118623322 | C | T | -1.04E-02 | 1.56E-03 | 2.50E-11 | 1.49E-03 | 1.60E-03 | 3.50E-01 |
| rs61217499 | 12 | 108417780 | G | C | 1.28E-02 | 1.67E-03 | 2.30E-14 | 1.60E-03 | 1.71E-03 | 3.50E-01 |
| rs57654548 | 3 | 125196904 | C | A | 1.16E-02 | 2.12E-03 | 4.20E-08 | 2.02E-03 | 2.17E-03 | 3.50E-01 |
| rs11642387 | 16 | 6753239 | A | G | 1.29E-02 | 2.29E-03 | 2.10E-08 | -2.20E-03 | 2.34E-03 | 3.50E-01 |
| rs4836133 | 5 | 124332103 | C | A | -8.36E-03 | 1.41E-03 | 3.10E-09 | 2.31E-03 | 2.47E-03 | 3.50E-01 |
| rs2164300 | 4 | 67813017 | C | T | 7.55E-03 | 1.38E-03 | 4.20E-08 | -1.26E-03 | 1.41E-03 | 3.70E-01 |
| rs1454687 | 3 | 94038085 | C | G | 1.23E-02 | 1.37E-03 | 3.00E-19 | 1.24E-03 | 1.40E-03 | 3.80E-01 |
| rs114728753 | 3 | 78483402 | A | C | -8.95E-03 | 1.47E-03 | 1.00E-09 | -1.32E-03 | 1.50E-03 | 3.80E-01 |
| rs396354 | 2 | 86850022 | T | C | 1.04E-02 | 1.52E-03 | 8.00E-12 | 1.36E-03 | 1.55E-03 | 3.80E-01 |
| rs236660 | 7 | 75050086 | T | C | -1.45E-02 | 1.44E-03 | 9.10E-24 | -1.27E-03 | 1.47E-03 | 3.90E-01 |
| rs12427047 | 12 | 90213070 | C | T | 1.09E-02 | 1.60E-03 | 8.90E-12 | 1.40E-03 | 1.63E-03 | 3.90E-01 |
| rs1547205 | 9 | 98815145 | G | C | 1.32E-02 | 2.32E-03 | 1.30E-08 | 2.02E-03 | 2.37E-03 | 3.90E-01 |
| rs1458156 | 12 | 41887940 | C | T | -9.29E-03 | 1.37E-03 | 1.30E-11 | -1.19E-03 | 1.40E-03 | 4.00E-01 |
| rs1704190 | 2 | 200760629 | G | A | -8.33E-03 | 1.42E-03 | 4.50E-09 | -1.23E-03 | 1.45E-03 | 4.00E-01 |
| rs3753639 | 1 | 154986091 | T | C | -1.15E-02 | 1.60E-03 | 7.30E-13 | -1.38E-03 | 1.64E-03 | 4.00E-01 |
| rs7539903 | 1 | 209208033 | T | A | 7.69E-03 | 1.41E-03 | 4.60E-08 | 1.18E-03 | 1.44E-03 | 4.10E-01 |
| rs7321285 | 13 | 54319327 | A | C | 1.13E-02 | 1.72E-03 | 4.10E-11 | 1.46E-03 | 1.76E-03 | 4.10E-01 |
| rs1411432 | 9 | 16728532 | A | C | -1.24E-02 | 1.77E-03 | 2.00E-12 | 1.49E-03 | 1.81E-03 | 4.10E-01 |
| rs10756555 | 9 | 14459089 | G | A | 1.03E-02 | 1.39E-03 | 1.60E-13 | 1.13E-03 | 1.42E-03 | 4.20E-01 |
| rs4419475 | 4 | 96150044 | A | T | -8.10E-03 | 1.39E-03 | 6.50E-09 | 1.16E-03 | 1.43E-03 | 4.20E-01 |
| rs9888533 | 13 | 107854612 | C | T | -7.65E-03 | 1.40E-03 | 4.50E-08 | -1.15E-03 | 1.43E-03 | 4.20E-01 |
| rs7175642 | 15 | 59450079 | T | G | 8.59E-03 | 1.47E-03 | 5.20E-09 | 1.22E-03 | 1.50E-03 | 4.20E-01 |
| rs111584879 | 15 | 66678173 | T | C | 8.79E-03 | 1.61E-03 | 4.80E-08 | 1.33E-03 | 1.64E-03 | 4.20E-01 |
| rs12630209 | 3 | 156881392 | T | G | -8.75E-03 | 1.58E-03 | 3.00E-08 | -1.27E-03 | 1.61E-03 | 4.30E-01 |
| rs12376870 | 9 | 117890567 | G | A | 8.87E-03 | 1.62E-03 | 4.10E-08 | 1.28E-03 | 1.65E-03 | 4.40E-01 |
| rs11513729 | 12 | 112273499 | C | T | 7.99E-03 | 1.41E-03 | 1.50E-08 | 1.07E-03 | 1.44E-03 | 4.60E-01 |
| rs11134512 | 5 | 167847460 | T | G | 8.24E-03 | 1.47E-03 | 1.90E-08 | 1.12E-03 | 1.50E-03 | 4.60E-01 |
| rs7928320 | 11 | 116942753 | C | G | -1.73E-02 | 2.97E-03 | 6.10E-09 | -2.23E-03 | 3.04E-03 | 4.60E-01 |
| rs473837 | 8 | 60906881 | G | T | 8.63E-03 | 1.44E-03 | 1.80E-09 | -1.01E-03 | 1.47E-03 | 4.90E-01 |
| rs11765062 | 7 | 54417515 | T | C | 7.52E-03 | 1.37E-03 | 4.60E-08 | 9.37E-04 | 1.40E-03 | 5.00E-01 |
| rs12281009 | 11 | 117032959 | A | G | -1.79E-02 | 2.95E-03 | 1.30E-09 | -2.02E-03 | 3.01E-03 | 5.00E-01 |
| rs6950388 | 7 | 1270699 | G | A | -9.45E-03 | 1.70E-03 | 2.60E-08 | 1.15E-03 | 1.74E-03 | 5.10E-01 |
| rs12538826 | 7 | 99030228 | T | C | 1.68E-02 | 2.15E-03 | 7.20E-15 | -1.43E-03 | 2.20E-03 | 5.10E-01 |
| rs7433076 | 3 | 90234502 | T | A | 9.52E-03 | 1.38E-03 | 5.10E-12 | -9.17E-04 | 1.41E-03 | 5.20E-01 |
| rs1977658 | 1 | 107607037 | T | G | 8.10E-03 | 1.45E-03 | 2.50E-08 | -9.64E-04 | 1.49E-03 | 5.20E-01 |
| rs217672 | 14 | 62361021 | A | C | -1.18E-02 | 1.55E-03 | 2.30E-14 | 1.02E-03 | 1.58E-03 | 5.20E-01 |
| rs1040046 | 6 | 83473573 | C | A | -1.13E-02 | 1.92E-03 | 4.60E-09 | -1.25E-03 | 1.96E-03 | 5.20E-01 |
| rs35390852 | 4 | 143067054 | G | A | -1.14E-02 | 2.09E-03 | 4.80E-08 | -1.33E-03 | 2.13E-03 | 5.30E-01 |
| rs4663213 | 2 | 236807893 | G | A | 9.46E-03 | 1.66E-03 | 1.20E-08 | 1.04E-03 | 1.70E-03 | 5.40E-01 |
| rs73213484 | 4 | 28489339 | A | T | 1.46E-02 | 1.97E-03 | 1.10E-13 | 1.24E-03 | 2.01E-03 | 5.40E-01 |
| rs6507054 | 18 | 31248323 | T | C | -9.28E-03 | 1.39E-03 | 2.70E-11 | 8.60E-04 | 1.42E-03 | 5.50E-01 |
| rs9462670 | 6 | 41014309 | G | C | -9.42E-03 | 1.63E-03 | 7.10E-09 | -9.86E-04 | 1.66E-03 | 5.50E-01 |
| rs13275517 | 8 | 143364521 | T | C | -8.15E-03 | 1.39E-03 | 4.10E-09 | -8.14E-04 | 1.42E-03 | 5.70E-01 |
| rs11782074 | 8 | 142617096 | G | T | -9.72E-03 | 1.43E-03 | 1.20E-11 | 8.24E-04 | 1.46E-03 | 5.70E-01 |
| rs10160769 | 11 | 76474827 | G | C | 9.18E-03 | 1.68E-03 | 4.60E-08 | -9.87E-04 | 1.72E-03 | 5.70E-01 |
| rs113079574 | 4 | 147354089 | C | T | 9.65E-03 | 1.75E-03 | 3.30E-08 | -9.93E-04 | 1.78E-03 | 5.80E-01 |
| rs10935143 | 3 | 134665159 | G | A | 8.40E-03 | 1.38E-03 | 1.10E-09 | 7.63E-04 | 1.41E-03 | 5.90E-01 |
| rs1905616 | 8 | 93235675 | G | A | 8.20E-03 | 1.46E-03 | 1.80E-08 | 8.02E-04 | 1.49E-03 | 5.90E-01 |
| rs12072739 | 1 | 98315893 | A | G | -1.16E-02 | 1.64E-03 | 1.80E-12 | -8.95E-04 | 1.68E-03 | 5.90E-01 |
| rs12037905 | 1 | 219628036 | C | T | 7.93E-03 | 1.39E-03 | 1.10E-08 | 7.49E-04 | 1.42E-03 | 6.00E-01 |
| rs8134638 | 21 | 40644170 | T | C | -7.80E-03 | 1.42E-03 | 4.00E-08 | 7.68E-04 | 1.45E-03 | 6.00E-01 |
| rs12033257 | 1 | 112318484 | A | G | 9.80E-03 | 1.42E-03 | 4.90E-12 | -7.57E-04 | 1.45E-03 | 6.00E-01 |
| rs2237402 | 7 | 39449768 | G | A | 9.23E-03 | 1.45E-03 | 1.80E-10 | 7.69E-04 | 1.48E-03 | 6.00E-01 |
| rs61985411 | 14 | 41336102 | T | A | -1.65E-02 | 2.76E-03 | 2.10E-09 | -1.47E-03 | 2.82E-03 | 6.00E-01 |
| rs809955 | 4 | 140874760 | G | A | 1.01E-02 | 1.42E-03 | 1.40E-12 | -7.30E-04 | 1.46E-03 | 6.20E-01 |
| rs8192675 | 3 | 170724883 | T | C | -1.17E-02 | 1.51E-03 | 8.70E-15 | -7.69E-04 | 1.54E-03 | 6.20E-01 |
| rs7331420 | 13 | 99236471 | G | A | 9.14E-03 | 1.53E-03 | 2.10E-09 | -7.63E-04 | 1.56E-03 | 6.20E-01 |
| rs1017529 | 17 | 27912415 | C | A | -1.06E-02 | 1.84E-03 | 7.70E-09 | -9.30E-04 | 1.88E-03 | 6.20E-01 |
| rs2576135 | 13 | 54691442 | T | A | 1.32E-02 | 2.39E-03 | 3.50E-08 | 1.21E-03 | 2.44E-03 | 6.20E-01 |
| rs8011566 | 14 | 42939471 | T | A | -8.73E-03 | 1.39E-03 | 3.50E-10 | -6.89E-04 | 1.42E-03 | 6.30E-01 |
| rs39674 | 16 | 9413210 | C | G | -8.71E-03 | 1.50E-03 | 6.00E-09 | -7.29E-04 | 1.53E-03 | 6.30E-01 |
| rs2433733 | 2 | 230816703 | G | A | 1.05E-02 | 1.46E-03 | 7.30E-13 | 6.91E-04 | 1.50E-03 | 6.40E-01 |
| rs36131051 | 3 | 107888841 | T | G | 1.08E-02 | 1.71E-03 | 2.90E-10 | 8.28E-04 | 1.75E-03 | 6.40E-01 |
| rs112380819 | 3 | 9498519 | G | A | -1.37E-02 | 2.26E-03 | 1.30E-09 | -1.05E-03 | 2.31E-03 | 6.50E-01 |
| rs147730268 | 12 | 123024476 | G | T | 2.28E-02 | 2.49E-03 | 4.00E-20 | -1.15E-03 | 2.54E-03 | 6.50E-01 |
| rs62259692 | 3 | 51847709 | G | A | -1.78E-02 | 2.93E-03 | 1.40E-09 | 1.34E-03 | 3.00E-03 | 6.50E-01 |
| rs13186637 | 5 | 153108558 | T | C | 8.68E-03 | 1.44E-03 | 1.70E-09 | -6.46E-04 | 1.47E-03 | 6.60E-01 |
| rs9395520 | 6 | 13183523 | C | T | 9.76E-03 | 1.49E-03 | 5.90E-11 | 6.65E-04 | 1.52E-03 | 6.60E-01 |
| rs10927006 | 1 | 243557659 | T | C | 1.20E-02 | 1.95E-03 | 8.30E-10 | 8.84E-04 | 2.00E-03 | 6.60E-01 |
| rs10204994 | 2 | 35443726 | G | A | 1.01E-02 | 1.63E-03 | 5.30E-10 | 7.02E-04 | 1.66E-03 | 6.70E-01 |
| rs10499014 | 6 | 97947755 | C | G | 1.00E-02 | 1.56E-03 | 1.10E-10 | -6.65E-04 | 1.59E-03 | 6.80E-01 |
| rs533493779 | 2 | 104447054 | A | T | -8.69E-03 | 1.42E-03 | 1.10E-09 | -5.76E-04 | 1.46E-03 | 6.90E-01 |
| rs4660586 | 1 | 42407229 | C | T | 8.96E-03 | 1.56E-03 | 1.00E-08 | -6.28E-04 | 1.60E-03 | 6.90E-01 |
| rs7020196 | 9 | 12289527 | C | T | 7.79E-03 | 1.41E-03 | 3.60E-08 | 5.52E-04 | 1.44E-03 | 7.00E-01 |
| rs1865341 | 9 | 8845911 | C | T | -9.00E-03 | 1.62E-03 | 2.50E-08 | -6.28E-04 | 1.65E-03 | 7.00E-01 |
| rs12681792 | 8 | 62054463 | C | A | -9.56E-03 | 1.75E-03 | 4.30E-08 | -6.92E-04 | 1.78E-03 | 7.00E-01 |
| rs59227842 | 11 | 43692423 | A | G | -1.52E-02 | 1.49E-03 | 1.80E-24 | -5.69E-04 | 1.53E-03 | 7.10E-01 |
| rs13427822 | 2 | 213414265 | A | G | 9.45E-03 | 1.56E-03 | 1.30E-09 | -5.84E-04 | 1.59E-03 | 7.10E-01 |
| rs270689 | 6 | 104790532 | A | T | -1.01E-02 | 1.72E-03 | 4.20E-09 | -6.49E-04 | 1.76E-03 | 7.10E-01 |
| rs6478538 | 9 | 124627012 | A | G | 8.81E-03 | 1.47E-03 | 1.90E-09 | -5.27E-04 | 1.50E-03 | 7.30E-01 |
| rs2616143 | 8 | 20632022 | G | A | 8.85E-03 | 1.47E-03 | 1.90E-09 | 5.15E-04 | 1.51E-03 | 7.30E-01 |
| rs72976986 | 19 | 4050424 | G | A | 1.39E-02 | 1.77E-03 | 4.40E-15 | 6.31E-04 | 1.80E-03 | 7.30E-01 |
| rs9515446 | 13 | 112217108 | A | G | -9.69E-03 | 1.38E-03 | 2.20E-12 | -4.59E-04 | 1.41E-03 | 7.40E-01 |
| rs4759073 | 12 | 54653258 | G | A | 9.35E-03 | 1.39E-03 | 1.90E-11 | 4.35E-04 | 1.42E-03 | 7.60E-01 |
| rs10169594 | 2 | 41637688 | T | C | -7.88E-03 | 1.43E-03 | 3.30E-08 | -4.55E-04 | 1.46E-03 | 7.60E-01 |
| rs2744801 | 1 | 41155486 | C | T | 7.95E-03 | 1.45E-03 | 4.30E-08 | 4.44E-04 | 1.48E-03 | 7.60E-01 |
| rs149778057 | 13 | 31007805 | A | C | 9.05E-03 | 1.53E-03 | 3.80E-09 | 4.37E-04 | 1.57E-03 | 7.80E-01 |
| rs1320251 | 17 | 21264396 | C | T | 1.24E-02 | 1.38E-03 | 3.20E-19 | -3.69E-04 | 1.41E-03 | 7.90E-01 |
| rs7255223 | 19 | 32824310 | C | A | 8.97E-03 | 1.56E-03 | 8.50E-09 | 4.27E-04 | 1.59E-03 | 7.90E-01 |
| rs60497719 | 19 | 33971746 | G | A | -9.67E-03 | 1.58E-03 | 1.00E-09 | -4.30E-04 | 1.62E-03 | 7.90E-01 |
| rs4077093 | 12 | 51593616 | T | G | 9.69E-03 | 1.68E-03 | 7.90E-09 | 4.66E-04 | 1.72E-03 | 7.90E-01 |
| rs11691869 | 2 | 100805996 | C | A | 1.12E-02 | 1.43E-03 | 3.50E-15 | -3.65E-04 | 1.46E-03 | 8.00E-01 |
| rs10779835 | 1 | 230299949 | T | C | 8.01E-03 | 1.41E-03 | 1.20E-08 | 3.37E-04 | 1.44E-03 | 8.10E-01 |
| rs12971645 | 19 | 45807945 | G | A | 8.59E-03 | 1.54E-03 | 2.60E-08 | 3.43E-04 | 1.58E-03 | 8.30E-01 |
| rs2237025 | 4 | 55541879 | T | C | 1.02E-02 | 1.39E-03 | 1.80E-13 | -2.88E-04 | 1.42E-03 | 8.40E-01 |
| rs9529148 | 13 | 67419495 | G | A | -8.00E-03 | 1.42E-03 | 1.90E-08 | -2.79E-04 | 1.45E-03 | 8.50E-01 |
| rs3759584 | 14 | 103990799 | T | C | 9.74E-03 | 1.43E-03 | 1.10E-11 | 2.76E-04 | 1.46E-03 | 8.50E-01 |
| rs7601895 | 2 | 55281901 | C | G | 1.03E-02 | 1.49E-03 | 4.10E-12 | 2.78E-04 | 1.52E-03 | 8.50E-01 |
| rs7498044 | 15 | 92573639 | G | A | 9.95E-03 | 1.68E-03 | 3.20E-09 | -3.19E-04 | 1.72E-03 | 8.50E-01 |
| rs4916229 | 1 | 171443368 | C | G | -1.51E-02 | 2.33E-03 | 8.90E-11 | 3.77E-04 | 2.39E-03 | 8.70E-01 |
| rs35852935 | 4 | 17991522 | A | C | -2.19E-02 | 3.74E-03 | 4.90E-09 | 6.39E-04 | 3.82E-03 | 8.70E-01 |
| rs13104584 | 4 | 80811227 | G | A | -8.64E-03 | 1.40E-03 | 6.20E-10 | -1.93E-04 | 1.43E-03 | 8.90E-01 |
| rs4500770 | 16 | 74658430 | A | T | 7.82E-03 | 1.43E-03 | 4.30E-08 | 1.96E-04 | 1.46E-03 | 8.90E-01 |
| rs71495049 | 10 | 34014435 | G | A | -1.71E-02 | 2.48E-03 | 5.70E-12 | -3.46E-04 | 2.53E-03 | 8.90E-01 |
| rs1229984 | 4 | 100239319 | T | C | -2.30E-02 | 4.16E-03 | 3.20E-08 | 5.90E-04 | 4.26E-03 | 8.90E-01 |
| rs113706999 | 3 | 44159156 | T | A | -2.88E-02 | 4.73E-03 | 1.10E-09 | -6.03E-04 | 4.84E-03 | 9.00E-01 |
| rs58351927 | 17 | 5297038 | A | G | -1.01E-02 | 1.50E-03 | 1.20E-11 | -1.67E-04 | 1.53E-03 | 9.10E-01 |
| rs113569731 | 3 | 47093206 | C | A | -1.52E-02 | 2.41E-03 | 3.10E-10 | -2.93E-04 | 2.47E-03 | 9.10E-01 |
| rs61971082 | 13 | 86494667 | T | G | -1.01E-02 | 1.52E-03 | 3.00E-11 | 1.62E-04 | 1.56E-03 | 9.20E-01 |
| rs9477762 | 6 | 18507853 | A | T | -2.02E-02 | 3.05E-03 | 3.80E-11 | 2.72E-04 | 3.12E-03 | 9.30E-01 |
| rs10185199 | 2 | 40282202 | G | A | 1.00E-02 | 1.56E-03 | 1.60E-10 | 1.00E-04 | 1.60E-03 | 9.50E-01 |
| rs4658403 | 1 | 243832560 | C | T | 1.36E-02 | 1.84E-03 | 1.20E-13 | 9.35E-05 | 1.88E-03 | 9.60E-01 |
| rs80236973 | 3 | 188001014 | C | T | 1.22E-02 | 2.01E-03 | 1.40E-09 | 9.83E-05 | 2.05E-03 | 9.60E-01 |
| rs4425224 | 3 | 56249398 | C | A | 1.24E-02 | 2.24E-03 | 2.70E-08 | -1.19E-04 | 2.29E-03 | 9.60E-01 |
| rs181617194 | 12 | 122011598 | T | C | 2.27E-02 | 3.76E-03 | 1.70E-09 | 1.22E-04 | 3.84E-03 | 9.70E-01 |
| rs28408562 | 15 | 60917079 | C | G | -7.84E-03 | 1.38E-03 | 1.30E-08 | 4.07E-05 | 1.41E-03 | 9.80E-01 |
| rs7206608 | 16 | 82872628 | C | G | -9.79E-03 | 1.47E-03 | 2.60E-11 | 4.55E-05 | 1.50E-03 | 9.80E-01 |
| rs12253527 | 10 | 21819824 | G | A | -1.36E-02 | 1.47E-03 | 2.00E-20 | -9.61E-06 | 1.50E-03 | 9.90E-01 |

**Table S2F.** The genetic variants strongly associated with adulthood adiposity at genome wide significance and not childhood adiposity (exclude child SNPs at P ≤ 5×10^-8^) in females.

| SNP | Chromosome | Base position | Effect allele | Other allele | Beta (Age 10) | SE (Age 10) | P (Age 10) | Beta (Adult) | SE (Adult) | P (Adult) |
| --- | --- | --- | --- | --- | --- | --- | --- | --- | --- | --- |
| rs74892851 | 1 | 1563789 | C | A | 1.22E-04 | 2.03E-03 | 9.70E-01 | 1.16E-02 | 2.06E-03 | 1.90E-08 |
| rs78886584 | 1 | 16859325 | A | G | -2.12E-03 | 1.96E-03 | 3.20E-01 | -1.33E-02 | 1.99E-03 | 2.60E-11 |
| rs72660086 | 1 | 39571992 | T | G | -3.11E-03 | 2.37E-03 | 1.90E-01 | -1.33E-02 | 2.41E-03 | 3.20E-08 |
| rs36017365 | 1 | 47682889 | C | CA | 6.64E-03 | 2.08E-03 | 1.70E-03 | 1.29E-02 | 2.12E-03 | 9.30E-10 |
| rs749593242 | 1 | 50224384 | C | CT | 2.39E-03 | 2.08E-03 | 1.70E-01 | 1.28E-02 | 2.11E-03 | 1.30E-09 |
| rs140681455 | 1 | 78444764 | C | CGGCCG | -1.09E-02 | 2.98E-03 | 2.30E-04 | -2.54E-02 | 3.02E-03 | 4.20E-17 |
| rs56951135 | 1 | 91211018 | A | ATTT | 3.91E-04 | 2.04E-03 | 8.30E-01 | -1.43E-02 | 2.07E-03 | 4.90E-12 |
| rs653958 | 1 | 96884006 | A | G | -5.19E-03 | 2.00E-03 | 1.00E-02 | -1.16E-02 | 2.04E-03 | 1.10E-08 |
| rs75641275 | 1 | 98327133 | A | C | 2.06E-03 | 2.76E-03 | 5.90E-01 | -1.85E-02 | 2.81E-03 | 4.70E-11 |
| rs12033257 | 1 | 112318484 | A | G | -3.09E-04 | 2.00E-03 | 6.80E-01 | 1.22E-02 | 2.04E-03 | 1.90E-09 |
| rs3753639 | 1 | 154986091 | T | C | 7.79E-04 | 2.26E-03 | 8.90E-01 | -1.26E-02 | 2.30E-03 | 3.70E-08 |
| rs61813324 | 1 | 156049877 | C | T | -4.74E-03 | 2.87E-03 | 8.10E-02 | -2.09E-02 | 2.91E-03 | 7.60E-13 |
| rs815163 | 1 | 190294726 | T | C | 9.19E-03 | 1.95E-03 | 2.60E-06 | 1.32E-02 | 1.98E-03 | 2.40E-11 |
| rs2678204 | 1 | 201800511 | T | G | -5.56E-03 | 2.04E-03 | 6.10E-03 | -1.34E-02 | 2.07E-03 | 1.20E-10 |
| rs2994320 | 1 | 243641247 | A | G | -2.73E-04 | 2.45E-03 | 7.90E-01 | 1.66E-02 | 2.49E-03 | 2.40E-11 |
| rs34606703 | 2 | 47014522 | G | A | 5.03E-03 | 2.02E-03 | 1.80E-02 | 1.17E-02 | 2.06E-03 | 1.10E-08 |
| rs13420048 | 2 | 50751414 | C | A | 6.99E-04 | 2.01E-03 | 5.80E-01 | 1.20E-02 | 2.04E-03 | 4.40E-09 |
| rs6545468 | 2 | 55277641 | C | G | 2.20E-03 | 1.97E-03 | 2.10E-01 | 1.20E-02 | 2.00E-03 | 2.20E-09 |
| rs4671328 | 2 | 58935282 | T | G | 1.65E-03 | 1.96E-03 | 3.20E-01 | 1.37E-02 | 1.99E-03 | 6.50E-12 |
| rs13416992 | 2 | 59298298 | A | C | 4.40E-03 | 1.98E-03 | 4.30E-02 | 1.48E-02 | 2.01E-03 | 1.90E-13 |
| rs10192894 | 2 | 62838936 | A | G | 1.93E-03 | 1.95E-03 | 3.00E-01 | -1.10E-02 | 1.98E-03 | 2.50E-08 |
| rs12477088 | 2 | 67841326 | T | C | 1.12E-03 | 1.96E-03 | 6.20E-01 | 1.19E-02 | 1.99E-03 | 2.70E-09 |
| rs11691869 | 2 | 100805996 | C | A | 1.03E-03 | 2.02E-03 | 6.30E-01 | 1.55E-02 | 2.05E-03 | 3.30E-14 |
| rs113607259 | 2 | 104343481 | G | GTA | 2.80E-03 | 1.95E-03 | 1.40E-01 | 1.18E-02 | 1.99E-03 | 2.60E-09 |
| rs7602120 | 2 | 144033069 | C | T | -2.71E-03 | 1.95E-03 | 1.50E-01 | -1.34E-02 | 1.98E-03 | 1.20E-11 |
| rs1083472 | 2 | 147873492 | C | G | -8.32E-04 | 1.99E-03 | 8.00E-01 | 1.13E-02 | 2.02E-03 | 2.10E-08 |
| rs758369774 | 2 | 181562177 | AT | A | -5.55E-03 | 2.01E-03 | 4.50E-03 | -1.23E-02 | 2.04E-03 | 1.50E-09 |
| rs4482463 | 2 | 205375909 | C | A | 6.82E-03 | 3.64E-03 | 3.20E-02 | 2.18E-02 | 3.70E-03 | 3.70E-09 |
| rs4673553 | 2 | 211608379 | T | G | -2.48E-03 | 1.95E-03 | 2.20E-01 | -1.17E-02 | 1.98E-03 | 3.20E-09 |
| rs573105257 | 2 | 230822932 | A | AT | 1.19E-03 | 2.08E-03 | 5.20E-01 | 1.18E-02 | 2.11E-03 | 2.20E-08 |
| rs62242071 | 3 | 20590313 | G | T | 2.93E-03 | 2.08E-03 | 1.70E-01 | 1.18E-02 | 2.11E-03 | 2.30E-08 |
| rs113706999 | 3 | 44159156 | T | A | -5.99E-03 | 6.65E-03 | 3.10E-01 | -3.87E-02 | 6.76E-03 | 1.00E-08 |
| rs9843653 | 3 | 49920571 | T | C | -3.97E-03 | 1.94E-03 | 4.30E-02 | -1.76E-02 | 1.97E-03 | 4.20E-19 |
| rs6774533 | 3 | 62471086 | C | T | -5.62E-03 | 2.16E-03 | 1.40E-02 | -1.22E-02 | 2.19E-03 | 2.80E-08 |
| rs34234711 | 3 | 82709447 | G | T | -3.99E-03 | 2.01E-03 | 3.90E-02 | -1.21E-02 | 2.04E-03 | 3.20E-09 |
| rs1454687 | 3 | 94038085 | C | G | 1.89E-03 | 1.93E-03 | 4.00E-01 | 1.23E-02 | 1.97E-03 | 4.10E-10 |
| rs9811252 | 3 | 128293392 | C | T | 1.04E-03 | 2.03E-03 | 8.10E-01 | 1.14E-02 | 2.07E-03 | 3.90E-08 |
| rs13063449 | 3 | 131770166 | T | G | -1.06E-02 | 2.08E-03 | 1.10E-07 | -1.36E-02 | 2.11E-03 | 1.30E-10 |
| rs13081671 | 3 | 135876549 | C | T | -6.69E-04 | 2.18E-03 | 7.20E-01 | -1.35E-02 | 2.22E-03 | 1.20E-09 |
| rs529200 | 3 | 173114305 | A | G | -3.02E-03 | 1.94E-03 | 1.90E-01 | -1.16E-02 | 1.97E-03 | 4.00E-09 |
| rs73052033 | 3 | 185828465 | T | C | 6.67E-03 | 2.50E-03 | 4.50E-03 | 1.72E-02 | 2.54E-03 | 1.10E-11 |
| rs61218008 | 3 | 194881130 | A | G | 5.35E-03 | 2.17E-03 | 1.30E-02 | 1.21E-02 | 2.21E-03 | 4.70E-08 |
| rs148928878 | 4 | 18548257 | G | GGTGT | -4.57E-03 | 1.95E-03 | 1.30E-02 | -1.12E-02 | 1.98E-03 | 1.60E-08 |
| rs9684942 | 4 | 20233035 | G | A | -5.57E-03 | 2.74E-03 | 4.60E-02 | -1.53E-02 | 2.79E-03 | 3.80E-08 |
| rs34811474 | 4 | 25408838 | G | A | 8.54E-03 | 2.30E-03 | 2.00E-04 | 1.97E-02 | 2.34E-03 | 3.20E-17 |
| rs73213484 | 4 | 28489339 | A | T | 2.48E-03 | 2.78E-03 | 4.00E-01 | 1.89E-02 | 2.83E-03 | 2.30E-11 |
| rs148712344 | 4 | 55476318 | G | T | 7.04E-03 | 5.02E-03 | 1.50E-01 | 2.98E-02 | 5.10E-03 | 5.10E-09 |
| rs925422 | 4 | 60254101 | T | G | 5.74E-03 | 2.22E-03 | 1.50E-02 | 1.25E-02 | 2.26E-03 | 2.70E-08 |
| rs1603179 | 4 | 67805347 | A | C | -3.60E-03 | 2.03E-03 | 9.20E-02 | 1.14E-02 | 2.06E-03 | 2.90E-08 |
| rs11098965 | 4 | 80888040 | C | T | 6.51E-04 | 2.22E-03 | 7.00E-01 | 1.36E-02 | 2.26E-03 | 1.60E-09 |
| rs2199936 | 4 | 89045331 | A | G | -6.25E-03 | 3.05E-03 | 5.20E-02 | -1.83E-02 | 3.10E-03 | 3.30E-09 |
| rs182851732 | 4 | 90684766 | A | G | 1.31E-02 | 1.18E-02 | 2.90E-01 | 6.96E-02 | 1.20E-02 | 7.70E-09 |
| rs57590313 | 4 | 113323430 | C | A | -9.11E-03 | 2.52E-03 | 2.30E-04 | -1.41E-02 | 2.57E-03 | 4.10E-08 |
| rs769668 | 4 | 140858717 | T | C | 1.97E-04 | 2.04E-03 | 9.70E-01 | 1.40E-02 | 2.08E-03 | 1.80E-11 |
| rs35390852 | 4 | 143067054 | G | A | -2.05E-03 | 2.94E-03 | 4.10E-01 | -1.67E-02 | 2.99E-03 | 2.40E-08 |
| rs828550 | 5 | 3539923 | C | T | -1.56E-03 | 2.04E-03 | 4.30E-01 | -1.17E-02 | 2.07E-03 | 1.50E-08 |
| rs796338714 | 5 | 43190861 | T | TA | -8.14E-03 | 2.03E-03 | 4.50E-05 | -1.17E-02 | 2.07E-03 | 1.40E-08 |
| rs55908499 | 5 | 63020950 | G | GA | -2.55E-03 | 1.98E-03 | 2.00E-01 | -1.41E-02 | 2.01E-03 | 2.20E-12 |
| rs34341 | 5 | 74934009 | A | T | -7.43E-03 | 1.96E-03 | 2.70E-04 | -2.00E-02 | 1.99E-03 | 1.10E-23 |
| rs59893724 | 5 | 80830788 | A | G | 6.30E-03 | 2.25E-03 | 4.30E-03 | 1.28E-02 | 2.29E-03 | 2.40E-08 |
| rs7442885 | 5 | 87682877 | C | G | 1.15E-02 | 2.36E-03 | 2.00E-06 | 1.70E-02 | 2.40E-03 | 1.50E-12 |
| rs35843836 | 5 | 88798726 | A | T | -5.26E-03 | 2.01E-03 | 4.90E-03 | -1.18E-02 | 2.04E-03 | 7.60E-09 |
| rs191621046 | 5 | 92547517 | G | A | -5.60E-03 | 5.06E-03 | 2.20E-01 | -2.98E-02 | 5.15E-03 | 7.10E-09 |
| rs10623997 | 5 | 107478679 | T | TATAATA | -3.07E-04 | 2.34E-03 | 9.10E-01 | 1.80E-02 | 2.38E-03 | 3.60E-14 |
| rs1366334 | 5 | 122683163 | C | G | 6.11E-03 | 2.13E-03 | 1.90E-03 | 1.18E-02 | 2.17E-03 | 4.70E-08 |
| rs71579590 | 5 | 139070398 | G | C | -3.73E-03 | 2.74E-03 | 1.80E-01 | -1.88E-02 | 2.79E-03 | 1.70E-11 |
| rs251353 | 5 | 140228164 | C | A | 2.52E-03 | 2.04E-03 | 1.50E-01 | 1.19E-02 | 2.07E-03 | 1.10E-08 |
| rs12658841 | 5 | 153106013 | C | G | -1.18E-03 | 1.95E-03 | 7.80E-01 | 1.17E-02 | 1.98E-03 | 3.80E-09 |
| rs11134679 | 5 | 170623391 | A | G | -8.42E-03 | 2.09E-03 | 1.20E-04 | -1.31E-02 | 2.13E-03 | 8.00E-10 |
| rs9395520 | 6 | 13183523 | C | T | 1.09E-03 | 2.10E-03 | 5.80E-01 | 1.25E-02 | 2.14E-03 | 4.60E-09 |
| rs2103646 | 6 | 34571195 | G | A | -8.58E-03 | 2.06E-03 | 1.50E-05 | -2.23E-02 | 2.10E-03 | 1.90E-26 |
| rs77253887 | 6 | 35626932 | G | C | 1.41E-03 | 5.40E-03 | 7.90E-01 | -3.03E-02 | 5.49E-03 | 3.50E-08 |
| rs34298980 | 6 | 40409243 | T | C | 7.36E-03 | 2.04E-03 | 2.50E-04 | 1.41E-02 | 2.07E-03 | 8.80E-12 |
| rs567230078 | 6 | 43588227 | T | A | 8.43E-03 | 5.99E-03 | 2.40E-01 | 3.40E-02 | 6.09E-03 | 2.50E-08 |
| rs760571548 | 6 | 98583487 | CAT | C | -9.25E-03 | 1.94E-03 | 4.40E-06 | 1.13E-02 | 1.98E-03 | 1.20E-08 |
| rs2253310 | 6 | 108888593 | C | G | -8.11E-03 | 2.00E-03 | 5.60E-05 | -1.18E-02 | 2.03E-03 | 6.10E-09 |
| rs9387640 | 6 | 119508871 | C | T | 3.06E-03 | 2.01E-03 | 1.30E-01 | 1.18E-02 | 2.05E-03 | 9.20E-09 |
| rs73046311 | 7 | 1854159 | C | G | 1.70E-03 | 2.65E-03 | 5.40E-01 | 1.57E-02 | 2.69E-03 | 5.70E-09 |
| rs2866720 | 7 | 70106310 | C | T | -1.60E-03 | 2.00E-03 | 3.10E-01 | -1.18E-02 | 2.04E-03 | 7.00E-09 |
| rs236660 | 7 | 75050086 | T | C | -2.89E-03 | 2.03E-03 | 1.50E-01 | -1.49E-02 | 2.06E-03 | 5.40E-13 |
| rs369428586 | 7 | 99134799 | CA | C | -2.52E-03 | 2.73E-03 | 4.00E-01 | 1.56E-02 | 2.78E-03 | 2.00E-08 |
| rs12375196 | 7 | 103416541 | C | A | -1.02E-02 | 1.97E-03 | 1.20E-07 | -1.17E-02 | 2.01E-03 | 5.50E-09 |
| rs2396625 | 7 | 113028634 | T | A | 3.73E-04 | 1.97E-03 | 7.10E-01 | 1.18E-02 | 2.00E-03 | 3.40E-09 |
| rs1840661 | 7 | 114352682 | T | A | 3.18E-03 | 1.97E-03 | 1.50E-01 | -1.22E-02 | 2.00E-03 | 1.20E-09 |
| rs7853 | 8 | 8890814 | A | G | -7.99E-03 | 1.95E-03 | 4.80E-05 | -1.20E-02 | 1.98E-03 | 1.30E-09 |
| rs6601415 | 8 | 9976805 | C | A | -6.18E-03 | 1.95E-03 | 1.20E-03 | -1.11E-02 | 1.98E-03 | 2.30E-08 |
| rs6557829 | 8 | 21973970 | C | A | 1.90E-03 | 1.97E-03 | 3.10E-01 | -1.16E-02 | 2.00E-03 | 6.90E-09 |
| rs117176448 | 8 | 27261138 | C | G | -4.48E-03 | 3.28E-03 | 1.80E-01 | -1.87E-02 | 3.34E-03 | 2.00E-08 |
| rs10957605 | 8 | 73433886 | C | T | 8.68E-04 | 2.08E-03 | 8.10E-01 | 1.58E-02 | 2.12E-03 | 9.80E-14 |
| rs17716502 | 8 | 116659731 | C | T | 2.81E-03 | 2.42E-03 | 1.90E-01 | 1.79E-02 | 2.46E-03 | 3.50E-13 |
| rs4740442 | 9 | 10153245 | C | T | -4.50E-03 | 2.15E-03 | 3.10E-02 | -1.21E-02 | 2.18E-03 | 2.60E-08 |
| rs13292699 | 9 | 15910044 | A | C | -2.66E-03 | 1.96E-03 | 1.90E-01 | 1.50E-02 | 1.99E-03 | 4.30E-14 |
| rs10962552 | 9 | 16723742 | C | T | -1.73E-03 | 2.61E-03 | 5.20E-01 | -1.46E-02 | 2.65E-03 | 3.60E-08 |
| rs377741138 | 9 | 28412183 | GAAAA | G | -2.70E-03 | 2.05E-03 | 2.20E-01 | -1.61E-02 | 2.09E-03 | 1.10E-14 |
| rs779728965 | 9 | 103100173 | ATC | A | -8.42E-03 | 2.08E-03 | 7.00E-05 | -1.25E-02 | 2.11E-03 | 3.30E-09 |
| rs6478538 | 9 | 124627012 | A | G | 6.79E-04 | 2.07E-03 | 5.70E-01 | 1.15E-02 | 2.10E-03 | 4.60E-08 |
| rs3003578 | 9 | 130994179 | C | T | 2.25E-03 | 1.96E-03 | 2.10E-01 | 1.12E-02 | 2.00E-03 | 1.80E-08 |
| rs1270799 | 10 | 21907423 | T | G | -2.76E-03 | 2.11E-03 | 1.70E-01 | -1.65E-02 | 2.14E-03 | 1.20E-14 |
| rs113585475 | 10 | 33985434 | C | T | 1.87E-03 | 3.27E-03 | 4.70E-01 | 2.20E-02 | 3.32E-03 | 3.60E-11 |
| rs3125326 | 10 | 63053788 | A | C | -4.42E-03 | 2.00E-03 | 2.50E-02 | -1.11E-02 | 2.04E-03 | 4.70E-08 |
| rs7090758 | 10 | 65335315 | T | C | -5.72E-03 | 1.94E-03 | 3.70E-03 | -1.29E-02 | 1.97E-03 | 5.70E-11 |
| rs562044398 | 10 | 76101122 | C | CA | -6.63E-03 | 2.91E-03 | 1.70E-02 | -1.68E-02 | 2.96E-03 | 1.50E-08 |
| rs1250535 | 10 | 81016112 | C | G | 2.24E-03 | 2.08E-03 | 2.20E-01 | -1.19E-02 | 2.11E-03 | 1.80E-08 |
| rs12357890 | 10 | 99762693 | A | G | 6.41E-04 | 1.96E-03 | 7.90E-01 | -1.48E-02 | 1.99E-03 | 1.20E-13 |
| rs10510025 | 10 | 118650996 | C | T | 2.28E-04 | 2.25E-03 | 7.90E-01 | -1.33E-02 | 2.29E-03 | 6.90E-09 |
| rs141090474 | 10 | 126729168 | TAC | T | 3.21E-03 | 2.00E-03 | 1.00E-01 | 1.15E-02 | 2.03E-03 | 1.40E-08 |
| rs201102222 | 10 | 134005569 | A | AT | 2.02E-03 | 1.96E-03 | 3.60E-01 | 1.15E-02 | 1.99E-03 | 7.60E-09 |
| rs201233808 | 11 | 867599 | C | CCAT | 4.92E-03 | 2.16E-03 | 3.80E-02 | 1.24E-02 | 2.20E-03 | 2.00E-08 |
| rs7950166 | 11 | 8642218 | C | T | -6.99E-03 | 2.02E-03 | 4.00E-04 | -1.44E-02 | 2.06E-03 | 3.00E-12 |
| rs11022766 | 11 | 13348249 | T | G | 6.71E-03 | 2.03E-03 | 1.10E-03 | 1.38E-02 | 2.07E-03 | 2.50E-11 |
| rs60572790 | 11 | 43692383 | T | C | -1.62E-03 | 2.09E-03 | 5.20E-01 | -1.65E-02 | 2.12E-03 | 7.20E-15 |
| rs34292685 | 11 | 64049021 | C | T | 4.46E-03 | 2.63E-03 | 8.00E-02 | 1.80E-02 | 2.67E-03 | 1.60E-11 |
| rs11218510 | 11 | 121922587 | G | A | 2.91E-03 | 1.98E-03 | 2.00E-01 | 1.12E-02 | 2.01E-03 | 2.90E-08 |
| rs199569565 | 11 | 130749351 | TAG | T | -6.34E-03 | 1.94E-03 | 1.70E-03 | -1.11E-02 | 1.98E-03 | 1.70E-08 |
| rs2512884 | 11 | 131467856 | C | A | -1.01E-02 | 1.94E-03 | 8.50E-08 | -1.24E-02 | 1.98E-03 | 3.30E-10 |
| rs11223204 | 11 | 132652554 | A | G | -4.88E-03 | 1.96E-03 | 1.50E-02 | -1.14E-02 | 1.99E-03 | 1.10E-08 |
| rs12364470 | 11 | 134601012 | T | G | -1.35E-02 | 2.61E-03 | 1.90E-07 | -1.47E-02 | 2.66E-03 | 2.90E-08 |
| rs7976757 | 12 | 19207948 | T | C | -3.22E-03 | 2.57E-03 | 2.70E-01 | -1.52E-02 | 2.62E-03 | 6.10E-09 |
| rs2292238 | 12 | 56493822 | A | C | 1.21E-03 | 1.97E-03 | 4.40E-01 | 1.22E-02 | 2.01E-03 | 1.30E-09 |
| rs770082 | 12 | 89776485 | G | A | -4.23E-03 | 1.96E-03 | 4.40E-02 | -1.34E-02 | 1.99E-03 | 2.10E-11 |
| rs34697864 | 12 | 103680717 | C | CA | 7.51E-03 | 2.32E-03 | 1.30E-03 | 1.38E-02 | 2.36E-03 | 5.60E-09 |
| rs61217499 | 12 | 108417780 | G | C | 1.78E-03 | 2.35E-03 | 5.30E-01 | 1.48E-02 | 2.40E-03 | 5.70E-10 |
| rs10849900 | 12 | 110974890 | T | C | 4.16E-03 | 2.09E-03 | 4.30E-02 | 1.20E-02 | 2.13E-03 | 1.60E-08 |
| rs111828690 | 12 | 117576767 | C | T | -1.01E-03 | 2.35E-03 | 6.50E-01 | -1.37E-02 | 2.39E-03 | 1.00E-08 |
| rs181617194 | 12 | 122011598 | T | C | -4.02E-03 | 5.30E-03 | 4.50E-01 | 3.17E-02 | 5.39E-03 | 4.20E-09 |
| rs3803005 | 12 | 123110654 | T | C | -2.78E-03 | 2.18E-03 | 1.90E-01 | -1.68E-02 | 2.22E-03 | 3.60E-14 |
| rs9579775 | 13 | 20616557 | A | C | -5.99E-03 | 2.94E-03 | 3.70E-02 | -1.92E-02 | 2.99E-03 | 1.40E-10 |
| rs1933440 | 13 | 28676971 | A | C | -6.27E-03 | 2.64E-03 | 1.10E-02 | -1.53E-02 | 2.69E-03 | 1.20E-08 |
| rs11415560 | 13 | 32996332 | C | CA | -4.94E-03 | 2.03E-03 | 1.80E-02 | -1.16E-02 | 2.07E-03 | 2.10E-08 |
| rs776543236 | 13 | 67348551 | ATGGAG | A | 6.04E-04 | 2.29E-03 | 7.90E-01 | -1.44E-02 | 2.33E-03 | 6.50E-10 |
| rs116394958 | 13 | 86477072 | C | T | -9.39E-05 | 2.17E-03 | 9.90E-01 | -1.26E-02 | 2.21E-03 | 1.40E-08 |
| rs7331420 | 13 | 99236471 | G | A | -2.37E-04 | 2.15E-03 | 8.40E-01 | 1.20E-02 | 2.19E-03 | 4.80E-08 |
| rs9522180 | 13 | 111970212 | C | T | 2.22E-03 | 1.95E-03 | 3.00E-01 | 1.08E-02 | 1.98E-03 | 4.40E-08 |
| rs367552155 | 14 | 29721943 | CA | C | 1.78E-03 | 2.29E-03 | 4.90E-01 | 1.44E-02 | 2.33E-03 | 7.40E-10 |
| rs75104958 | 14 | 33269269 | A | C | -7.87E-03 | 2.41E-03 | 1.10E-03 | -1.57E-02 | 2.45E-03 | 1.30E-10 |
| rs10142359 | 14 | 73884540 | A | G | -2.28E-03 | 1.94E-03 | 2.20E-01 | -1.11E-02 | 1.97E-03 | 1.70E-08 |
| rs8022132 | 14 | 79955864 | A | T | -4.02E-03 | 2.11E-03 | 3.80E-02 | -1.46E-02 | 2.15E-03 | 1.00E-11 |
| rs6575340 | 14 | 94023972 | G | A | -4.92E-03 | 2.02E-03 | 7.20E-03 | -1.46E-02 | 2.05E-03 | 1.10E-12 |
| rs34651747 | 14 | 103255806 | T | TG | 9.51E-03 | 2.04E-03 | 2.50E-06 | 1.21E-02 | 2.08E-03 | 5.60E-09 |
| rs11374426 | 14 | 104337630 | G | GA | 2.28E-03 | 2.04E-03 | 2.70E-01 | 1.36E-02 | 2.08E-03 | 6.90E-11 |
| rs1466276 | 15 | 52025950 | C | G | 4.68E-03 | 1.95E-03 | 2.90E-02 | 1.11E-02 | 1.98E-03 | 1.90E-08 |
| rs67962220 | 15 | 74188926 | T | G | -8.14E-03 | 2.58E-03 | 1.50E-03 | -1.51E-02 | 2.63E-03 | 9.80E-09 |
| rs715724 | 15 | 80984293 | A | G | 3.43E-03 | 2.03E-03 | 5.70E-02 | 1.17E-02 | 2.06E-03 | 1.40E-08 |
| rs939624 | 15 | 99480551 | C | T | -3.03E-04 | 1.94E-03 | 9.70E-01 | -1.23E-02 | 1.98E-03 | 4.70E-10 |
| rs7200589 | 16 | 349331 | G | A | 4.56E-03 | 2.18E-03 | 3.00E-02 | 1.56E-02 | 2.22E-03 | 2.00E-12 |
| rs13329943 | 16 | 24733751 | C | T | -3.48E-03 | 2.19E-03 | 1.40E-01 | -1.28E-02 | 2.22E-03 | 7.90E-09 |
| rs3814883 | 16 | 29994922 | C | T | -6.32E-03 | 1.94E-03 | 1.90E-03 | -1.50E-02 | 1.98E-03 | 3.50E-14 |
| rs34898535 | 16 | 31025641 | C | T | 6.97E-03 | 1.99E-03 | 1.10E-03 | 1.92E-02 | 2.03E-03 | 2.80E-21 |
| rs72523664 | 16 | 69704122 | C | CTG | -9.98E-03 | 1.97E-03 | 5.60E-07 | -1.54E-02 | 2.01E-03 | 2.00E-14 |
| rs4790292 | 17 | 1824305 | C | A | 9.33E-03 | 2.69E-03 | 9.60E-04 | 1.75E-02 | 2.74E-03 | 1.80E-10 |
| rs55678940 | 17 | 21251092 | C | CTGTAAAGAAA | 7.31E-04 | 2.04E-03 | 6.60E-01 | 1.38E-02 | 2.08E-03 | 3.00E-11 |
| rs34177018 | 17 | 34912592 | CTTTTTTTTTTTT | C | -6.38E-03 | 2.12E-03 | 4.00E-03 | -1.25E-02 | 2.16E-03 | 7.00E-09 |
| rs11079849 | 17 | 47090785 | C | T | -2.10E-03 | 2.06E-03 | 3.30E-01 | 1.27E-02 | 2.10E-03 | 1.60E-09 |
| rs77706698 | 17 | 65953348 | G | A | -3.42E-03 | 2.87E-03 | 2.30E-01 | -1.72E-02 | 2.92E-03 | 4.20E-09 |
| rs2619976 | 17 | 71754545 | C | T | -3.26E-03 | 1.98E-03 | 1.40E-01 | -1.17E-02 | 2.01E-03 | 5.40E-09 |
| rs150703258 | 18 | 21079921 | C | CTGTATGTA | -8.88E-03 | 1.96E-03 | 3.20E-06 | -1.18E-02 | 1.99E-03 | 3.20E-09 |
| rs11660335 | 18 | 22154235 | T | C | 3.65E-03 | 2.47E-03 | 1.20E-01 | 1.59E-02 | 2.52E-03 | 2.50E-10 |
| rs784257 | 18 | 53397199 | T | C | 5.24E-05 | 2.49E-03 | 9.20E-01 | -1.41E-02 | 2.53E-03 | 2.90E-08 |
| rs9962947 | 18 | 72903636 | C | T | -7.12E-03 | 2.01E-03 | 5.80E-04 | -1.12E-02 | 2.05E-03 | 4.60E-08 |
| rs149080927 | 19 | 1854253 | G | GC | -8.89E-04 | 1.96E-03 | 5.80E-01 | -1.24E-02 | 1.99E-03 | 5.90E-10 |
| rs350832 | 19 | 4069426 | G | A | 3.72E-04 | 2.32E-03 | 6.20E-01 | -1.37E-02 | 2.36E-03 | 6.50E-09 |
| rs10404726 | 19 | 18834514 | C | T | 8.04E-03 | 1.94E-03 | 4.30E-05 | 1.38E-02 | 1.98E-03 | 2.50E-12 |
| rs544638445 | 19 | 19580240 | C | CAAAAAAAA | 1.54E-02 | 2.99E-03 | 1.20E-07 | 1.75E-02 | 3.05E-03 | 1.00E-08 |
| rs111640872 | 19 | 30290357 | G | C | -5.36E-03 | 2.06E-03 | 6.70E-03 | -1.26E-02 | 2.10E-03 | 1.80E-09 |
| rs429358 | 19 | 45411941 | T | C | -2.48E-03 | 2.68E-03 | 3.60E-01 | 1.75E-02 | 2.73E-03 | 1.40E-10 |
| rs1800437 | 19 | 46181392 | G | C | 1.19E-02 | 2.44E-03 | 1.60E-06 | 2.30E-02 | 2.49E-03 | 1.80E-20 |
| rs8124896 | 20 | 21385659 | T | C | -5.99E-03 | 3.21E-03 | 9.90E-02 | -2.01E-02 | 3.27E-03 | 6.90E-10 |
| rs116948922 | 20 | 25534854 | C | T | 2.83E-03 | 5.52E-03 | 7.20E-01 | 3.40E-02 | 5.62E-03 | 1.50E-09 |
| rs151157954 | 20 | 51171126 | A | ATG | 4.59E-03 | 2.65E-03 | 7.60E-02 | 1.78E-02 | 2.69E-03 | 3.50E-11 |
| rs915814 | 21 | 46493003 | G | A | 1.04E-03 | 2.27E-03 | 7.00E-01 | 1.31E-02 | 2.31E-03 | 1.50E-08 |
| rs400997 | 21 | 46564154 | T | A | -2.31E-03 | 1.96E-03 | 2.80E-01 | -1.43E-02 | 2.00E-03 | 8.00E-13 |
| rs738140 | 22 | 41884954 | A | G | 1.13E-03 | 2.09E-03 | 6.30E-01 | 1.20E-02 | 2.13E-03 | 1.80E-08 |

**Table S2G.** The genetic variants strongly associated with childhood adiposity at genome wide significance and not adulthood adiposity (exclude adult SNPs at P ≤ 0.05 with Bonferroni correction*).

| SNP | Chromosome | Base position | Effect allele | Other allele | Beta (Adult) | SE (Adult) | P (Adult) | Beta (Age 10) | SE (Age 10) | P (Age 10) |
| --- | --- | --- | --- | --- | --- | --- | --- | --- | --- | --- |
| rs4744246 | 9 | 96254464 | A | G | 9.44E-04 | 1.45E-03 | 5.10E-01 | -1.58E-02 | 1.48E-03 | 1.60E-26 |
| rs34260097 | 6 | 100727703 | T | G | -2.36E-03 | 1.64E-03 | 1.50E-01 | -1.78E-02 | 1.68E-03 | 2.50E-26 |
| rs2767486 | 1 | 65991203 | A | G | -1.36E-03 | 1.70E-03 | 4.20E-01 | -1.54E-02 | 1.74E-03 | 1.20E-18 |
| rs2594994 | 3 | 11339960 | T | A | 2.11E-03 | 1.79E-03 | 2.40E-01 | 1.54E-02 | 1.83E-03 | 3.10E-17 |
| rs117903946 | 16 | 67449639 | G | A | -9.78E-03 | 3.87E-03 | 1.10E-02 | -3.23E-02 | 3.95E-03 | 3.20E-16 |
| rs788858 | 4 | 82138300 | A | G | 5.76E-04 | 1.51E-03 | 7.00E-01 | 1.23E-02 | 1.54E-03 | 1.70E-15 |
| rs2722406 | 7 | 24306762 | C | T | -4.31E-03 | 1.52E-03 | 4.60E-03 | -1.22E-02 | 1.55E-03 | 4.40E-15 |
| rs2187642 | 12 | 11855624 | A | C | -1.58E-03 | 1.41E-03 | 2.60E-01 | -1.12E-02 | 1.44E-03 | 1.00E-14 |
| rs1342831 | 6 | 54096151 | T | C | -6.59E-03 | 2.95E-03 | 2.60E-02 | -2.25E-02 | 3.02E-03 | 9.00E-14 |
| rs117911387 | 9 | 130446836 | G | A | -3.68E-03 | 3.26E-03 | 2.60E-01 | -2.46E-02 | 3.33E-03 | 1.60E-13 |
| rs2229330 | 1 | 6649228 | T | G | -3.47E-03 | 2.64E-03 | 1.90E-01 | -1.97E-02 | 2.69E-03 | 2.30E-13 |
| rs200801362 | 6 | 31555480 | T | C | -6.08E-03 | 2.54E-03 | 1.70E-02 | -1.88E-02 | 2.60E-03 | 3.80E-13 |
| rs2275241 | 9 | 129370576 | G | A | -4.11E-03 | 1.42E-03 | 3.70E-03 | -1.04E-02 | 1.45E-03 | 7.50E-13 |
| rs7306710 | 12 | 66376091 | T | C | 7.35E-04 | 1.38E-03 | 5.90E-01 | 9.97E-03 | 1.41E-03 | 1.60E-12 |
| rs7606059 | 2 | 188152749 | T | C | -4.05E-03 | 1.46E-03 | 5.70E-03 | -1.05E-02 | 1.50E-03 | 2.10E-12 |
| rs7305424 | 12 | 118399491 | A | T | -3.88E-03 | 1.45E-03 | 7.60E-03 | -1.04E-02 | 1.48E-03 | 2.40E-12 |
| rs61937656 | 12 | 39483502 | G | A | 5.07E-03 | 1.64E-03 | 2.00E-03 | 1.17E-02 | 1.68E-03 | 2.80E-12 |
| rs3817428 | 15 | 89415247 | C | G | -3.19E-03 | 1.56E-03 | 4.10E-02 | -1.08E-02 | 1.59E-03 | 1.30E-11 |
| rs836179 | 12 | 50503082 | A | G | 2.37E-03 | 1.42E-03 | 9.50E-02 | 9.81E-03 | 1.45E-03 | 1.40E-11 |
| rs11205303 | 1 | 149906413 | T | C | 1.24E-03 | 1.39E-03 | 3.80E-01 | 9.58E-03 | 1.43E-03 | 1.80E-11 |
| rs601338 | 19 | 49206674 | G | A | 9.17E-04 | 1.37E-03 | 5.00E-01 | 9.38E-03 | 1.40E-03 | 2.10E-11 |
| rs6979832 | 7 | 127856276 | A | G | -3.30E-03 | 1.38E-03 | 1.70E-02 | -9.42E-03 | 1.41E-03 | 2.30E-11 |
| rs112898427 | 2 | 67561335 | C | T | 1.04E-03 | 1.52E-03 | 4.90E-01 | 1.03E-02 | 1.55E-03 | 2.50E-11 |
| rs7808296 | 7 | 103127620 | C | T | -2.55E-03 | 1.48E-03 | 8.50E-02 | -1.00E-02 | 1.51E-03 | 2.80E-11 |
| rs146910503 | 2 | 25446473 | G | A | 1.48E-02 | 4.91E-03 | 2.60E-03 | 3.34E-02 | 5.03E-03 | 2.90E-11 |
| rs7759938 | 6 | 105378954 | C | T | -3.05E-03 | 1.47E-03 | 3.80E-02 | -9.98E-03 | 1.50E-03 | 2.90E-11 |
| rs824207 | 15 | 24007729 | A | G | -2.21E-03 | 1.38E-03 | 1.10E-01 | -9.31E-03 | 1.40E-03 | 3.30E-11 |
| rs7162542 | 15 | 84514290 | C | G | 3.15E-03 | 1.38E-03 | 2.30E-02 | 9.31E-03 | 1.41E-03 | 4.00E-11 |
| rs2970356 | 15 | 90623540 | C | G | -3.62E-03 | 1.55E-03 | 2.00E-02 | -1.05E-02 | 1.58E-03 | 4.10E-11 |
| rs4074404 | 1 | 187683956 | T | A | -4.84E-03 | 1.88E-03 | 1.00E-02 | -1.27E-02 | 1.92E-03 | 4.30E-11 |
| rs7020564 | 9 | 109670016 | A | T | 4.97E-03 | 1.52E-03 | 1.10E-03 | 1.00E-02 | 1.55E-03 | 1.10E-10 |
| rs10095724 | 8 | 53739232 | G | A | 2.96E-03 | 1.43E-03 | 3.90E-02 | 9.37E-03 | 1.46E-03 | 1.40E-10 |
| rs8096658 | 18 | 77156537 | C | G | 4.29E-03 | 1.39E-03 | 2.00E-03 | 9.02E-03 | 1.42E-03 | 2.00E-10 |
| rs4958568 | 5 | 152016093 | G | A | 4.81E-03 | 1.53E-03 | 1.70E-03 | 9.96E-03 | 1.57E-03 | 2.10E-10 |
| rs7424771 | 2 | 161276378 | G | A | -2.31E-03 | 1.38E-03 | 9.40E-02 | 8.93E-03 | 1.41E-03 | 2.20E-10 |
| rs4572029 | 10 | 70889053 | A | G | -7.22E-04 | 1.71E-03 | 6.70E-01 | 1.11E-02 | 1.75E-03 | 2.20E-10 |
| rs77976727 | 8 | 4300554 | C | T | -7.44E-03 | 2.36E-03 | 1.60E-03 | -1.51E-02 | 2.41E-03 | 3.60E-10 |
| rs2629881 | 3 | 59778271 | C | T | -2.64E-03 | 1.65E-03 | 1.10E-01 | -1.06E-02 | 1.69E-03 | 3.80E-10 |
| rs10503246 | 8 | 4130363 | A | G | -3.08E-03 | 1.52E-03 | 4.20E-02 | -9.66E-03 | 1.55E-03 | 4.60E-10 |
| rs62621197 | 19 | 8670147 | C | T | -6.50E-03 | 3.77E-03 | 8.50E-02 | -2.35E-02 | 3.85E-03 | 9.60E-10 |
| rs212517 | 1 | 21577159 | T | A | 1.56E-03 | 1.40E-03 | 2.60E-01 | 8.75E-03 | 1.43E-03 | 9.60E-10 |
| rs62134189 | 2 | 45046339 | A | G | -1.82E-03 | 2.27E-03 | 4.20E-01 | 1.42E-02 | 2.32E-03 | 1.00E-09 |
| rs117455294 | 20 | 57427951 | C | A | 4.90E-03 | 3.11E-03 | 1.10E-01 | 1.93E-02 | 3.17E-03 | 1.10E-09 |
| rs10234366 | 7 | 46743746 | G | A | -1.75E-03 | 2.24E-03 | 4.40E-01 | -1.40E-02 | 2.29E-03 | 1.10E-09 |
| rs3936511 | 5 | 55860781 | A | G | 2.42E-04 | 1.74E-03 | 8.90E-01 | 1.08E-02 | 1.78E-03 | 1.10E-09 |
| rs75387636 | 10 | 120278394 | G | A | -7.46E-03 | 3.44E-03 | 3.00E-02 | -2.12E-02 | 3.51E-03 | 1.50E-09 |
| rs10860295 | 12 | 98542699 | T | C | -1.81E-03 | 1.38E-03 | 1.90E-01 | -8.52E-03 | 1.41E-03 | 1.60E-09 |
| rs10498713 | 6 | 22729300 | G | T | -1.36E-03 | 1.92E-03 | 4.80E-01 | -1.18E-02 | 1.97E-03 | 2.00E-09 |
| rs2999158 | 1 | 113239478 | T | C | -3.26E-03 | 1.45E-03 | 2.50E-02 | 8.87E-03 | 1.48E-03 | 2.20E-09 |
| rs7814267 | 8 | 5545084 | A | G | -5.53E-03 | 1.79E-03 | 2.00E-03 | -1.09E-02 | 1.83E-03 | 2.50E-09 |
| rs146980124 | 17 | 44627649 | A | C | 5.48E-03 | 1.69E-03 | 1.20E-03 | 1.02E-02 | 1.72E-03 | 2.90E-09 |
| rs10953577 | 7 | 108263540 | T | C | -2.48E-03 | 1.42E-03 | 8.00E-02 | -8.58E-03 | 1.45E-03 | 3.00E-09 |
| rs12748436 | 1 | 177761109 | C | G | -5.65E-03 | 2.58E-03 | 2.90E-02 | -1.56E-02 | 2.64E-03 | 3.50E-09 |
| rs4723263 | 7 | 33194826 | G | C | -2.54E-03 | 1.38E-03 | 6.60E-02 | -8.33E-03 | 1.41E-03 | 3.70E-09 |
| rs7123283 | 11 | 122809055 | C | T | 2.53E-03 | 1.38E-03 | 6.70E-02 | 8.32E-03 | 1.41E-03 | 3.70E-09 |
| rs67603370 | 17 | 7524504 | G | A | -4.59E-04 | 2.63E-03 | 8.60E-01 | -1.58E-02 | 2.68E-03 | 3.70E-09 |
| rs11891707 | 2 | 207120604 | T | C | 4.36E-04 | 2.01E-03 | 8.30E-01 | 1.21E-02 | 2.05E-03 | 3.80E-09 |
| rs9652090 | 13 | 27983367 | G | T | -3.71E-03 | 1.39E-03 | 7.40E-03 | -8.33E-03 | 1.42E-03 | 4.00E-09 |
| rs62032001 | 16 | 49065630 | A | C | -1.94E-03 | 1.73E-03 | 2.60E-01 | -1.04E-02 | 1.77E-03 | 4.20E-09 |
| rs884152 | 8 | 25770557 | G | T | -3.32E-03 | 1.43E-03 | 2.00E-02 | -8.51E-03 | 1.46E-03 | 6.00E-09 |
| rs3118252 | 9 | 25115154 | G | C | -1.47E-03 | 1.40E-03 | 2.90E-01 | -8.32E-03 | 1.43E-03 | 6.00E-09 |
| rs61936936 | 12 | 116391685 | A | T | -4.86E-03 | 2.30E-03 | 3.40E-02 | -1.36E-02 | 2.34E-03 | 6.20E-09 |
| rs3181269 | 11 | 33755956 | C | T | 3.49E-03 | 1.57E-03 | 2.60E-02 | 9.33E-03 | 1.61E-03 | 6.40E-09 |
| rs9370527 | 6 | 56245812 | G | A | -3.28E-03 | 1.60E-03 | 4.10E-02 | -9.49E-03 | 1.64E-03 | 6.40E-09 |
| rs686431 | 6 | 35974217 | C | T | -1.48E-02 | 5.09E-03 | 3.70E-03 | -3.02E-02 | 5.20E-03 | 6.50E-09 |
| rs7354849 | 1 | 232765308 | A | G | -4.26E-03 | 1.38E-03 | 2.00E-03 | -8.17E-03 | 1.41E-03 | 6.80E-09 |
| rs7672 | 16 | 68294800 | C | G | 4.69E-03 | 1.53E-03 | 2.20E-03 | 9.00E-03 | 1.56E-03 | 7.90E-09 |
| rs7503580 | 17 | 79087036 | C | T | -4.88E-03 | 1.89E-03 | 1.00E-02 | -1.11E-02 | 1.93E-03 | 8.90E-09 |
| rs115359679 | 7 | 755987 | C | A | -5.38E-03 | 2.80E-03 | 5.40E-02 | -1.64E-02 | 2.86E-03 | 9.20E-09 |
| rs7565437 | 2 | 65646966 | T | C | 2.01E-03 | 1.39E-03 | 1.50E-01 | 8.18E-03 | 1.43E-03 | 9.50E-09 |
| rs11256627 | 10 | 10535954 | G | A | -9.45E-04 | 1.51E-03 | 5.30E-01 | -8.88E-03 | 1.55E-03 | 9.50E-09 |
| rs12941038 | 17 | 66509143 | C | T | -4.75E-03 | 1.63E-03 | 3.70E-03 | -9.54E-03 | 1.67E-03 | 1.10E-08 |
| rs4545941 | 19 | 16534207 | T | C | -2.12E-03 | 1.86E-03 | 2.60E-01 | -1.09E-02 | 1.90E-03 | 1.10E-08 |
| rs4783789 | 16 | 51446707 | T | C | 1.65E-03 | 1.65E-03 | 3.20E-01 | 9.58E-03 | 1.68E-03 | 1.20E-08 |
| rs12308065 | 12 | 120624085 | A | G | -6.54E-04 | 1.42E-03 | 6.50E-01 | -8.29E-03 | 1.45E-03 | 1.20E-08 |
| rs201666051 | 9 | 20920868 | C | T | -1.49E-03 | 1.42E-03 | 2.90E-01 | -8.24E-03 | 1.45E-03 | 1.30E-08 |
| rs1476698 | 2 | 242296449 | A | G | 1.39E-03 | 1.42E-03 | 3.30E-01 | 8.22E-03 | 1.45E-03 | 1.40E-08 |
| rs2755253 | 1 | 67470843 | C | T | 1.53E-03 | 1.51E-03 | 3.10E-01 | 8.71E-03 | 1.54E-03 | 1.50E-08 |
| rs1177279 | 2 | 61295122 | A | G | 2.06E-03 | 1.53E-03 | 1.80E-01 | 8.83E-03 | 1.56E-03 | 1.60E-08 |
| rs60644673 | 7 | 100096742 | G | T | -4.06E-03 | 1.74E-03 | 2.00E-02 | 1.00E-02 | 1.78E-03 | 1.70E-08 |
| rs1696057 | 12 | 90767160 | T | C | 3.91E-03 | 1.45E-03 | 6.80E-03 | 8.31E-03 | 1.48E-03 | 1.80E-08 |
| rs6577497 | 1 | 8605667 | A | T | -2.20E-03 | 1.40E-03 | 1.20E-01 | 8.07E-03 | 1.43E-03 | 1.80E-08 |
| rs2958542 | 11 | 62181882 | C | T | 3.51E-04 | 1.43E-03 | 8.10E-01 | 8.24E-03 | 1.46E-03 | 1.80E-08 |
| rs115903965 | 3 | 66009529 | G | A | 1.86E-03 | 4.44E-03 | 6.80E-01 | -2.55E-02 | 4.54E-03 | 2.00E-08 |
| rs2939931 | 10 | 121636406 | T | C | -2.74E-03 | 1.37E-03 | 4.60E-02 | -7.86E-03 | 1.40E-03 | 2.20E-08 |
| rs8130408 | 21 | 39237138 | A | C | -4.68E-03 | 1.58E-03 | 3.10E-03 | -9.01E-03 | 1.61E-03 | 2.30E-08 |
| rs3815156 | 17 | 29685150 | A | G | -5.29E-03 | 1.81E-03 | 3.40E-03 | -1.03E-02 | 1.84E-03 | 2.30E-08 |
| rs2268762 | 3 | 38516075 | A | G | -4.39E-03 | 1.40E-03 | 1.80E-03 | -7.99E-03 | 1.44E-03 | 2.60E-08 |
| rs1402989 | 3 | 27056851 | C | T | -4.03E-03 | 1.37E-03 | 3.30E-03 | -7.79E-03 | 1.40E-03 | 2.70E-08 |
| rs7840305 | 8 | 57168101 | A | G | 3.42E-03 | 1.42E-03 | 1.60E-02 | 8.06E-03 | 1.45E-03 | 2.70E-08 |
| rs6719507 | 2 | 29733801 | G | A | 3.02E-03 | 1.38E-03 | 2.90E-02 | 7.82E-03 | 1.41E-03 | 2.90E-08 |
| rs73085586 | 20 | 22430241 | G | A | -4.65E-03 | 1.72E-03 | 6.70E-03 | -9.71E-03 | 1.75E-03 | 3.00E-08 |
| rs11040333 | 11 | 49346332 | G | A | -3.98E-03 | 1.51E-03 | 8.50E-03 | -8.55E-03 | 1.54E-03 | 3.10E-08 |
| rs3791478 | 2 | 240064139 | T | C | 4.64E-03 | 2.21E-03 | 3.60E-02 | 1.25E-02 | 2.26E-03 | 3.30E-08 |
| rs10823504 | 10 | 72034062 | G | A | 9.02E-04 | 2.81E-03 | 7.50E-01 | 1.59E-02 | 2.87E-03 | 3.40E-08 |
| rs16839832 | 1 | 196349909 | G | T | -2.65E-03 | 2.50E-03 | 2.90E-01 | -1.41E-02 | 2.55E-03 | 3.50E-08 |
| rs76187039 | 6 | 43233990 | G | T | -1.12E-03 | 2.01E-03 | 5.80E-01 | -1.13E-02 | 2.05E-03 | 3.50E-08 |
| rs2175171 | 1 | 7028842 | G | C | -1.93E-03 | 1.38E-03 | 1.60E-01 | -7.77E-03 | 1.41E-03 | 3.60E-08 |
| rs1000471 | 15 | 89986583 | C | T | 9.10E-04 | 1.69E-03 | 5.90E-01 | -9.51E-03 | 1.73E-03 | 3.60E-08 |
| rs9610387 | 22 | 36476762 | G | A | 7.89E-03 | 2.46E-03 | 1.30E-03 | 1.38E-02 | 2.51E-03 | 3.70E-08 |
| rs4677156 | 3 | 72417857 | A | T | 3.78E-03 | 1.64E-03 | 2.10E-02 | 9.25E-03 | 1.68E-03 | 3.70E-08 |
| rs3172332 | 3 | 153973408 | T | C | 2.69E-03 | 1.42E-03 | 5.80E-02 | 8.01E-03 | 1.46E-03 | 3.70E-08 |
| rs11655704 | 17 | 47448172 | T | C | 2.44E-03 | 1.47E-03 | 9.70E-02 | 8.27E-03 | 1.50E-03 | 3.80E-08 |
| rs10503555 | 8 | 15763818 | A | G | -6.46E-04 | 1.39E-03 | 6.40E-01 | 7.78E-03 | 1.41E-03 | 3.90E-08 |
| rs10111937 | 8 | 54160092 | C | T | -4.90E-03 | 1.50E-03 | 1.10E-03 | -8.37E-03 | 1.53E-03 | 4.30E-08 |
| rs67679818 | 7 | 110672704 | C | T | 4.06E-04 | 1.40E-03 | 7.70E-01 | 7.80E-03 | 1.43E-03 | 4.90E-08 |
|  |  |  |  |  |  |  |  |  |  |  |
| * Genetic variants strongly associated with adulthood adiposity at genome wide significance and not childhood adiposity (exclude child SNPs at P ≤ 0.05) were counted. We then divided 0.05 by this number to generate the Bonferroni corrected P value. | | | | | | | | | | |

**Table S2H.** The genetic variants strongly associated with childhood adiposity at genome wide significance and not adulthood adiposity (exclude adult SNPs at P ≤ 0.05 with Bonferroni correction*) in females.

| SNP | Chromosome | Base position | Effect allele | Other allele | Beta (Age 10) | SE (Age 10) | P (Age 10) | Beta (Adult) | SE (Adult) | P (Adult) |
| --- | --- | --- | --- | --- | --- | --- | --- | --- | --- | --- |
| rs212540 | 1 | 21593117 | C | T | 1.27E-02 | 2.00E-03 | 9.90E-10 | 1.56E-03 | 2.03E-03 | 4.40E-01 |
| rs2767486 | 1 | 65991203 | A | G | -2.03E-02 | 2.41E-03 | 9.90E-17 | -1.24E-03 | 2.45E-03 | 6.10E-01 |
| rs1483153 | 2 | 142358477 | C | T | -1.45E-02 | 2.34E-03 | 3.40E-10 | -5.33E-03 | 2.37E-03 | 2.50E-02 |
| rs36134621 | 2 | 161046192 | G | A | 1.11E-02 | 1.95E-03 | 3.50E-08 | -2.61E-03 | 1.98E-03 | 1.90E-01 |
| rs17464221 | 2 | 188278203 | C | T | 1.23E-02 | 2.14E-03 | 1.50E-08 | 2.65E-03 | 2.17E-03 | 2.20E-01 |
| rs115319174 | 2 | 207066474 | G | C | -4.11E-02 | 4.20E-03 | 7.60E-23 | -7.38E-03 | 4.27E-03 | 8.40E-02 |
| rs2594989 | 3 | 11316143 | C | T | 1.87E-02 | 2.53E-03 | 4.00E-14 | -4.61E-04 | 2.58E-03 | 8.60E-01 |
| rs2034963 | 3 | 48170802 | G | C | 1.34E-02 | 2.04E-03 | 5.40E-11 | 3.43E-03 | 2.08E-03 | 9.90E-02 |
| rs2629881 | 3 | 59778271 | C | T | -1.32E-02 | 2.34E-03 | 1.70E-08 | -4.55E-03 | 2.37E-03 | 5.50E-02 |
| rs818219 | 3 | 85374589 | T | C | -1.34E-02 | 1.95E-03 | 1.50E-11 | -3.91E-03 | 1.98E-03 | 4.80E-02 |
| rs1349641 | 4 | 82212652 | T | G | 1.25E-02 | 1.98E-03 | 9.70E-10 | 2.39E-03 | 2.02E-03 | 2.40E-01 |
| rs3936511 | 5 | 55860781 | A | G | 1.54E-02 | 2.46E-03 | 7.10E-11 | 8.43E-04 | 2.50E-03 | 7.40E-01 |
| rs13190020 | 5 | 65012526 | G | A | -1.15E-02 | 2.03E-03 | 1.40E-08 | -1.57E-03 | 2.07E-03 | 4.50E-01 |
| rs4235642 | 5 | 103818412 | A | G | 1.24E-02 | 2.00E-03 | 7.70E-10 | -5.90E-03 | 2.03E-03 | 3.70E-03 |
| rs767647218 | 6 | 10015295 | TTA | T | 1.37E-02 | 2.05E-03 | 2.40E-11 | 6.45E-03 | 2.09E-03 | 2.00E-03 |
| rs12110721 | 6 | 55190480 | G | A | -1.93E-02 | 2.61E-03 | 4.70E-14 | -6.96E-03 | 2.66E-03 | 8.70E-03 |
| rs34260097 | 6 | 100727703 | T | G | -2.52E-02 | 2.32E-03 | 2.40E-28 | -3.62E-03 | 2.36E-03 | 1.20E-01 |
| rs7759938 | 6 | 105378954 | C | T | -1.16E-02 | 2.07E-03 | 1.70E-08 | -1.87E-03 | 2.11E-03 | 3.80E-01 |
| rs62425122 | 6 | 166311987 | G | A | 1.24E-02 | 2.12E-03 | 4.10E-09 | 3.30E-03 | 2.15E-03 | 1.30E-01 |
| rs983949 | 7 | 24299013 | T | G | -1.30E-02 | 2.15E-03 | 4.10E-09 | -5.59E-03 | 2.18E-03 | 1.00E-02 |
| rs7808296 | 7 | 103127620 | C | T | -1.17E-02 | 2.08E-03 | 4.80E-08 | -3.17E-03 | 2.12E-03 | 1.30E-01 |
| rs6979832 | 7 | 127856276 | A | G | -1.13E-02 | 1.95E-03 | 1.60E-08 | -3.09E-03 | 1.98E-03 | 1.20E-01 |
| rs351776 | 8 | 28191306 | A | C | -1.17E-02 | 1.94E-03 | 2.00E-09 | -3.90E-03 | 1.98E-03 | 4.90E-02 |
| rs62515439 | 8 | 57165417 | C | T | 1.27E-02 | 2.01E-03 | 1.20E-10 | 3.68E-03 | 2.04E-03 | 7.20E-02 |
| rs10821163 | 9 | 96343060 | G | C | -1.84E-02 | 2.05E-03 | 3.50E-19 | 2.15E-03 | 2.09E-03 | 3.00E-01 |
| rs7084503 | 10 | 2666859 | T | C | 1.54E-02 | 1.94E-03 | 2.00E-16 | 5.66E-03 | 1.97E-03 | 4.20E-03 |
| rs79855417 | 11 | 29189203 | T | TAC | 1.90E-02 | 2.85E-03 | 7.40E-11 | 6.20E-03 | 2.90E-03 | 3.30E-02 |
| rs11215403 | 11 | 115058585 | G | A | 1.62E-02 | 2.25E-03 | 7.90E-13 | 6.71E-03 | 2.29E-03 | 3.40E-03 |
| rs2187642 | 12 | 11855624 | A | C | -1.19E-02 | 1.99E-03 | 3.80E-09 | -5.08E-04 | 2.03E-03 | 8.00E-01 |
| rs10876457 | 12 | 39453689 | G | A | 1.28E-02 | 2.32E-03 | 2.90E-08 | 6.15E-03 | 2.37E-03 | 9.30E-03 |
| rs10784514 | 12 | 66452879 | C | T | -1.16E-02 | 2.07E-03 | 4.20E-08 | -5.94E-04 | 2.11E-03 | 7.80E-01 |
| rs2364232 | 12 | 93994827 | A | C | 1.23E-02 | 2.21E-03 | 2.10E-08 | 1.02E-03 | 2.25E-03 | 6.50E-01 |
| rs7305424 | 12 | 118399491 | A | T | -1.21E-02 | 2.05E-03 | 5.30E-09 | -3.79E-03 | 2.08E-03 | 6.90E-02 |
| rs9551428 | 13 | 28618462 | C | T | 1.53E-02 | 2.00E-03 | 2.90E-14 | 6.26E-03 | 2.04E-03 | 2.10E-03 |
| rs1336486 | 13 | 40784814 | T | G | -1.47E-02 | 2.06E-03 | 1.90E-12 | -6.46E-03 | 2.10E-03 | 2.10E-03 |
| rs58681688 | 13 | 62467001 | C | G | -1.37E-02 | 2.50E-03 | 1.90E-08 | -7.24E-03 | 2.54E-03 | 4.40E-03 |
| rs4932430 | 15 | 89363866 | A | C | 1.18E-02 | 1.95E-03 | 3.70E-09 | 4.89E-03 | 1.99E-03 | 1.40E-02 |
| rs2970356 | 15 | 90623540 | C | G | -1.33E-02 | 2.19E-03 | 7.70E-10 | -5.48E-03 | 2.23E-03 | 1.40E-02 |
| rs8049326 | 16 | 3572268 | G | A | -1.22E-02 | 2.17E-03 | 9.40E-09 | -4.98E-03 | 2.20E-03 | 2.40E-02 |
| rs11863799 | 16 | 61933401 | C | T | 1.14E-02 | 2.07E-03 | 1.80E-08 | 1.35E-03 | 2.10E-03 | 5.20E-01 |
| rs34229857 | 16 | 67434917 | C | T | -4.47E-02 | 5.78E-03 | 1.70E-15 | -1.80E-02 | 5.88E-03 | 2.20E-03 |
| rs999493 | 17 | 46625519 | G | A | -1.12E-02 | 2.01E-03 | 6.30E-09 | -5.23E-03 | 2.04E-03 | 1.00E-02 |
| rs12185242 | 17 | 47407071 | A | C | -1.10E-02 | 1.94E-03 | 7.10E-09 | -2.62E-03 | 1.98E-03 | 1.90E-01 |
| rs1013737 | 18 | 937050 | G | C | -1.09E-02 | 1.94E-03 | 1.80E-08 | -5.75E-03 | 1.97E-03 | 3.60E-03 |
| rs3810304 | 19 | 30861683 | A | G | 1.43E-02 | 2.32E-03 | 1.30E-09 | 1.90E-04 | 2.36E-03 | 9.40E-01 |
| rs4805881 | 19 | 33896432 | A | C | -1.17E-02 | 2.05E-03 | 4.50E-08 | -5.86E-03 | 2.09E-03 | 5.00E-03 |
| rs633372 | 19 | 49209226 | G | A | 1.11E-02 | 1.94E-03 | 5.70E-09 | 1.84E-03 | 1.97E-03 | 3.50E-01 |
| rs7268466 | 20 | 15810676 | C | T | -2.01E-02 | 2.78E-03 | 7.40E-13 | -8.36E-03 | 2.83E-03 | 3.10E-03 |
| rs763842194 | 20 | 54377089 | AG | A | 1.50E-02 | 2.20E-03 | 1.30E-11 | 2.91E-03 | 2.23E-03 | 1.90E-01 |
| rs374873051 | 22 | 22266944 | CTTTTTTTTTTTTT | C | -1.13E-02 | 2.02E-03 | 1.70E-09 | -2.21E-03 | 2.06E-03 | 2.80E-01 |
|  |  |  |  |  |  |  |  |  |  |  |
| * Genetic variants strongly associated with adulthood adiposity at genome wide significance and not childhood adiposity (exclude child SNPs at P ≤ 0.05) were counted. We then divided 0.05 by this number to generate the Bonferroni corrected P value. | | | | | | | | | | |

**Table S2I.** The genetic variants strongly associated with adulthood adiposity at genome wide significance and not childhood adiposity (exclude child SNPs at P ≤ 0.05 with Bonferroni correction**).

| SNP | Chromosome | Base position | Effect allele | Other allele | Beta (Adult) | SE (Adult) | P (Adult) | Beta (Age 10) | SE (Age 10) | P (Age 10) |
| --- | --- | --- | --- | --- | --- | --- | --- | --- | --- | --- |
| rs3931548 | 9 | 103113652 | C | A | -1.05E-02 | 1.44E-03 | 2.80E-13 | -5.41E-03 | 1.47E-03 | 2.30E-04 |
| rs1286138 | 14 | 91485445 | T | G | -9.00E-03 | 1.47E-03 | 8.40E-10 | -5.44E-03 | 1.50E-03 | 2.80E-04 |
| rs4482463 | 2 | 205375909 | C | A | 1.89E-02 | 2.57E-03 | 2.30E-13 | 9.47E-03 | 2.63E-03 | 3.20E-04 |
| rs1805123 | 7 | 150645534 | T | G | 1.10E-02 | 1.59E-03 | 4.10E-12 | 5.84E-03 | 1.63E-03 | 3.30E-04 |
| rs12788343 | 11 | 131452912 | T | C | -9.65E-03 | 1.39E-03 | 4.30E-12 | -5.10E-03 | 1.42E-03 | 3.40E-04 |
| rs142315514 | 1 | 147050816 | C | A | -2.14E-02 | 3.79E-03 | 1.70E-08 | -1.39E-02 | 3.87E-03 | 3.50E-04 |
| rs2450444 | 10 | 93010383 | G | A | 8.07E-03 | 1.44E-03 | 2.00E-08 | 5.24E-03 | 1.47E-03 | 3.60E-04 |
| rs2253310 | 6 | 108888593 | C | G | -1.06E-02 | 1.42E-03 | 9.00E-14 | -5.14E-03 | 1.45E-03 | 3.90E-04 |
| rs56803094 | 15 | 99222509 | A | G | 9.58E-03 | 1.65E-03 | 5.80E-09 | 5.94E-03 | 1.68E-03 | 4.00E-04 |
| rs6963840 | 7 | 78144371 | C | T | -1.29E-02 | 1.89E-03 | 8.50E-12 | -6.84E-03 | 1.93E-03 | 4.10E-04 |
| rs12147845 | 14 | 101144596 | C | T | -1.36E-02 | 2.15E-03 | 2.60E-10 | -7.75E-03 | 2.19E-03 | 4.10E-04 |
| rs1296685 | 22 | 18230964 | A | G | -9.49E-03 | 1.70E-03 | 2.20E-08 | -6.09E-03 | 1.73E-03 | 4.40E-04 |
| rs115866895 | 1 | 1592638 | A | G | 1.20E-02 | 1.57E-03 | 1.70E-14 | 5.60E-03 | 1.60E-03 | 4.70E-04 |
| rs4677813 | 3 | 194863860 | T | C | 8.93E-03 | 1.58E-03 | 1.60E-08 | 5.60E-03 | 1.62E-03 | 5.30E-04 |
| rs72618637 | 2 | 48953979 | T | A | 9.99E-03 | 1.77E-03 | 1.80E-08 | 6.29E-03 | 1.81E-03 | 5.30E-04 |
| rs6548220 | 2 | 225951 | A | G | 9.36E-03 | 1.48E-03 | 2.80E-10 | 5.24E-03 | 1.52E-03 | 5.50E-04 |
| rs13329943 | 16 | 24733751 | C | T | -1.13E-02 | 1.55E-03 | 3.20E-13 | -5.46E-03 | 1.59E-03 | 5.70E-04 |
| rs12634936 | 3 | 147716498 | T | C | -1.81E-02 | 3.15E-03 | 8.80E-09 | -1.11E-02 | 3.22E-03 | 5.80E-04 |
| rs12462975 | 19 | 30272202 | G | A | -1.15E-02 | 1.47E-03 | 5.90E-15 | -5.15E-03 | 1.50E-03 | 6.00E-04 |
| rs11633022 | 15 | 73074890 | C | A | -1.09E-02 | 1.44E-03 | 4.30E-14 | -5.04E-03 | 1.47E-03 | 6.30E-04 |
| rs3823674 | 7 | 50571996 | C | T | 7.66E-03 | 1.39E-03 | 3.50E-08 | 4.84E-03 | 1.42E-03 | 6.40E-04 |
| rs6843852 | 4 | 162132758 | C | T | -8.72E-03 | 1.37E-03 | 2.00E-10 | -4.77E-03 | 1.40E-03 | 6.70E-04 |
| rs1633418 | 22 | 20091756 | T | C | 7.92E-03 | 1.41E-03 | 2.00E-08 | 4.89E-03 | 1.44E-03 | 6.90E-04 |
| rs6798941 | 3 | 52893465 | C | T | -1.10E-02 | 1.51E-03 | 2.70E-13 | -5.21E-03 | 1.54E-03 | 7.20E-04 |
| rs9814758 | 3 | 123062657 | T | G | 8.39E-03 | 1.44E-03 | 5.40E-09 | 4.89E-03 | 1.47E-03 | 8.80E-04 |
| rs7102934 | 11 | 84648068 | T | C | -9.08E-03 | 1.49E-03 | 1.20E-09 | -5.07E-03 | 1.53E-03 | 8.90E-04 |
| rs113962925 | 17 | 46044446 | C | T | -1.71E-02 | 2.64E-03 | 8.60E-11 | -8.95E-03 | 2.69E-03 | 8.90E-04 |
| rs78565420 | 8 | 85703065 | C | T | -1.88E-02 | 3.15E-03 | 2.70E-09 | -1.07E-02 | 3.22E-03 | 9.20E-04 |
| rs112875651 | 8 | 126506694 | G | A | -8.35E-03 | 1.42E-03 | 4.30E-09 | -4.79E-03 | 1.45E-03 | 9.70E-04 |
| rs811054 | 16 | 72251132 | C | T | -8.64E-03 | 1.38E-03 | 4.40E-10 | -4.66E-03 | 1.41E-03 | 9.80E-04 |
| rs80082536 | 3 | 35195311 | A | G | -1.29E-02 | 2.12E-03 | 1.30E-09 | -7.15E-03 | 2.17E-03 | 9.90E-04 |
| rs8038574 | 15 | 95275890 | T | C | 9.07E-03 | 1.45E-03 | 4.00E-10 | 4.85E-03 | 1.48E-03 | 1.10E-03 |
| rs13061117 | 3 | 181186466 | T | C | -1.47E-02 | 2.42E-03 | 1.30E-09 | -7.99E-03 | 2.48E-03 | 1.20E-03 |
| rs273505 | 19 | 18217147 | T | C | -1.01E-02 | 1.39E-03 | 3.10E-13 | -4.52E-03 | 1.42E-03 | 1.40E-03 |
| rs12517187 | 5 | 112444682 | C | T | -8.25E-03 | 1.39E-03 | 2.80E-09 | -4.51E-03 | 1.42E-03 | 1.50E-03 |
| rs2289379 | 7 | 44804225 | C | T | 9.76E-03 | 1.41E-03 | 4.10E-12 | 4.57E-03 | 1.44E-03 | 1.50E-03 |
| rs2186118 | 1 | 66456465 | C | A | 9.86E-03 | 1.51E-03 | 6.90E-11 | 4.90E-03 | 1.55E-03 | 1.50E-03 |
| rs62171698 | 2 | 143959096 | C | A | -1.21E-02 | 1.97E-03 | 8.80E-10 | -6.39E-03 | 2.01E-03 | 1.50E-03 |
| rs1167311 | 1 | 49996959 | G | A | 1.23E-02 | 1.48E-03 | 1.20E-16 | 4.77E-03 | 1.51E-03 | 1.60E-03 |
| rs2875762 | 6 | 124925032 | G | C | -1.10E-02 | 1.60E-03 | 6.00E-12 | -5.17E-03 | 1.64E-03 | 1.60E-03 |
| rs1436348 | 3 | 104612668 | A | G | -9.66E-03 | 1.39E-03 | 3.50E-12 | -4.46E-03 | 1.42E-03 | 1.70E-03 |
| rs845084 | 10 | 125220036 | G | A | -9.85E-03 | 1.57E-03 | 3.70E-10 | -5.04E-03 | 1.61E-03 | 1.70E-03 |
| rs724623 | 14 | 47303577 | A | C | 1.02E-02 | 1.37E-03 | 9.00E-14 | 4.37E-03 | 1.40E-03 | 1.80E-03 |
| rs7103389 | 11 | 881639 | T | C | -9.46E-03 | 1.42E-03 | 2.90E-11 | -4.53E-03 | 1.45E-03 | 1.80E-03 |
| rs34994596 | 15 | 80991447 | T | C | 1.12E-02 | 1.50E-03 | 1.10E-13 | 4.77E-03 | 1.53E-03 | 1.80E-03 |
| rs544200874 | 4 | 20124826 | C | T | -1.77E-02 | 2.71E-03 | 7.20E-11 | -8.65E-03 | 2.77E-03 | 1.80E-03 |
| rs4672338 | 2 | 60217457 | C | T | -8.35E-03 | 1.45E-03 | 8.00E-09 | -4.60E-03 | 1.48E-03 | 1.90E-03 |
| rs4648450 | 1 | 2723214 | C | A | 9.80E-03 | 1.38E-03 | 1.20E-12 | 4.35E-03 | 1.41E-03 | 2.00E-03 |
| rs3125326 | 10 | 63053788 | A | C | -7.89E-03 | 1.42E-03 | 2.70E-08 | -4.48E-03 | 1.45E-03 | 2.00E-03 |
| rs11856579 | 15 | 78012688 | G | A | 9.18E-03 | 1.55E-03 | 3.40E-09 | 4.88E-03 | 1.59E-03 | 2.10E-03 |
| rs114263339 | 5 | 50932343 | C | T | -2.53E-02 | 4.33E-03 | 5.20E-09 | -1.36E-02 | 4.42E-03 | 2.10E-03 |
| rs1477890 | 4 | 18511738 | A | G | -9.78E-03 | 1.37E-03 | 1.10E-12 | -4.27E-03 | 1.40E-03 | 2.40E-03 |
| rs6029180 | 20 | 39178923 | A | G | -8.19E-03 | 1.48E-03 | 3.10E-08 | -4.57E-03 | 1.51E-03 | 2.40E-03 |
| rs7749708 | 6 | 153375907 | C | T | -9.98E-03 | 1.51E-03 | 3.70E-11 | -4.69E-03 | 1.54E-03 | 2.40E-03 |
| rs1222216 | 11 | 30346052 | C | T | 1.22E-02 | 1.64E-03 | 1.10E-13 | 5.09E-03 | 1.67E-03 | 2.40E-03 |
| rs35894137 | 8 | 43071838 | C | T | 1.42E-02 | 2.52E-03 | 1.60E-08 | 7.82E-03 | 2.57E-03 | 2.40E-03 |
| rs72652703 | 8 | 67209548 | T | C | 9.92E-03 | 1.58E-03 | 3.20E-10 | 4.87E-03 | 1.61E-03 | 2.50E-03 |
| rs12363672 | 11 | 55684028 | A | C | -2.38E-02 | 4.29E-03 | 2.80E-08 | -1.32E-02 | 4.38E-03 | 2.50E-03 |
| rs74929176 | 17 | 54905494 | C | T | 9.05E-03 | 1.65E-03 | 4.40E-08 | 5.04E-03 | 1.69E-03 | 2.80E-03 |
| rs17619860 | 8 | 87779603 | T | C | -1.04E-02 | 1.86E-03 | 2.20E-08 | -5.67E-03 | 1.90E-03 | 2.80E-03 |
| rs35221880 | 12 | 133301500 | T | C | 8.57E-03 | 1.55E-03 | 3.50E-08 | 4.72E-03 | 1.59E-03 | 2.90E-03 |
| rs11000993 | 10 | 76084111 | T | C | -1.38E-02 | 2.08E-03 | 3.10E-11 | -6.34E-03 | 2.12E-03 | 2.90E-03 |
| rs35809007 | 2 | 47019521 | G | A | 1.06E-02 | 1.43E-03 | 1.20E-13 | 4.34E-03 | 1.46E-03 | 3.00E-03 |
| rs9480184 | 6 | 155987788 | C | T | -9.86E-03 | 1.68E-03 | 4.90E-09 | -5.11E-03 | 1.72E-03 | 3.00E-03 |
| rs66460909 | 20 | 51195387 | G | A | 1.57E-02 | 1.75E-03 | 2.90E-19 | 5.27E-03 | 1.79E-03 | 3.10E-03 |
| rs7924036 | 10 | 65191645 | G | T | 9.63E-03 | 1.37E-03 | 2.20E-12 | 4.10E-03 | 1.40E-03 | 3.40E-03 |
| rs4575195 | 10 | 114765747 | C | A | 9.59E-03 | 1.48E-03 | 9.00E-11 | 4.43E-03 | 1.51E-03 | 3.40E-03 |
| rs4148155 | 4 | 89054667 | A | G | 1.43E-02 | 2.16E-03 | 2.90E-11 | 6.42E-03 | 2.21E-03 | 3.60E-03 |
| rs9843653 | 3 | 49920571 | T | C | -1.76E-02 | 1.37E-03 | 1.50E-37 | -4.07E-03 | 1.40E-03 | 3.70E-03 |
| rs7141912 | 14 | 35649431 | A | T | 1.18E-02 | 2.07E-03 | 1.10E-08 | 6.14E-03 | 2.11E-03 | 3.70E-03 |
| rs148137538 | 1 | 173399677 | A | G | 2.50E-02 | 4.58E-03 | 4.40E-08 | 1.35E-02 | 4.68E-03 | 3.80E-03 |
| rs45521740 | 19 | 2245622 | G | A | -1.80E-02 | 2.95E-03 | 1.00E-09 | -8.69E-03 | 3.01E-03 | 3.90E-03 |
| rs13218383 | 6 | 120173501 | C | G | 9.32E-03 | 1.45E-03 | 1.40E-10 | 4.22E-03 | 1.48E-03 | 4.40E-03 |
| rs3902951 | 14 | 69789755 | T | G | -1.05E-02 | 1.63E-03 | 1.10E-10 | -4.73E-03 | 1.67E-03 | 4.50E-03 |
| rs680071 | 11 | 103088414 | T | C | -1.17E-02 | 2.11E-03 | 2.80E-08 | -6.14E-03 | 2.16E-03 | 4.50E-03 |
| rs412243 | 16 | 339672 | T | C | 1.05E-02 | 1.42E-03 | 1.40E-13 | 4.08E-03 | 1.45E-03 | 4.70E-03 |
| rs10791113 | 11 | 130873165 | A | G | -8.57E-03 | 1.38E-03 | 4.60E-10 | -3.96E-03 | 1.40E-03 | 4.80E-03 |
| rs113132247 | 9 | 131026108 | G | A | -1.23E-02 | 1.91E-03 | 1.10E-10 | -5.50E-03 | 1.95E-03 | 4.80E-03 |
| rs651533 | 1 | 82375561 | T | A | 9.30E-03 | 1.59E-03 | 5.60E-09 | 4.56E-03 | 1.63E-03 | 5.20E-03 |
| rs34292685 | 11 | 64049021 | C | T | 1.27E-02 | 1.86E-03 | 7.90E-12 | 5.31E-03 | 1.90E-03 | 5.20E-03 |
| rs17029006 | 3 | 12329452 | C | T | 1.04E-02 | 1.55E-03 | 2.20E-11 | 4.42E-03 | 1.59E-03 | 5.40E-03 |
| rs7424120 | 2 | 59313974 | C | T | 1.42E-02 | 1.40E-03 | 4.00E-24 | 3.98E-03 | 1.43E-03 | 5.50E-03 |
| rs544957562 | 9 | 33978015 | A | T | 1.24E-02 | 2.02E-03 | 9.00E-10 | 5.74E-03 | 2.07E-03 | 5.50E-03 |
| rs1250597 | 10 | 81010250 | A | G | -8.54E-03 | 1.40E-03 | 1.00E-09 | 3.94E-03 | 1.43E-03 | 5.80E-03 |
| rs2083323 | 18 | 1856272 | G | A | -1.04E-02 | 1.80E-03 | 7.30E-09 | -5.03E-03 | 1.84E-03 | 6.20E-03 |
| rs719802 | 11 | 113234679 | T | C | 8.20E-03 | 1.41E-03 | 5.70E-09 | 3.91E-03 | 1.44E-03 | 6.50E-03 |
| rs1568488 | 3 | 153657951 | G | C | -1.10E-02 | 1.41E-03 | 4.40E-15 | -3.86E-03 | 1.44E-03 | 7.20E-03 |
| rs17716502 | 8 | 116659731 | C | T | 1.62E-02 | 1.71E-03 | 2.70E-21 | 4.71E-03 | 1.75E-03 | 7.20E-03 |
| rs57488047 | 15 | 79403002 | T | C | 9.86E-03 | 1.39E-03 | 1.10E-12 | 3.77E-03 | 1.41E-03 | 7.60E-03 |
| rs28839214 | 4 | 145313641 | G | T | -8.92E-03 | 1.41E-03 | 2.20E-10 | -3.83E-03 | 1.44E-03 | 7.60E-03 |
| rs2583410 | 4 | 102182199 | A | C | -1.34E-02 | 1.94E-03 | 5.30E-12 | -5.22E-03 | 1.98E-03 | 8.30E-03 |
| rs2343681 | 3 | 136535024 | G | A | -1.24E-02 | 1.68E-03 | 1.80E-13 | -4.51E-03 | 1.72E-03 | 8.60E-03 |
| rs12821683 | 12 | 58588964 | G | C | -1.12E-02 | 2.01E-03 | 2.80E-08 | -5.32E-03 | 2.06E-03 | 9.60E-03 |
| rs72917533 | 2 | 175238924 | T | C | 1.15E-02 | 1.76E-03 | 8.20E-11 | 4.66E-03 | 1.80E-03 | 9.70E-03 |
| rs76824303 | 3 | 62459819 | A | C | 1.52E-02 | 2.34E-03 | 9.20E-11 | 6.18E-03 | 2.40E-03 | 9.80E-03 |
| rs143662847 | 8 | 48804722 | C | T | 1.93E-02 | 3.53E-03 | 4.60E-08 | 9.32E-03 | 3.61E-03 | 9.90E-03 |
| rs1778830 | 1 | 156489974 | G | A | -9.91E-03 | 1.43E-03 | 3.80E-12 | -3.74E-03 | 1.46E-03 | 1.00E-02 |
| rs17770336 | 9 | 28414625 | C | T | -1.55E-02 | 1.46E-03 | 3.10E-26 | -3.84E-03 | 1.50E-03 | 1.00E-02 |
| rs61813324 | 1 | 156049877 | C | T | -1.83E-02 | 2.03E-03 | 1.80E-19 | -5.34E-03 | 2.07E-03 | 1.00E-02 |
| rs80082351 | 3 | 114415926 | A | G | 1.85E-02 | 2.85E-03 | 8.10E-11 | 7.49E-03 | 2.91E-03 | 1.00E-02 |
| rs7038966 | 9 | 73777777 | C | T | -9.48E-03 | 1.40E-03 | 1.40E-11 | -3.65E-03 | 1.43E-03 | 1.10E-02 |
| rs2247401 | 15 | 53156672 | G | A | -8.52E-03 | 1.56E-03 | 4.70E-08 | -4.08E-03 | 1.59E-03 | 1.10E-02 |
| rs329118 | 5 | 133861663 | C | T | 9.49E-03 | 1.39E-03 | 8.80E-12 | 3.53E-03 | 1.42E-03 | 1.30E-02 |
| rs7549358 | 1 | 115252609 | G | C | 8.83E-03 | 1.43E-03 | 6.50E-10 | 3.62E-03 | 1.46E-03 | 1.30E-02 |
| rs8124896 | 20 | 21385659 | T | C | -1.32E-02 | 2.28E-03 | 8.10E-09 | -5.77E-03 | 2.33E-03 | 1.30E-02 |
| rs2425856 | 20 | 44911954 | A | G | 8.42E-03 | 1.38E-03 | 1.10E-09 | 3.48E-03 | 1.41E-03 | 1.40E-02 |
| rs4792716 | 17 | 15943144 | A | G | -8.11E-03 | 1.38E-03 | 4.80E-09 | -3.47E-03 | 1.41E-03 | 1.40E-02 |
| rs9834519 | 3 | 156379637 | C | T | 1.56E-02 | 2.54E-03 | 8.40E-10 | 6.36E-03 | 2.60E-03 | 1.40E-02 |
| rs6752979 | 2 | 81741750 | G | A | -8.96E-03 | 1.47E-03 | 1.10E-09 | -3.67E-03 | 1.50E-03 | 1.50E-02 |
| rs1451533 | 2 | 105466005 | G | A | -1.10E-02 | 1.55E-03 | 1.30E-12 | -3.84E-03 | 1.58E-03 | 1.50E-02 |
| rs79686965 | 11 | 46020909 | A | G | -3.10E-02 | 5.56E-03 | 2.30E-08 | -1.38E-02 | 5.68E-03 | 1.50E-02 |
| rs61746970 | 19 | 51132746 | G | A | -2.13E-02 | 3.62E-03 | 3.80E-09 | -8.88E-03 | 3.69E-03 | 1.60E-02 |
| rs215634 | 7 | 32369148 | A | G | 1.04E-02 | 1.41E-03 | 1.40E-13 | 3.44E-03 | 1.44E-03 | 1.70E-02 |
| rs12031634 | 1 | 34584393 | G | A | 8.81E-03 | 1.50E-03 | 4.80E-09 | 3.60E-03 | 1.54E-03 | 1.90E-02 |
| rs2658797 | 11 | 93212254 | C | T | 7.79E-03 | 1.37E-03 | 1.30E-08 | 3.25E-03 | 1.40E-03 | 2.00E-02 |
| rs529200 | 3 | 173114305 | A | G | -1.03E-02 | 1.37E-03 | 5.80E-14 | -3.24E-03 | 1.40E-03 | 2.10E-02 |
| rs6583310 | 3 | 196170985 | G | C | -8.78E-03 | 1.38E-03 | 2.20E-10 | -3.26E-03 | 1.42E-03 | 2.10E-02 |
| rs61986330 | 14 | 73314450 | C | A | 8.73E-03 | 1.54E-03 | 1.40E-08 | 3.63E-03 | 1.57E-03 | 2.10E-02 |
| rs537508 | 4 | 171042158 | G | C | -7.77E-03 | 1.40E-03 | 2.60E-08 | -3.26E-03 | 1.43E-03 | 2.20E-02 |
| rs556992087 | 12 | 124500725 | T | C | -1.06E-02 | 1.67E-03 | 2.00E-10 | -3.91E-03 | 1.70E-03 | 2.20E-02 |
| rs73078357 | 3 | 48695834 | T | C | 1.19E-02 | 2.09E-03 | 1.30E-08 | 4.89E-03 | 2.14E-03 | 2.20E-02 |
| rs9673839 | 16 | 76895693 | A | G | -8.30E-03 | 1.38E-03 | 1.70E-09 | -3.20E-03 | 1.41E-03 | 2.30E-02 |
| rs788163 | 2 | 172931559 | A | C | -1.01E-02 | 1.54E-03 | 5.90E-11 | -3.57E-03 | 1.57E-03 | 2.30E-02 |
| rs62379271 | 5 | 105870033 | T | G | -7.81E-03 | 1.39E-03 | 2.00E-08 | -3.22E-03 | 1.42E-03 | 2.40E-02 |
| rs2725371 | 8 | 30854033 | A | G | 1.00E-02 | 1.50E-03 | 2.00E-11 | -3.46E-03 | 1.53E-03 | 2.40E-02 |
| rs6069625 | 20 | 54747469 | A | G | 8.73E-03 | 1.56E-03 | 2.00E-08 | 3.58E-03 | 1.59E-03 | 2.40E-02 |
| rs12213441 | 6 | 143208838 | C | T | -1.05E-02 | 1.67E-03 | 3.30E-10 | -3.85E-03 | 1.71E-03 | 2.40E-02 |
| rs76702514 | 1 | 195148296 | C | G | 1.01E-02 | 1.69E-03 | 2.00E-09 | 3.91E-03 | 1.73E-03 | 2.40E-02 |
| rs11218510 | 11 | 121922587 | G | A | 8.10E-03 | 1.40E-03 | 7.80E-09 | 3.22E-03 | 1.43E-03 | 2.50E-02 |
| rs1598121 | 3 | 82694710 | A | G | -8.96E-03 | 1.42E-03 | 3.00E-10 | -3.23E-03 | 1.46E-03 | 2.70E-02 |
| rs10457469 | 6 | 126083658 | G | A | -7.63E-03 | 1.37E-03 | 2.60E-08 | -3.08E-03 | 1.40E-03 | 2.80E-02 |
| rs9579775 | 13 | 20616557 | A | C | -1.46E-02 | 2.08E-03 | 2.10E-12 | -4.68E-03 | 2.13E-03 | 2.80E-02 |
| rs6511826 | 19 | 12706991 | G | A | 1.40E-02 | 2.46E-03 | 1.30E-08 | 5.51E-03 | 2.51E-03 | 2.80E-02 |
| rs7925100 | 11 | 118941596 | G | A | -9.17E-03 | 1.40E-03 | 6.30E-11 | -3.13E-03 | 1.43E-03 | 2.90E-02 |
| rs347551 | 5 | 119389031 | C | G | -8.89E-03 | 1.39E-03 | 1.80E-10 | -3.09E-03 | 1.43E-03 | 3.00E-02 |
| rs1899689 | 7 | 121964349 | C | T | -7.74E-03 | 1.41E-03 | 3.70E-08 | -3.10E-03 | 1.44E-03 | 3.10E-02 |
| rs11150462 | 16 | 82451679 | T | A | 8.44E-03 | 1.42E-03 | 3.10E-09 | 3.10E-03 | 1.46E-03 | 3.30E-02 |
| rs6597653 | 9 | 133788465 | G | C | -8.53E-03 | 1.41E-03 | 1.30E-09 | -3.05E-03 | 1.44E-03 | 3.40E-02 |
| rs115778101 | 1 | 78198554 | T | C | 1.78E-02 | 3.22E-03 | 3.40E-08 | 6.92E-03 | 3.29E-03 | 3.50E-02 |
| rs67913249 | 5 | 43204126 | C | G | 9.35E-03 | 1.45E-03 | 1.20E-10 | 3.07E-03 | 1.48E-03 | 3.80E-02 |
| rs7511698 | 1 | 25015638 | C | T | 8.21E-03 | 1.49E-03 | 3.20E-08 | 3.14E-03 | 1.52E-03 | 3.80E-02 |
| rs9852062 | 3 | 45373442 | T | A | 8.17E-03 | 1.38E-03 | 3.50E-09 | 2.89E-03 | 1.41E-03 | 4.10E-02 |
| rs2114210 | 8 | 95595162 | G | A | -9.86E-03 | 1.45E-03 | 1.20E-11 | -3.02E-03 | 1.49E-03 | 4.20E-02 |
| rs6030803 | 20 | 41986507 | T | C | 1.29E-02 | 2.07E-03 | 4.50E-10 | 4.30E-03 | 2.12E-03 | 4.20E-02 |
| rs1409158 | 1 | 119538890 | C | T | 9.23E-03 | 1.61E-03 | 1.00E-08 | 3.33E-03 | 1.65E-03 | 4.30E-02 |
| rs13174863 | 5 | 139080745 | A | G | -1.36E-02 | 1.94E-03 | 2.40E-12 | -4.03E-03 | 1.99E-03 | 4.30E-02 |
| rs10174253 | 2 | 181323160 | A | C | -1.12E-02 | 1.53E-03 | 2.50E-13 | -3.08E-03 | 1.57E-03 | 4.90E-02 |
| rs61754230 | 12 | 72179446 | C | T | -2.74E-02 | 4.93E-03 | 2.80E-08 | -9.81E-03 | 5.03E-03 | 5.10E-02 |
| rs7086898 | 10 | 104386152 | A | G | -1.39E-02 | 2.53E-03 | 4.30E-08 | -4.98E-03 | 2.59E-03 | 5.40E-02 |
| rs1631026 | 2 | 26953850 | C | T | -1.07E-02 | 1.37E-03 | 5.90E-15 | -2.69E-03 | 1.40E-03 | 5.50E-02 |
| rs1964926 | 21 | 42653121 | A | G | -8.23E-03 | 1.44E-03 | 1.10E-08 | -2.82E-03 | 1.47E-03 | 5.50E-02 |
| rs112852122 | 20 | 47498117 | G | A | 1.34E-02 | 1.91E-03 | 2.70E-12 | 3.75E-03 | 1.95E-03 | 5.50E-02 |
| rs6761463 | 2 | 50201547 | G | C | 1.26E-02 | 1.86E-03 | 1.30E-11 | 3.61E-03 | 1.90E-03 | 5.70E-02 |
| rs11047138 | 12 | 24019853 | C | G | -1.44E-02 | 2.63E-03 | 4.60E-08 | -5.08E-03 | 2.68E-03 | 5.80E-02 |
| rs16940823 | 18 | 22137319 | C | A | 1.08E-02 | 1.79E-03 | 1.60E-09 | 3.42E-03 | 1.82E-03 | 6.10E-02 |
| rs66674732 | 13 | 62721160 | G | A | -8.16E-03 | 1.41E-03 | 7.20E-09 | -2.68E-03 | 1.44E-03 | 6.30E-02 |
| rs1381010 | 4 | 112677085 | G | A | 8.24E-03 | 1.49E-03 | 3.00E-08 | 2.81E-03 | 1.52E-03 | 6.40E-02 |
| rs369461388 | 14 | 40104718 | G | C | -1.26E-02 | 2.01E-03 | 3.60E-10 | -3.80E-03 | 2.05E-03 | 6.40E-02 |
| rs1369159 | 15 | 66360842 | C | T | 7.88E-03 | 1.40E-03 | 1.70E-08 | 2.63E-03 | 1.43E-03 | 6.50E-02 |
| rs4989244 | 9 | 102100348 | G | A | 7.63E-03 | 1.39E-03 | 3.60E-08 | 2.61E-03 | 1.42E-03 | 6.60E-02 |
| rs12149660 | 16 | 70309237 | G | A | 1.62E-02 | 2.16E-03 | 6.20E-14 | 4.01E-03 | 2.21E-03 | 6.90E-02 |
| rs2035806 | 10 | 133984916 | G | A | 9.55E-03 | 1.39E-03 | 5.70E-12 | 2.56E-03 | 1.42E-03 | 7.00E-02 |
| rs429358 | 19 | 45411941 | T | C | 1.61E-02 | 1.90E-03 | 2.90E-17 | -3.45E-03 | 1.94E-03 | 7.50E-02 |
| rs7230240 | 18 | 42597978 | C | T | 9.40E-03 | 1.50E-03 | 4.20E-10 | 2.73E-03 | 1.54E-03 | 7.60E-02 |
| rs34542489 | 17 | 51917844 | A | C | 7.78E-03 | 1.40E-03 | 2.80E-08 | 2.52E-03 | 1.43E-03 | 7.80E-02 |
| rs201475383 | 20 | 26273991 | G | A | 2.40E-02 | 3.92E-03 | 8.80E-10 | 6.98E-03 | 4.00E-03 | 8.10E-02 |
| rs2056477 | 7 | 2079744 | G | C | 1.16E-02 | 1.64E-03 | 1.50E-12 | 2.90E-03 | 1.67E-03 | 8.30E-02 |
| rs6075658 | 20 | 2094078 | T | C | 8.49E-03 | 1.38E-03 | 6.80E-10 | 2.43E-03 | 1.40E-03 | 8.40E-02 |
| rs368540015 | 7 | 74292165 | A | G | -1.82E-02 | 3.25E-03 | 2.20E-08 | -5.73E-03 | 3.32E-03 | 8.40E-02 |
| rs142503704 | 5 | 92622421 | G | A | -2.63E-02 | 4.68E-03 | 1.90E-08 | -8.13E-03 | 4.78E-03 | 8.90E-02 |
| rs6050446 | 20 | 25195509 | A | G | -2.64E-02 | 3.89E-03 | 1.20E-11 | -6.70E-03 | 3.98E-03 | 9.20E-02 |
| rs1987960 | 20 | 30649834 | T | C | -1.89E-02 | 3.31E-03 | 1.10E-08 | -5.63E-03 | 3.38E-03 | 9.60E-02 |
| rs2660241 | 16 | 4940023 | T | C | -8.13E-03 | 1.43E-03 | 1.20E-08 | -2.41E-03 | 1.46E-03 | 9.90E-02 |
| rs3737992 | 1 | 33234128 | G | A | 1.37E-02 | 1.83E-03 | 5.30E-14 | 3.07E-03 | 1.87E-03 | 9.90E-02 |
| rs6823268 | 4 | 145982563 | A | G | -8.18E-03 | 1.42E-03 | 8.20E-09 | -2.36E-03 | 1.45E-03 | 1.00E-01 |
| rs75957461 | 19 | 11166163 | C | T | -1.77E-02 | 3.05E-03 | 6.60E-09 | -5.11E-03 | 3.11E-03 | 1.00E-01 |
| rs7182917 | 15 | 52080803 | T | C | 8.74E-03 | 1.39E-03 | 2.80E-10 | 2.25E-03 | 1.41E-03 | 1.10E-01 |
| rs9615723 | 22 | 48386670 | C | T | 7.71E-03 | 1.40E-03 | 3.80E-08 | 2.26E-03 | 1.43E-03 | 1.10E-01 |
| rs10960276 | 9 | 11819686 | C | A | 8.09E-03 | 1.43E-03 | 1.60E-08 | -2.34E-03 | 1.46E-03 | 1.10E-01 |
| rs4911382 | 20 | 32553095 | C | T | -8.32E-03 | 1.40E-03 | 2.60E-09 | -2.23E-03 | 1.43E-03 | 1.20E-01 |
| rs868784 | 11 | 43944388 | G | A | 7.87E-03 | 1.42E-03 | 2.70E-08 | 2.28E-03 | 1.45E-03 | 1.20E-01 |
| rs10823826 | 10 | 53649431 | C | T | -8.98E-03 | 1.59E-03 | 1.60E-08 | -2.56E-03 | 1.62E-03 | 1.20E-01 |
| rs12885251 | 14 | 99670791 | G | A | 7.63E-03 | 1.39E-03 | 4.10E-08 | 2.13E-03 | 1.42E-03 | 1.30E-01 |
| rs10505836 | 12 | 19288508 | A | C | -1.21E-02 | 1.99E-03 | 1.10E-09 | -3.08E-03 | 2.03E-03 | 1.30E-01 |
| rs77560793 | 1 | 175001179 | G | A | 2.46E-02 | 3.99E-03 | 7.80E-10 | 6.25E-03 | 4.08E-03 | 1.30E-01 |
| rs7570446 | 2 | 193801010 | C | A | -7.80E-03 | 1.37E-03 | 1.30E-08 | 2.06E-03 | 1.40E-03 | 1.40E-01 |
| rs12477088 | 2 | 67841326 | T | C | 1.05E-02 | 1.39E-03 | 4.70E-14 | 2.08E-03 | 1.42E-03 | 1.40E-01 |
| rs2542615 | 10 | 131128952 | C | T | 8.44E-03 | 1.46E-03 | 7.30E-09 | 2.21E-03 | 1.49E-03 | 1.40E-01 |
| rs1799507 | 12 | 16427314 | G | A | -1.08E-02 | 1.96E-03 | 3.10E-08 | -2.95E-03 | 2.00E-03 | 1.40E-01 |
| rs945211 | 1 | 32191798 | G | C | -8.49E-03 | 1.41E-03 | 1.60E-09 | -2.06E-03 | 1.44E-03 | 1.50E-01 |
| rs2516726 | 16 | 2095065 | T | C | 9.91E-03 | 1.64E-03 | 1.70E-09 | 2.42E-03 | 1.68E-03 | 1.50E-01 |
| rs72753485 | 9 | 96673230 | G | C | -1.56E-02 | 2.50E-03 | 4.30E-10 | -3.68E-03 | 2.55E-03 | 1.50E-01 |
| rs1373349 | 18 | 63282992 | C | T | 9.81E-03 | 1.48E-03 | 3.50E-11 | 2.13E-03 | 1.51E-03 | 1.60E-01 |
| rs79675564 | 2 | 211286896 | C | A | -1.51E-02 | 2.55E-03 | 3.40E-09 | -3.67E-03 | 2.61E-03 | 1.60E-01 |
| rs12927792 | 16 | 9713194 | C | T | -8.67E-03 | 1.40E-03 | 5.20E-10 | -1.97E-03 | 1.43E-03 | 1.70E-01 |
| rs7264802 | 20 | 62692440 | A | G | -9.81E-03 | 1.59E-03 | 7.20E-10 | -2.22E-03 | 1.62E-03 | 1.70E-01 |
| rs12477385 | 2 | 166144850 | G | T | 9.24E-03 | 1.64E-03 | 1.80E-08 | 2.28E-03 | 1.68E-03 | 1.70E-01 |
| rs10774018 | 12 | 2157925 | G | C | -9.48E-03 | 1.66E-03 | 1.10E-08 | -2.32E-03 | 1.69E-03 | 1.70E-01 |
| rs12705894 | 7 | 113351252 | G | A | 7.80E-03 | 1.38E-03 | 1.60E-08 | 1.90E-03 | 1.41E-03 | 1.80E-01 |
| rs187067151 | 20 | 29539588 | G | T | 2.67E-02 | 3.98E-03 | 2.00E-11 | 5.44E-03 | 4.06E-03 | 1.80E-01 |
| rs403694 | 21 | 46567625 | C | T | -1.22E-02 | 1.38E-03 | 1.30E-18 | -1.86E-03 | 1.41E-03 | 1.90E-01 |
| rs7893571 | 10 | 16750129 | G | T | -1.02E-02 | 1.46E-03 | 2.90E-12 | -1.93E-03 | 1.49E-03 | 1.90E-01 |
| rs36007635 | 6 | 163009335 | G | A | 1.37E-02 | 1.99E-03 | 5.60E-12 | 2.65E-03 | 2.03E-03 | 1.90E-01 |
| rs7548936 | 1 | 91207757 | G | C | -8.48E-03 | 1.42E-03 | 2.10E-09 | 1.85E-03 | 1.45E-03 | 2.00E-01 |
| rs151252883 | 2 | 228998026 | T | G | -9.22E-03 | 1.44E-03 | 1.60E-10 | -1.91E-03 | 1.47E-03 | 2.00E-01 |
| rs183315407 | 1 | 46201427 | G | A | 2.64E-02 | 3.49E-03 | 4.30E-14 | 4.54E-03 | 3.57E-03 | 2.00E-01 |
| rs2074686 | 7 | 100800635 | G | A | 8.28E-03 | 1.39E-03 | 2.90E-09 | 1.79E-03 | 1.42E-03 | 2.10E-01 |
| rs34373881 | 3 | 20432033 | G | A | 8.83E-03 | 1.54E-03 | 9.00E-09 | 1.98E-03 | 1.57E-03 | 2.10E-01 |
| rs12644329 | 4 | 143634746 | G | A | 7.91E-03 | 1.42E-03 | 2.50E-08 | 1.78E-03 | 1.45E-03 | 2.20E-01 |
| rs7518221 | 1 | 225561346 | T | C | 7.95E-03 | 1.43E-03 | 2.80E-08 | 1.80E-03 | 1.46E-03 | 2.20E-01 |
| rs4759228 | 12 | 56508409 | G | C | 1.16E-02 | 1.51E-03 | 1.30E-14 | 1.89E-03 | 1.54E-03 | 2.20E-01 |
| rs575840515 | 1 | 80796649 | A | G | -9.01E-03 | 1.52E-03 | 3.20E-09 | -1.90E-03 | 1.56E-03 | 2.20E-01 |
| rs10973159 | 9 | 36992547 | G | T | -8.05E-03 | 1.41E-03 | 1.20E-08 | 1.74E-03 | 1.44E-03 | 2.30E-01 |
| rs10760277 | 9 | 126093999 | C | T | -8.87E-03 | 1.41E-03 | 3.60E-10 | -1.68E-03 | 1.44E-03 | 2.40E-01 |
| rs61903695 | 11 | 89922417 | A | G | -1.13E-02 | 1.58E-03 | 6.60E-13 | -1.87E-03 | 1.61E-03 | 2.40E-01 |
| rs78517245 | 3 | 42587865 | T | C | -3.44E-02 | 5.85E-03 | 4.10E-09 | -6.98E-03 | 5.98E-03 | 2.40E-01 |
| rs10805383 | 5 | 63034606 | G | A | -1.05E-02 | 1.37E-03 | 2.60E-14 | -1.57E-03 | 1.40E-03 | 2.60E-01 |
| rs9522180 | 13 | 111970212 | C | T | 9.24E-03 | 1.38E-03 | 2.40E-11 | 1.55E-03 | 1.41E-03 | 2.70E-01 |
| rs1324110 | 6 | 93913200 | G | C | 7.77E-03 | 1.38E-03 | 2.00E-08 | 1.57E-03 | 1.41E-03 | 2.70E-01 |
| rs72910629 | 6 | 69761994 | A | G | -1.33E-02 | 2.01E-03 | 4.30E-11 | 2.26E-03 | 2.05E-03 | 2.70E-01 |
| rs78369934 | 17 | 61739101 | T | C | 1.99E-02 | 3.05E-03 | 6.00E-11 | -3.44E-03 | 3.11E-03 | 2.70E-01 |
| rs78886584 | 1 | 16859325 | A | G | -8.49E-03 | 1.38E-03 | 8.70E-10 | -1.52E-03 | 1.42E-03 | 2.80E-01 |
| rs409696 | 2 | 147900651 | G | A | 1.14E-02 | 1.39E-03 | 1.80E-16 | 1.55E-03 | 1.42E-03 | 2.80E-01 |
| rs12357890 | 10 | 99762693 | A | G | -1.22E-02 | 1.39E-03 | 1.60E-18 | -1.53E-03 | 1.42E-03 | 2.80E-01 |
| rs4806814 | 19 | 1860147 | G | A | 1.27E-02 | 1.90E-03 | 3.10E-11 | -2.10E-03 | 1.94E-03 | 2.80E-01 |
| rs78508049 | 1 | 210344884 | T | C | -1.11E-02 | 1.75E-03 | 2.10E-10 | -1.88E-03 | 1.79E-03 | 2.90E-01 |
| rs55931203 | 17 | 65854602 | C | T | -1.26E-02 | 1.78E-03 | 1.60E-12 | -1.92E-03 | 1.82E-03 | 2.90E-01 |
| rs149457 | 5 | 107438057 | C | T | 1.50E-02 | 1.83E-03 | 2.10E-16 | 1.98E-03 | 1.87E-03 | 2.90E-01 |
| rs11708540 | 3 | 70593081 | G | A | -1.06E-02 | 1.89E-03 | 2.00E-08 | 2.07E-03 | 1.93E-03 | 2.90E-01 |
| rs567230078 | 6 | 43588227 | T | A | 2.51E-02 | 4.25E-03 | 3.70E-09 | 4.57E-03 | 4.34E-03 | 2.90E-01 |
| rs13292699 | 9 | 15910044 | A | C | 1.25E-02 | 1.39E-03 | 1.50E-19 | -1.47E-03 | 1.42E-03 | 3.00E-01 |
| rs7913496 | 10 | 10257277 | C | T | 1.00E-02 | 1.79E-03 | 2.00E-08 | -1.88E-03 | 1.83E-03 | 3.00E-01 |
| rs76520838 | 15 | 47916618 | C | T | -2.32E-02 | 3.87E-03 | 1.90E-09 | -4.12E-03 | 3.95E-03 | 3.00E-01 |
| rs2396625 | 7 | 113028634 | T | A | 9.94E-03 | 1.40E-03 | 1.10E-12 | 1.45E-03 | 1.43E-03 | 3.10E-01 |
| rs3806114 | 6 | 20482335 | G | A | 8.79E-03 | 1.47E-03 | 2.30E-09 | 1.51E-03 | 1.50E-03 | 3.10E-01 |
| rs116195355 | 1 | 39941508 | C | A | 2.60E-02 | 3.96E-03 | 5.90E-11 | 4.09E-03 | 4.05E-03 | 3.10E-01 |
| rs698147 | 5 | 3513485 | A | G | 8.78E-03 | 1.38E-03 | 1.80E-10 | 1.41E-03 | 1.41E-03 | 3.20E-01 |
| rs1840660 | 7 | 114352615 | G | A | -9.95E-03 | 1.41E-03 | 2.00E-12 | 1.44E-03 | 1.45E-03 | 3.20E-01 |
| rs2866720 | 7 | 70106310 | C | T | -9.13E-03 | 1.42E-03 | 1.20E-10 | -1.46E-03 | 1.45E-03 | 3.20E-01 |
| rs2269610 | 6 | 33289935 | G | C | -1.28E-02 | 1.77E-03 | 5.10E-13 | 1.78E-03 | 1.81E-03 | 3.20E-01 |
| rs4962725 | 10 | 126733321 | T | C | -9.97E-03 | 1.39E-03 | 6.60E-13 | -1.39E-03 | 1.42E-03 | 3.30E-01 |
| rs2837398 | 21 | 41427168 | A | C | -8.20E-03 | 1.40E-03 | 4.80E-09 | 1.39E-03 | 1.43E-03 | 3.30E-01 |
| rs262956 | 3 | 183486117 | T | G | 9.12E-03 | 1.44E-03 | 2.20E-10 | 1.43E-03 | 1.47E-03 | 3.30E-01 |
| rs6530737 | 8 | 14095763 | A | G | 9.02E-03 | 1.44E-03 | 3.50E-10 | 1.40E-03 | 1.47E-03 | 3.40E-01 |
| rs11079849 | 17 | 47090785 | C | T | 1.22E-02 | 1.46E-03 | 6.40E-17 | 1.42E-03 | 1.49E-03 | 3.40E-01 |
| rs35957544 | 8 | 73440371 | G | T | 1.27E-02 | 1.39E-03 | 7.40E-20 | 1.33E-03 | 1.42E-03 | 3.50E-01 |
| rs28726372 | 1 | 84353839 | T | C | -9.08E-03 | 1.48E-03 | 9.40E-10 | -1.43E-03 | 1.52E-03 | 3.50E-01 |
| rs9421249 | 10 | 118623322 | C | T | -1.04E-02 | 1.56E-03 | 2.50E-11 | 1.49E-03 | 1.60E-03 | 3.50E-01 |
| rs61217499 | 12 | 108417780 | G | C | 1.28E-02 | 1.67E-03 | 2.30E-14 | 1.60E-03 | 1.71E-03 | 3.50E-01 |
| rs57654548 | 3 | 125196904 | C | A | 1.16E-02 | 2.12E-03 | 4.20E-08 | 2.02E-03 | 2.17E-03 | 3.50E-01 |
| rs11642387 | 16 | 6753239 | A | G | 1.29E-02 | 2.29E-03 | 2.10E-08 | -2.20E-03 | 2.34E-03 | 3.50E-01 |
| rs4836133 | 5 | 124332103 | C | A | -8.36E-03 | 1.41E-03 | 3.10E-09 | 2.31E-03 | 2.47E-03 | 3.50E-01 |
| rs2164300 | 4 | 67813017 | C | T | 7.55E-03 | 1.38E-03 | 4.20E-08 | -1.26E-03 | 1.41E-03 | 3.70E-01 |
| rs1454687 | 3 | 94038085 | C | G | 1.23E-02 | 1.37E-03 | 3.00E-19 | 1.24E-03 | 1.40E-03 | 3.80E-01 |
| rs114728753 | 3 | 78483402 | A | C | -8.95E-03 | 1.47E-03 | 1.00E-09 | -1.32E-03 | 1.50E-03 | 3.80E-01 |
| rs396354 | 2 | 86850022 | T | C | 1.04E-02 | 1.52E-03 | 8.00E-12 | 1.36E-03 | 1.55E-03 | 3.80E-01 |
| rs236660 | 7 | 75050086 | T | C | -1.45E-02 | 1.44E-03 | 9.10E-24 | -1.27E-03 | 1.47E-03 | 3.90E-01 |
| rs12427047 | 12 | 90213070 | C | T | 1.09E-02 | 1.60E-03 | 8.90E-12 | 1.40E-03 | 1.63E-03 | 3.90E-01 |
| rs1547205 | 9 | 98815145 | G | C | 1.32E-02 | 2.32E-03 | 1.30E-08 | 2.02E-03 | 2.37E-03 | 3.90E-01 |
| rs1458156 | 12 | 41887940 | C | T | -9.29E-03 | 1.37E-03 | 1.30E-11 | -1.19E-03 | 1.40E-03 | 4.00E-01 |
| rs1704190 | 2 | 200760629 | G | A | -8.33E-03 | 1.42E-03 | 4.50E-09 | -1.23E-03 | 1.45E-03 | 4.00E-01 |
| rs3753639 | 1 | 154986091 | T | C | -1.15E-02 | 1.60E-03 | 7.30E-13 | -1.38E-03 | 1.64E-03 | 4.00E-01 |
| rs7539903 | 1 | 209208033 | T | A | 7.69E-03 | 1.41E-03 | 4.60E-08 | 1.18E-03 | 1.44E-03 | 4.10E-01 |
| rs7321285 | 13 | 54319327 | A | C | 1.13E-02 | 1.72E-03 | 4.10E-11 | 1.46E-03 | 1.76E-03 | 4.10E-01 |
| rs1411432 | 9 | 16728532 | A | C | -1.24E-02 | 1.77E-03 | 2.00E-12 | 1.49E-03 | 1.81E-03 | 4.10E-01 |
| rs10756555 | 9 | 14459089 | G | A | 1.03E-02 | 1.39E-03 | 1.60E-13 | 1.13E-03 | 1.42E-03 | 4.20E-01 |
| rs4419475 | 4 | 96150044 | A | T | -8.10E-03 | 1.39E-03 | 6.50E-09 | 1.16E-03 | 1.43E-03 | 4.20E-01 |
| rs9888533 | 13 | 107854612 | C | T | -7.65E-03 | 1.40E-03 | 4.50E-08 | -1.15E-03 | 1.43E-03 | 4.20E-01 |
| rs7175642 | 15 | 59450079 | T | G | 8.59E-03 | 1.47E-03 | 5.20E-09 | 1.22E-03 | 1.50E-03 | 4.20E-01 |
| rs111584879 | 15 | 66678173 | T | C | 8.79E-03 | 1.61E-03 | 4.80E-08 | 1.33E-03 | 1.64E-03 | 4.20E-01 |
| rs12630209 | 3 | 156881392 | T | G | -8.75E-03 | 1.58E-03 | 3.00E-08 | -1.27E-03 | 1.61E-03 | 4.30E-01 |
| rs12376870 | 9 | 117890567 | G | A | 8.87E-03 | 1.62E-03 | 4.10E-08 | 1.28E-03 | 1.65E-03 | 4.40E-01 |
| rs11513729 | 12 | 112273499 | C | T | 7.99E-03 | 1.41E-03 | 1.50E-08 | 1.07E-03 | 1.44E-03 | 4.60E-01 |
| rs11134512 | 5 | 167847460 | T | G | 8.24E-03 | 1.47E-03 | 1.90E-08 | 1.12E-03 | 1.50E-03 | 4.60E-01 |
| rs7928320 | 11 | 116942753 | C | G | -1.73E-02 | 2.97E-03 | 6.10E-09 | -2.23E-03 | 3.04E-03 | 4.60E-01 |
| rs473837 | 8 | 60906881 | G | T | 8.63E-03 | 1.44E-03 | 1.80E-09 | -1.01E-03 | 1.47E-03 | 4.90E-01 |
| rs11765062 | 7 | 54417515 | T | C | 7.52E-03 | 1.37E-03 | 4.60E-08 | 9.37E-04 | 1.40E-03 | 5.00E-01 |
| rs12281009 | 11 | 117032959 | A | G | -1.79E-02 | 2.95E-03 | 1.30E-09 | -2.02E-03 | 3.01E-03 | 5.00E-01 |
| rs6950388 | 7 | 1270699 | G | A | -9.45E-03 | 1.70E-03 | 2.60E-08 | 1.15E-03 | 1.74E-03 | 5.10E-01 |
| rs12538826 | 7 | 99030228 | T | C | 1.68E-02 | 2.15E-03 | 7.20E-15 | -1.43E-03 | 2.20E-03 | 5.10E-01 |
| rs7433076 | 3 | 90234502 | T | A | 9.52E-03 | 1.38E-03 | 5.10E-12 | -9.17E-04 | 1.41E-03 | 5.20E-01 |
| rs1977658 | 1 | 107607037 | T | G | 8.10E-03 | 1.45E-03 | 2.50E-08 | -9.64E-04 | 1.49E-03 | 5.20E-01 |
| rs217672 | 14 | 62361021 | A | C | -1.18E-02 | 1.55E-03 | 2.30E-14 | 1.02E-03 | 1.58E-03 | 5.20E-01 |
| rs1040046 | 6 | 83473573 | C | A | -1.13E-02 | 1.92E-03 | 4.60E-09 | -1.25E-03 | 1.96E-03 | 5.20E-01 |
| rs35390852 | 4 | 143067054 | G | A | -1.14E-02 | 2.09E-03 | 4.80E-08 | -1.33E-03 | 2.13E-03 | 5.30E-01 |
| rs4663213 | 2 | 236807893 | G | A | 9.46E-03 | 1.66E-03 | 1.20E-08 | 1.04E-03 | 1.70E-03 | 5.40E-01 |
| rs73213484 | 4 | 28489339 | A | T | 1.46E-02 | 1.97E-03 | 1.10E-13 | 1.24E-03 | 2.01E-03 | 5.40E-01 |
| rs6507054 | 18 | 31248323 | T | C | -9.28E-03 | 1.39E-03 | 2.70E-11 | 8.60E-04 | 1.42E-03 | 5.50E-01 |
| rs9462670 | 6 | 41014309 | G | C | -9.42E-03 | 1.63E-03 | 7.10E-09 | -9.86E-04 | 1.66E-03 | 5.50E-01 |
| rs13275517 | 8 | 143364521 | T | C | -8.15E-03 | 1.39E-03 | 4.10E-09 | -8.14E-04 | 1.42E-03 | 5.70E-01 |
| rs11782074 | 8 | 142617096 | G | T | -9.72E-03 | 1.43E-03 | 1.20E-11 | 8.24E-04 | 1.46E-03 | 5.70E-01 |
| rs10160769 | 11 | 76474827 | G | C | 9.18E-03 | 1.68E-03 | 4.60E-08 | -9.87E-04 | 1.72E-03 | 5.70E-01 |
| rs113079574 | 4 | 147354089 | C | T | 9.65E-03 | 1.75E-03 | 3.30E-08 | -9.93E-04 | 1.78E-03 | 5.80E-01 |
| rs10935143 | 3 | 134665159 | G | A | 8.40E-03 | 1.38E-03 | 1.10E-09 | 7.63E-04 | 1.41E-03 | 5.90E-01 |
| rs1905616 | 8 | 93235675 | G | A | 8.20E-03 | 1.46E-03 | 1.80E-08 | 8.02E-04 | 1.49E-03 | 5.90E-01 |
| rs12072739 | 1 | 98315893 | A | G | -1.16E-02 | 1.64E-03 | 1.80E-12 | -8.95E-04 | 1.68E-03 | 5.90E-01 |
| rs12037905 | 1 | 219628036 | C | T | 7.93E-03 | 1.39E-03 | 1.10E-08 | 7.49E-04 | 1.42E-03 | 6.00E-01 |
| rs8134638 | 21 | 40644170 | T | C | -7.80E-03 | 1.42E-03 | 4.00E-08 | 7.68E-04 | 1.45E-03 | 6.00E-01 |
| rs12033257 | 1 | 112318484 | A | G | 9.80E-03 | 1.42E-03 | 4.90E-12 | -7.57E-04 | 1.45E-03 | 6.00E-01 |
| rs2237402 | 7 | 39449768 | G | A | 9.23E-03 | 1.45E-03 | 1.80E-10 | 7.69E-04 | 1.48E-03 | 6.00E-01 |
| rs61985411 | 14 | 41336102 | T | A | -1.65E-02 | 2.76E-03 | 2.10E-09 | -1.47E-03 | 2.82E-03 | 6.00E-01 |
| rs809955 | 4 | 140874760 | G | A | 1.01E-02 | 1.42E-03 | 1.40E-12 | -7.30E-04 | 1.46E-03 | 6.20E-01 |
| rs8192675 | 3 | 170724883 | T | C | -1.17E-02 | 1.51E-03 | 8.70E-15 | -7.69E-04 | 1.54E-03 | 6.20E-01 |
| rs7331420 | 13 | 99236471 | G | A | 9.14E-03 | 1.53E-03 | 2.10E-09 | -7.63E-04 | 1.56E-03 | 6.20E-01 |
| rs1017529 | 17 | 27912415 | C | A | -1.06E-02 | 1.84E-03 | 7.70E-09 | -9.30E-04 | 1.88E-03 | 6.20E-01 |
| rs2576135 | 13 | 54691442 | T | A | 1.32E-02 | 2.39E-03 | 3.50E-08 | 1.21E-03 | 2.44E-03 | 6.20E-01 |
| rs8011566 | 14 | 42939471 | T | A | -8.73E-03 | 1.39E-03 | 3.50E-10 | -6.89E-04 | 1.42E-03 | 6.30E-01 |
| rs39674 | 16 | 9413210 | C | G | -8.71E-03 | 1.50E-03 | 6.00E-09 | -7.29E-04 | 1.53E-03 | 6.30E-01 |
| rs2433733 | 2 | 230816703 | G | A | 1.05E-02 | 1.46E-03 | 7.30E-13 | 6.91E-04 | 1.50E-03 | 6.40E-01 |
| rs36131051 | 3 | 107888841 | T | G | 1.08E-02 | 1.71E-03 | 2.90E-10 | 8.28E-04 | 1.75E-03 | 6.40E-01 |
| rs112380819 | 3 | 9498519 | G | A | -1.37E-02 | 2.26E-03 | 1.30E-09 | -1.05E-03 | 2.31E-03 | 6.50E-01 |
| rs147730268 | 12 | 123024476 | G | T | 2.28E-02 | 2.49E-03 | 4.00E-20 | -1.15E-03 | 2.54E-03 | 6.50E-01 |
| rs62259692 | 3 | 51847709 | G | A | -1.78E-02 | 2.93E-03 | 1.40E-09 | 1.34E-03 | 3.00E-03 | 6.50E-01 |
| rs13186637 | 5 | 153108558 | T | C | 8.68E-03 | 1.44E-03 | 1.70E-09 | -6.46E-04 | 1.47E-03 | 6.60E-01 |
| rs9395520 | 6 | 13183523 | C | T | 9.76E-03 | 1.49E-03 | 5.90E-11 | 6.65E-04 | 1.52E-03 | 6.60E-01 |
| rs10927006 | 1 | 243557659 | T | C | 1.20E-02 | 1.95E-03 | 8.30E-10 | 8.84E-04 | 2.00E-03 | 6.60E-01 |
| rs10204994 | 2 | 35443726 | G | A | 1.01E-02 | 1.63E-03 | 5.30E-10 | 7.02E-04 | 1.66E-03 | 6.70E-01 |
| rs10499014 | 6 | 97947755 | C | G | 1.00E-02 | 1.56E-03 | 1.10E-10 | -6.65E-04 | 1.59E-03 | 6.80E-01 |
| rs533493779 | 2 | 104447054 | A | T | -8.69E-03 | 1.42E-03 | 1.10E-09 | -5.76E-04 | 1.46E-03 | 6.90E-01 |
| rs4660586 | 1 | 42407229 | C | T | 8.96E-03 | 1.56E-03 | 1.00E-08 | -6.28E-04 | 1.60E-03 | 6.90E-01 |
| rs7020196 | 9 | 12289527 | C | T | 7.79E-03 | 1.41E-03 | 3.60E-08 | 5.52E-04 | 1.44E-03 | 7.00E-01 |
| rs1865341 | 9 | 8845911 | C | T | -9.00E-03 | 1.62E-03 | 2.50E-08 | -6.28E-04 | 1.65E-03 | 7.00E-01 |
| rs12681792 | 8 | 62054463 | C | A | -9.56E-03 | 1.75E-03 | 4.30E-08 | -6.92E-04 | 1.78E-03 | 7.00E-01 |
| rs59227842 | 11 | 43692423 | A | G | -1.52E-02 | 1.49E-03 | 1.80E-24 | -5.69E-04 | 1.53E-03 | 7.10E-01 |
| rs13427822 | 2 | 213414265 | A | G | 9.45E-03 | 1.56E-03 | 1.30E-09 | -5.84E-04 | 1.59E-03 | 7.10E-01 |
| rs270689 | 6 | 104790532 | A | T | -1.01E-02 | 1.72E-03 | 4.20E-09 | -6.49E-04 | 1.76E-03 | 7.10E-01 |
| rs6478538 | 9 | 124627012 | A | G | 8.81E-03 | 1.47E-03 | 1.90E-09 | -5.27E-04 | 1.50E-03 | 7.30E-01 |
| rs2616143 | 8 | 20632022 | G | A | 8.85E-03 | 1.47E-03 | 1.90E-09 | 5.15E-04 | 1.51E-03 | 7.30E-01 |
| rs72976986 | 19 | 4050424 | G | A | 1.39E-02 | 1.77E-03 | 4.40E-15 | 6.31E-04 | 1.80E-03 | 7.30E-01 |
| rs9515446 | 13 | 112217108 | A | G | -9.69E-03 | 1.38E-03 | 2.20E-12 | -4.59E-04 | 1.41E-03 | 7.40E-01 |
| rs4759073 | 12 | 54653258 | G | A | 9.35E-03 | 1.39E-03 | 1.90E-11 | 4.35E-04 | 1.42E-03 | 7.60E-01 |
| rs10169594 | 2 | 41637688 | T | C | -7.88E-03 | 1.43E-03 | 3.30E-08 | -4.55E-04 | 1.46E-03 | 7.60E-01 |
| rs2744801 | 1 | 41155486 | C | T | 7.95E-03 | 1.45E-03 | 4.30E-08 | 4.44E-04 | 1.48E-03 | 7.60E-01 |
| rs149778057 | 13 | 31007805 | A | C | 9.05E-03 | 1.53E-03 | 3.80E-09 | 4.37E-04 | 1.57E-03 | 7.80E-01 |
| rs1320251 | 17 | 21264396 | C | T | 1.24E-02 | 1.38E-03 | 3.20E-19 | -3.69E-04 | 1.41E-03 | 7.90E-01 |
| rs7255223 | 19 | 32824310 | C | A | 8.97E-03 | 1.56E-03 | 8.50E-09 | 4.27E-04 | 1.59E-03 | 7.90E-01 |
| rs60497719 | 19 | 33971746 | G | A | -9.67E-03 | 1.58E-03 | 1.00E-09 | -4.30E-04 | 1.62E-03 | 7.90E-01 |
| rs4077093 | 12 | 51593616 | T | G | 9.69E-03 | 1.68E-03 | 7.90E-09 | 4.66E-04 | 1.72E-03 | 7.90E-01 |
| rs11691869 | 2 | 100805996 | C | A | 1.12E-02 | 1.43E-03 | 3.50E-15 | -3.65E-04 | 1.46E-03 | 8.00E-01 |
| rs10779835 | 1 | 230299949 | T | C | 8.01E-03 | 1.41E-03 | 1.20E-08 | 3.37E-04 | 1.44E-03 | 8.10E-01 |
| rs12971645 | 19 | 45807945 | G | A | 8.59E-03 | 1.54E-03 | 2.60E-08 | 3.43E-04 | 1.58E-03 | 8.30E-01 |
| rs2237025 | 4 | 55541879 | T | C | 1.02E-02 | 1.39E-03 | 1.80E-13 | -2.88E-04 | 1.42E-03 | 8.40E-01 |
| rs9529148 | 13 | 67419495 | G | A | -8.00E-03 | 1.42E-03 | 1.90E-08 | -2.79E-04 | 1.45E-03 | 8.50E-01 |
| rs3759584 | 14 | 103990799 | T | C | 9.74E-03 | 1.43E-03 | 1.10E-11 | 2.76E-04 | 1.46E-03 | 8.50E-01 |
| rs7601895 | 2 | 55281901 | C | G | 1.03E-02 | 1.49E-03 | 4.10E-12 | 2.78E-04 | 1.52E-03 | 8.50E-01 |
| rs7498044 | 15 | 92573639 | G | A | 9.95E-03 | 1.68E-03 | 3.20E-09 | -3.19E-04 | 1.72E-03 | 8.50E-01 |
| rs4916229 | 1 | 171443368 | C | G | -1.51E-02 | 2.33E-03 | 8.90E-11 | 3.77E-04 | 2.39E-03 | 8.70E-01 |
| rs35852935 | 4 | 17991522 | A | C | -2.19E-02 | 3.74E-03 | 4.90E-09 | 6.39E-04 | 3.82E-03 | 8.70E-01 |
| rs13104584 | 4 | 80811227 | G | A | -8.64E-03 | 1.40E-03 | 6.20E-10 | -1.93E-04 | 1.43E-03 | 8.90E-01 |
| rs4500770 | 16 | 74658430 | A | T | 7.82E-03 | 1.43E-03 | 4.30E-08 | 1.96E-04 | 1.46E-03 | 8.90E-01 |
| rs71495049 | 10 | 34014435 | G | A | -1.71E-02 | 2.48E-03 | 5.70E-12 | -3.46E-04 | 2.53E-03 | 8.90E-01 |
| rs1229984 | 4 | 100239319 | T | C | -2.30E-02 | 4.16E-03 | 3.20E-08 | 5.90E-04 | 4.26E-03 | 8.90E-01 |
| rs113706999 | 3 | 44159156 | T | A | -2.88E-02 | 4.73E-03 | 1.10E-09 | -6.03E-04 | 4.84E-03 | 9.00E-01 |
| rs58351927 | 17 | 5297038 | A | G | -1.01E-02 | 1.50E-03 | 1.20E-11 | -1.67E-04 | 1.53E-03 | 9.10E-01 |
| rs113569731 | 3 | 47093206 | C | A | -1.52E-02 | 2.41E-03 | 3.10E-10 | -2.93E-04 | 2.47E-03 | 9.10E-01 |
| rs61971082 | 13 | 86494667 | T | G | -1.01E-02 | 1.52E-03 | 3.00E-11 | 1.62E-04 | 1.56E-03 | 9.20E-01 |
| rs9477762 | 6 | 18507853 | A | T | -2.02E-02 | 3.05E-03 | 3.80E-11 | 2.72E-04 | 3.12E-03 | 9.30E-01 |
| rs10185199 | 2 | 40282202 | G | A | 1.00E-02 | 1.56E-03 | 1.60E-10 | 1.00E-04 | 1.60E-03 | 9.50E-01 |
| rs4658403 | 1 | 243832560 | C | T | 1.36E-02 | 1.84E-03 | 1.20E-13 | 9.35E-05 | 1.88E-03 | 9.60E-01 |
| rs80236973 | 3 | 188001014 | C | T | 1.22E-02 | 2.01E-03 | 1.40E-09 | 9.83E-05 | 2.05E-03 | 9.60E-01 |
| rs4425224 | 3 | 56249398 | C | A | 1.24E-02 | 2.24E-03 | 2.70E-08 | -1.19E-04 | 2.29E-03 | 9.60E-01 |
| rs181617194 | 12 | 122011598 | T | C | 2.27E-02 | 3.76E-03 | 1.70E-09 | 1.22E-04 | 3.84E-03 | 9.70E-01 |
| rs28408562 | 15 | 60917079 | C | G | -7.84E-03 | 1.38E-03 | 1.30E-08 | 4.07E-05 | 1.41E-03 | 9.80E-01 |
| rs7206608 | 16 | 82872628 | C | G | -9.79E-03 | 1.47E-03 | 2.60E-11 | 4.55E-05 | 1.50E-03 | 9.80E-01 |
| rs12253527 | 10 | 21819824 | G | A | -1.36E-02 | 1.47E-03 | 2.00E-20 | -9.61E-06 | 1.50E-03 | 9.90E-01 |
|  |  |  |  |  |  |  |  |  |  |  |
| ** Genetic variants strongly associated with childhood adiposity at genome wide significance and not adulthood adiposity (exclude adult SNPs at P ≤ 0.05) were counted. We then divided 0.05 by this number to generate the Bonferroni corrected P value. | | | | | | | | | | |

**Table S2J.** The genetic variants strongly associated with adulthood adiposity at genome wide significance and not childhood adiposity (exclude child SNPs at P ≤ 0.05 with Bonferroni correction**) in females.

| SNP | Chromosome | Base position | Effect allele | Other allele | Beta (Age 10) | SE (Age 10) | P (Age 10) | Beta (Adult) | SE (Adult) | P (Adult) |
| --- | --- | --- | --- | --- | --- | --- | --- | --- | --- | --- |
| rs74892851 | 1 | 1563789 | C | A | 1.22E-04 | 2.03E-03 | 9.70E-01 | 1.16E-02 | 2.06E-03 | 1.90E-08 |
| rs78886584 | 1 | 16859325 | A | G | -2.12E-03 | 1.96E-03 | 3.20E-01 | -1.33E-02 | 1.99E-03 | 2.60E-11 |
| rs72660086 | 1 | 39571992 | T | G | -3.11E-03 | 2.37E-03 | 1.90E-01 | -1.33E-02 | 2.41E-03 | 3.20E-08 |
| rs36017365 | 1 | 47682889 | C | CA | 6.64E-03 | 2.08E-03 | 1.70E-03 | 1.29E-02 | 2.12E-03 | 9.30E-10 |
| rs749593242 | 1 | 50224384 | C | CT | 2.39E-03 | 2.08E-03 | 1.70E-01 | 1.28E-02 | 2.11E-03 | 1.30E-09 |
| rs56951135 | 1 | 91211018 | A | ATTT | 3.91E-04 | 2.04E-03 | 8.30E-01 | -1.43E-02 | 2.07E-03 | 4.90E-12 |
| rs653958 | 1 | 96884006 | A | G | -5.19E-03 | 2.00E-03 | 1.00E-02 | -1.16E-02 | 2.04E-03 | 1.10E-08 |
| rs75641275 | 1 | 98327133 | A | C | 2.06E-03 | 2.76E-03 | 5.90E-01 | -1.85E-02 | 2.81E-03 | 4.70E-11 |
| rs12033257 | 1 | 112318484 | A | G | -3.09E-04 | 2.00E-03 | 6.80E-01 | 1.22E-02 | 2.04E-03 | 1.90E-09 |
| rs3753639 | 1 | 154986091 | T | C | 7.79E-04 | 2.26E-03 | 8.90E-01 | -1.26E-02 | 2.30E-03 | 3.70E-08 |
| rs61813324 | 1 | 156049877 | C | T | -4.74E-03 | 2.87E-03 | 8.10E-02 | -2.09E-02 | 2.91E-03 | 7.60E-13 |
| rs2678204 | 1 | 201800511 | T | G | -5.56E-03 | 2.04E-03 | 6.10E-03 | -1.34E-02 | 2.07E-03 | 1.20E-10 |
| rs2994320 | 1 | 243641247 | A | G | -2.73E-04 | 2.45E-03 | 7.90E-01 | 1.66E-02 | 2.49E-03 | 2.40E-11 |
| rs34606703 | 2 | 47014522 | G | A | 5.03E-03 | 2.02E-03 | 1.80E-02 | 1.17E-02 | 2.06E-03 | 1.10E-08 |
| rs13420048 | 2 | 50751414 | C | A | 6.99E-04 | 2.01E-03 | 5.80E-01 | 1.20E-02 | 2.04E-03 | 4.40E-09 |
| rs6545468 | 2 | 55277641 | C | G | 2.20E-03 | 1.97E-03 | 2.10E-01 | 1.20E-02 | 2.00E-03 | 2.20E-09 |
| rs4671328 | 2 | 58935282 | T | G | 1.65E-03 | 1.96E-03 | 3.20E-01 | 1.37E-02 | 1.99E-03 | 6.50E-12 |
| rs13416992 | 2 | 59298298 | A | C | 4.40E-03 | 1.98E-03 | 4.30E-02 | 1.48E-02 | 2.01E-03 | 1.90E-13 |
| rs10192894 | 2 | 62838936 | A | G | 1.93E-03 | 1.95E-03 | 3.00E-01 | -1.10E-02 | 1.98E-03 | 2.50E-08 |
| rs12477088 | 2 | 67841326 | T | C | 1.12E-03 | 1.96E-03 | 6.20E-01 | 1.19E-02 | 1.99E-03 | 2.70E-09 |
| rs11691869 | 2 | 100805996 | C | A | 1.03E-03 | 2.02E-03 | 6.30E-01 | 1.55E-02 | 2.05E-03 | 3.30E-14 |
| rs113607259 | 2 | 104343481 | G | GTA | 2.80E-03 | 1.95E-03 | 1.40E-01 | 1.18E-02 | 1.99E-03 | 2.60E-09 |
| rs7602120 | 2 | 144033069 | C | T | -2.71E-03 | 1.95E-03 | 1.50E-01 | -1.34E-02 | 1.98E-03 | 1.20E-11 |
| rs1083472 | 2 | 147873492 | C | G | -8.32E-04 | 1.99E-03 | 8.00E-01 | 1.13E-02 | 2.02E-03 | 2.10E-08 |
| rs758369774 | 2 | 181562177 | AT | A | -5.55E-03 | 2.01E-03 | 4.50E-03 | -1.23E-02 | 2.04E-03 | 1.50E-09 |
| rs4482463 | 2 | 205375909 | C | A | 6.82E-03 | 3.64E-03 | 3.20E-02 | 2.18E-02 | 3.70E-03 | 3.70E-09 |
| rs4673553 | 2 | 211608379 | T | G | -2.48E-03 | 1.95E-03 | 2.20E-01 | -1.17E-02 | 1.98E-03 | 3.20E-09 |
| rs573105257 | 2 | 230822932 | A | AT | 1.19E-03 | 2.08E-03 | 5.20E-01 | 1.18E-02 | 2.11E-03 | 2.20E-08 |
| rs62242071 | 3 | 20590313 | G | T | 2.93E-03 | 2.08E-03 | 1.70E-01 | 1.18E-02 | 2.11E-03 | 2.30E-08 |
| rs113706999 | 3 | 44159156 | T | A | -5.99E-03 | 6.65E-03 | 3.10E-01 | -3.87E-02 | 6.76E-03 | 1.00E-08 |
| rs9843653 | 3 | 49920571 | T | C | -3.97E-03 | 1.94E-03 | 4.30E-02 | -1.76E-02 | 1.97E-03 | 4.20E-19 |
| rs6774533 | 3 | 62471086 | C | T | -5.62E-03 | 2.16E-03 | 1.40E-02 | -1.22E-02 | 2.19E-03 | 2.80E-08 |
| rs34234711 | 3 | 82709447 | G | T | -3.99E-03 | 2.01E-03 | 3.90E-02 | -1.21E-02 | 2.04E-03 | 3.20E-09 |
| rs1454687 | 3 | 94038085 | C | G | 1.89E-03 | 1.93E-03 | 4.00E-01 | 1.23E-02 | 1.97E-03 | 4.10E-10 |
| rs9811252 | 3 | 128293392 | C | T | 1.04E-03 | 2.03E-03 | 8.10E-01 | 1.14E-02 | 2.07E-03 | 3.90E-08 |
| rs13081671 | 3 | 135876549 | C | T | -6.69E-04 | 2.18E-03 | 7.20E-01 | -1.35E-02 | 2.22E-03 | 1.20E-09 |
| rs529200 | 3 | 173114305 | A | G | -3.02E-03 | 1.94E-03 | 1.90E-01 | -1.16E-02 | 1.97E-03 | 4.00E-09 |
| rs73052033 | 3 | 185828465 | T | C | 6.67E-03 | 2.50E-03 | 4.50E-03 | 1.72E-02 | 2.54E-03 | 1.10E-11 |
| rs61218008 | 3 | 194881130 | A | G | 5.35E-03 | 2.17E-03 | 1.30E-02 | 1.21E-02 | 2.21E-03 | 4.70E-08 |
| rs148928878 | 4 | 18548257 | G | GGTGT | -4.57E-03 | 1.95E-03 | 1.30E-02 | -1.12E-02 | 1.98E-03 | 1.60E-08 |
| rs9684942 | 4 | 20233035 | G | A | -5.57E-03 | 2.74E-03 | 4.60E-02 | -1.53E-02 | 2.79E-03 | 3.80E-08 |
| rs73213484 | 4 | 28489339 | A | T | 2.48E-03 | 2.78E-03 | 4.00E-01 | 1.89E-02 | 2.83E-03 | 2.30E-11 |
| rs148712344 | 4 | 55476318 | G | T | 7.04E-03 | 5.02E-03 | 1.50E-01 | 2.98E-02 | 5.10E-03 | 5.10E-09 |
| rs925422 | 4 | 60254101 | T | G | 5.74E-03 | 2.22E-03 | 1.50E-02 | 1.25E-02 | 2.26E-03 | 2.70E-08 |
| rs1603179 | 4 | 67805347 | A | C | -3.60E-03 | 2.03E-03 | 9.20E-02 | 1.14E-02 | 2.06E-03 | 2.90E-08 |
| rs11098965 | 4 | 80888040 | C | T | 6.51E-04 | 2.22E-03 | 7.00E-01 | 1.36E-02 | 2.26E-03 | 1.60E-09 |
| rs2199936 | 4 | 89045331 | A | G | -6.25E-03 | 3.05E-03 | 5.20E-02 | -1.83E-02 | 3.10E-03 | 3.30E-09 |
| rs182851732 | 4 | 90684766 | A | G | 1.31E-02 | 1.18E-02 | 2.90E-01 | 6.96E-02 | 1.20E-02 | 7.70E-09 |
| rs769668 | 4 | 140858717 | T | C | 1.97E-04 | 2.04E-03 | 9.70E-01 | 1.40E-02 | 2.08E-03 | 1.80E-11 |
| rs35390852 | 4 | 143067054 | G | A | -2.05E-03 | 2.94E-03 | 4.10E-01 | -1.67E-02 | 2.99E-03 | 2.40E-08 |
| rs828550 | 5 | 3539923 | C | T | -1.56E-03 | 2.04E-03 | 4.30E-01 | -1.17E-02 | 2.07E-03 | 1.50E-08 |
| rs55908499 | 5 | 63020950 | G | GA | -2.55E-03 | 1.98E-03 | 2.00E-01 | -1.41E-02 | 2.01E-03 | 2.20E-12 |
| rs59893724 | 5 | 80830788 | A | G | 6.30E-03 | 2.25E-03 | 4.30E-03 | 1.28E-02 | 2.29E-03 | 2.40E-08 |
| rs35843836 | 5 | 88798726 | A | T | -5.26E-03 | 2.01E-03 | 4.90E-03 | -1.18E-02 | 2.04E-03 | 7.60E-09 |
| rs191621046 | 5 | 92547517 | G | A | -5.60E-03 | 5.06E-03 | 2.20E-01 | -2.98E-02 | 5.15E-03 | 7.10E-09 |
| rs10623997 | 5 | 107478679 | T | TATAATA | -3.07E-04 | 2.34E-03 | 9.10E-01 | 1.80E-02 | 2.38E-03 | 3.60E-14 |
| rs1366334 | 5 | 122683163 | C | G | 6.11E-03 | 2.13E-03 | 1.90E-03 | 1.18E-02 | 2.17E-03 | 4.70E-08 |
| rs71579590 | 5 | 139070398 | G | C | -3.73E-03 | 2.74E-03 | 1.80E-01 | -1.88E-02 | 2.79E-03 | 1.70E-11 |
| rs251353 | 5 | 140228164 | C | A | 2.52E-03 | 2.04E-03 | 1.50E-01 | 1.19E-02 | 2.07E-03 | 1.10E-08 |
| rs12658841 | 5 | 153106013 | C | G | -1.18E-03 | 1.95E-03 | 7.80E-01 | 1.17E-02 | 1.98E-03 | 3.80E-09 |
| rs9395520 | 6 | 13183523 | C | T | 1.09E-03 | 2.10E-03 | 5.80E-01 | 1.25E-02 | 2.14E-03 | 4.60E-09 |
| rs77253887 | 6 | 35626932 | G | C | 1.41E-03 | 5.40E-03 | 7.90E-01 | -3.03E-02 | 5.49E-03 | 3.50E-08 |
| rs567230078 | 6 | 43588227 | T | A | 8.43E-03 | 5.99E-03 | 2.40E-01 | 3.40E-02 | 6.09E-03 | 2.50E-08 |
| rs9387640 | 6 | 119508871 | C | T | 3.06E-03 | 2.01E-03 | 1.30E-01 | 1.18E-02 | 2.05E-03 | 9.20E-09 |
| rs73046311 | 7 | 1854159 | C | G | 1.70E-03 | 2.65E-03 | 5.40E-01 | 1.57E-02 | 2.69E-03 | 5.70E-09 |
| rs2866720 | 7 | 70106310 | C | T | -1.60E-03 | 2.00E-03 | 3.10E-01 | -1.18E-02 | 2.04E-03 | 7.00E-09 |
| rs236660 | 7 | 75050086 | T | C | -2.89E-03 | 2.03E-03 | 1.50E-01 | -1.49E-02 | 2.06E-03 | 5.40E-13 |
| rs369428586 | 7 | 99134799 | CA | C | -2.52E-03 | 2.73E-03 | 4.00E-01 | 1.56E-02 | 2.78E-03 | 2.00E-08 |
| rs2396625 | 7 | 113028634 | T | A | 3.73E-04 | 1.97E-03 | 7.10E-01 | 1.18E-02 | 2.00E-03 | 3.40E-09 |
| rs1840661 | 7 | 114352682 | T | A | 3.18E-03 | 1.97E-03 | 1.50E-01 | -1.22E-02 | 2.00E-03 | 1.20E-09 |
| rs6601415 | 8 | 9976805 | C | A | -6.18E-03 | 1.95E-03 | 1.20E-03 | -1.11E-02 | 1.98E-03 | 2.30E-08 |
| rs6557829 | 8 | 21973970 | C | A | 1.90E-03 | 1.97E-03 | 3.10E-01 | -1.16E-02 | 2.00E-03 | 6.90E-09 |
| rs117176448 | 8 | 27261138 | C | G | -4.48E-03 | 3.28E-03 | 1.80E-01 | -1.87E-02 | 3.34E-03 | 2.00E-08 |
| rs10957605 | 8 | 73433886 | C | T | 8.68E-04 | 2.08E-03 | 8.10E-01 | 1.58E-02 | 2.12E-03 | 9.80E-14 |
| rs17716502 | 8 | 116659731 | C | T | 2.81E-03 | 2.42E-03 | 1.90E-01 | 1.79E-02 | 2.46E-03 | 3.50E-13 |
| rs4740442 | 9 | 10153245 | C | T | -4.50E-03 | 2.15E-03 | 3.10E-02 | -1.21E-02 | 2.18E-03 | 2.60E-08 |
| rs13292699 | 9 | 15910044 | A | C | -2.66E-03 | 1.96E-03 | 1.90E-01 | 1.50E-02 | 1.99E-03 | 4.30E-14 |
| rs10962552 | 9 | 16723742 | C | T | -1.73E-03 | 2.61E-03 | 5.20E-01 | -1.46E-02 | 2.65E-03 | 3.60E-08 |
| rs377741138 | 9 | 28412183 | GAAAA | G | -2.70E-03 | 2.05E-03 | 2.20E-01 | -1.61E-02 | 2.09E-03 | 1.10E-14 |
| rs6478538 | 9 | 124627012 | A | G | 6.79E-04 | 2.07E-03 | 5.70E-01 | 1.15E-02 | 2.10E-03 | 4.60E-08 |
| rs3003578 | 9 | 130994179 | C | T | 2.25E-03 | 1.96E-03 | 2.10E-01 | 1.12E-02 | 2.00E-03 | 1.80E-08 |
| rs1270799 | 10 | 21907423 | T | G | -2.76E-03 | 2.11E-03 | 1.70E-01 | -1.65E-02 | 2.14E-03 | 1.20E-14 |
| rs113585475 | 10 | 33985434 | C | T | 1.87E-03 | 3.27E-03 | 4.70E-01 | 2.20E-02 | 3.32E-03 | 3.60E-11 |
| rs3125326 | 10 | 63053788 | A | C | -4.42E-03 | 2.00E-03 | 2.50E-02 | -1.11E-02 | 2.04E-03 | 4.70E-08 |
| rs7090758 | 10 | 65335315 | T | C | -5.72E-03 | 1.94E-03 | 3.70E-03 | -1.29E-02 | 1.97E-03 | 5.70E-11 |
| rs562044398 | 10 | 76101122 | C | CA | -6.63E-03 | 2.91E-03 | 1.70E-02 | -1.68E-02 | 2.96E-03 | 1.50E-08 |
| rs1250535 | 10 | 81016112 | C | G | 2.24E-03 | 2.08E-03 | 2.20E-01 | -1.19E-02 | 2.11E-03 | 1.80E-08 |
| rs12357890 | 10 | 99762693 | A | G | 6.41E-04 | 1.96E-03 | 7.90E-01 | -1.48E-02 | 1.99E-03 | 1.20E-13 |
| rs10510025 | 10 | 118650996 | C | T | 2.28E-04 | 2.25E-03 | 7.90E-01 | -1.33E-02 | 2.29E-03 | 6.90E-09 |
| rs141090474 | 10 | 126729168 | TAC | T | 3.21E-03 | 2.00E-03 | 1.00E-01 | 1.15E-02 | 2.03E-03 | 1.40E-08 |
| rs201102222 | 10 | 134005569 | A | AT | 2.02E-03 | 1.96E-03 | 3.60E-01 | 1.15E-02 | 1.99E-03 | 7.60E-09 |
| rs201233808 | 11 | 867599 | C | CCAT | 4.92E-03 | 2.16E-03 | 3.80E-02 | 1.24E-02 | 2.20E-03 | 2.00E-08 |
| rs11022766 | 11 | 13348249 | T | G | 6.71E-03 | 2.03E-03 | 1.10E-03 | 1.38E-02 | 2.07E-03 | 2.50E-11 |
| rs60572790 | 11 | 43692383 | T | C | -1.62E-03 | 2.09E-03 | 5.20E-01 | -1.65E-02 | 2.12E-03 | 7.20E-15 |
| rs34292685 | 11 | 64049021 | C | T | 4.46E-03 | 2.63E-03 | 8.00E-02 | 1.80E-02 | 2.67E-03 | 1.60E-11 |
| rs11218510 | 11 | 121922587 | G | A | 2.91E-03 | 1.98E-03 | 2.00E-01 | 1.12E-02 | 2.01E-03 | 2.90E-08 |
| rs199569565 | 11 | 130749351 | TAG | T | -6.34E-03 | 1.94E-03 | 1.70E-03 | -1.11E-02 | 1.98E-03 | 1.70E-08 |
| rs11223204 | 11 | 132652554 | A | G | -4.88E-03 | 1.96E-03 | 1.50E-02 | -1.14E-02 | 1.99E-03 | 1.10E-08 |
| rs7976757 | 12 | 19207948 | T | C | -3.22E-03 | 2.57E-03 | 2.70E-01 | -1.52E-02 | 2.62E-03 | 6.10E-09 |
| rs2292238 | 12 | 56493822 | A | C | 1.21E-03 | 1.97E-03 | 4.40E-01 | 1.22E-02 | 2.01E-03 | 1.30E-09 |
| rs770082 | 12 | 89776485 | G | A | -4.23E-03 | 1.96E-03 | 4.40E-02 | -1.34E-02 | 1.99E-03 | 2.10E-11 |
| rs34697864 | 12 | 103680717 | C | CA | 7.51E-03 | 2.32E-03 | 1.30E-03 | 1.38E-02 | 2.36E-03 | 5.60E-09 |
| rs61217499 | 12 | 108417780 | G | C | 1.78E-03 | 2.35E-03 | 5.30E-01 | 1.48E-02 | 2.40E-03 | 5.70E-10 |
| rs10849900 | 12 | 110974890 | T | C | 4.16E-03 | 2.09E-03 | 4.30E-02 | 1.20E-02 | 2.13E-03 | 1.60E-08 |
| rs111828690 | 12 | 117576767 | C | T | -1.01E-03 | 2.35E-03 | 6.50E-01 | -1.37E-02 | 2.39E-03 | 1.00E-08 |
| rs181617194 | 12 | 122011598 | T | C | -4.02E-03 | 5.30E-03 | 4.50E-01 | 3.17E-02 | 5.39E-03 | 4.20E-09 |
| rs3803005 | 12 | 123110654 | T | C | -2.78E-03 | 2.18E-03 | 1.90E-01 | -1.68E-02 | 2.22E-03 | 3.60E-14 |
| rs9579775 | 13 | 20616557 | A | C | -5.99E-03 | 2.94E-03 | 3.70E-02 | -1.92E-02 | 2.99E-03 | 1.40E-10 |
| rs1933440 | 13 | 28676971 | A | C | -6.27E-03 | 2.64E-03 | 1.10E-02 | -1.53E-02 | 2.69E-03 | 1.20E-08 |
| rs11415560 | 13 | 32996332 | C | CA | -4.94E-03 | 2.03E-03 | 1.80E-02 | -1.16E-02 | 2.07E-03 | 2.10E-08 |
| rs776543236 | 13 | 67348551 | ATGGAG | A | 6.04E-04 | 2.29E-03 | 7.90E-01 | -1.44E-02 | 2.33E-03 | 6.50E-10 |
| rs116394958 | 13 | 86477072 | C | T | -9.39E-05 | 2.17E-03 | 9.90E-01 | -1.26E-02 | 2.21E-03 | 1.40E-08 |
| rs7331420 | 13 | 99236471 | G | A | -2.37E-04 | 2.15E-03 | 8.40E-01 | 1.20E-02 | 2.19E-03 | 4.80E-08 |
| rs9522180 | 13 | 111970212 | C | T | 2.22E-03 | 1.95E-03 | 3.00E-01 | 1.08E-02 | 1.98E-03 | 4.40E-08 |
| rs367552155 | 14 | 29721943 | CA | C | 1.78E-03 | 2.29E-03 | 4.90E-01 | 1.44E-02 | 2.33E-03 | 7.40E-10 |
| rs75104958 | 14 | 33269269 | A | C | -7.87E-03 | 2.41E-03 | 1.10E-03 | -1.57E-02 | 2.45E-03 | 1.30E-10 |
| rs10142359 | 14 | 73884540 | A | G | -2.28E-03 | 1.94E-03 | 2.20E-01 | -1.11E-02 | 1.97E-03 | 1.70E-08 |
| rs8022132 | 14 | 79955864 | A | T | -4.02E-03 | 2.11E-03 | 3.80E-02 | -1.46E-02 | 2.15E-03 | 1.00E-11 |
| rs6575340 | 14 | 94023972 | G | A | -4.92E-03 | 2.02E-03 | 7.20E-03 | -1.46E-02 | 2.05E-03 | 1.10E-12 |
| rs11374426 | 14 | 104337630 | G | GA | 2.28E-03 | 2.04E-03 | 2.70E-01 | 1.36E-02 | 2.08E-03 | 6.90E-11 |
| rs1466276 | 15 | 52025950 | C | G | 4.68E-03 | 1.95E-03 | 2.90E-02 | 1.11E-02 | 1.98E-03 | 1.90E-08 |
| rs67962220 | 15 | 74188926 | T | G | -8.14E-03 | 2.58E-03 | 1.50E-03 | -1.51E-02 | 2.63E-03 | 9.80E-09 |
| rs715724 | 15 | 80984293 | A | G | 3.43E-03 | 2.03E-03 | 5.70E-02 | 1.17E-02 | 2.06E-03 | 1.40E-08 |
| rs939624 | 15 | 99480551 | C | T | -3.03E-04 | 1.94E-03 | 9.70E-01 | -1.23E-02 | 1.98E-03 | 4.70E-10 |
| rs7200589 | 16 | 349331 | G | A | 4.56E-03 | 2.18E-03 | 3.00E-02 | 1.56E-02 | 2.22E-03 | 2.00E-12 |
| rs13329943 | 16 | 24733751 | C | T | -3.48E-03 | 2.19E-03 | 1.40E-01 | -1.28E-02 | 2.22E-03 | 7.90E-09 |
| rs3814883 | 16 | 29994922 | C | T | -6.32E-03 | 1.94E-03 | 1.90E-03 | -1.50E-02 | 1.98E-03 | 3.50E-14 |
| rs34898535 | 16 | 31025641 | C | T | 6.97E-03 | 1.99E-03 | 1.10E-03 | 1.92E-02 | 2.03E-03 | 2.80E-21 |
| rs4790292 | 17 | 1824305 | C | A | 9.33E-03 | 2.69E-03 | 9.60E-04 | 1.75E-02 | 2.74E-03 | 1.80E-10 |
| rs55678940 | 17 | 21251092 | C | CTGTAAAGAAA | 7.31E-04 | 2.04E-03 | 6.60E-01 | 1.38E-02 | 2.08E-03 | 3.00E-11 |
| rs34177018 | 17 | 34912592 | CTTTTTTTTTTTT | C | -6.38E-03 | 2.12E-03 | 4.00E-03 | -1.25E-02 | 2.16E-03 | 7.00E-09 |
| rs11079849 | 17 | 47090785 | C | T | -2.10E-03 | 2.06E-03 | 3.30E-01 | 1.27E-02 | 2.10E-03 | 1.60E-09 |
| rs77706698 | 17 | 65953348 | G | A | -3.42E-03 | 2.87E-03 | 2.30E-01 | -1.72E-02 | 2.92E-03 | 4.20E-09 |
| rs2619976 | 17 | 71754545 | C | T | -3.26E-03 | 1.98E-03 | 1.40E-01 | -1.17E-02 | 2.01E-03 | 5.40E-09 |
| rs11660335 | 18 | 22154235 | T | C | 3.65E-03 | 2.47E-03 | 1.20E-01 | 1.59E-02 | 2.52E-03 | 2.50E-10 |
| rs784257 | 18 | 53397199 | T | C | 5.24E-05 | 2.49E-03 | 9.20E-01 | -1.41E-02 | 2.53E-03 | 2.90E-08 |
| rs9962947 | 18 | 72903636 | C | T | -7.12E-03 | 2.01E-03 | 5.80E-04 | -1.12E-02 | 2.05E-03 | 4.60E-08 |
| rs149080927 | 19 | 1854253 | G | GC | -8.89E-04 | 1.96E-03 | 5.80E-01 | -1.24E-02 | 1.99E-03 | 5.90E-10 |
| rs350832 | 19 | 4069426 | G | A | 3.72E-04 | 2.32E-03 | 6.20E-01 | -1.37E-02 | 2.36E-03 | 6.50E-09 |
| rs111640872 | 19 | 30290357 | G | C | -5.36E-03 | 2.06E-03 | 6.70E-03 | -1.26E-02 | 2.10E-03 | 1.80E-09 |
| rs429358 | 19 | 45411941 | T | C | -2.48E-03 | 2.68E-03 | 3.60E-01 | 1.75E-02 | 2.73E-03 | 1.40E-10 |
| rs8124896 | 20 | 21385659 | T | C | -5.99E-03 | 3.21E-03 | 9.90E-02 | -2.01E-02 | 3.27E-03 | 6.90E-10 |
| rs116948922 | 20 | 25534854 | C | T | 2.83E-03 | 5.52E-03 | 7.20E-01 | 3.40E-02 | 5.62E-03 | 1.50E-09 |
| rs151157954 | 20 | 51171126 | A | ATG | 4.59E-03 | 2.65E-03 | 7.60E-02 | 1.78E-02 | 2.69E-03 | 3.50E-11 |
| rs915814 | 21 | 46493003 | G | A | 1.04E-03 | 2.27E-03 | 7.00E-01 | 1.31E-02 | 2.31E-03 | 1.50E-08 |
| rs400997 | 21 | 46564154 | T | A | -2.31E-03 | 1.96E-03 | 2.80E-01 | -1.43E-02 | 2.00E-03 | 8.00E-13 |
| rs738140 | 22 | 41884954 | A | G | 1.13E-03 | 2.09E-03 | 6.30E-01 | 1.20E-02 | 2.13E-03 | 1.80E-08 |
|  |  |  |  |  |  |  |  |  |  |  |
| ** Genetic variants strongly associated with childhood adiposity at genome wide significance and not adulthood adiposity (exclude adult SNPs at P ≤ 0.05) were counted. We then divided 0.05 by this number to generate the Bonferroni corrected P value. | | | | | | | | | | |

**Table S2K.** The genetic variants strongly associated with childhood adiposity at genome wide significance and not adulthood adiposity (exclude adult SNPs at P ≤ 0.05).

| SNP | Chromosome | Base position | Effect allele | Other allele | Beta (Adult) | SE (Adult) | P (Adult) | Beta (Age 10) | SE (Age 10) | P (Age 10) |
| --- | --- | --- | --- | --- | --- | --- | --- | --- | --- | --- |
| rs4744246 | 9 | 96254464 | A | G | 9.44E-04 | 1.45E-03 | 5.10E-01 | -1.58E-02 | 1.48E-03 | 1.60E-26 |
| rs34260097 | 6 | 100727703 | T | G | -2.36E-03 | 1.64E-03 | 1.50E-01 | -1.78E-02 | 1.68E-03 | 2.50E-26 |
| rs2767486 | 1 | 65991203 | A | G | -1.36E-03 | 1.70E-03 | 4.20E-01 | -1.54E-02 | 1.74E-03 | 1.20E-18 |
| rs2594994 | 3 | 11339960 | T | A | 2.11E-03 | 1.79E-03 | 2.40E-01 | 1.54E-02 | 1.83E-03 | 3.10E-17 |
| rs788858 | 4 | 82138300 | A | G | 5.76E-04 | 1.51E-03 | 7.00E-01 | 1.23E-02 | 1.54E-03 | 1.70E-15 |
| rs2187642 | 12 | 11855624 | A | C | -1.58E-03 | 1.41E-03 | 2.60E-01 | -1.12E-02 | 1.44E-03 | 1.00E-14 |
| rs117911387 | 9 | 130446836 | G | A | -3.68E-03 | 3.26E-03 | 2.60E-01 | -2.46E-02 | 3.33E-03 | 1.60E-13 |
| rs2229330 | 1 | 6649228 | T | G | -3.47E-03 | 2.64E-03 | 1.90E-01 | -1.97E-02 | 2.69E-03 | 2.30E-13 |
| rs7306710 | 12 | 66376091 | T | C | 7.35E-04 | 1.38E-03 | 5.90E-01 | 9.97E-03 | 1.41E-03 | 1.60E-12 |
| rs836179 | 12 | 50503082 | A | G | 2.37E-03 | 1.42E-03 | 9.50E-02 | 9.81E-03 | 1.45E-03 | 1.40E-11 |
| rs11205303 | 1 | 149906413 | T | C | 1.24E-03 | 1.39E-03 | 3.80E-01 | 9.58E-03 | 1.43E-03 | 1.80E-11 |
| rs601338 | 19 | 49206674 | G | A | 9.17E-04 | 1.37E-03 | 5.00E-01 | 9.38E-03 | 1.40E-03 | 2.10E-11 |
| rs112898427 | 2 | 67561335 | C | T | 1.04E-03 | 1.52E-03 | 4.90E-01 | 1.03E-02 | 1.55E-03 | 2.50E-11 |
| rs7808296 | 7 | 103127620 | C | T | -2.55E-03 | 1.48E-03 | 8.50E-02 | -1.00E-02 | 1.51E-03 | 2.80E-11 |
| rs824207 | 15 | 24007729 | A | G | -2.21E-03 | 1.38E-03 | 1.10E-01 | -9.31E-03 | 1.40E-03 | 3.30E-11 |
| rs7424771 | 2 | 161276378 | G | A | -2.31E-03 | 1.38E-03 | 9.40E-02 | 8.93E-03 | 1.41E-03 | 2.20E-10 |
| rs4572029 | 10 | 70889053 | A | G | -7.22E-04 | 1.71E-03 | 6.70E-01 | 1.11E-02 | 1.75E-03 | 2.20E-10 |
| rs2629881 | 3 | 59778271 | C | T | -2.64E-03 | 1.65E-03 | 1.10E-01 | -1.06E-02 | 1.69E-03 | 3.80E-10 |
| rs62621197 | 19 | 8670147 | C | T | -6.50E-03 | 3.77E-03 | 8.50E-02 | -2.35E-02 | 3.85E-03 | 9.60E-10 |
| rs212517 | 1 | 21577159 | T | A | 1.56E-03 | 1.40E-03 | 2.60E-01 | 8.75E-03 | 1.43E-03 | 9.60E-10 |
| rs62134189 | 2 | 45046339 | A | G | -1.82E-03 | 2.27E-03 | 4.20E-01 | 1.42E-02 | 2.32E-03 | 1.00E-09 |
| rs117455294 | 20 | 57427951 | C | A | 4.90E-03 | 3.11E-03 | 1.10E-01 | 1.93E-02 | 3.17E-03 | 1.10E-09 |
| rs10234366 | 7 | 46743746 | G | A | -1.75E-03 | 2.24E-03 | 4.40E-01 | -1.40E-02 | 2.29E-03 | 1.10E-09 |
| rs3936511 | 5 | 55860781 | A | G | 2.42E-04 | 1.74E-03 | 8.90E-01 | 1.08E-02 | 1.78E-03 | 1.10E-09 |
| rs10860295 | 12 | 98542699 | T | C | -1.81E-03 | 1.38E-03 | 1.90E-01 | -8.52E-03 | 1.41E-03 | 1.60E-09 |
| rs10498713 | 6 | 22729300 | G | T | -1.36E-03 | 1.92E-03 | 4.80E-01 | -1.18E-02 | 1.97E-03 | 2.00E-09 |
| rs10953577 | 7 | 108263540 | T | C | -2.48E-03 | 1.42E-03 | 8.00E-02 | -8.58E-03 | 1.45E-03 | 3.00E-09 |
| rs4723263 | 7 | 33194826 | G | C | -2.54E-03 | 1.38E-03 | 6.60E-02 | -8.33E-03 | 1.41E-03 | 3.70E-09 |
| rs7123283 | 11 | 122809055 | C | T | 2.53E-03 | 1.38E-03 | 6.70E-02 | 8.32E-03 | 1.41E-03 | 3.70E-09 |
| rs67603370 | 17 | 7524504 | G | A | -4.59E-04 | 2.63E-03 | 8.60E-01 | -1.58E-02 | 2.68E-03 | 3.70E-09 |
| rs11891707 | 2 | 207120604 | T | C | 4.36E-04 | 2.01E-03 | 8.30E-01 | 1.21E-02 | 2.05E-03 | 3.80E-09 |
| rs62032001 | 16 | 49065630 | A | C | -1.94E-03 | 1.73E-03 | 2.60E-01 | -1.04E-02 | 1.77E-03 | 4.20E-09 |
| rs3118252 | 9 | 25115154 | G | C | -1.47E-03 | 1.40E-03 | 2.90E-01 | -8.32E-03 | 1.43E-03 | 6.00E-09 |
| rs115359679 | 7 | 755987 | C | A | -5.38E-03 | 2.80E-03 | 5.40E-02 | -1.64E-02 | 2.86E-03 | 9.20E-09 |
| rs7565437 | 2 | 65646966 | T | C | 2.01E-03 | 1.39E-03 | 1.50E-01 | 8.18E-03 | 1.43E-03 | 9.50E-09 |
| rs11256627 | 10 | 10535954 | G | A | -9.45E-04 | 1.51E-03 | 5.30E-01 | -8.88E-03 | 1.55E-03 | 9.50E-09 |
| rs4545941 | 19 | 16534207 | T | C | -2.12E-03 | 1.86E-03 | 2.60E-01 | -1.09E-02 | 1.90E-03 | 1.10E-08 |
| rs4783789 | 16 | 51446707 | T | C | 1.65E-03 | 1.65E-03 | 3.20E-01 | 9.58E-03 | 1.68E-03 | 1.20E-08 |
| rs12308065 | 12 | 120624085 | A | G | -6.54E-04 | 1.42E-03 | 6.50E-01 | -8.29E-03 | 1.45E-03 | 1.20E-08 |
| rs201666051 | 9 | 20920868 | C | T | -1.49E-03 | 1.42E-03 | 2.90E-01 | -8.24E-03 | 1.45E-03 | 1.30E-08 |
| rs1476698 | 2 | 242296449 | A | G | 1.39E-03 | 1.42E-03 | 3.30E-01 | 8.22E-03 | 1.45E-03 | 1.40E-08 |
| rs2755253 | 1 | 67470843 | C | T | 1.53E-03 | 1.51E-03 | 3.10E-01 | 8.71E-03 | 1.54E-03 | 1.50E-08 |
| rs1177279 | 2 | 61295122 | A | G | 2.06E-03 | 1.53E-03 | 1.80E-01 | 8.83E-03 | 1.56E-03 | 1.60E-08 |
| rs6577497 | 1 | 8605667 | A | T | -2.20E-03 | 1.40E-03 | 1.20E-01 | 8.07E-03 | 1.43E-03 | 1.80E-08 |
| rs2958542 | 11 | 62181882 | C | T | 3.51E-04 | 1.43E-03 | 8.10E-01 | 8.24E-03 | 1.46E-03 | 1.80E-08 |
| rs115903965 | 3 | 66009529 | G | A | 1.86E-03 | 4.44E-03 | 6.80E-01 | -2.55E-02 | 4.54E-03 | 2.00E-08 |
| rs10823504 | 10 | 72034062 | G | A | 9.02E-04 | 2.81E-03 | 7.50E-01 | 1.59E-02 | 2.87E-03 | 3.40E-08 |
| rs16839832 | 1 | 196349909 | G | T | -2.65E-03 | 2.50E-03 | 2.90E-01 | -1.41E-02 | 2.55E-03 | 3.50E-08 |
| rs76187039 | 6 | 43233990 | G | T | -1.12E-03 | 2.01E-03 | 5.80E-01 | -1.13E-02 | 2.05E-03 | 3.50E-08 |
| rs2175171 | 1 | 7028842 | G | C | -1.93E-03 | 1.38E-03 | 1.60E-01 | -7.77E-03 | 1.41E-03 | 3.60E-08 |
| rs1000471 | 15 | 89986583 | C | T | 9.10E-04 | 1.69E-03 | 5.90E-01 | -9.51E-03 | 1.73E-03 | 3.60E-08 |
| rs3172332 | 3 | 153973408 | T | C | 2.69E-03 | 1.42E-03 | 5.80E-02 | 8.01E-03 | 1.46E-03 | 3.70E-08 |
| rs11655704 | 17 | 47448172 | T | C | 2.44E-03 | 1.47E-03 | 9.70E-02 | 8.27E-03 | 1.50E-03 | 3.80E-08 |
| rs10503555 | 8 | 15763818 | A | G | -6.46E-04 | 1.39E-03 | 6.40E-01 | 7.78E-03 | 1.41E-03 | 3.90E-08 |
| rs67679818 | 7 | 110672704 | C | T | 4.06E-04 | 1.40E-03 | 7.70E-01 | 7.80E-03 | 1.43E-03 | 4.90E-08 |

**Table S2L.** The genetic variants strongly associated with childhood adiposity at genome wide significance and not adulthood adiposity (exclude adult SNPs at P ≤ 0.05) in females.

| SNP | Chromosome | Base position | Effect allele | Other allele | Beta (Age 10) | SE (Age 10) | P (Age 10) | Beta (Adult) | SE (Adult) | P (Adult) |
| --- | --- | --- | --- | --- | --- | --- | --- | --- | --- | --- |
| rs212540 | 1 | 21593117 | C | T | 1.27E-02 | 2.00E-03 | 9.90E-10 | 1.56E-03 | 2.03E-03 | 4.40E-01 |
| rs2767486 | 1 | 65991203 | A | G | -2.03E-02 | 2.41E-03 | 9.90E-17 | -1.24E-03 | 2.45E-03 | 6.10E-01 |
| rs36134621 | 2 | 161046192 | G | A | 1.11E-02 | 1.95E-03 | 3.50E-08 | -2.61E-03 | 1.98E-03 | 1.90E-01 |
| rs17464221 | 2 | 188278203 | C | T | 1.23E-02 | 2.14E-03 | 1.50E-08 | 2.65E-03 | 2.17E-03 | 2.20E-01 |
| rs115319174 | 2 | 207066474 | G | C | -4.11E-02 | 4.20E-03 | 7.60E-23 | -7.38E-03 | 4.27E-03 | 8.40E-02 |
| rs2594989 | 3 | 11316143 | C | T | 1.87E-02 | 2.53E-03 | 4.00E-14 | -4.61E-04 | 2.58E-03 | 8.60E-01 |
| rs2034963 | 3 | 48170802 | G | C | 1.34E-02 | 2.04E-03 | 5.40E-11 | 3.43E-03 | 2.08E-03 | 9.90E-02 |
| rs2629881 | 3 | 59778271 | C | T | -1.32E-02 | 2.34E-03 | 1.70E-08 | -4.55E-03 | 2.37E-03 | 5.50E-02 |
| rs1349641 | 4 | 82212652 | T | G | 1.25E-02 | 1.98E-03 | 9.70E-10 | 2.39E-03 | 2.02E-03 | 2.40E-01 |
| rs3936511 | 5 | 55860781 | A | G | 1.54E-02 | 2.46E-03 | 7.10E-11 | 8.43E-04 | 2.50E-03 | 7.40E-01 |
| rs13190020 | 5 | 65012526 | G | A | -1.15E-02 | 2.03E-03 | 1.40E-08 | -1.57E-03 | 2.07E-03 | 4.50E-01 |
| rs34260097 | 6 | 100727703 | T | G | -2.52E-02 | 2.32E-03 | 2.40E-28 | -3.62E-03 | 2.36E-03 | 1.20E-01 |
| rs7759938 | 6 | 105378954 | C | T | -1.16E-02 | 2.07E-03 | 1.70E-08 | -1.87E-03 | 2.11E-03 | 3.80E-01 |
| rs62425122 | 6 | 166311987 | G | A | 1.24E-02 | 2.12E-03 | 4.10E-09 | 3.30E-03 | 2.15E-03 | 1.30E-01 |
| rs7808296 | 7 | 103127620 | C | T | -1.17E-02 | 2.08E-03 | 4.80E-08 | -3.17E-03 | 2.12E-03 | 1.30E-01 |
| rs6979832 | 7 | 127856276 | A | G | -1.13E-02 | 1.95E-03 | 1.60E-08 | -3.09E-03 | 1.98E-03 | 1.20E-01 |
| rs62515439 | 8 | 57165417 | C | T | 1.27E-02 | 2.01E-03 | 1.20E-10 | 3.68E-03 | 2.04E-03 | 7.20E-02 |
| rs10821163 | 9 | 96343060 | G | C | -1.84E-02 | 2.05E-03 | 3.50E-19 | 2.15E-03 | 2.09E-03 | 3.00E-01 |
| rs2187642 | 12 | 11855624 | A | C | -1.19E-02 | 1.99E-03 | 3.80E-09 | -5.08E-04 | 2.03E-03 | 8.00E-01 |
| rs10784514 | 12 | 66452879 | C | T | -1.16E-02 | 2.07E-03 | 4.20E-08 | -5.94E-04 | 2.11E-03 | 7.80E-01 |
| rs2364232 | 12 | 93994827 | A | C | 1.23E-02 | 2.21E-03 | 2.10E-08 | 1.02E-03 | 2.25E-03 | 6.50E-01 |
| rs7305424 | 12 | 118399491 | A | T | -1.21E-02 | 2.05E-03 | 5.30E-09 | -3.79E-03 | 2.08E-03 | 6.90E-02 |
| rs11863799 | 16 | 61933401 | C | T | 1.14E-02 | 2.07E-03 | 1.80E-08 | 1.35E-03 | 2.10E-03 | 5.20E-01 |
| rs12185242 | 17 | 47407071 | A | C | -1.10E-02 | 1.94E-03 | 7.10E-09 | -2.62E-03 | 1.98E-03 | 1.90E-01 |
| rs3810304 | 19 | 30861683 | A | G | 1.43E-02 | 2.32E-03 | 1.30E-09 | 1.90E-04 | 2.36E-03 | 9.40E-01 |
| rs633372 | 19 | 49209226 | G | A | 1.11E-02 | 1.94E-03 | 5.70E-09 | 1.84E-03 | 1.97E-03 | 3.50E-01 |
| rs763842194 | 20 | 54377089 | AG | A | 1.50E-02 | 2.20E-03 | 1.30E-11 | 2.91E-03 | 2.23E-03 | 1.90E-01 |
| rs374873051 | 22 | 22266944 | CTTTTTTTTTTTTT | C | -1.13E-02 | 2.02E-03 | 1.70E-09 | -2.21E-03 | 2.06E-03 | 2.80E-01 |

**Table 2SM.** The genetic variants strongly associated with adulthood adiposity at genome wide significance and not childhood adiposity (exclude child SNPs at P ≤ 0.05).

| SNP | Chromosome | Base position | Effect allele | Other allele | Beta (Adult) | SE (Adult) | P (Adult) | Beta (Age 10) | SE (Age 10) | P (Age 10) |
| --- | --- | --- | --- | --- | --- | --- | --- | --- | --- | --- |
| rs61754230 | 12 | 72179446 | C | T | -2.74E-02 | 4.93E-03 | 2.80E-08 | -9.81E-03 | 5.03E-03 | 5.10E-02 |
| rs7086898 | 10 | 104386152 | A | G | -1.39E-02 | 2.53E-03 | 4.30E-08 | -4.98E-03 | 2.59E-03 | 5.40E-02 |
| rs1631026 | 2 | 26953850 | C | T | -1.07E-02 | 1.37E-03 | 5.90E-15 | -2.69E-03 | 1.40E-03 | 5.50E-02 |
| rs1964926 | 21 | 42653121 | A | G | -8.23E-03 | 1.44E-03 | 1.10E-08 | -2.82E-03 | 1.47E-03 | 5.50E-02 |
| rs112852122 | 20 | 47498117 | G | A | 1.34E-02 | 1.91E-03 | 2.70E-12 | 3.75E-03 | 1.95E-03 | 5.50E-02 |
| rs6761463 | 2 | 50201547 | G | C | 1.26E-02 | 1.86E-03 | 1.30E-11 | 3.61E-03 | 1.90E-03 | 5.70E-02 |
| rs11047138 | 12 | 24019853 | C | G | -1.44E-02 | 2.63E-03 | 4.60E-08 | -5.08E-03 | 2.68E-03 | 5.80E-02 |
| rs16940823 | 18 | 22137319 | C | A | 1.08E-02 | 1.79E-03 | 1.60E-09 | 3.42E-03 | 1.82E-03 | 6.10E-02 |
| rs66674732 | 13 | 62721160 | G | A | -8.16E-03 | 1.41E-03 | 7.20E-09 | -2.68E-03 | 1.44E-03 | 6.30E-02 |
| rs1381010 | 4 | 112677085 | G | A | 8.24E-03 | 1.49E-03 | 3.00E-08 | 2.81E-03 | 1.52E-03 | 6.40E-02 |
| rs369461388 | 14 | 40104718 | G | C | -1.26E-02 | 2.01E-03 | 3.60E-10 | -3.80E-03 | 2.05E-03 | 6.40E-02 |
| rs1369159 | 15 | 66360842 | C | T | 7.88E-03 | 1.40E-03 | 1.70E-08 | 2.63E-03 | 1.43E-03 | 6.50E-02 |
| rs4989244 | 9 | 102100348 | G | A | 7.63E-03 | 1.39E-03 | 3.60E-08 | 2.61E-03 | 1.42E-03 | 6.60E-02 |
| rs12149660 | 16 | 70309237 | G | A | 1.62E-02 | 2.16E-03 | 6.20E-14 | 4.01E-03 | 2.21E-03 | 6.90E-02 |
| rs2035806 | 10 | 133984916 | G | A | 9.55E-03 | 1.39E-03 | 5.70E-12 | 2.56E-03 | 1.42E-03 | 7.00E-02 |
| rs429358 | 19 | 45411941 | T | C | 1.61E-02 | 1.90E-03 | 2.90E-17 | -3.45E-03 | 1.94E-03 | 7.50E-02 |
| rs7230240 | 18 | 42597978 | C | T | 9.40E-03 | 1.50E-03 | 4.20E-10 | 2.73E-03 | 1.54E-03 | 7.60E-02 |
| rs34542489 | 17 | 51917844 | A | C | 7.78E-03 | 1.40E-03 | 2.80E-08 | 2.52E-03 | 1.43E-03 | 7.80E-02 |
| rs201475383 | 20 | 26273991 | G | A | 2.40E-02 | 3.92E-03 | 8.80E-10 | 6.98E-03 | 4.00E-03 | 8.10E-02 |
| rs2056477 | 7 | 2079744 | G | C | 1.16E-02 | 1.64E-03 | 1.50E-12 | 2.90E-03 | 1.67E-03 | 8.30E-02 |
| rs6075658 | 20 | 2094078 | T | C | 8.49E-03 | 1.38E-03 | 6.80E-10 | 2.43E-03 | 1.40E-03 | 8.40E-02 |
| rs368540015 | 7 | 74292165 | A | G | -1.82E-02 | 3.25E-03 | 2.20E-08 | -5.73E-03 | 3.32E-03 | 8.40E-02 |
| rs142503704 | 5 | 92622421 | G | A | -2.63E-02 | 4.68E-03 | 1.90E-08 | -8.13E-03 | 4.78E-03 | 8.90E-02 |
| rs6050446 | 20 | 25195509 | A | G | -2.64E-02 | 3.89E-03 | 1.20E-11 | -6.70E-03 | 3.98E-03 | 9.20E-02 |
| rs1987960 | 20 | 30649834 | T | C | -1.89E-02 | 3.31E-03 | 1.10E-08 | -5.63E-03 | 3.38E-03 | 9.60E-02 |
| rs2660241 | 16 | 4940023 | T | C | -8.13E-03 | 1.43E-03 | 1.20E-08 | -2.41E-03 | 1.46E-03 | 9.90E-02 |
| rs3737992 | 1 | 33234128 | G | A | 1.37E-02 | 1.83E-03 | 5.30E-14 | 3.07E-03 | 1.87E-03 | 9.90E-02 |
| rs6823268 | 4 | 145982563 | A | G | -8.18E-03 | 1.42E-03 | 8.20E-09 | -2.36E-03 | 1.45E-03 | 1.00E-01 |
| rs75957461 | 19 | 11166163 | C | T | -1.77E-02 | 3.05E-03 | 6.60E-09 | -5.11E-03 | 3.11E-03 | 1.00E-01 |
| rs7182917 | 15 | 52080803 | T | C | 8.74E-03 | 1.39E-03 | 2.80E-10 | 2.25E-03 | 1.41E-03 | 1.10E-01 |
| rs9615723 | 22 | 48386670 | C | T | 7.71E-03 | 1.40E-03 | 3.80E-08 | 2.26E-03 | 1.43E-03 | 1.10E-01 |
| rs10960276 | 9 | 11819686 | C | A | 8.09E-03 | 1.43E-03 | 1.60E-08 | -2.34E-03 | 1.46E-03 | 1.10E-01 |
| rs4911382 | 20 | 32553095 | C | T | -8.32E-03 | 1.40E-03 | 2.60E-09 | -2.23E-03 | 1.43E-03 | 1.20E-01 |
| rs868784 | 11 | 43944388 | G | A | 7.87E-03 | 1.42E-03 | 2.70E-08 | 2.28E-03 | 1.45E-03 | 1.20E-01 |
| rs10823826 | 10 | 53649431 | C | T | -8.98E-03 | 1.59E-03 | 1.60E-08 | -2.56E-03 | 1.62E-03 | 1.20E-01 |
| rs12885251 | 14 | 99670791 | G | A | 7.63E-03 | 1.39E-03 | 4.10E-08 | 2.13E-03 | 1.42E-03 | 1.30E-01 |
| rs10505836 | 12 | 19288508 | A | C | -1.21E-02 | 1.99E-03 | 1.10E-09 | -3.08E-03 | 2.03E-03 | 1.30E-01 |
| rs77560793 | 1 | 175001179 | G | A | 2.46E-02 | 3.99E-03 | 7.80E-10 | 6.25E-03 | 4.08E-03 | 1.30E-01 |
| rs7570446 | 2 | 193801010 | C | A | -7.80E-03 | 1.37E-03 | 1.30E-08 | 2.06E-03 | 1.40E-03 | 1.40E-01 |
| rs12477088 | 2 | 67841326 | T | C | 1.05E-02 | 1.39E-03 | 4.70E-14 | 2.08E-03 | 1.42E-03 | 1.40E-01 |
| rs2542615 | 10 | 131128952 | C | T | 8.44E-03 | 1.46E-03 | 7.30E-09 | 2.21E-03 | 1.49E-03 | 1.40E-01 |
| rs1799507 | 12 | 16427314 | G | A | -1.08E-02 | 1.96E-03 | 3.10E-08 | -2.95E-03 | 2.00E-03 | 1.40E-01 |
| rs945211 | 1 | 32191798 | G | C | -8.49E-03 | 1.41E-03 | 1.60E-09 | -2.06E-03 | 1.44E-03 | 1.50E-01 |
| rs2516726 | 16 | 2095065 | T | C | 9.91E-03 | 1.64E-03 | 1.70E-09 | 2.42E-03 | 1.68E-03 | 1.50E-01 |
| rs72753485 | 9 | 96673230 | G | C | -1.56E-02 | 2.50E-03 | 4.30E-10 | -3.68E-03 | 2.55E-03 | 1.50E-01 |
| rs1373349 | 18 | 63282992 | C | T | 9.81E-03 | 1.48E-03 | 3.50E-11 | 2.13E-03 | 1.51E-03 | 1.60E-01 |
| rs79675564 | 2 | 211286896 | C | A | -1.51E-02 | 2.55E-03 | 3.40E-09 | -3.67E-03 | 2.61E-03 | 1.60E-01 |
| rs12927792 | 16 | 9713194 | C | T | -8.67E-03 | 1.40E-03 | 5.20E-10 | -1.97E-03 | 1.43E-03 | 1.70E-01 |
| rs7264802 | 20 | 62692440 | A | G | -9.81E-03 | 1.59E-03 | 7.20E-10 | -2.22E-03 | 1.62E-03 | 1.70E-01 |
| rs12477385 | 2 | 166144850 | G | T | 9.24E-03 | 1.64E-03 | 1.80E-08 | 2.28E-03 | 1.68E-03 | 1.70E-01 |
| rs10774018 | 12 | 2157925 | G | C | -9.48E-03 | 1.66E-03 | 1.10E-08 | -2.32E-03 | 1.69E-03 | 1.70E-01 |
| rs12705894 | 7 | 113351252 | G | A | 7.80E-03 | 1.38E-03 | 1.60E-08 | 1.90E-03 | 1.41E-03 | 1.80E-01 |
| rs187067151 | 20 | 29539588 | G | T | 2.67E-02 | 3.98E-03 | 2.00E-11 | 5.44E-03 | 4.06E-03 | 1.80E-01 |
| rs403694 | 21 | 46567625 | C | T | -1.22E-02 | 1.38E-03 | 1.30E-18 | -1.86E-03 | 1.41E-03 | 1.90E-01 |
| rs7893571 | 10 | 16750129 | G | T | -1.02E-02 | 1.46E-03 | 2.90E-12 | -1.93E-03 | 1.49E-03 | 1.90E-01 |
| rs36007635 | 6 | 163009335 | G | A | 1.37E-02 | 1.99E-03 | 5.60E-12 | 2.65E-03 | 2.03E-03 | 1.90E-01 |
| rs7548936 | 1 | 91207757 | G | C | -8.48E-03 | 1.42E-03 | 2.10E-09 | 1.85E-03 | 1.45E-03 | 2.00E-01 |
| rs151252883 | 2 | 228998026 | T | G | -9.22E-03 | 1.44E-03 | 1.60E-10 | -1.91E-03 | 1.47E-03 | 2.00E-01 |
| rs183315407 | 1 | 46201427 | G | A | 2.64E-02 | 3.49E-03 | 4.30E-14 | 4.54E-03 | 3.57E-03 | 2.00E-01 |
| rs2074686 | 7 | 100800635 | G | A | 8.28E-03 | 1.39E-03 | 2.90E-09 | 1.79E-03 | 1.42E-03 | 2.10E-01 |
| rs34373881 | 3 | 20432033 | G | A | 8.83E-03 | 1.54E-03 | 9.00E-09 | 1.98E-03 | 1.57E-03 | 2.10E-01 |
| rs12644329 | 4 | 143634746 | G | A | 7.91E-03 | 1.42E-03 | 2.50E-08 | 1.78E-03 | 1.45E-03 | 2.20E-01 |
| rs7518221 | 1 | 225561346 | T | C | 7.95E-03 | 1.43E-03 | 2.80E-08 | 1.80E-03 | 1.46E-03 | 2.20E-01 |
| rs4759228 | 12 | 56508409 | G | C | 1.16E-02 | 1.51E-03 | 1.30E-14 | 1.89E-03 | 1.54E-03 | 2.20E-01 |
| rs575840515 | 1 | 80796649 | A | G | -9.01E-03 | 1.52E-03 | 3.20E-09 | -1.90E-03 | 1.56E-03 | 2.20E-01 |
| rs10973159 | 9 | 36992547 | G | T | -8.05E-03 | 1.41E-03 | 1.20E-08 | 1.74E-03 | 1.44E-03 | 2.30E-01 |
| rs10760277 | 9 | 126093999 | C | T | -8.87E-03 | 1.41E-03 | 3.60E-10 | -1.68E-03 | 1.44E-03 | 2.40E-01 |
| rs61903695 | 11 | 89922417 | A | G | -1.13E-02 | 1.58E-03 | 6.60E-13 | -1.87E-03 | 1.61E-03 | 2.40E-01 |
| rs78517245 | 3 | 42587865 | T | C | -3.44E-02 | 5.85E-03 | 4.10E-09 | -6.98E-03 | 5.98E-03 | 2.40E-01 |
| rs10805383 | 5 | 63034606 | G | A | -1.05E-02 | 1.37E-03 | 2.60E-14 | -1.57E-03 | 1.40E-03 | 2.60E-01 |
| rs9522180 | 13 | 111970212 | C | T | 9.24E-03 | 1.38E-03 | 2.40E-11 | 1.55E-03 | 1.41E-03 | 2.70E-01 |
| rs1324110 | 6 | 93913200 | G | C | 7.77E-03 | 1.38E-03 | 2.00E-08 | 1.57E-03 | 1.41E-03 | 2.70E-01 |
| rs72910629 | 6 | 69761994 | A | G | -1.33E-02 | 2.01E-03 | 4.30E-11 | 2.26E-03 | 2.05E-03 | 2.70E-01 |
| rs78369934 | 17 | 61739101 | T | C | 1.99E-02 | 3.05E-03 | 6.00E-11 | -3.44E-03 | 3.11E-03 | 2.70E-01 |
| rs78886584 | 1 | 16859325 | A | G | -8.49E-03 | 1.38E-03 | 8.70E-10 | -1.52E-03 | 1.42E-03 | 2.80E-01 |
| rs409696 | 2 | 147900651 | G | A | 1.14E-02 | 1.39E-03 | 1.80E-16 | 1.55E-03 | 1.42E-03 | 2.80E-01 |
| rs12357890 | 10 | 99762693 | A | G | -1.22E-02 | 1.39E-03 | 1.60E-18 | -1.53E-03 | 1.42E-03 | 2.80E-01 |
| rs4806814 | 19 | 1860147 | G | A | 1.27E-02 | 1.90E-03 | 3.10E-11 | -2.10E-03 | 1.94E-03 | 2.80E-01 |
| rs78508049 | 1 | 210344884 | T | C | -1.11E-02 | 1.75E-03 | 2.10E-10 | -1.88E-03 | 1.79E-03 | 2.90E-01 |
| rs55931203 | 17 | 65854602 | C | T | -1.26E-02 | 1.78E-03 | 1.60E-12 | -1.92E-03 | 1.82E-03 | 2.90E-01 |
| rs149457 | 5 | 107438057 | C | T | 1.50E-02 | 1.83E-03 | 2.10E-16 | 1.98E-03 | 1.87E-03 | 2.90E-01 |
| rs11708540 | 3 | 70593081 | G | A | -1.06E-02 | 1.89E-03 | 2.00E-08 | 2.07E-03 | 1.93E-03 | 2.90E-01 |
| rs567230078 | 6 | 43588227 | T | A | 2.51E-02 | 4.25E-03 | 3.70E-09 | 4.57E-03 | 4.34E-03 | 2.90E-01 |
| rs13292699 | 9 | 15910044 | A | C | 1.25E-02 | 1.39E-03 | 1.50E-19 | -1.47E-03 | 1.42E-03 | 3.00E-01 |
| rs7913496 | 10 | 10257277 | C | T | 1.00E-02 | 1.79E-03 | 2.00E-08 | -1.88E-03 | 1.83E-03 | 3.00E-01 |
| rs76520838 | 15 | 47916618 | C | T | -2.32E-02 | 3.87E-03 | 1.90E-09 | -4.12E-03 | 3.95E-03 | 3.00E-01 |
| rs2396625 | 7 | 113028634 | T | A | 9.94E-03 | 1.40E-03 | 1.10E-12 | 1.45E-03 | 1.43E-03 | 3.10E-01 |
| rs3806114 | 6 | 20482335 | G | A | 8.79E-03 | 1.47E-03 | 2.30E-09 | 1.51E-03 | 1.50E-03 | 3.10E-01 |
| rs116195355 | 1 | 39941508 | C | A | 2.60E-02 | 3.96E-03 | 5.90E-11 | 4.09E-03 | 4.05E-03 | 3.10E-01 |
| rs698147 | 5 | 3513485 | A | G | 8.78E-03 | 1.38E-03 | 1.80E-10 | 1.41E-03 | 1.41E-03 | 3.20E-01 |
| rs1840660 | 7 | 114352615 | G | A | -9.95E-03 | 1.41E-03 | 2.00E-12 | 1.44E-03 | 1.45E-03 | 3.20E-01 |
| rs2866720 | 7 | 70106310 | C | T | -9.13E-03 | 1.42E-03 | 1.20E-10 | -1.46E-03 | 1.45E-03 | 3.20E-01 |
| rs2269610 | 6 | 33289935 | G | C | -1.28E-02 | 1.77E-03 | 5.10E-13 | 1.78E-03 | 1.81E-03 | 3.20E-01 |
| rs4962725 | 10 | 126733321 | T | C | -9.97E-03 | 1.39E-03 | 6.60E-13 | -1.39E-03 | 1.42E-03 | 3.30E-01 |
| rs2837398 | 21 | 41427168 | A | C | -8.20E-03 | 1.40E-03 | 4.80E-09 | 1.39E-03 | 1.43E-03 | 3.30E-01 |
| rs262956 | 3 | 183486117 | T | G | 9.12E-03 | 1.44E-03 | 2.20E-10 | 1.43E-03 | 1.47E-03 | 3.30E-01 |
| rs6530737 | 8 | 14095763 | A | G | 9.02E-03 | 1.44E-03 | 3.50E-10 | 1.40E-03 | 1.47E-03 | 3.40E-01 |
| rs11079849 | 17 | 47090785 | C | T | 1.22E-02 | 1.46E-03 | 6.40E-17 | 1.42E-03 | 1.49E-03 | 3.40E-01 |
| rs35957544 | 8 | 73440371 | G | T | 1.27E-02 | 1.39E-03 | 7.40E-20 | 1.33E-03 | 1.42E-03 | 3.50E-01 |
| rs28726372 | 1 | 84353839 | T | C | -9.08E-03 | 1.48E-03 | 9.40E-10 | -1.43E-03 | 1.52E-03 | 3.50E-01 |
| rs9421249 | 10 | 118623322 | C | T | -1.04E-02 | 1.56E-03 | 2.50E-11 | 1.49E-03 | 1.60E-03 | 3.50E-01 |
| rs61217499 | 12 | 108417780 | G | C | 1.28E-02 | 1.67E-03 | 2.30E-14 | 1.60E-03 | 1.71E-03 | 3.50E-01 |
| rs57654548 | 3 | 125196904 | C | A | 1.16E-02 | 2.12E-03 | 4.20E-08 | 2.02E-03 | 2.17E-03 | 3.50E-01 |
| rs11642387 | 16 | 6753239 | A | G | 1.29E-02 | 2.29E-03 | 2.10E-08 | -2.20E-03 | 2.34E-03 | 3.50E-01 |
| rs4836133 | 5 | 124332103 | C | A | -8.36E-03 | 1.41E-03 | 3.10E-09 | 2.31E-03 | 2.47E-03 | 3.50E-01 |
| rs2164300 | 4 | 67813017 | C | T | 7.55E-03 | 1.38E-03 | 4.20E-08 | -1.26E-03 | 1.41E-03 | 3.70E-01 |
| rs1454687 | 3 | 94038085 | C | G | 1.23E-02 | 1.37E-03 | 3.00E-19 | 1.24E-03 | 1.40E-03 | 3.80E-01 |
| rs114728753 | 3 | 78483402 | A | C | -8.95E-03 | 1.47E-03 | 1.00E-09 | -1.32E-03 | 1.50E-03 | 3.80E-01 |
| rs396354 | 2 | 86850022 | T | C | 1.04E-02 | 1.52E-03 | 8.00E-12 | 1.36E-03 | 1.55E-03 | 3.80E-01 |
| rs236660 | 7 | 75050086 | T | C | -1.45E-02 | 1.44E-03 | 9.10E-24 | -1.27E-03 | 1.47E-03 | 3.90E-01 |
| rs12427047 | 12 | 90213070 | C | T | 1.09E-02 | 1.60E-03 | 8.90E-12 | 1.40E-03 | 1.63E-03 | 3.90E-01 |
| rs1547205 | 9 | 98815145 | G | C | 1.32E-02 | 2.32E-03 | 1.30E-08 | 2.02E-03 | 2.37E-03 | 3.90E-01 |
| rs1458156 | 12 | 41887940 | C | T | -9.29E-03 | 1.37E-03 | 1.30E-11 | -1.19E-03 | 1.40E-03 | 4.00E-01 |
| rs1704190 | 2 | 200760629 | G | A | -8.33E-03 | 1.42E-03 | 4.50E-09 | -1.23E-03 | 1.45E-03 | 4.00E-01 |
| rs3753639 | 1 | 154986091 | T | C | -1.15E-02 | 1.60E-03 | 7.30E-13 | -1.38E-03 | 1.64E-03 | 4.00E-01 |
| rs7539903 | 1 | 209208033 | T | A | 7.69E-03 | 1.41E-03 | 4.60E-08 | 1.18E-03 | 1.44E-03 | 4.10E-01 |
| rs7321285 | 13 | 54319327 | A | C | 1.13E-02 | 1.72E-03 | 4.10E-11 | 1.46E-03 | 1.76E-03 | 4.10E-01 |
| rs1411432 | 9 | 16728532 | A | C | -1.24E-02 | 1.77E-03 | 2.00E-12 | 1.49E-03 | 1.81E-03 | 4.10E-01 |
| rs10756555 | 9 | 14459089 | G | A | 1.03E-02 | 1.39E-03 | 1.60E-13 | 1.13E-03 | 1.42E-03 | 4.20E-01 |
| rs4419475 | 4 | 96150044 | A | T | -8.10E-03 | 1.39E-03 | 6.50E-09 | 1.16E-03 | 1.43E-03 | 4.20E-01 |
| rs9888533 | 13 | 107854612 | C | T | -7.65E-03 | 1.40E-03 | 4.50E-08 | -1.15E-03 | 1.43E-03 | 4.20E-01 |
| rs7175642 | 15 | 59450079 | T | G | 8.59E-03 | 1.47E-03 | 5.20E-09 | 1.22E-03 | 1.50E-03 | 4.20E-01 |
| rs111584879 | 15 | 66678173 | T | C | 8.79E-03 | 1.61E-03 | 4.80E-08 | 1.33E-03 | 1.64E-03 | 4.20E-01 |
| rs12630209 | 3 | 156881392 | T | G | -8.75E-03 | 1.58E-03 | 3.00E-08 | -1.27E-03 | 1.61E-03 | 4.30E-01 |
| rs12376870 | 9 | 117890567 | G | A | 8.87E-03 | 1.62E-03 | 4.10E-08 | 1.28E-03 | 1.65E-03 | 4.40E-01 |
| rs11513729 | 12 | 112273499 | C | T | 7.99E-03 | 1.41E-03 | 1.50E-08 | 1.07E-03 | 1.44E-03 | 4.60E-01 |
| rs11134512 | 5 | 167847460 | T | G | 8.24E-03 | 1.47E-03 | 1.90E-08 | 1.12E-03 | 1.50E-03 | 4.60E-01 |
| rs7928320 | 11 | 116942753 | C | G | -1.73E-02 | 2.97E-03 | 6.10E-09 | -2.23E-03 | 3.04E-03 | 4.60E-01 |
| rs473837 | 8 | 60906881 | G | T | 8.63E-03 | 1.44E-03 | 1.80E-09 | -1.01E-03 | 1.47E-03 | 4.90E-01 |
| rs11765062 | 7 | 54417515 | T | C | 7.52E-03 | 1.37E-03 | 4.60E-08 | 9.37E-04 | 1.40E-03 | 5.00E-01 |
| rs12281009 | 11 | 117032959 | A | G | -1.79E-02 | 2.95E-03 | 1.30E-09 | -2.02E-03 | 3.01E-03 | 5.00E-01 |
| rs6950388 | 7 | 1270699 | G | A | -9.45E-03 | 1.70E-03 | 2.60E-08 | 1.15E-03 | 1.74E-03 | 5.10E-01 |
| rs12538826 | 7 | 99030228 | T | C | 1.68E-02 | 2.15E-03 | 7.20E-15 | -1.43E-03 | 2.20E-03 | 5.10E-01 |
| rs7433076 | 3 | 90234502 | T | A | 9.52E-03 | 1.38E-03 | 5.10E-12 | -9.17E-04 | 1.41E-03 | 5.20E-01 |
| rs1977658 | 1 | 107607037 | T | G | 8.10E-03 | 1.45E-03 | 2.50E-08 | -9.64E-04 | 1.49E-03 | 5.20E-01 |
| rs217672 | 14 | 62361021 | A | C | -1.18E-02 | 1.55E-03 | 2.30E-14 | 1.02E-03 | 1.58E-03 | 5.20E-01 |
| rs1040046 | 6 | 83473573 | C | A | -1.13E-02 | 1.92E-03 | 4.60E-09 | -1.25E-03 | 1.96E-03 | 5.20E-01 |
| rs35390852 | 4 | 143067054 | G | A | -1.14E-02 | 2.09E-03 | 4.80E-08 | -1.33E-03 | 2.13E-03 | 5.30E-01 |
| rs4663213 | 2 | 236807893 | G | A | 9.46E-03 | 1.66E-03 | 1.20E-08 | 1.04E-03 | 1.70E-03 | 5.40E-01 |
| rs73213484 | 4 | 28489339 | A | T | 1.46E-02 | 1.97E-03 | 1.10E-13 | 1.24E-03 | 2.01E-03 | 5.40E-01 |
| rs6507054 | 18 | 31248323 | T | C | -9.28E-03 | 1.39E-03 | 2.70E-11 | 8.60E-04 | 1.42E-03 | 5.50E-01 |
| rs9462670 | 6 | 41014309 | G | C | -9.42E-03 | 1.63E-03 | 7.10E-09 | -9.86E-04 | 1.66E-03 | 5.50E-01 |
| rs13275517 | 8 | 143364521 | T | C | -8.15E-03 | 1.39E-03 | 4.10E-09 | -8.14E-04 | 1.42E-03 | 5.70E-01 |
| rs11782074 | 8 | 142617096 | G | T | -9.72E-03 | 1.43E-03 | 1.20E-11 | 8.24E-04 | 1.46E-03 | 5.70E-01 |
| rs10160769 | 11 | 76474827 | G | C | 9.18E-03 | 1.68E-03 | 4.60E-08 | -9.87E-04 | 1.72E-03 | 5.70E-01 |
| rs113079574 | 4 | 147354089 | C | T | 9.65E-03 | 1.75E-03 | 3.30E-08 | -9.93E-04 | 1.78E-03 | 5.80E-01 |
| rs10935143 | 3 | 134665159 | G | A | 8.40E-03 | 1.38E-03 | 1.10E-09 | 7.63E-04 | 1.41E-03 | 5.90E-01 |
| rs1905616 | 8 | 93235675 | G | A | 8.20E-03 | 1.46E-03 | 1.80E-08 | 8.02E-04 | 1.49E-03 | 5.90E-01 |
| rs12072739 | 1 | 98315893 | A | G | -1.16E-02 | 1.64E-03 | 1.80E-12 | -8.95E-04 | 1.68E-03 | 5.90E-01 |
| rs12037905 | 1 | 219628036 | C | T | 7.93E-03 | 1.39E-03 | 1.10E-08 | 7.49E-04 | 1.42E-03 | 6.00E-01 |
| rs8134638 | 21 | 40644170 | T | C | -7.80E-03 | 1.42E-03 | 4.00E-08 | 7.68E-04 | 1.45E-03 | 6.00E-01 |
| rs12033257 | 1 | 112318484 | A | G | 9.80E-03 | 1.42E-03 | 4.90E-12 | -7.57E-04 | 1.45E-03 | 6.00E-01 |
| rs2237402 | 7 | 39449768 | G | A | 9.23E-03 | 1.45E-03 | 1.80E-10 | 7.69E-04 | 1.48E-03 | 6.00E-01 |
| rs61985411 | 14 | 41336102 | T | A | -1.65E-02 | 2.76E-03 | 2.10E-09 | -1.47E-03 | 2.82E-03 | 6.00E-01 |
| rs809955 | 4 | 140874760 | G | A | 1.01E-02 | 1.42E-03 | 1.40E-12 | -7.30E-04 | 1.46E-03 | 6.20E-01 |
| rs8192675 | 3 | 170724883 | T | C | -1.17E-02 | 1.51E-03 | 8.70E-15 | -7.69E-04 | 1.54E-03 | 6.20E-01 |
| rs7331420 | 13 | 99236471 | G | A | 9.14E-03 | 1.53E-03 | 2.10E-09 | -7.63E-04 | 1.56E-03 | 6.20E-01 |
| rs1017529 | 17 | 27912415 | C | A | -1.06E-02 | 1.84E-03 | 7.70E-09 | -9.30E-04 | 1.88E-03 | 6.20E-01 |
| rs2576135 | 13 | 54691442 | T | A | 1.32E-02 | 2.39E-03 | 3.50E-08 | 1.21E-03 | 2.44E-03 | 6.20E-01 |
| rs8011566 | 14 | 42939471 | T | A | -8.73E-03 | 1.39E-03 | 3.50E-10 | -6.89E-04 | 1.42E-03 | 6.30E-01 |
| rs39674 | 16 | 9413210 | C | G | -8.71E-03 | 1.50E-03 | 6.00E-09 | -7.29E-04 | 1.53E-03 | 6.30E-01 |
| rs2433733 | 2 | 230816703 | G | A | 1.05E-02 | 1.46E-03 | 7.30E-13 | 6.91E-04 | 1.50E-03 | 6.40E-01 |
| rs36131051 | 3 | 107888841 | T | G | 1.08E-02 | 1.71E-03 | 2.90E-10 | 8.28E-04 | 1.75E-03 | 6.40E-01 |
| rs112380819 | 3 | 9498519 | G | A | -1.37E-02 | 2.26E-03 | 1.30E-09 | -1.05E-03 | 2.31E-03 | 6.50E-01 |
| rs147730268 | 12 | 123024476 | G | T | 2.28E-02 | 2.49E-03 | 4.00E-20 | -1.15E-03 | 2.54E-03 | 6.50E-01 |
| rs62259692 | 3 | 51847709 | G | A | -1.78E-02 | 2.93E-03 | 1.40E-09 | 1.34E-03 | 3.00E-03 | 6.50E-01 |
| rs13186637 | 5 | 153108558 | T | C | 8.68E-03 | 1.44E-03 | 1.70E-09 | -6.46E-04 | 1.47E-03 | 6.60E-01 |
| rs9395520 | 6 | 13183523 | C | T | 9.76E-03 | 1.49E-03 | 5.90E-11 | 6.65E-04 | 1.52E-03 | 6.60E-01 |
| rs10927006 | 1 | 243557659 | T | C | 1.20E-02 | 1.95E-03 | 8.30E-10 | 8.84E-04 | 2.00E-03 | 6.60E-01 |
| rs10204994 | 2 | 35443726 | G | A | 1.01E-02 | 1.63E-03 | 5.30E-10 | 7.02E-04 | 1.66E-03 | 6.70E-01 |
| rs10499014 | 6 | 97947755 | C | G | 1.00E-02 | 1.56E-03 | 1.10E-10 | -6.65E-04 | 1.59E-03 | 6.80E-01 |
| rs533493779 | 2 | 104447054 | A | T | -8.69E-03 | 1.42E-03 | 1.10E-09 | -5.76E-04 | 1.46E-03 | 6.90E-01 |
| rs4660586 | 1 | 42407229 | C | T | 8.96E-03 | 1.56E-03 | 1.00E-08 | -6.28E-04 | 1.60E-03 | 6.90E-01 |
| rs7020196 | 9 | 12289527 | C | T | 7.79E-03 | 1.41E-03 | 3.60E-08 | 5.52E-04 | 1.44E-03 | 7.00E-01 |
| rs1865341 | 9 | 8845911 | C | T | -9.00E-03 | 1.62E-03 | 2.50E-08 | -6.28E-04 | 1.65E-03 | 7.00E-01 |
| rs12681792 | 8 | 62054463 | C | A | -9.56E-03 | 1.75E-03 | 4.30E-08 | -6.92E-04 | 1.78E-03 | 7.00E-01 |
| rs59227842 | 11 | 43692423 | A | G | -1.52E-02 | 1.49E-03 | 1.80E-24 | -5.69E-04 | 1.53E-03 | 7.10E-01 |
| rs13427822 | 2 | 213414265 | A | G | 9.45E-03 | 1.56E-03 | 1.30E-09 | -5.84E-04 | 1.59E-03 | 7.10E-01 |
| rs270689 | 6 | 104790532 | A | T | -1.01E-02 | 1.72E-03 | 4.20E-09 | -6.49E-04 | 1.76E-03 | 7.10E-01 |
| rs6478538 | 9 | 124627012 | A | G | 8.81E-03 | 1.47E-03 | 1.90E-09 | -5.27E-04 | 1.50E-03 | 7.30E-01 |
| rs2616143 | 8 | 20632022 | G | A | 8.85E-03 | 1.47E-03 | 1.90E-09 | 5.15E-04 | 1.51E-03 | 7.30E-01 |
| rs72976986 | 19 | 4050424 | G | A | 1.39E-02 | 1.77E-03 | 4.40E-15 | 6.31E-04 | 1.80E-03 | 7.30E-01 |
| rs9515446 | 13 | 112217108 | A | G | -9.69E-03 | 1.38E-03 | 2.20E-12 | -4.59E-04 | 1.41E-03 | 7.40E-01 |
| rs4759073 | 12 | 54653258 | G | A | 9.35E-03 | 1.39E-03 | 1.90E-11 | 4.35E-04 | 1.42E-03 | 7.60E-01 |
| rs10169594 | 2 | 41637688 | T | C | -7.88E-03 | 1.43E-03 | 3.30E-08 | -4.55E-04 | 1.46E-03 | 7.60E-01 |
| rs2744801 | 1 | 41155486 | C | T | 7.95E-03 | 1.45E-03 | 4.30E-08 | 4.44E-04 | 1.48E-03 | 7.60E-01 |
| rs149778057 | 13 | 31007805 | A | C | 9.05E-03 | 1.53E-03 | 3.80E-09 | 4.37E-04 | 1.57E-03 | 7.80E-01 |
| rs1320251 | 17 | 21264396 | C | T | 1.24E-02 | 1.38E-03 | 3.20E-19 | -3.69E-04 | 1.41E-03 | 7.90E-01 |
| rs7255223 | 19 | 32824310 | C | A | 8.97E-03 | 1.56E-03 | 8.50E-09 | 4.27E-04 | 1.59E-03 | 7.90E-01 |
| rs60497719 | 19 | 33971746 | G | A | -9.67E-03 | 1.58E-03 | 1.00E-09 | -4.30E-04 | 1.62E-03 | 7.90E-01 |
| rs4077093 | 12 | 51593616 | T | G | 9.69E-03 | 1.68E-03 | 7.90E-09 | 4.66E-04 | 1.72E-03 | 7.90E-01 |
| rs11691869 | 2 | 100805996 | C | A | 1.12E-02 | 1.43E-03 | 3.50E-15 | -3.65E-04 | 1.46E-03 | 8.00E-01 |
| rs10779835 | 1 | 230299949 | T | C | 8.01E-03 | 1.41E-03 | 1.20E-08 | 3.37E-04 | 1.44E-03 | 8.10E-01 |
| rs12971645 | 19 | 45807945 | G | A | 8.59E-03 | 1.54E-03 | 2.60E-08 | 3.43E-04 | 1.58E-03 | 8.30E-01 |
| rs2237025 | 4 | 55541879 | T | C | 1.02E-02 | 1.39E-03 | 1.80E-13 | -2.88E-04 | 1.42E-03 | 8.40E-01 |
| rs9529148 | 13 | 67419495 | G | A | -8.00E-03 | 1.42E-03 | 1.90E-08 | -2.79E-04 | 1.45E-03 | 8.50E-01 |
| rs3759584 | 14 | 103990799 | T | C | 9.74E-03 | 1.43E-03 | 1.10E-11 | 2.76E-04 | 1.46E-03 | 8.50E-01 |
| rs7601895 | 2 | 55281901 | C | G | 1.03E-02 | 1.49E-03 | 4.10E-12 | 2.78E-04 | 1.52E-03 | 8.50E-01 |
| rs7498044 | 15 | 92573639 | G | A | 9.95E-03 | 1.68E-03 | 3.20E-09 | -3.19E-04 | 1.72E-03 | 8.50E-01 |
| rs4916229 | 1 | 171443368 | C | G | -1.51E-02 | 2.33E-03 | 8.90E-11 | 3.77E-04 | 2.39E-03 | 8.70E-01 |
| rs35852935 | 4 | 17991522 | A | C | -2.19E-02 | 3.74E-03 | 4.90E-09 | 6.39E-04 | 3.82E-03 | 8.70E-01 |
| rs13104584 | 4 | 80811227 | G | A | -8.64E-03 | 1.40E-03 | 6.20E-10 | -1.93E-04 | 1.43E-03 | 8.90E-01 |
| rs4500770 | 16 | 74658430 | A | T | 7.82E-03 | 1.43E-03 | 4.30E-08 | 1.96E-04 | 1.46E-03 | 8.90E-01 |
| rs71495049 | 10 | 34014435 | G | A | -1.71E-02 | 2.48E-03 | 5.70E-12 | -3.46E-04 | 2.53E-03 | 8.90E-01 |
| rs1229984 | 4 | 100239319 | T | C | -2.30E-02 | 4.16E-03 | 3.20E-08 | 5.90E-04 | 4.26E-03 | 8.90E-01 |
| rs113706999 | 3 | 44159156 | T | A | -2.88E-02 | 4.73E-03 | 1.10E-09 | -6.03E-04 | 4.84E-03 | 9.00E-01 |
| rs58351927 | 17 | 5297038 | A | G | -1.01E-02 | 1.50E-03 | 1.20E-11 | -1.67E-04 | 1.53E-03 | 9.10E-01 |
| rs113569731 | 3 | 47093206 | C | A | -1.52E-02 | 2.41E-03 | 3.10E-10 | -2.93E-04 | 2.47E-03 | 9.10E-01 |
| rs61971082 | 13 | 86494667 | T | G | -1.01E-02 | 1.52E-03 | 3.00E-11 | 1.62E-04 | 1.56E-03 | 9.20E-01 |
| rs9477762 | 6 | 18507853 | A | T | -2.02E-02 | 3.05E-03 | 3.80E-11 | 2.72E-04 | 3.12E-03 | 9.30E-01 |
| rs10185199 | 2 | 40282202 | G | A | 1.00E-02 | 1.56E-03 | 1.60E-10 | 1.00E-04 | 1.60E-03 | 9.50E-01 |
| rs4658403 | 1 | 243832560 | C | T | 1.36E-02 | 1.84E-03 | 1.20E-13 | 9.35E-05 | 1.88E-03 | 9.60E-01 |
| rs80236973 | 3 | 188001014 | C | T | 1.22E-02 | 2.01E-03 | 1.40E-09 | 9.83E-05 | 2.05E-03 | 9.60E-01 |
| rs4425224 | 3 | 56249398 | C | A | 1.24E-02 | 2.24E-03 | 2.70E-08 | -1.19E-04 | 2.29E-03 | 9.60E-01 |
| rs181617194 | 12 | 122011598 | T | C | 2.27E-02 | 3.76E-03 | 1.70E-09 | 1.22E-04 | 3.84E-03 | 9.70E-01 |
| rs28408562 | 15 | 60917079 | C | G | -7.84E-03 | 1.38E-03 | 1.30E-08 | 4.07E-05 | 1.41E-03 | 9.80E-01 |
| rs7206608 | 16 | 82872628 | C | G | -9.79E-03 | 1.47E-03 | 2.60E-11 | 4.55E-05 | 1.50E-03 | 9.80E-01 |
| rs12253527 | 10 | 21819824 | G | A | -1.36E-02 | 1.47E-03 | 2.00E-20 | -9.61E-06 | 1.50E-03 | 9.90E-01 |

**Table S2N.** The genetic variants strongly associated with adulthood adiposity at genome wide significance and not childhood adiposity (exclude child SNPs at P ≤ 0.05) in females.

| SNP | Chromosome | Base position | Effect allele | Other allele | Beta (Age 10) | SE (Age 10) | P (Age 10) | Beta (Adult) | SE (Adult) | P (Adult) |
| --- | --- | --- | --- | --- | --- | --- | --- | --- | --- | --- |
| rs74892851 | 1 | 1563789 | C | A | 1.22E-04 | 2.03E-03 | 9.70E-01 | 1.16E-02 | 2.06E-03 | 1.90E-08 |
| rs78886584 | 1 | 16859325 | A | G | -2.12E-03 | 1.96E-03 | 3.20E-01 | -1.33E-02 | 1.99E-03 | 2.60E-11 |
| rs72660086 | 1 | 39571992 | T | G | -3.11E-03 | 2.37E-03 | 1.90E-01 | -1.33E-02 | 2.41E-03 | 3.20E-08 |
| rs749593242 | 1 | 50224384 | C | CT | 2.39E-03 | 2.08E-03 | 1.70E-01 | 1.28E-02 | 2.11E-03 | 1.30E-09 |
| rs56951135 | 1 | 91211018 | A | ATTT | 3.91E-04 | 2.04E-03 | 8.30E-01 | -1.43E-02 | 2.07E-03 | 4.90E-12 |
| rs75641275 | 1 | 98327133 | A | C | 2.06E-03 | 2.76E-03 | 5.90E-01 | -1.85E-02 | 2.81E-03 | 4.70E-11 |
| rs12033257 | 1 | 112318484 | A | G | -3.09E-04 | 2.00E-03 | 6.80E-01 | 1.22E-02 | 2.04E-03 | 1.90E-09 |
| rs3753639 | 1 | 154986091 | T | C | 7.79E-04 | 2.26E-03 | 8.90E-01 | -1.26E-02 | 2.30E-03 | 3.70E-08 |
| rs61813324 | 1 | 156049877 | C | T | -4.74E-03 | 2.87E-03 | 8.10E-02 | -2.09E-02 | 2.91E-03 | 7.60E-13 |
| rs2994320 | 1 | 243641247 | A | G | -2.73E-04 | 2.45E-03 | 7.90E-01 | 1.66E-02 | 2.49E-03 | 2.40E-11 |
| rs13420048 | 2 | 50751414 | C | A | 6.99E-04 | 2.01E-03 | 5.80E-01 | 1.20E-02 | 2.04E-03 | 4.40E-09 |
| rs6545468 | 2 | 55277641 | C | G | 2.20E-03 | 1.97E-03 | 2.10E-01 | 1.20E-02 | 2.00E-03 | 2.20E-09 |
| rs4671328 | 2 | 58935282 | T | G | 1.65E-03 | 1.96E-03 | 3.20E-01 | 1.37E-02 | 1.99E-03 | 6.50E-12 |
| rs10192894 | 2 | 62838936 | A | G | 1.93E-03 | 1.95E-03 | 3.00E-01 | -1.10E-02 | 1.98E-03 | 2.50E-08 |
| rs12477088 | 2 | 67841326 | T | C | 1.12E-03 | 1.96E-03 | 6.20E-01 | 1.19E-02 | 1.99E-03 | 2.70E-09 |
| rs11691869 | 2 | 100805996 | C | A | 1.03E-03 | 2.02E-03 | 6.30E-01 | 1.55E-02 | 2.05E-03 | 3.30E-14 |
| rs113607259 | 2 | 104343481 | G | GTA | 2.80E-03 | 1.95E-03 | 1.40E-01 | 1.18E-02 | 1.99E-03 | 2.60E-09 |
| rs7602120 | 2 | 144033069 | C | T | -2.71E-03 | 1.95E-03 | 1.50E-01 | -1.34E-02 | 1.98E-03 | 1.20E-11 |
| rs1083472 | 2 | 147873492 | C | G | -8.32E-04 | 1.99E-03 | 8.00E-01 | 1.13E-02 | 2.02E-03 | 2.10E-08 |
| rs4673553 | 2 | 211608379 | T | G | -2.48E-03 | 1.95E-03 | 2.20E-01 | -1.17E-02 | 1.98E-03 | 3.20E-09 |
| rs573105257 | 2 | 230822932 | A | AT | 1.19E-03 | 2.08E-03 | 5.20E-01 | 1.18E-02 | 2.11E-03 | 2.20E-08 |
| rs62242071 | 3 | 20590313 | G | T | 2.93E-03 | 2.08E-03 | 1.70E-01 | 1.18E-02 | 2.11E-03 | 2.30E-08 |
| rs113706999 | 3 | 44159156 | T | A | -5.99E-03 | 6.65E-03 | 3.10E-01 | -3.87E-02 | 6.76E-03 | 1.00E-08 |
| rs1454687 | 3 | 94038085 | C | G | 1.89E-03 | 1.93E-03 | 4.00E-01 | 1.23E-02 | 1.97E-03 | 4.10E-10 |
| rs9811252 | 3 | 128293392 | C | T | 1.04E-03 | 2.03E-03 | 8.10E-01 | 1.14E-02 | 2.07E-03 | 3.90E-08 |
| rs13081671 | 3 | 135876549 | C | T | -6.69E-04 | 2.18E-03 | 7.20E-01 | -1.35E-02 | 2.22E-03 | 1.20E-09 |
| rs529200 | 3 | 173114305 | A | G | -3.02E-03 | 1.94E-03 | 1.90E-01 | -1.16E-02 | 1.97E-03 | 4.00E-09 |
| rs73213484 | 4 | 28489339 | A | T | 2.48E-03 | 2.78E-03 | 4.00E-01 | 1.89E-02 | 2.83E-03 | 2.30E-11 |
| rs148712344 | 4 | 55476318 | G | T | 7.04E-03 | 5.02E-03 | 1.50E-01 | 2.98E-02 | 5.10E-03 | 5.10E-09 |
| rs1603179 | 4 | 67805347 | A | C | -3.60E-03 | 2.03E-03 | 9.20E-02 | 1.14E-02 | 2.06E-03 | 2.90E-08 |
| rs11098965 | 4 | 80888040 | C | T | 6.51E-04 | 2.22E-03 | 7.00E-01 | 1.36E-02 | 2.26E-03 | 1.60E-09 |
| rs2199936 | 4 | 89045331 | A | G | -6.25E-03 | 3.05E-03 | 5.20E-02 | -1.83E-02 | 3.10E-03 | 3.30E-09 |
| rs182851732 | 4 | 90684766 | A | G | 1.31E-02 | 1.18E-02 | 2.90E-01 | 6.96E-02 | 1.20E-02 | 7.70E-09 |
| rs769668 | 4 | 140858717 | T | C | 1.97E-04 | 2.04E-03 | 9.70E-01 | 1.40E-02 | 2.08E-03 | 1.80E-11 |
| rs35390852 | 4 | 143067054 | G | A | -2.05E-03 | 2.94E-03 | 4.10E-01 | -1.67E-02 | 2.99E-03 | 2.40E-08 |
| rs828550 | 5 | 3539923 | C | T | -1.56E-03 | 2.04E-03 | 4.30E-01 | -1.17E-02 | 2.07E-03 | 1.50E-08 |
| rs55908499 | 5 | 63020950 | G | GA | -2.55E-03 | 1.98E-03 | 2.00E-01 | -1.41E-02 | 2.01E-03 | 2.20E-12 |
| rs191621046 | 5 | 92547517 | G | A | -5.60E-03 | 5.06E-03 | 2.20E-01 | -2.98E-02 | 5.15E-03 | 7.10E-09 |
| rs10623997 | 5 | 107478679 | T | TATAATA | -3.07E-04 | 2.34E-03 | 9.10E-01 | 1.80E-02 | 2.38E-03 | 3.60E-14 |
| rs71579590 | 5 | 139070398 | G | C | -3.73E-03 | 2.74E-03 | 1.80E-01 | -1.88E-02 | 2.79E-03 | 1.70E-11 |
| rs251353 | 5 | 140228164 | C | A | 2.52E-03 | 2.04E-03 | 1.50E-01 | 1.19E-02 | 2.07E-03 | 1.10E-08 |
| rs12658841 | 5 | 153106013 | C | G | -1.18E-03 | 1.95E-03 | 7.80E-01 | 1.17E-02 | 1.98E-03 | 3.80E-09 |
| rs9395520 | 6 | 13183523 | C | T | 1.09E-03 | 2.10E-03 | 5.80E-01 | 1.25E-02 | 2.14E-03 | 4.60E-09 |
| rs77253887 | 6 | 35626932 | G | C | 1.41E-03 | 5.40E-03 | 7.90E-01 | -3.03E-02 | 5.49E-03 | 3.50E-08 |
| rs567230078 | 6 | 43588227 | T | A | 8.43E-03 | 5.99E-03 | 2.40E-01 | 3.40E-02 | 6.09E-03 | 2.50E-08 |
| rs9387640 | 6 | 119508871 | C | T | 3.06E-03 | 2.01E-03 | 1.30E-01 | 1.18E-02 | 2.05E-03 | 9.20E-09 |
| rs73046311 | 7 | 1854159 | C | G | 1.70E-03 | 2.65E-03 | 5.40E-01 | 1.57E-02 | 2.69E-03 | 5.70E-09 |
| rs2866720 | 7 | 70106310 | C | T | -1.60E-03 | 2.00E-03 | 3.10E-01 | -1.18E-02 | 2.04E-03 | 7.00E-09 |
| rs236660 | 7 | 75050086 | T | C | -2.89E-03 | 2.03E-03 | 1.50E-01 | -1.49E-02 | 2.06E-03 | 5.40E-13 |
| rs369428586 | 7 | 99134799 | CA | C | -2.52E-03 | 2.73E-03 | 4.00E-01 | 1.56E-02 | 2.78E-03 | 2.00E-08 |
| rs2396625 | 7 | 113028634 | T | A | 3.73E-04 | 1.97E-03 | 7.10E-01 | 1.18E-02 | 2.00E-03 | 3.40E-09 |
| rs1840661 | 7 | 114352682 | T | A | 3.18E-03 | 1.97E-03 | 1.50E-01 | -1.22E-02 | 2.00E-03 | 1.20E-09 |
| rs6557829 | 8 | 21973970 | C | A | 1.90E-03 | 1.97E-03 | 3.10E-01 | -1.16E-02 | 2.00E-03 | 6.90E-09 |
| rs117176448 | 8 | 27261138 | C | G | -4.48E-03 | 3.28E-03 | 1.80E-01 | -1.87E-02 | 3.34E-03 | 2.00E-08 |
| rs10957605 | 8 | 73433886 | C | T | 8.68E-04 | 2.08E-03 | 8.10E-01 | 1.58E-02 | 2.12E-03 | 9.80E-14 |
| rs17716502 | 8 | 116659731 | C | T | 2.81E-03 | 2.42E-03 | 1.90E-01 | 1.79E-02 | 2.46E-03 | 3.50E-13 |
| rs13292699 | 9 | 15910044 | A | C | -2.66E-03 | 1.96E-03 | 1.90E-01 | 1.50E-02 | 1.99E-03 | 4.30E-14 |
| rs10962552 | 9 | 16723742 | C | T | -1.73E-03 | 2.61E-03 | 5.20E-01 | -1.46E-02 | 2.65E-03 | 3.60E-08 |
| rs377741138 | 9 | 28412183 | GAAAA | G | -2.70E-03 | 2.05E-03 | 2.20E-01 | -1.61E-02 | 2.09E-03 | 1.10E-14 |
| rs6478538 | 9 | 124627012 | A | G | 6.79E-04 | 2.07E-03 | 5.70E-01 | 1.15E-02 | 2.10E-03 | 4.60E-08 |
| rs3003578 | 9 | 130994179 | C | T | 2.25E-03 | 1.96E-03 | 2.10E-01 | 1.12E-02 | 2.00E-03 | 1.80E-08 |
| rs1270799 | 10 | 21907423 | T | G | -2.76E-03 | 2.11E-03 | 1.70E-01 | -1.65E-02 | 2.14E-03 | 1.20E-14 |
| rs113585475 | 10 | 33985434 | C | T | 1.87E-03 | 3.27E-03 | 4.70E-01 | 2.20E-02 | 3.32E-03 | 3.60E-11 |
| rs1250535 | 10 | 81016112 | C | G | 2.24E-03 | 2.08E-03 | 2.20E-01 | -1.19E-02 | 2.11E-03 | 1.80E-08 |
| rs12357890 | 10 | 99762693 | A | G | 6.41E-04 | 1.96E-03 | 7.90E-01 | -1.48E-02 | 1.99E-03 | 1.20E-13 |
| rs10510025 | 10 | 118650996 | C | T | 2.28E-04 | 2.25E-03 | 7.90E-01 | -1.33E-02 | 2.29E-03 | 6.90E-09 |
| rs141090474 | 10 | 126729168 | TAC | T | 3.21E-03 | 2.00E-03 | 1.00E-01 | 1.15E-02 | 2.03E-03 | 1.40E-08 |
| rs201102222 | 10 | 134005569 | A | AT | 2.02E-03 | 1.96E-03 | 3.60E-01 | 1.15E-02 | 1.99E-03 | 7.60E-09 |
| rs60572790 | 11 | 43692383 | T | C | -1.62E-03 | 2.09E-03 | 5.20E-01 | -1.65E-02 | 2.12E-03 | 7.20E-15 |
| rs34292685 | 11 | 64049021 | C | T | 4.46E-03 | 2.63E-03 | 8.00E-02 | 1.80E-02 | 2.67E-03 | 1.60E-11 |
| rs11218510 | 11 | 121922587 | G | A | 2.91E-03 | 1.98E-03 | 2.00E-01 | 1.12E-02 | 2.01E-03 | 2.90E-08 |
| rs7976757 | 12 | 19207948 | T | C | -3.22E-03 | 2.57E-03 | 2.70E-01 | -1.52E-02 | 2.62E-03 | 6.10E-09 |
| rs2292238 | 12 | 56493822 | A | C | 1.21E-03 | 1.97E-03 | 4.40E-01 | 1.22E-02 | 2.01E-03 | 1.30E-09 |
| rs61217499 | 12 | 108417780 | G | C | 1.78E-03 | 2.35E-03 | 5.30E-01 | 1.48E-02 | 2.40E-03 | 5.70E-10 |
| rs111828690 | 12 | 117576767 | C | T | -1.01E-03 | 2.35E-03 | 6.50E-01 | -1.37E-02 | 2.39E-03 | 1.00E-08 |
| rs181617194 | 12 | 122011598 | T | C | -4.02E-03 | 5.30E-03 | 4.50E-01 | 3.17E-02 | 5.39E-03 | 4.20E-09 |
| rs3803005 | 12 | 123110654 | T | C | -2.78E-03 | 2.18E-03 | 1.90E-01 | -1.68E-02 | 2.22E-03 | 3.60E-14 |
| rs776543236 | 13 | 67348551 | ATGGAG | A | 6.04E-04 | 2.29E-03 | 7.90E-01 | -1.44E-02 | 2.33E-03 | 6.50E-10 |
| rs116394958 | 13 | 86477072 | C | T | -9.39E-05 | 2.17E-03 | 9.90E-01 | -1.26E-02 | 2.21E-03 | 1.40E-08 |
| rs7331420 | 13 | 99236471 | G | A | -2.37E-04 | 2.15E-03 | 8.40E-01 | 1.20E-02 | 2.19E-03 | 4.80E-08 |
| rs9522180 | 13 | 111970212 | C | T | 2.22E-03 | 1.95E-03 | 3.00E-01 | 1.08E-02 | 1.98E-03 | 4.40E-08 |
| rs367552155 | 14 | 29721943 | CA | C | 1.78E-03 | 2.29E-03 | 4.90E-01 | 1.44E-02 | 2.33E-03 | 7.40E-10 |
| rs10142359 | 14 | 73884540 | A | G | -2.28E-03 | 1.94E-03 | 2.20E-01 | -1.11E-02 | 1.97E-03 | 1.70E-08 |
| rs11374426 | 14 | 104337630 | G | GA | 2.28E-03 | 2.04E-03 | 2.70E-01 | 1.36E-02 | 2.08E-03 | 6.90E-11 |
| rs715724 | 15 | 80984293 | A | G | 3.43E-03 | 2.03E-03 | 5.70E-02 | 1.17E-02 | 2.06E-03 | 1.40E-08 |
| rs939624 | 15 | 99480551 | C | T | -3.03E-04 | 1.94E-03 | 9.70E-01 | -1.23E-02 | 1.98E-03 | 4.70E-10 |
| rs13329943 | 16 | 24733751 | C | T | -3.48E-03 | 2.19E-03 | 1.40E-01 | -1.28E-02 | 2.22E-03 | 7.90E-09 |
| rs55678940 | 17 | 21251092 | C | CTGTAAAGAAA | 7.31E-04 | 2.04E-03 | 6.60E-01 | 1.38E-02 | 2.08E-03 | 3.00E-11 |
| rs11079849 | 17 | 47090785 | C | T | -2.10E-03 | 2.06E-03 | 3.30E-01 | 1.27E-02 | 2.10E-03 | 1.60E-09 |
| rs77706698 | 17 | 65953348 | G | A | -3.42E-03 | 2.87E-03 | 2.30E-01 | -1.72E-02 | 2.92E-03 | 4.20E-09 |
| rs2619976 | 17 | 71754545 | C | T | -3.26E-03 | 1.98E-03 | 1.40E-01 | -1.17E-02 | 2.01E-03 | 5.40E-09 |
| rs11660335 | 18 | 22154235 | T | C | 3.65E-03 | 2.47E-03 | 1.20E-01 | 1.59E-02 | 2.52E-03 | 2.50E-10 |
| rs784257 | 18 | 53397199 | T | C | 5.24E-05 | 2.49E-03 | 9.20E-01 | -1.41E-02 | 2.53E-03 | 2.90E-08 |
| rs149080927 | 19 | 1854253 | G | GC | -8.89E-04 | 1.96E-03 | 5.80E-01 | -1.24E-02 | 1.99E-03 | 5.90E-10 |
| rs350832 | 19 | 4069426 | G | A | 3.72E-04 | 2.32E-03 | 6.20E-01 | -1.37E-02 | 2.36E-03 | 6.50E-09 |
| rs429358 | 19 | 45411941 | T | C | -2.48E-03 | 2.68E-03 | 3.60E-01 | 1.75E-02 | 2.73E-03 | 1.40E-10 |
| rs8124896 | 20 | 21385659 | T | C | -5.99E-03 | 3.21E-03 | 9.90E-02 | -2.01E-02 | 3.27E-03 | 6.90E-10 |
| rs116948922 | 20 | 25534854 | C | T | 2.83E-03 | 5.52E-03 | 7.20E-01 | 3.40E-02 | 5.62E-03 | 1.50E-09 |
| rs151157954 | 20 | 51171126 | A | ATG | 4.59E-03 | 2.65E-03 | 7.60E-02 | 1.78E-02 | 2.69E-03 | 3.50E-11 |
| rs915814 | 21 | 46493003 | G | A | 1.04E-03 | 2.27E-03 | 7.00E-01 | 1.31E-02 | 2.31E-03 | 1.50E-08 |
| rs400997 | 21 | 46564154 | T | A | -2.31E-03 | 1.96E-03 | 2.80E-01 | -1.43E-02 | 2.00E-03 | 8.00E-13 |
| rs738140 | 22 | 41884954 | A | G | 1.13E-03 | 2.09E-03 | 6.30E-01 | 1.20E-02 | 2.13E-03 | 1.80E-08 |

**Table S3A.** Univariable and multivariable Mendelian randomization (MR) analyses using structural mean modelling (SMM) for child and adult adiposity on cardiovascular disease (CVD) - using UKB outcome data.

| Exposure | Outcome | Risk difference | Standard error | P value | Sample size | MR |
| --- | --- | --- | --- | --- | --- | --- |
| Child associated SNPs* - regardless of adult SNP association | CVD | 0.021 | 0.004 | 1.75E-08 | 452960 | Univariable MR |
| Adult associated SNPs* - regardless of child SNP association | CVD | 0.062 | 0.003 | <2E-16 | 452960 | Univariable MR |
| Child associated SNPs* - regardless of adult SNP association, conditioning on adult associated SNPs | CVD | -0.024 | 0.005 | 3.92E-06 | 452960 | Multivariable MR |
| Adult associated SNPs* - regardless of child SNP association, conditioning on child associated SNPs | CVD | 0.074 | 0.004 | <2E-16 | 452960 | Multivariable MR |
| Child associated SNPs* - exclude adult associated SNPs at P ≤ 5*10^-8^ (genome-wide significance) | CVD | 0.004 | 0.006 | 0.419 | 452960 | Univariable MR |
| Adult associated SNPs* - exclude child associated SNPs at P ≤ 5*10^-8^ (genome-wide significance) | CVD | 0.067 | 0.004 | <2E-16 | 452960 | Univariable MR |
| Child associated SNPs* - exclude adult associated SNPs at P ≤ 0.05 with Bonferroni correction | CVD | 0.002 | 0.007 | 0.801 | 452960 | Univariable MR |
| Adult associated SNPs* - exclude child associated SNPs at P ≤ 0.05 with Bonferroni correction | CVD | 0.065 | 0.004 | <2E-16 | 452960 | Univariable MR |
| Child associated SNPs* - exclude adult associated SNPs at P ≤ 0.05 | CVD | -0.002 | 0.010 | 0.809 | 452960 | Univariable MR |
| Adult associated SNPs* - exclude child associated SNPs at P ≤ 0.05 | CVD | 0.070 | 0.005 | <2E-16 | 452960 | Univariable MR |

*Associated SNPs refers to strongly associated at genome wide significance (P≤ 5×10^-8^)

**Table S3B.** Univariable and multivariable Mendelian randomization analyses using structural mean models for child and adult adiposity on type 2 diabetes (T2D) - using UKB outcome data.

| Exposure | Outcome | Risk difference | Standard error | P value | Sample size | MR |
| --- | --- | --- | --- | --- | --- | --- |
| Child associated SNPs* - regardless of adult SNP association | T2D | 0.049 | 0.003 | <2E-16 | 452960 | Univariable MR |
| Adult associated SNPs* - regardless of child SNP association | T2D | 0.107 | 0.003 | <2E-16 | 452960 | Univariable MR |
| Child associated SNPs* - regardless of adult SNP association, conditioning on adult associated SNPs | T2D | -0.024 | 0.004 | 5.39E-08 | 452960 | Multivariable MR |
| Adult associated SNPs* - regardless of child SNP association, conditioning on child associated SNPs | T2D | 0.118 | 0.004 | <2E-16 | 452960 | Multivariable MR |
| Child associated SNPs* - exclude adult associated SNPs at P ≤ 5*10^-8^ (genome-wide significance) | T2D | 0.018 | 0.005 | 1.64E-04 | 452960 | Univariable MR |
| Adult associated SNPs* - exclude child associated SNPs at P ≤ 5*10^-8^ (genome-wide significance) | T2D | 0.109 | 0.003 | <2E-16 | 452960 | Univariable MR |
| Child associated SNPs* - exclude adult associated SNPs at P ≤ 0.05 with Bonferroni correction | T2D | -0.004 | 0.006 | 4.69E-01 | 452960 | Univariable MR |
| Adult associated SNPs* - exclude child associated SNPs at P ≤ 0.05 with Bonferroni correction | T2D | 0.108 | 0.003 | <2E-16 | 452960 | Univariable MR |
| Child associated SNPs* - exclude adult associated SNPs at P ≤ 0.05 | T2D | -0.008 | 0.008 | 0.364 | 452960 | Univariable MR |
| Adult associated SNPs* - exclude child associated SNPs at P ≤ 0.05 | T2D | 0.115 | 0.004 | <2E-16 | 452960 | Univariable MR |

*Associated SNPs refers to strongly associated at genome wide significance (P≤ 5×10^-8^)

**Table S3C.** Univariable and multivariable Mendelian randomization analyses using structural mean models for child and adult adiposity on breast cancer - using UKB outcome data.

| Exposure | Outcome | Risk difference | Standard error | P value | Sample size | MR | Population |
| --- | --- | --- | --- | --- | --- | --- | --- |
| Child associated SNPs* - regardless of adult SNP association | Breast cancer | -0.032 | 0.005 | 6.72E-12 | 246404 | Univariable MR | Female |
| Adult associated SNPs* - regardless of child SNP association | Breast cancer | -0.008 | 0.004 | 0.053 | 246404 | Univariable MR | Female |
| Child associated SNPs* - regardless of adult SNP association, conditioning on adult associated SNPs | Breast cancer | -0.040 | 0.007 | 8.27E-10 | 246404 | Multivariable MR | Female |
| Adult associated SNPs* - regardless of child SNP association, conditioning on child associated SNPs | Breast cancer | 0.013 | 0.006 | 0.03 | 246404 | Multivariable MR | Female |
| Child associated SNPs* - exclude adult associated SNPs at P ≤ 5*10^-8^ (genome-wide significance) | Breast cancer | -0.029 | 0.007 | 3.42E-05 | 246404 | Univariable MR | Female |
| Adult associated SNPs* - exclude child associated SNPs at P ≤ 5*10^-8^ (genome-wide significance) | Breast cancer | 0.006 | 0.005 | 1.83E-01 | 246404 | Univariable MR | Female |
| Child associated SNPs* - exclude adult associated SNPs at P ≤ 0.05 with Bonferroni correction | Breast cancer | -0.029 | 0.009 | 9.22E-04 | 246407 | Univariable MR | Female |
| Adult associated SNPs* - exclude child associated SNPs at P ≤ 0.05 with Bonferroni correction | Breast cancer | 0.014 | 0.005 | 0.0106 | 246408 | Univariable MR | Female |
| Child associated SNPs* - exclude adult associated SNPs at P ≤ 0.05 | Breast cancer | -0.035 | 0.012 | 2.93E-03 | 246405 | Univariable MR | Female |
| Adult associated SNPs* - exclude child associated SNPs at P ≤ 0.05 | Breast cancer | 0.017 | 0.006 | 7.15E-03 | 246406 | Univariable MR | Female |

*Associated SNPs refers to strongly associated at genome wide significance (P≤ 5×10^-8^)

**Table S4A.** Univariable and multivariable two-sample Mendelian randomization (MR) analyses using inverse probability weighting for child and adult adiposity on cardiovascular disease (CVD) - using UKB outcome data.

| Exposure | Outcome | nSNP | Risk difference | Standard error | P value | Sample size | MR |
| --- | --- | --- | --- | --- | --- | --- | --- |
| Child associated SNPs* - regardless of adult SNP association | CVD | 313 | 0.022 | 0.005 | 1.28E-06 | 452960 | Univariable MR |
| Adult associated SNPs* - regardless of child SNP association | CVD | 580 | 0.062 | 0.004 | 1.24E-52 | 452960 | Univariable MR |
| Child associated SNPs* - regardless of adult SNP association, conditioning on adult associated SNPs | CVD | 267 | -0.021 | 0.006 | 1.18E-03 | 452960 | Multivariable MR |
| Adult associated SNPs* - regardless of child SNP association, conditioning on child associated SNPs | CVD | 535 | 0.070 | 0.006 | 9.59E-37 | 452960 | Multivariable MR |
| Child associated SNPs* - exclude adult associated SNPs at P ≤ 5*10^-8^ (genome-wide significance) | CVD | 180 | 0.007 | 0.006 | 2.93E-01 | 452960 | Univariable MR |
| Adult associated SNPs* - exclude child associated SNPs at P ≤ 5*10^-8^ (genome-wide significance) | CVD | 439 | 0.069 | 0.005 | 5.35E-43 | 452960 | Univariable MR |
| Child associated SNPs* - exclude adult associated SNPs at P ≤ 0.05 with Bonferroni correction | CVD | 106 | 0.006 | 0.010 | 5.43E-01 | 452960 | Univariable MR |
| Adult associated SNPs* - exclude child associated SNPs at P ≤ 0.05 with Bonferroni correction | CVD | 365 | 0.067 | 0.006 | 8.80E-32 | 452960 | Univariable MR |
| Child associated SNPs* - exclude adult associated SNPs at P ≤ 0.05 | CVD | 55 | -0.005 | 0.014 | 7.51E-01 | 452960 | Univariable MR |
| Adult associated SNPs* - exclude child associated SNPs at P ≤ 0.05 | CVD | 218 | 0.071 | 0.008 | 2.88E-21 | 452960 | Univariable MR |

*Associated SNPs refers to strongly associated at genome wide significance (P≤ 5×10^-8^)

**Table S4B.** Univariable and multivariable two-sample Mendelian randomization (MR) analyses using inverse probability weighting for child and adult adiposity on type 2 diabetes (T2D) - using UKB outcome data.

| Exposure | Outcome | nSNP | Risk difference | Standard error | P value | Sample size | MR |
| --- | --- | --- | --- | --- | --- | --- | --- |
| Child associated SNPs* - regardless of adult SNP association | T2D | 313 | 0.055 | 0.005 | 2.09E-29 | 452960 | Univariable MR |
| Adult associated SNPs* - regardless of child SNP association | T2D | 580 | 0.113 | 0.005 | 1.39E-134 | 452960 | Univariable MR |
| Child associated SNPs* - regardless of adult SNP association, conditioning on adult associated SNPs | T2D | 267 | -0.022 | 0.007 | 1.87E-03 | 452960 | Multivariable MR |
| Adult associated SNPs* - regardless of child SNP association, conditioning on child associated SNPs | T2D | 535 | 0.123 | 0.006 | 4.20E-91 | 452960 | Multivariable MR |
| Child associated SNPs* - exclude adult associated SNPs at P ≤ 5*10^-8^ (genome-wide significance) | T2D | 180 | 0.021 | 0.007 | 2.60E-03 | 452960 | Univariable MR |
| Adult associated SNPs* - exclude child associated SNPs at P ≤ 5*10^-8^ (genome-wide significance) | T2D | 439 | 0.112 | 0.006 | 1.64E-77 | 452960 | Univariable MR |
| Child associated SNPs* - exclude adult associated SNPs at P ≤ 0.05 with Bonferroni correction | T2D | 106 | -0.005 | 0.010 | 6.33E-01 | 452960 | Univariable MR |
| Adult associated SNPs* - exclude child associated SNPs at P ≤ 0.05 with Bonferroni correction | T2D | 365 | 0.111 | 0.007 | 6.61E-56 | 452960 | Univariable MR |
| Child associated SNPs* - exclude adult associated SNPs at P ≤ 0.05 | T2D | 55 | -0.011 | 0.016 | 4.72E-01 | 452960 | Univariable MR |
| Adult associated SNPs* - exclude child associated SNPs at P ≤ 0.05 | T2D | 218 | 0.117 | 0.006 | 5.58E-80 | 452960 | Univariable MR |

*Associated SNPs refers to strongly associated at genome wide significance (P≤ 5×10^-8^)

**Table S4C.** Univariable and multivariable two-sample Mendelian randomization (MR) analyses using inverse probability weighting for child and adult adiposity on breast cancer - using UKB outcome data.

| Exposure | Outcome | nSNP | Risk difference | Standard error | P value | Sample size | MR | Population |
| --- | --- | --- | --- | --- | --- | --- | --- | --- |
| Child associated SNPs* - regardless of adult SNP association | Breast cancer | 142 | -0.018 | 0.003 | 1.34E-09 | 246404 | Univariable MR | Female |
| Adult associated SNPs* - regardless of child SNP association | Breast cancer | 221 | -0.004 | 0.003 | 2.06E-01 | 246404 | Univariable MR | Female |
| Child associated SNPs* - regardless of adult SNP association, conditioning on adult associated SNPs | Breast cancer | 132 | -0.023 | 0.004 | 1.08E-09 | 246404 | Multivariable MR | Female |
| Adult associated SNPs* - regardless of child SNP association, conditioning on child associated SNPs | Breast cancer | 202 | 0.009 | 0.004 | 1.58E-02 | 246404 | Multivariable MR | Female |
| Child associated SNPs* - exclude adult associated SNPs at P ≤ 5*10^-8^ (genome-wide significance) | Breast cancer | 95 | -0.015 | 0.004 | 4.49E-05 | 246404 | Univariable MR | Female |
| Adult associated SNPs* - exclude child associated SNPs at P ≤ 5*10^-8^ (genome-wide significance) | Breast cancer | 172 | 0.004 | 0.003 | 2.27E-01 | 246406 | Univariable MR | Female |
| Child associated SNPs* - exclude adult associated SNPs at P ≤ 0.05 with Bonferroni correction | Breast cancer | 50 | -0.015 | 0.006 | 5.23E-03 | 246405 | Univariable MR | Female |
| Adult associated SNPs* - exclude child associated SNPs at P ≤ 0.05 with Bonferroni correction | Breast cancer | 147 | 0.008 | 0.003 | 2.04E-02 | 246407 | Univariable MR | Female |
| Child associated SNPs* - exclude adult associated SNPs at P ≤ 0.05 | Breast cancer | 28 | -0.018 | 0.006 | 4.98E-03 | 246405 | Univariable MR | Female |
| Adult associated SNPs* - exclude child associated SNPs at P ≤ 0.05 | Breast cancer | 102 | 0.010 | 0.004 | 2.41E-02 | 246408 | Univariable MR | Female |

*Associated SNPs refers to strongly associated at genome wide significance (P≤ 5×10^-8^)

**Table S5A.** Univariable and multivariable two-sample Mendelian randomization (MR) analyses using inverse probability weighting for child and adult adiposity on cardiovascular (CVD) – using large scale consortium data.

| Exposure | Outcome | nSNP | Risk difference | Standard error | P value | Sample size | MR |
| --- | --- | --- | --- | --- | --- | --- | --- |
| Child associated SNPs* - regardless of adult SNP association | CVD | 245 | 0.418 | 0.065 | 9.57E-11 | 452960 | Univariable MR |
| Adult associated SNPs* - regardless of child SNP association | CVD | 463 | 0.554 | 0.056 | 6.71E-23 | 452960 | Univariable MR |
| Child associated SNPs* - regardless of adult SNP association, conditioning on adult associated SNPs | CVD | 169 | -0.154 | 0.226 | 4.96E-01 | 452960 | Multivariable MR |
| Adult associated SNPs* - regardless of child SNP association, conditioning on child associated SNPs | CVD | 318 | 1.126 | 0.195 | 7.65E-09 | 452960 | Multivariable MR |
| Child associated SNPs* - exclude adult associated SNPs at P ≤ 5*10^-8^ (genome-wide significance) | CVD | 148 | 0.276 | 0.095 | 3.82E-03 | 452960 | Univariable MR |
| Adult associated SNPs* - exclude child associated SNPs at P ≤ 5*10^-8^ (genome-wide significance) | CVD | 362 | 0.556 | 0.074 | 6.36E-14 | 452960 | Univariable MR |
| Child associated SNPs* - exclude adult associated SNPs at P ≤ 0.05 with Bonferroni correction | CVD | 85 | 0.243 | 0.135 | 7.21E-02 | 452960 | Univariable MR |
| Adult associated SNPs* - exclude child associated SNPs at P ≤ 0.05 with Bonferroni correction | CVD | 297 | 0.502 | 0.087 | 6.59E-09 | 452960 | Univariable MR |
| Child associated SNPs* - exclude adult associated SNPs at P ≤ 0.05 | CVD | 47 | -0.027 | 0.181 | 8.83E-01 | 452960 | Univariable MR |
| Adult associated SNPs* - exclude child associated SNPs at P ≤ 0.05 | CVD | 172 | 0.548 | 0.116 | 2.40E-06 | 452960 | Univariable MR |

*Associated SNPs refers to strongly associated at genome wide significance (P≤ 5×10^-8^)

**Table S5B.** Univariable and multivariable two-sample Mendelian randomization (MR) analyses using inverse probability weighting for child and adult adiposity on type 2 diabetes (T2D) – using large scale consortium data.

| Exposure | Outcome | nSNP | Risk difference | Standard error | P value | Sample size | MR |
| --- | --- | --- | --- | --- | --- | --- | --- |
| Child associated SNPs* - regardless of adult SNP association | T2D | 163 | 0.738 | 0.157 | 2.55E-06 | 452960 | Univariable MR |
| Adult associated SNPs* - regardless of child SNP association | T2D | 286 | 1.158 | 0.163 | 1.05E-12 | 452960 | Univariable MR |
| Child associated SNPs* - regardless of adult SNP association, conditioning on adult associated SNPs | T2D | 169 | -0.154 | 0.226 | 4.96E-01 | 452960 | Multivariable MR |
| Adult associated SNPs* - regardless of child SNP association, conditioning on child associated SNPs | T2D | 318 | 1.126 | 0.195 | 7.65E-09 | 452960 | Multivariable MR |
| Child associated SNPs* - exclude adult associated SNPs at P ≤ 5*10^-8^ (genome-wide significance) | T2D | 114 | 0.460 | 0.224 | 3.97E-02 | 452960 | Univariable MR |
| Adult associated SNPs* - exclude child associated SNPs at P ≤ 5*10^-8^ (genome-wide significance) | T2D | 254 | 0.839 | 0.194 | 1.49E-05 | 452960 | Univariable MR |
| Child associated SNPs* - exclude adult associated SNPs at P ≤ 0.05 with Bonferroni correction | T2D | 67 | 0.302 | 0.346 | 3.82E-01 | 452960 | Univariable MR |
| Adult associated SNPs* - exclude child associated SNPs at P ≤ 0.05 with Bonferroni correction | T2D | 208 | 0.799 | 0.231 | 5.51E-04 | 452960 | Univariable MR |
| Child associated SNPs* - exclude adult associated SNPs at P ≤ 0.05 | T2D | 35 | 0.115 | 0.465 | 8.05E-01 | 452960 | Univariable MR |
| Adult associated SNPs* - exclude child associated SNPs at P ≤ 0.05 | T2D | 120 | 1.071 | 0.222 | 1.35E-06 | 452960 | Univariable MR |

*Associated SNPs refers to strongly associated at genome wide significance (P≤ 5×10^-8^)

**Table S5C.** Univariable and multivariable two-sample Mendelian randomization (MR) analyses using inverse probability weighting for child and adult adiposity on breast cancer – using large scale consortium data.

| Exposure | Outcome | nSNP | Risk difference | Standard error | P value | Sample size | MR | Population |
| --- | --- | --- | --- | --- | --- | --- | --- | --- |
| Child associated SNPs* - regardless of adult SNP association | Breast cancer | 114 | -0.460 | 0.072 | 1.63E-10 | 246404 | Univariable MR | Female |
| Adult associated SNPs* - regardless of child SNP association | Breast cancer | 167 | -0.245 | 0.065 | 1.80E-04 | 246404 | Univariable MR | Female |
| Child associated SNPs* - regardless of adult SNP association, conditioning on adult associated SNPs | Breast cancer | 12 | -0.631 | 0.187 | 7.29E-04 | 246404 | Multivariable MR | Female |
| Adult associated SNPs* - regardless of child SNP association, conditioning on child associated SNPs | Breast cancer | 143 | 0.131 | 0.094 | 1.66E-01 | 246404 | Multivariable MR | Female |
| Child associated SNPs* - exclude adult associated SNPs at P ≤ 5*10^-8^ (genome-wide significance) | Breast cancer | 67 | -0.328 | 0.092 | 3.75E-04 | 246404 | Univariable MR | Female |
| Adult associated SNPs* - exclude child associated SNPs at P ≤ 5*10^-8^ (genome-wide significance) | Breast cancer | 110 | 0.039 | 0.074 | 6.03E-01 | 246404 | Univariable MR | Female |
| Child associated SNPs* - exclude adult associated SNPs at P ≤ 0.05 with Bonferroni correction | Breast cancer | 34 | -0.237 | 0.103 | 2.13E-02 | 246404 | Univariable MR | Female |
| Adult associated SNPs* - exclude child associated SNPs at P ≤ 0.05 with Bonferroni correction | Breast cancer | 97 | 0.075 | 0.080 | 3.51E-01 | 246406 | Univariable MR | Female |
| Child associated SNPs* - exclude adult associated SNPs at P ≤ 0.05 | Breast cancer | 18 | -0.297 | 0.158 | 6.03E-02 | 246407 | Univariable MR | Female |
| Adult associated SNPs* - exclude child associated SNPs at P ≤ 0.05 | Breast cancer | 68 | 0.089 | 0.094 | 3.44E-01 | 246408 | Univariable MR | Female |

*Associated SNPs refers to strongly associated at genome wide significance (P≤ 5×10^-8^)

**Table S6.** Description on time-varying confounding in the context of this study.

| **Confounding type** | **Description** |
| --- | --- |
| Time-varying | We have assumed throughout our study that there is no time-varying confounding. Time-varying confounding occurs when earlier measures of the exposure causally affect confounders of later exposures (3), creating a feedback loop over time between the exposure and the confounder. Later values of the exposure will become affected by the confounder. This can complicate efforts when trying to discern the effects of different combinations of an intervention on the exposure of interest at different time points. Although methods exist to address time-varying confounding in traditional conventional studies (4), its implications for MR estimation are less well-explored. Importantly for our analyses, time-varying confounding does not bias estimation when every timepoint is assumed to shift proportionally in response to an intervention and the separate effects of the separate time points cannot be identified. Time-varying confounding is relevant for the period and lifetime effects we consider here. In these settings IVW-MR estimates the effect of a shift in the liability to the exposure, which is constant over the period, although it may have different associations with the exposure at different points. SMM-MR assumes a constant effect of the genetic instruments on the exposure across the time period and estimates a single effect for the period. These approaches do not try to identify effects of different combinations of exposures within that time period. The feedback loops generated by time varying confounding will however form part of the causal pathway from some of the exposures to the outcome that is being estimated. |

**Figure S1.** Causal risk difference estimates from univariable and multivariable MR using inverse variance weighted models (IVW) for childhood and adult adiposity period effects on the outcome measures listed using large scale consortia data referenced in manuscript. (A) Childhood and adult adiposity period and lifetime effects on cardiovascular disease (CVD). (B) Childhood and adult adiposity period and lifetime effects on Type 2 diabetes (T2D). (C) Childhood and adult adiposity period and lifetime effects on breast cancer.

A


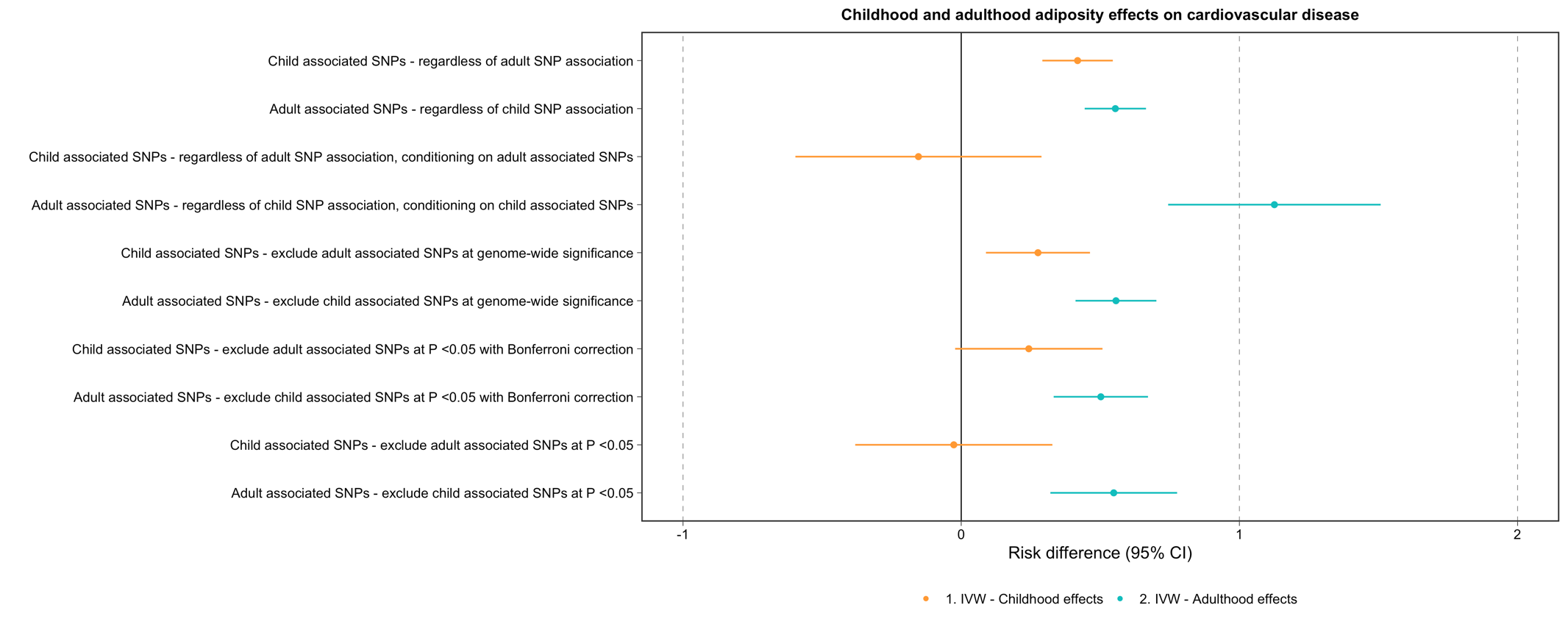


B


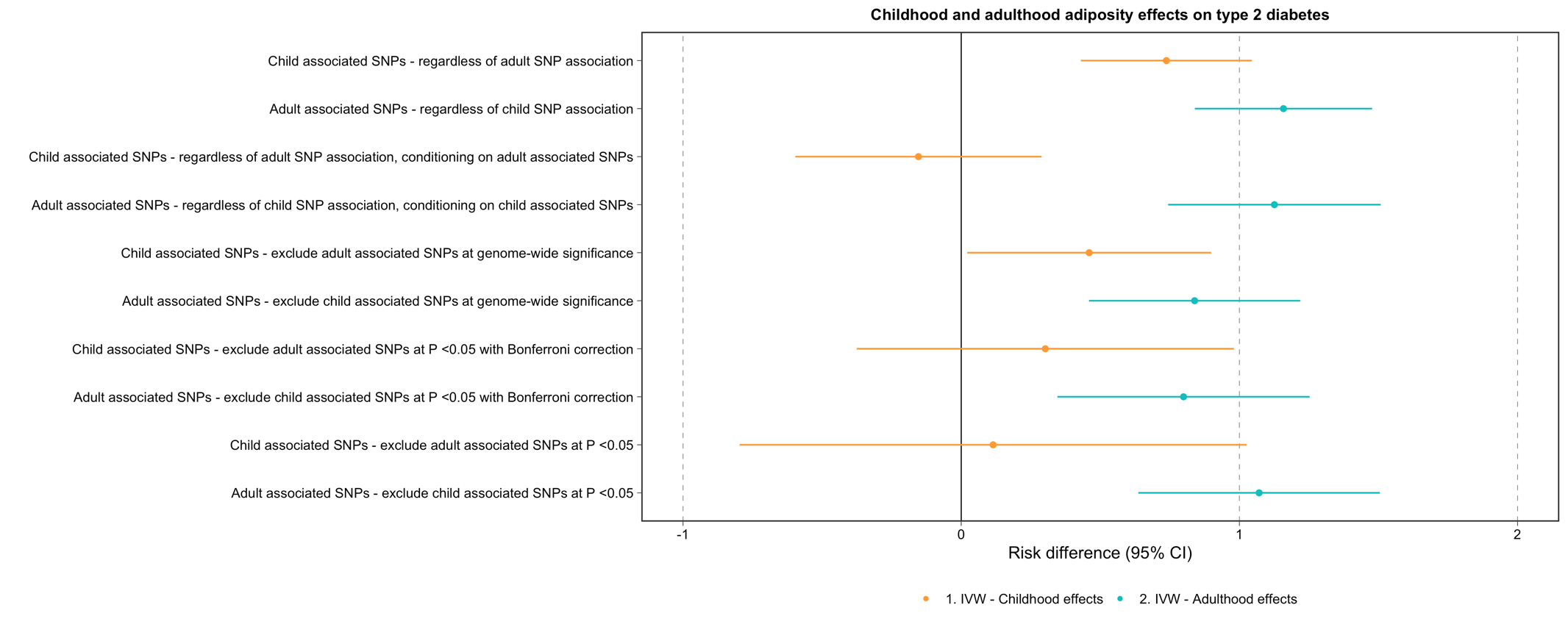


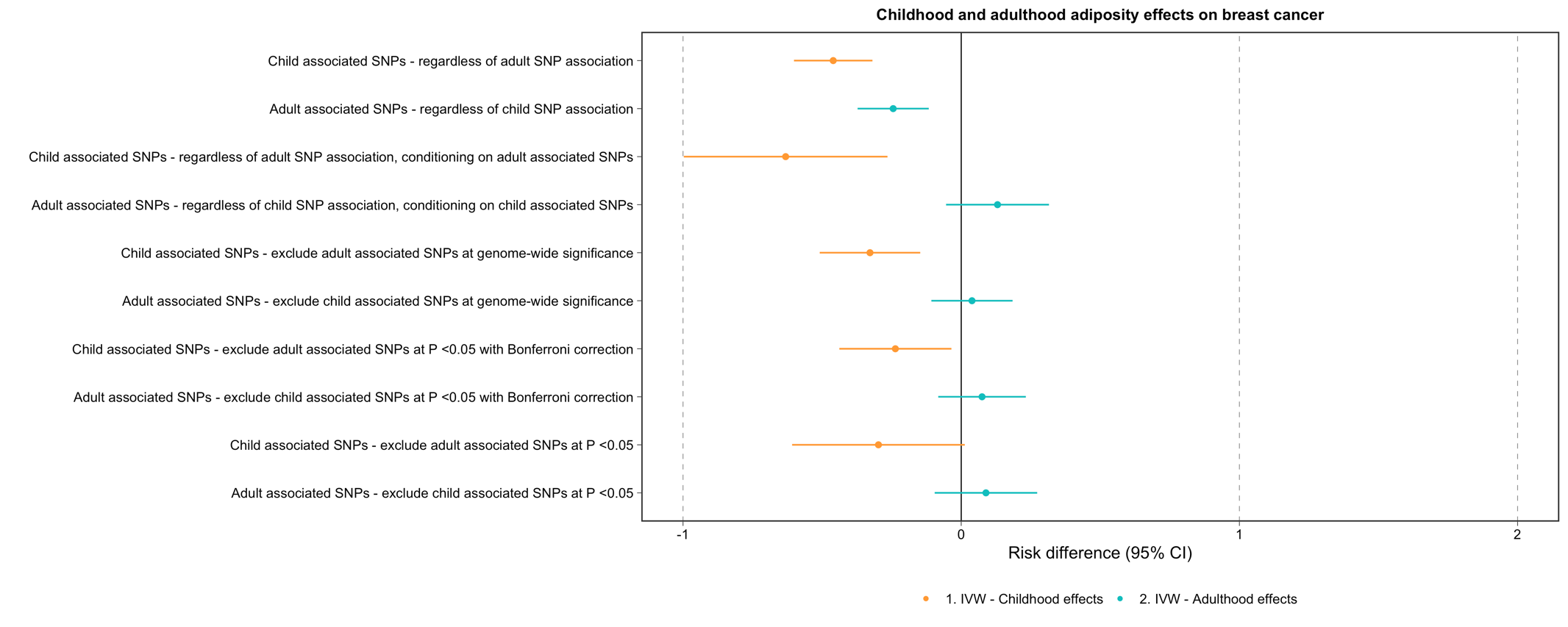


C

**References**

1. Richardson TG, Mykkänen J, Pahkala K, Ala-Korpela M, Bell JA, Taylor K, et al. Evaluating the direct effects of childhood adiposity on adult systemic metabolism: a multivariable Mendelian randomization analysis. Int J Epidemiol. 2021.

2. Brandkvist M, Bjørngaard JH, Ødegård RA, Åsvold BO, Davey Smith G, Brumpton B, et al. Separating the genetics of childhood and adult obesity: a validation study of genetic scores for body mass index in adolescence and adulthood in the HUNT Study. Hum Mol Genet. 2021;29(24):3966-73.

3. Robins J. A new approach to causal inference in mortality studies with a sustained exposure period—application to control of the healthy worker survivor effect. Mathematical Modelling. 1986;7(9):1393-512.

4. Daniel RM, Cousens SN, De Stavola BL, Kenward MG, Sterne JA. Methods for dealing with time-dependent confounding. Stat Med. 2013;32(9):1584-618.
